# Programmable Strategies for the Conversion of Aldehydes to Unsymmetrical (Deuterated) Diarylmethanes and Diarylketones

**DOI:** 10.1021/acs.orglett.5c00748

**Published:** 2025-03-28

**Authors:** Vipin R. Gavit, Nicole Hanania, Nadim Eghbarieh, Israa Shioukhi, Ahmad Masarwa

**Affiliations:** Institute of Chemistry, The Center for Nanoscience and Nanotechnology, and Casali Center for Applied Chemistry, The Hebrew University of Jerusalem, Jerusalem 9190401, Israel

## Abstract

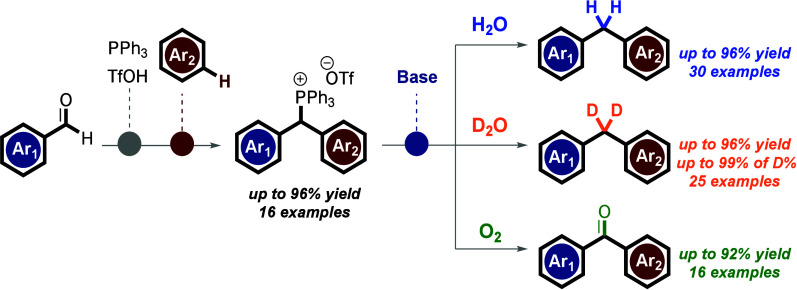

Herein, we present a versatile method for synthesizing unsymmetrical diarylmethanes and diarylketones from aldehydes and arenes. This involves: (1) regioselective Ar–H alkylation to form benzhydrylphosphonium salts via a one-pot, four-component reaction with simple reagents and (2) chemoselective reductions or oxidations of the benzylic C–P bond. Notably, reductions with D_2_O yield fully deuterated diarylmethanes. This high-yielding, straightforward approach offers valuable building blocks and enables novel transformations for academic and pharmaceutical research.

Diarylmethanes and diarylketones have attracted considerable interest due to their unique structural, chemical, and physical properties.^1−7^ They are essential in the synthesis of natural products, bioactive compounds,^3^ pharmaceuticals,^4^ agrochemicals, and modern synthetic materials.^4,5,7,8^ An important focus in organic synthesis is the modular and efficient construction of these scaffolds, particularly their unsymmetrical variants containing two distinct aryl groups.^1,2,5,6,9^

Considerable efforts have been dedicated to the synthesis of diarylmethanes and diarylketones using methods such as Friedel–Crafts-type reactions,^6,10^ Grignard-type reactions,^11−13^ and transition-metal-catalyzed cross-coupling reactions,^14−16^ including Suzuki–Miyaura,^16−18^ Negishi,^19^ Kumada,^2,12,20,21^ and Fukuyama couplings (see Scheme 1).^2,5,18,20−23^ While these elegant methods play a pivotal role in chemical transformations, they are often limited by challenges such as regioselectivity, air and moisture sensitivity, and the use of excess amounts of reagents, which are associated with the use of additives. Additionally, these reactions are frequently incompatible with unprotected functional groups.^1,24^

**Scheme 1:**
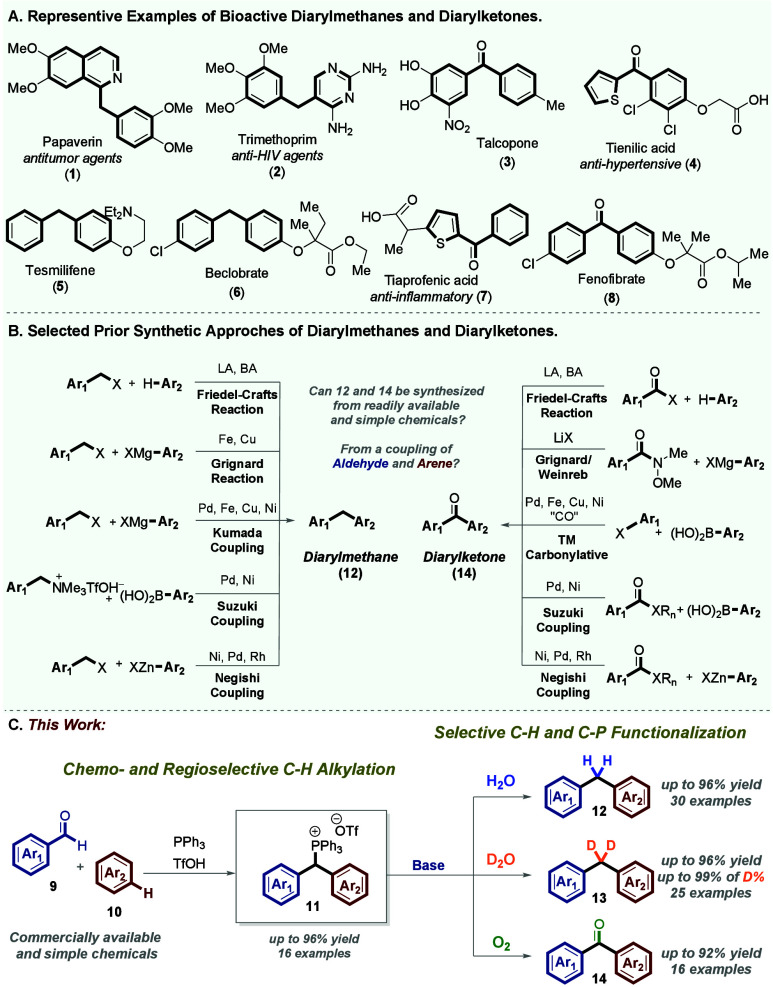
Overview of the Work

Although several promising state-of-the-art approaches have been introduced,^1,6,24^ there remains a need for the development of a simple, complementary, and comprehensive methodology for the site-selective and substrate-oriented synthesis of unsymmetrical diarylmethane and diarylketone frameworks.^9,18^ Importantly, there is growing demand for methodologies that enable the synthesis of rare yet valuable benzylic deuterium-isotope-labeled diarylmethanes.^25−27^

As part of our overarching strategies to synthesize a diverse range of diarylated-based bioactive compounds (**1**–**8**; Scheme 1A) encompassing diarylmethane and diarylketone derivatives, we identified organophosphonium salts (i.e., **11**) as versatile core scaffolds for these molecules (Scheme 1C).^7,18,28−30^

Organophosphonium salts, characterized by the presence of C–^+^P moieties, particularly organophosphorus-based Wittig salts, are among the most widely utilized reagents in organic synthesis for constructing C–C double bonds.^28,29,31−35^ In recent years, innovative methods for functionalizing the C–^+^P bond have emerged, employing organophosphonium compounds in novel ways to enable the formation of new chemical bonds, such as C–O, C–N, C–S, and C–C bonds.^28,33,34,36,37^

In this context, our research group^28,29^ and others^32,33,37−39^ recently developed a versatile method for synthesizing benzhydryl triarylphosphonium salts (**11**) via a convenient one-pot, regioselective four-component coupling reaction using readily available starting materials (Scheme 1C).^28,29^ These benzhydryl phosphonium salt building blocks (**11**) demonstrated exceptional reactivity and utility as evidenced by their selective post-functionalization at the C(sp^3^)–^+^PPh_3_ position.^28,29,33,34,37^ In terms of reactivity, they have been shown to undergo photo- and radical-mediated^34,35,37^ reactions and can also act as C-electrophilic reactive groups,^28,33,34^ engaging in reactions via a S_N_1 mechanism.^29,33,37^ These approaches enabled a range of transformations, including aminations,^28^ thiolations,^28^ arylations,^28,33^ alkenylation,^37,40^ and alkylation,^37,38^ to produce structural motifs commonly found in pharmaceuticals and agrochemicals.^28^

We envision that the research on these triarylphosphonium salts (**11**) can be further extended to explore new areas of their reactivity and applications in synthesis.^28,29^ We realize that one way to achieving this is by applying them to reduction and oxidation reactions, which could lead to the synthesis of important unsymmetrical (deuterated) diarylmethanes and diarylketones. In fact, this approach can be considered as a programmable, sequential two-step strategy to synthesize these diarylmethanes and diarylketones from simple starting materials, such as aldehydes and arenes.^28,29^

Here, we present a general and operationally straightforward approach for preparing phosphonium salts **11** and their late-stage transformation into various (deuterated) diarylmethanes (**12** and **13**) and diarylketone (**14**) compounds.^28^ This is achieved through reduction and oxidation reactions of compound **11**, respectively, using only water (H_2_O or D_2_O) as a reductant and O_2_ as an oxidant (Scheme 1C).^29^

Building upon our previous work^28,29^ as well as that of Lin and others,^40^ we initiated our studies with the development of a facile, mild, and efficient synthesis of benzhydryl triarylphosphonium salts (**11**).^28,29,32^ For further details and discussion on the preparation of new benzhydryl triarylphosphonium salts (**11a**–**11p**), please refer to pages S7–S9 of the Supporting Information.

Next, we aimed to demonstrate the synthetic utility of these phosphonium salt products (**11**) through a selective reduction of the C–^+^P bonds, affording the important (unsymmetrical) diarylmethane scaffolds **12**.^29^ In this regard, a hydrolysis-based reduction protocol has been developed for benzhydryl triarylphosphonium salts **11** using H_2_O (Scheme 2A and B).^29^ In this method, we explored the reduction of a benzhydryl triarylphosphonium salt, specifically utilizing compound **11t** as a standard salt for reaction optimization. Various organic and inorganic bases were employed for the reaction, and the Supporting Information provides further details on these bases (see Table S2 and page S28 of the Supporting Information). Ultimately, KOH was identified as the optimal base for the hydrolytic reduction of benzhydryl triarylphosphonium salt, leading to the formation of diarylmethane **12i** with an isolated yield of 87% (Scheme 2A and B).^29^

**Scheme 2:**
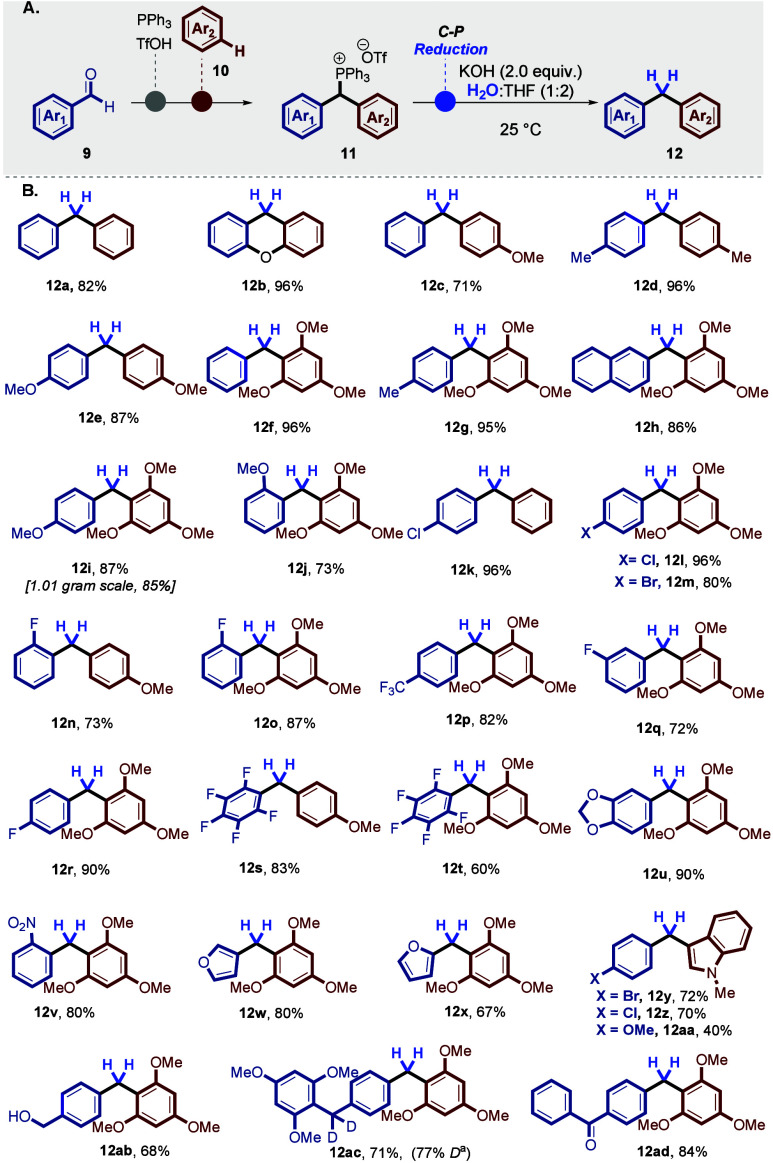
Reduction (Hydrolysis) of the C–P Bond Reaction conditions: The reaction was performed with compound **11** (0.2 mmol) and KOH (0.4 mmol) in 3 mL of H_2_O/THF (1:2, v/v) at 25 °C for a duration of 1–5 h, under an open atmosphere. Yields of isolated products are given.

Subsequently, we successfully synthesized a variety of unsymmetrical diarylmethane derivatives **12a**–**12ad** by adapting the standard reaction conditions outlined in Scheme 2A and B. The reaction exhibited a broad scope, encompassing aryls with substituents, such as −OMe, −CH_2_OH, −F, −Cl, −Br, −Ph, −NO_2_, −CF_3_, pentafluoro, 1,3-dioxole, and −Me, with good yields. Notably, even benzhydryl triarylphosphonium salts containing heteroarenes, such as furan and indoles, yielded the desired diarylmethane derivatives (**12w**–**12aa**, respectively) with good yields.

Additionally, benzhydryl triarylphosphonium salt **11d** was subjected to the standard reaction conditions, yielding xanthene derivative **12b** with a yield of 96%. Furthermore, the reduction of salts **11o** and **11p** successfully produced polyarylated frameworks **12ac** and **12ad**, respectively, in good yields. Additionally, the reduction of benzhydryl triphenylphosphonium salt **11t** was achieved, affording diarylmethane **12i** on a 1.01 g scale (1.45 mmol, 85%) (for synthesis details, see page S37 of the Supporting Information).

Deuterium-labeled compounds are essential in chemistry and life sciences, particularly in drug discovery.^25,27,29,41^ As a stable hydrogen isotope, deuterium aids in mechanistic studies, mass spectrometry, and deuterated drug development.^25,26,41^ Benzylic positions are prone to oxidative metabolism, but the greater stability of C–D bonds can influence drug absorption, distribution, metabolism, and excretion.^25−27,41,42^ Thus, significant efforts focus on late-stage benzylic deuteration.^26,42^ Transition-metal-catalyzed C–H activation and hydrogen transfer reactions are widely used, but selective benzylic deuteration remains challenging due to competing C–H labeling, especially without directing heteroatoms.^25−27,41,42^

Encouraged by our results on C–P bond hydrolysis, we anticipated that benzhydryl triarylphosphonium salts **11** could offer a valuable opportunity for benzylic deuterium labeling, not just once but twice, allowing the incorporation of two deuterium atoms. We hypothesize that this can be achieved through the synergistic deuterolysis of the benzylic C–H and C–P bonds.^29^

Replacing water with D_2_O enabled the synthesis of fully α-deuterated unsymmetrical diarylmethanes (**13**) with excellent deuterium incorporation (up to 99%) and good yields (Scheme 3A and B).^29^ Notably, we successfully synthesized D-labeled unsymmetrical diarylmethanes **13** bearing *ortho*-, *meta*-, and *para*-substituted halides, including −F (**13k**–**13n**), −Cl (**13h** and **13i**), and −Br (**13j** and **13u**). The reaction was also effective in affording D-labeled unsymmetrical diarylmethanes with various functional groups, such as −OMe (**13b**–**13e**), −NO_2_ (**13s**), −CF_3_ (**13o**), indole (**13u**–**13w**), 1,3-benzodioxole (**13x**), furan (**13t**), and xanthen derivative (**13y**).

**Scheme 3:**
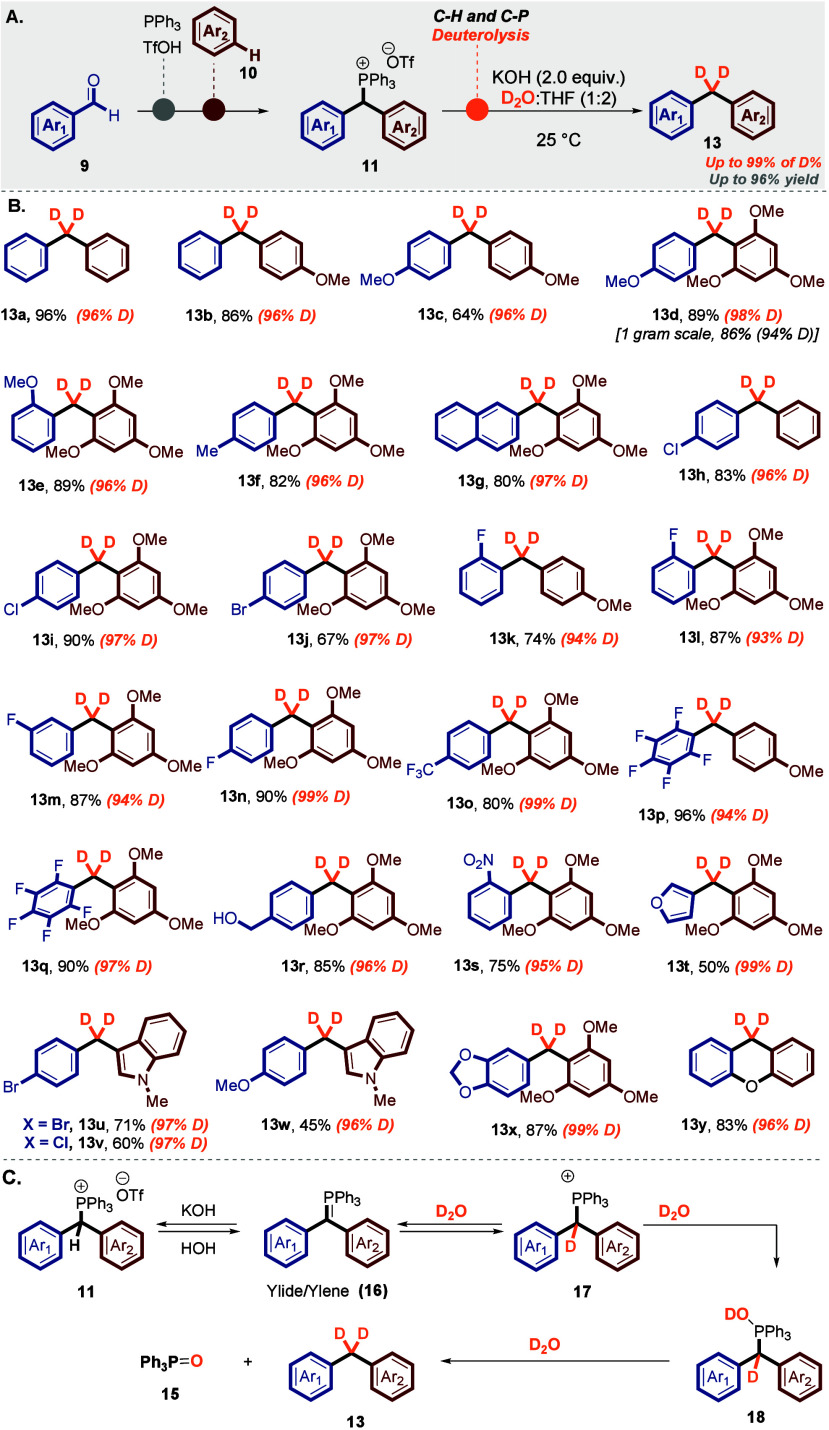
Metal-Free Reduction (Deuterolysis) of the Benzylic C–H and C–P Bonds The reaction was performed with compound **11** (0.2 mmol) and KOH (0.4 mmol) in 3 mL of D_2_O/THF (1:2, v/v) at 25 °C for a duration of 1–5 h, under an open atmosphere. Compound **13p** needed 3 days for full conversion. Yields of isolated products are given. The percentage of D incorporation for all deuterated molecules was assessed using ^1^H nuclear magnetic resonance (NMR).

Furthermore, we performed the deuterolysis of benzhydryl triphenylphosphonium salt **11t**, yielding diarylmethane (**13d**) on a gram scale (1.43 mmol) with deuterium incorporation (94% D) (for detailed synthesis information, please refer to page S55 of the Supporting Information).

A proposed mechanism for the deuterolysis (and hydrolysis)-based reduction pathway is shown in Scheme 3C.^29^ Based on literature reports,^28,29^ this process involves the formation of ylide/ylene **16**, followed by protonation or deuteration of ylene **16**.^29^ The subsequent cleavage of the C(sp^3^)–P^+^ bond occurs via P-selective nucleophilic substitution (see **16** to **13**), releasing triphenylphosphine oxide (Ph_3_P=O, **15**).^28,29,31^

To further support this mechanism through the generation of the ylene **16** intermediate, a hydrolysis–reduction reaction of the benzylic-deuterated benzhydryl triarylphosphonium salt **11a**_*d*_ with H_2_O was performed, affording the hydrolysis product with complete loss of deuterium incorporation (falling to 0%) (for detailed synthesis information, please refer to page S48 of the Supporting Information).^29^

Next, we were contemplating the possibility of synthesizing important unsymmetrical diarylated ketone motifs using the benzhydryl triarylphosphonium salts **11** (Scheme 4).^29,43^ We imagined that this could be achieved by the generation of phosphorus ylide (**16**) from salt **11**, followed by a direct oxidation of this ylide (**16**).^29,44−46^

**Scheme 4:**
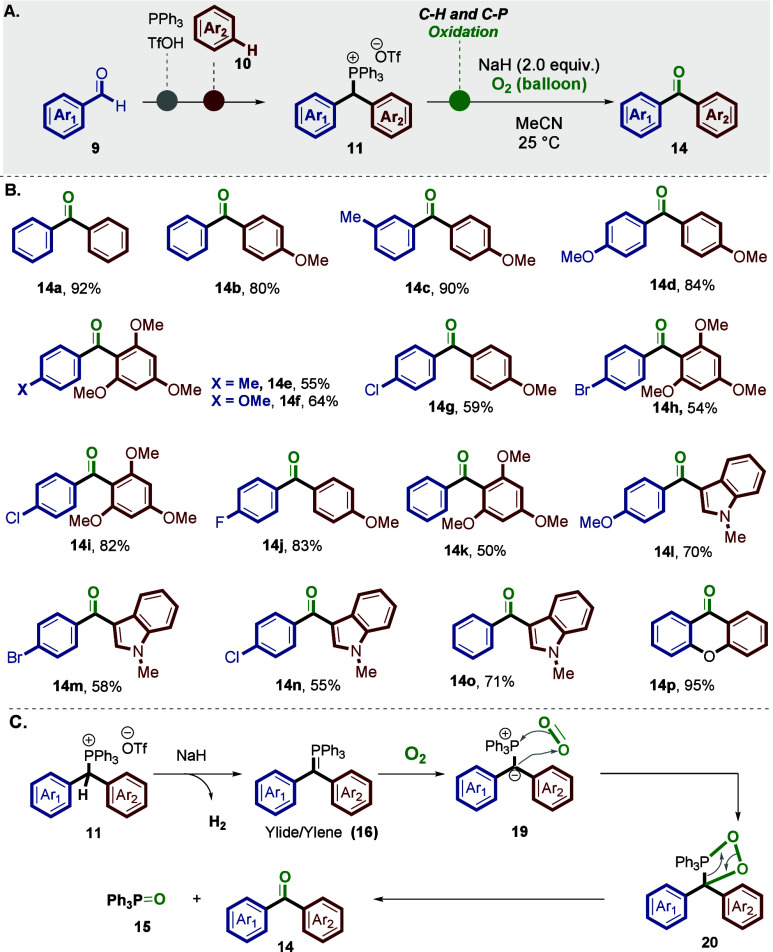
Oxidation of the Benzylic C–H and C–P Bonds The reaction was performed with compound **11** (0.2 mmol) and NaH (0.6 mmol) under O_2_ (balloon) in 3 mL of MeCN at 25 °C for 5 h. Yields of isolated products are given.

Our initial optimization efforts focused on the formation of benzophenone **14a** from benzhydryl triarylphosphonium salt **11q** and a base under O_2_ (balloon), via the corresponding ylide **16**.^29,44,46,47^ After many experiments, the use of NaH in dry acetonitrile (CH_3_CN) was found to be the best reaction condition to obtain ketone product **14a** with a yield of 92%.

Next, we explored the reaction’s scope, and we employed identical reaction conditions. Consequently, benzhydryl triarylphosphonium salts **11** effectively participated in the reaction, resulting in the formation of the desired product **14**, as depicted in Scheme 4A and B, with satisfactory yields. Accordingly, the oxidation reaction was effective across a range of benzhydryl triarylphosphonium salts with different substituted groups, including −Me (**14c** and **14e**), −OMe (**14c**–**14l**), −Cl (**14g**, **14i**, and **14n**), −Br (**14h** and **14m**), and −F (**14j**). Notably, the reaction also proceeded efficiently with benzhydryl triarylphosphonium salts containing heteroarene groups, such as indole (**14l**–**14o**) and xanthone (**14p**).^29^

The proposed mechanism for the oxidation of benzhydryl triarylphosphonium salts (**11**) is illustrated in Scheme 4C.^28,29,31,46,47^ The process begins with the abstraction of an acidic proton from the benzylic position of compound **11**, generating the corresponding ylide species (**16**).^28,29,31,46,47^ This ylide intermediate **16** then undergoes a concerted addition reaction with molecular oxygen (O_2_), leading to the formation of a 1,2-dioxetane intermediate (**20**).^29,46,47^ Finally, this intermediate collapses, yielding the desired diarylketone (**14**) and triphenylphosphine oxide (**15**) as a byproduct.^29,46,47^

In summary, we have developed a straightforward and programmable protocol for the direct selective synthesis of a diverse range of unsymmetrical diarylmethanes and diarylketones, structural motifs found in many important chemicals.^48^ These protocols involve a site-selective, one-pot, four-component coupling reaction of commercially available aldehydes and arenes with Ph_3_P and TfOH, yielding benzhydryl triarylphosphonium salts. The utility of these salts as versatile building blocks was demonstrated through the chemoselective post-functionalization of the benzylic C(sp^3^)–H and C(sp^3^)–PPh_3_ bonds, enabling divergent reduction and oxidation reactions. Notably, this strategy also provides access to fully benzylic-dideuterated unsymmetrical diarylmethanes, a previously challenging class of compounds. The synergistic C–H and C–P post-functionalization strategy is operationally simple and metal-free, utilizing mild and friendly reagents, such as H_2_O/D_2_O and O_2_, as sources for reduction and oxidation, respectively, affording products in good yields. These protocols should significantly streamline the synthesis of bioactive molecules and broaden their applications across various fields.

## Data Availability

The data underlying this study are available in the published article and its Supporting Information.

## Supporting Information Available

Experimental procedures, product characterization data, and NMR spectra (PDF)
