# Supplementary material for: Programmable Strategies for the Conversion of Aldehydes to Unsymmetrical (Deuterated) Diarylmethanes and Diarylketones
Source: Org Lett. 2025 Mar 28;27(14):3637–42. doi: 10.1021/acs.orglett.5c00748 (PMC11998069; doi:10.1021/acs.orglett.5c00748)
Supplement: Supplementary file 1 — ol5c00748_si_001.pdf [file ol5c00748_si_001.pdf]

Supporting Information

Programmable Strategies for the Conversion of Aldehydes to Unsymmetrical (Deuterated) Diarylmethanes and Diarylketones

Vipin R. Gavit,[&] Nicole Hanania,[&] Nadim Eghbarieh, Israa Shioukhi, and Ahmad Masarwa*

Institute of Chemistry, The Center for Nanoscience and Nanotechnology, and Casali Center for Applied Chemistry, The Hebrew University of Jerusalem, Jerusalem, 9190401, Israel

[&]Contributed equally to the work

*Corresponding author: E-mail: Ahmad.Masarwa1@mail.huji.ac.il

Table of Contents

1. Notes…………………………………………………….………………..……..……......S3

1.1. Materials and General Remarks……………………...……………………..……....S3

2. Methods……………………….……………………………………………..............S4

2.1 Information and preparation for Strating Martials……………………………………..S4

2.2 Benzhydryl triarylphosphonium salts (**11**)……...…...…………………………….…......S7

2.2.1 Results and discussion on the synthesis of new examples of benzhydryl triarylphosphonium salts (**11**)……………………………………………………………S7

2.2.2 General Procedure-A and Characterization for Phosphonium Salts (**11**)..…...…....S9

2.3 General Procedure-B and Characterization for Diarylmethanes Products (**12**)…..……S27

2.4 NMR analysis for the reduction reaction of (**11ad**) to product (**12i**)…………………....S48

2.5 General Procedure-C and Characterization for Diarylmethanes-D_2 Products (**13**)..........S50

2.6 Sequential alkylation deuterolysis and hydrolysis of compound (**12ac**)………………..S67

2.7 General Procedure-D and Characterization for Diarylketones Products (**14**)......…........S68

2.8 Control experiment for the oxidation reaction…………………………….……….S79

3. NMR Spectra ……………..…...……………………………………………...…S80

3.1 Reaction progress of site-selective oxidation of product (**14i**)………………………..S183

4. References………………………………………………………….....…...………S184

1. Notes:

1.1. Materials and General Remarks

Unless stated otherwise, reactions were performed in oven-dried glassware/vials fitted with either rubber septa or plastic screw cap and were stirred with Teflon-coated magnetic stirring bars. Commercially obtained reagents such as trifluoromethanesulfonic acid (TfOH), PPh_3, aldehydes and arene were used as received. Commercial grade solvents, i.e., acetonitrile, diethyl ether, *n*-Pentane, Ethyl acetate, dichloromethane, methanol, and ethanol were used as received unless mentioned otherwise. Thin-layer chromatography was performed using silica gel 60 F-254 precoated plates (0.25 mm) and was visualized by UV irradiation, CAM stain, $KMnO_4$ stain, and other stains. Silica gel of particle size 230 – 400 mesh was used for Column chromatography. ^{1}H and ^{13}C NMR spectra were recorded using 400 and 500 MHz spectrometers with ^{13}C operating frequencies of 100 and 125 MHz, respectively. ^{31}P and ^{19}F operation frequencies were 162 MHz, 203 MHz and 376 MHz, 471 MHz, respectively. Chemical shifts (δ) are reported in ppm relative to the residual solvent ($CDCl_3$) signal (δ = 7.26 for ^{1}H NMR and δ = 77.16 for ^{13}C NMR), (DMSO–d_6) signal (δ = 2.50 (p) for ^{1}H NMR; δ = 39.52 (septet) for ^{13}C NMR), (CD_3CN) signal (δ = 1.96 (p) for ^{1}H NMR; δ = 118.26 for ^{13}C NMR). Data for ^{1}H NMR spectra are reported as follows: chemical shift (multiplicity, coupling constants, and the number of hydrogen). Abbreviations are as follows: s (singlet), d (doublet), t (triplet), q (quartet), m (multiplet), dd (doublet of doublet), dt (doublet of triplet), ddd (doublet of doublet of doublet) brs (broad singlet). High-Resolution Mass Spectrometry (HRMS) were recorded on SCIEX X500R QTOF spectrometer using acetonitrile as solvent.

2. Methods

2.1. Information and preparation for Strating Martials

All starting materials of aldehydes **9a** – **9k**, **9p**, and arenes **10a** – **10d**, were purchased from a commercial source and used as received unless otherwise noted. Compounds **9d**, **9l**, and **9o** were prepared according to a literature reported procedure.[1-3]

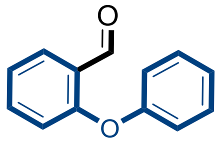

9d

2-phenoxybenzaldehyde (9d):

Prepared according to previously reported procedure.[1]

Compound (**9d**) was isolated in (90% yield) as a colorless oil.

^{1}H NMR (400 MHz, $CDCl_3$) δ: 10.52 (d, J = 0.8 Hz, 1H), 7.94 (dd, J = 7.8, 1.9 Hz, 1H), 7.50 (ddd, J = 8.4, 7.2, 1.9 Hz, 1H), 7.42 - 7.35 (m, 2H), 7.22 - 7.15 (m, 2H), 7.10 - 7.04 (m, 2H), 6.90 (dd, J = 8.4, 1.0 Hz, 1H).

The spectral data are consistent with those reported in the literature. [2]

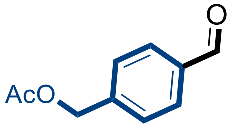

9l

4-formylbenzyl acetate (9l):

Prepared according to previously reported procedure. [3]

Compound (**9l**) was isolated in (60% yield) as a colorless oil.

^{1}H NMR (400 MHz, $CDCl_3$) δ: 10.00 (s, 1H), 7.88 - 7.85 (m, 2H), 7.52 - 7.48 (m, 2H), 5.17 (s, 2H), 2.13 (s, 3H).

The spectral data are consistent with those reported in the literature. [3]

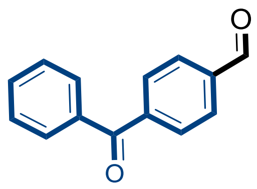

9o

4-benzoylbenzaldehyde (9o):

Prepared according to previously reported procedure. [4]

Compound (**9o**) was isolated in (80% yield) as a white solid.

^{1}H NMR (400 MHz, $CDCl_3$) δ: 10.13 (s, 1H), 8.04 - 7.99 (m, 2H), 7.95 - 7.91 (m, 2H), 7.84 – 7.79 (m, 2H), 7.66 - 7.60 (m, 1H), 7.55 – 7.48 (m, 2H).

The spectral data are consistent with those reported in the literature. [4]

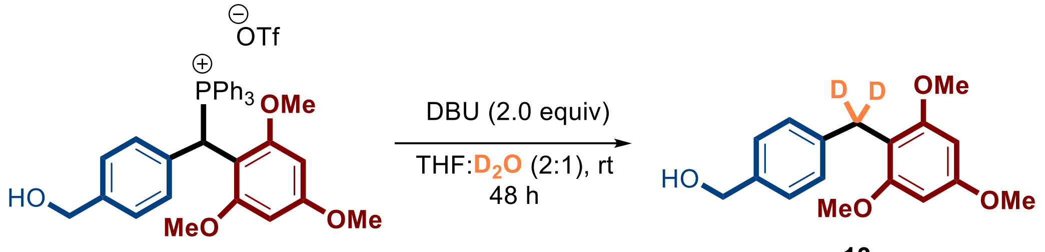

(4-((2,4,6-trimethoxyphenyl)methyl-d2)phenyl)methanol (13r):

A 7 mL screw-cap vial containing a Teflon-coated magnetic stirring bar was charged with benzhydryl phosphonium salt **11m** (0.9 mmol, 1.0 equivalent, prepared according general Procedure-A), 4 mL of THF, and 20.0 equivalent of D_2O. The mixture was stirred at room temperature for five minutes. DBU (1.8 mmol, 2.0 equivalents) was then added in a single portion. The progress of the reaction was monitored using thin-layer chromatography (TLC). The reaction mixture was quenched with H_2O, and the aqueous phase was extracted with EtOAc (2 x 7 mL). The combined organic phases were dried over Na_2SO_4, filtered and concentrated under reduced pressure (by evaporator). The crude material was purified by column chromatography, yielding the desired product (**13r**) in (200 mg, 76% yield) as a colorless oil. 77% Deuterium-incorporation.

R_f = 0.43 (30% EtOAc in hexane).

^{1}H NMR (400 MHz, $CDCl_3$) δ 7.17 - 7.08 (m, 4H), 6.06 (s, 2H), 4.50 (s, 2H), 3.90 - 3.80 (m, 0.44H), 3.71 (s, 3H), 3.69 (s, 6H).

^{13}**C NMR** (101 MHz, $CDCl_3$) δ 159.7, 158.9, 141.9 (d, J = 4.0 Hz), 137.9, 128.7, 127.0, 110.1 (d, J = 2.3 Hz), 90.7, 65.5, 55.8, 55.42, 28.2 – 27.4 (m).

HRMS (ESI) was calculated for $[C_{17}H_{18}D_2O_4 + H]^+$ $[M + H]^+$: m/z 291.1559, found = 291.1554.

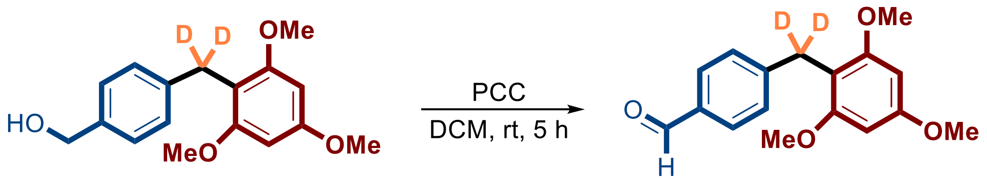

13r → **9n**

4-((2,4,6-trimethoxyphenyl)methyl-d2)benzaldehyde (9n):

An oven dry flask containing a Teflon-coated magnetic stirring bar was charged with **13r** (0.6 mmol, 1.0 equivalent), and added DCM (8 mL). The mixture was stirred at room temperature for five minutes. PCC (0.72 mmol, 1.20 equivalents) was then added in a single portion. The progress of the reaction was monitored using thin-layer chromatography (TLC). The reaction mixture was quenched with H_2O, and the aqueous phase was extracted with EtOAc (2 x 10 mL). The combined organic phases were dried over Na_2SO4, filtered and concentrated under reduced pressure (by evaporator). The crude material was purified by column chromatography, yielding the desired product (**9n**) in (132 mg, 76% yield) as a colorless oil. 77% Deuterium-incorporation.

R_f = 0.46 (30% EtOAc in hexane).

^{1}**H NMR** (400 MHz, $CDCl_3$) δ 9.92 (s, 1H), 7.74 - 7.71 (m, 2H), 7.40 - 7.36 (m, 2H), 6.15 (s, 2H), 4.00 - 3.96 (m, 55H), 3.81 (s, 3H), 3.79 (s, 6H).

^{13}**C NMR** (101 MHz, $CDCl_3$) δ 192.3, 160.1, 158.9, 150.1 (d, J = 4.4 Hz), 134.2, 129.8, 129.2, 108.9 (d, J = 2.4 Hz), 90.7, 55.8, 55.4, 28.9 – 28.3 (m).

HRMS (ESI) was calculated for $[C_{17}H_{16}D_2O_4]^+$ $[M]^+$: m/z 288.1325, found = 288.1318.

2.2 Benzhydryl triarylphosphonium salts (11):

2.2.1. Results and discussion on the synthesis of new examples of benzhydryl triarylphosphonium salts (11):

First, the aldehyde (**9**) undergoes activation, leading to the formation of the hydroxyl-triphenylphosphonium[5] intermediate (**11-Int**). This intermediate then undergoes coupling with the arene derivatives (**10**) via Friedel–Crafts-type reactions (Scheme S1A).[6] Based on this foundation, we aimed to generalize this approach to achieve new chemo- and regioselective coupling of (hetero)arene derivatives (**10**) with aldehydes (**9**), leading to new examples of benzhydryl triarylphosphonium salts (**11**).

The four-component coupling alkylation reaction proceeded as follows: aldehyde (**9**) was treated with arene (**10**), triphenylphosphine (PPh_3), and triflic acid in CH_3CN at 45-80 °C for 24-72 hours, yielding the desired phosphonium salts (**11**) in up to 96% yield and exclusive site-selectivity (Scheme S1).[6,7]

Following this procedure, a series of new phosphonium salts 11a-p were synthesized from different aldehyde derivatives (**10**) bearing electron-donating groups (EDG; e.g., **11ad**, **11f**, **11l-m**, **11o**), as well as derivatives with electron-withdrawing groups (EWD, **11c**, **11g-k**, **11p**).[6] In addition, 1,3,5-trimethoxybenzenes (**11ad**, **11e-11m**,**11o-11p**), and different types of aromatic arenes (**11b-d**, **11n**, Scheme S1) were explored. In all cases, the Friedel-Crafts phosphonium alkylation-type reaction occurs selectively para- to the EDG of arene **10** (For more synthesized examples of **11** see Table S2, Page S9).

The reaction showed high functional group tolerance as evidenced by fluorine- (**11c**, **11h-k**), ether- (**11f**), nitro- (**11g**), and hydroxy-containing (**11m**) substrates (Scheme S1B). Similarly, the deuterated salt **11a*d*** was prepared in good yield using deuterated *p*-anisaldehyde (**9a*d***). Importantly, a selective intramolecular coupling/cyclization reaction of 2-phenoxybenzaldehyde **9d** under the given conditions afforded the exclusive cyclized product, xanthene benzhydryl triarylphosphonium salts **11d**, in good yield (for synthesis details, see page S15).

Scheme S1. Preparation of benzhydryl triarylphosphonium salts (11).

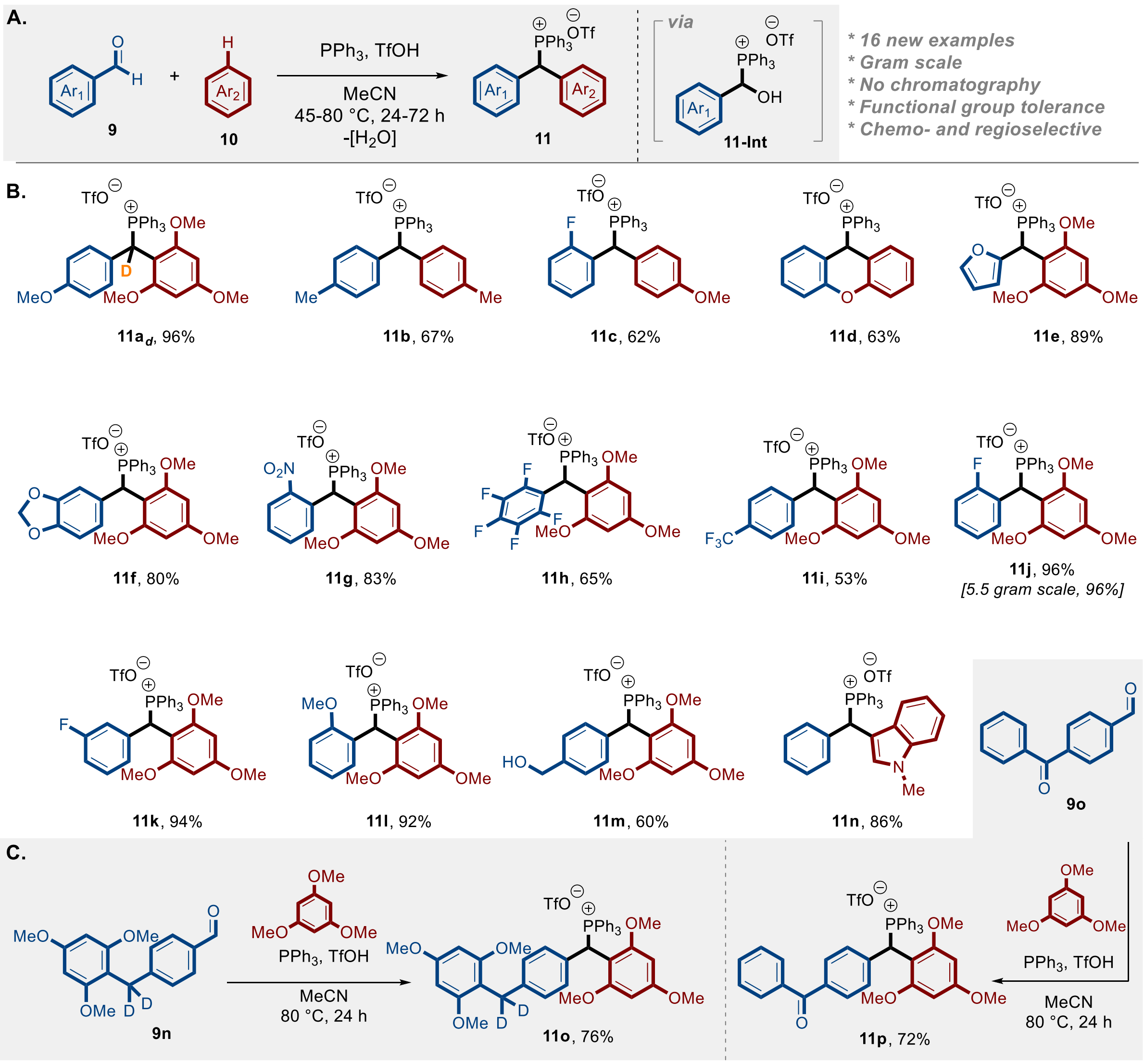

Reaction conditions: A mixture of PPh_3 (0.80 mmol; 1.1 equiv), MeCN (2 mL), and TfOH (0.87 mmol; 1.2 equiv) was prepared. The reaction mixture was stirred for 5 minutes, then charged with 9 (0.73 mmol; 1.0 equiv) and 10 (0.73 mmol; 1.0 equiv) at room temperature, and the reaction was heated to 45-80 ºC. Yields of isolated products are given.

Moreover, most of these products (**11**) were purified by simple precipitation — an additional advantage. Additionally, phosphonium salts (**11**) containing heteroarenes were successfully synthesized from readily available furan aldehydes (in **11e**) and indole arene (in **11n**) by heating the reaction to 80 °C. Using the same selective approach, we successfully synthesized a poly-aromatic-containing benzhydryl triarylphosphonium salts **11o-p** by reacting diarylmethane-aldehydes (**9n** and **9o**) with 1,3,4-trimethoxybenzene (**10a**, Scheme S1C).

To demonstrate the practicality, and scalability of this alkylation reaction, phosphonium salt **11j** was synthesized on a 5.5 gram-scale under the same reaction conditions, achieving a 96% yield. (For synthesis details, see Page S19).[6]

2.2.2 General Procedure-A and Characterization for Phosphonium Salts (11)

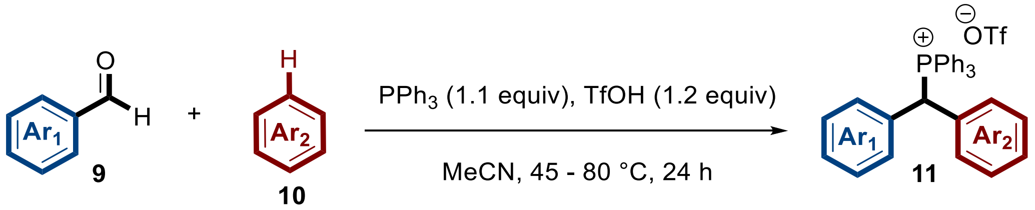

According to a literature-reported procedure, [6,7] an oven-dried, screw-capped 15 mL vial equipped with a Teflon-coated magnetic stirring bar was charged with PPh_3 (0.80 mmol, 1.1 equiv), MeCN (1 mL), and TfOH (0.87 mmol, 1.2 equiv). The reaction mixture was stirred at room temperature for 5 minutes. Subsequently, arene (**10**) (0.73 mmol, 1.0 equiv) and aldehyde (**9**) (0.73 mmol, 1.0 equiv) were added. The mixture was then stirred at either 45 °C or 80 °C in an oil bath for 24 hours. Upon completion the solvent was removed under reduced pressure (by evaporator). The resulting crude product was dissolved in 1.5 mL of a 1:1:1 mixture of EtOH, EtOAc, and DCM, followed by the addition of 10 mL of a 5:1 (Et_2O:pentane) solution. The mixture was left to stand for precipitation. After standing, the excess solvent was decanted, and the precipitate was repeatedly washed with diethyl ether to yield the phosphonium salt (**11**), which was used in the subsequent step without further purification.

Notes: (**1**) In specific cases, the salts underwent additional purification via column chromatography, as described in the characterization section.

Table S1: Scope of the phosphonium salts (**11**):

Entry	Aldehydes (9)	Arenes (10)	Tempreature °C	Products (11)	Yield[a]
1.	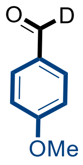 9a$_d$	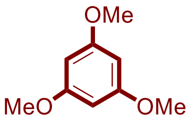 10a	80	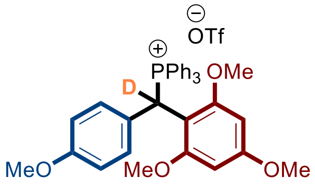 11a$_d$	96%

2.	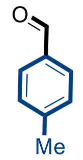 **9b**	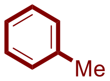 **10b**	80	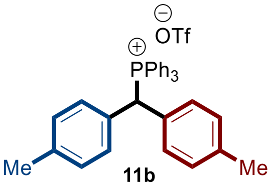 **11b**	67%
3.	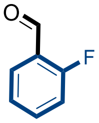 **9c**	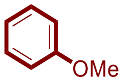 **10c**	45	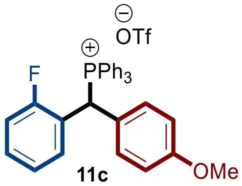 **11c**	62%
4.	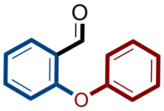 **9d**		45	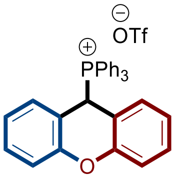 **11d**	63%
5.	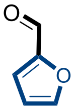 **9e**	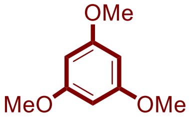 **10a**	80	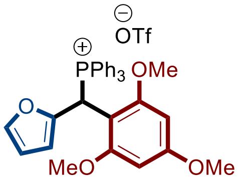 **11e**	89%
6.	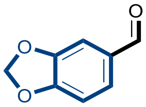 **9f**	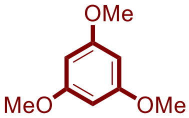 **10a**	80	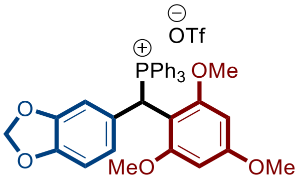 **11f**	80%
7.	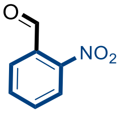 **9g**	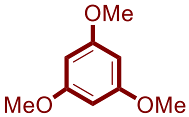 **10a**	80	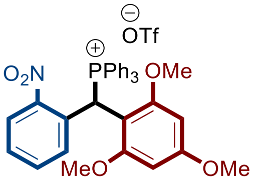 **11g**	83%
8.	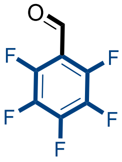 **9h**	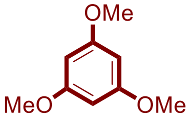 **10a**	80	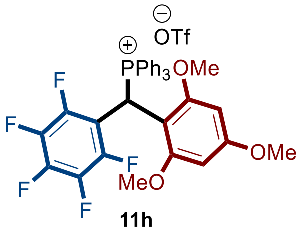 **11h**	65%
9.	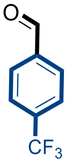 **9i**	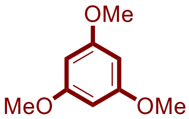 **10a**	80	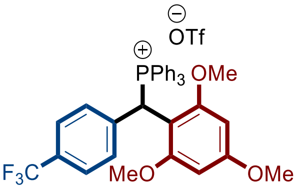 **11i**	53%

10.	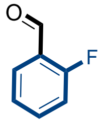 **9c**	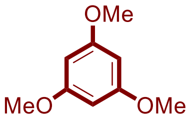 **10a**	45	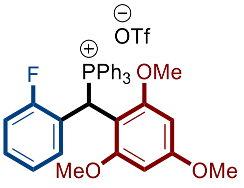 **11j**	96%
11.	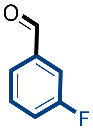 **9j**	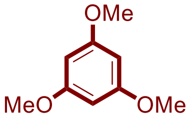 **10a**	80	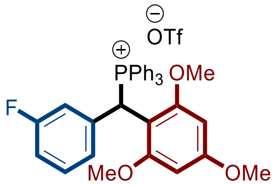 **11k**	94%
12.	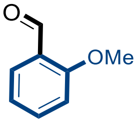 **9k**	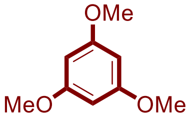 **10a**	80	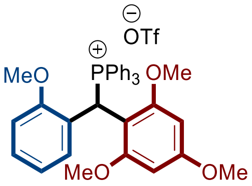 **11l**	92%
13.	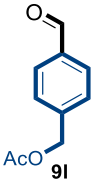 **9l**	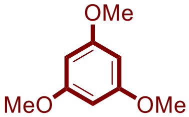 **10a**	80	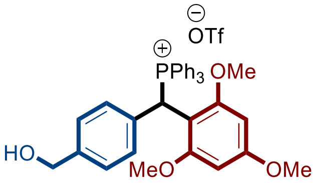 **11m**	60%
14.	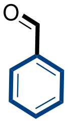 **9p**	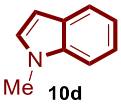 **10d**	80	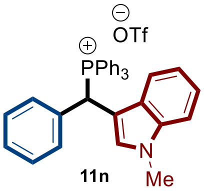 **11n**	86%
15.	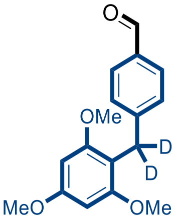 **9n**	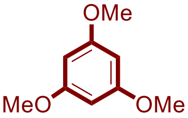 **10a**	80	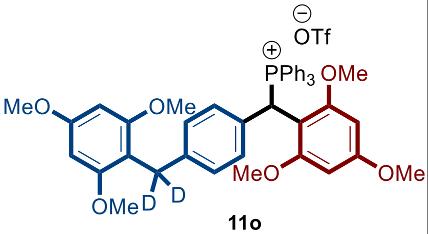 **11o**	76%
16.	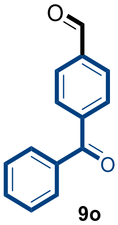 **9o**	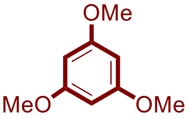 **10a**	80	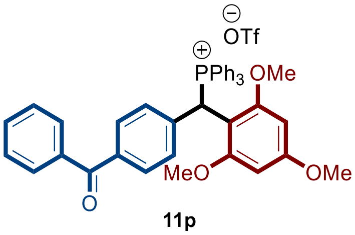 **11p**	72%

[a] Isolated yield.

Previously Reported benzhydryl phosphonium salts (11):

Note: Compounds **11q-11ai** were prepared following general Procedure-A. The spectroscopic data for the benzhydryl phosphonium salt align with those previously reported in the literature. [6, 8]

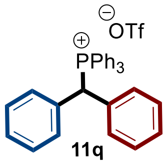

11q

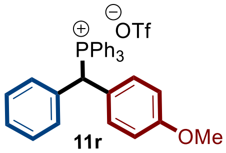

11r

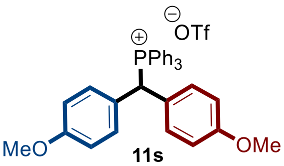

11s

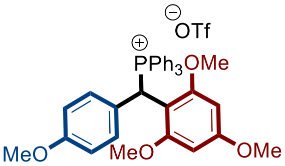

11t

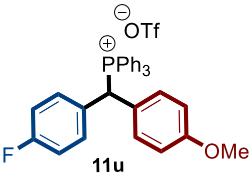

11u

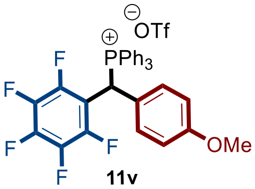

11v

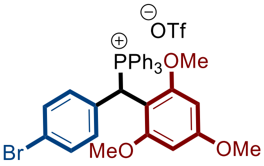

11w

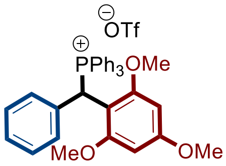

11x

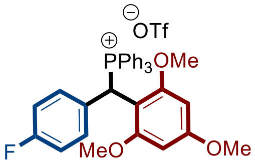

11y

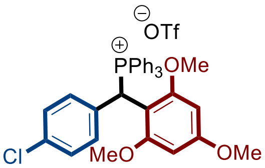

11z

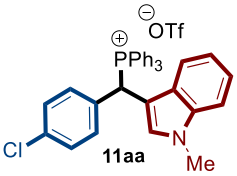

11aa

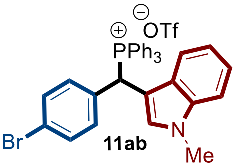

11ab

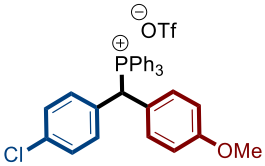

11ac

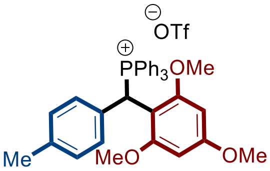

11ad

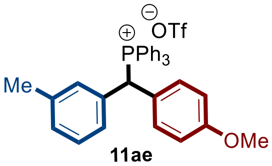

11ae

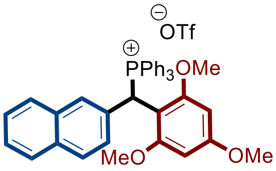

11af

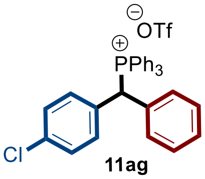

11ag

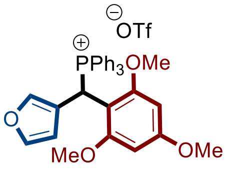

11ah

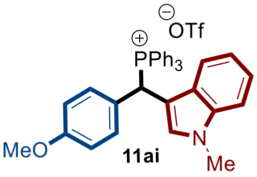

11ai

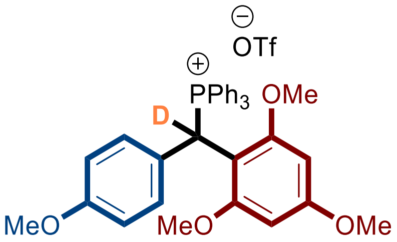

11a$_d$

((4-methoxyphenyl)(2,4,6-trimethoxyphenyl)methyl-d)triphenylphosphonium trifluoromethanesulfonate (11a$_d$):

Prepared according to general Procedure-A using aldehyde (**9a$_d$**) (0.95 mmol, 130 mg) and arene (**10a**) (0.95 mmol, 160 mg), product (**11a$_d$**) was isolated in (640 mg, 96% yield) as a white solid.

^{1}H NMR (400 MHz, $CDCl_3$) δ: 7.81 - 7.74 (m, 3H), 7.63 - 7.56 (m, 6H), 7.36 - 7.28 (m, 6H), 6.92 - 6.86 (m, 2H), 6.73 - 6.68 (m, 2H), 6.13 (d, J = 0.8 Hz, 2H), 3.84 (s, 3H), 3.76 (s, 3H), 3.48 (s, 6H).

^{13}C NMR (101 MHz, $CDCl_3$) δ: 162.8, 159.99, 159.96, 158.5, 158.4, 134.8, 134.8, 134.3, 134.2, 131.48, 131.43, 130.1, 130.0, 120.7, 119.8, 114.5, 114.4, 102.5, 102.4, 91.3, 55.9, 55.5.

^{31}P NMR (162 MHz, $CDCl_3$) δ: 23.0.

^{19}F NMR (376 MHz, $CDCl_3$) δ: −78.1.

HRMS (ESI) was calculated for $[C_{35}H_{33}DO_4P]^+$ $[M]^+$: m/z 550.2252, found = 550.2224.

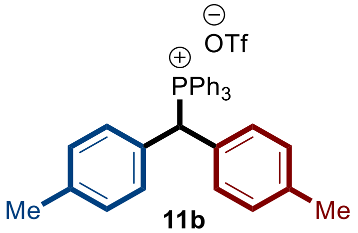

11b

(di-p-tolylmethyl)triphenylphosphonium trifluoromethanesulfonate (11b):

Prepared according to general Procedure-A using aldehyde (**9b**) (1.61 mmol, 200 mg) and arene (**10b**) (1.61 mmol, 153 mg), product (**11b**) was isolated in (680 mg, 67% yield) as a white solid.

^{1}H NMR (400 MHz, $CDCl_3$) δ: 7.78 - 7.72 (m, 3H), 7.63 - 7.50 (m, 12H), 7.13 (dd, J = 8.3, 1.9 Hz, 4H), 7.03 (dd, J = 7.9 Hz, 4H), 6.68 (d, J = 17.7 Hz, 1H), 2.30 - 2.25 (m, 6H).

^{13}**C NMR** (101 MHz, $CDCl_3$) δ: 139.0 (d, J = 2.7 Hz), 135.07, 135.03, 134.9, 130.8 (d, J = 6.9 Hz), 130.1 (d, J = 12.3 Hz), 130.0 (d, J = 1.7 Hz), 129.8 (d, J = 4.1 Hz), 118.5 (d, J = 82.1 Hz), 47.0 (d, J = 42.8 Hz), 21.2.

^{31}**P NMR** (162 MHz, $CDCl_3$) δ: 21.3.

^{19}**F NMR** (376 MHz, $CDCl_3$) δ: −78.1.

HRMS (ESI) was calculated for $[C_{33}H_{30}P]^+$ $[M]^+$: m/z 457.2079, found = 457.2061.

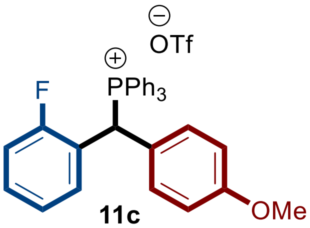

11c

(2-fluorophenyl)(4-methoxyphenyl)methyl)triphenylphosphonium trifluoromethanesulfonate (11c):

Prepared according to general Procedure-A using aldehyde (**9c**) (1.61 mmol, 200 mg) and arene (**10c**) (1.61 mmol, 175 mg), product (**11c**) was isolated in (625 mg, 62% yield) as a white solid.

^{1}**H NMR** (400 MHz, $CDCl_3$) δ: 7.85 - 7.76 (m, 3H), 7.69 - 7.60 (m, 6H), 7.55 - 7.45 (m, 6H), 7.41 - 7.27 (m, 2H), 7.14 (t, J = 7.7 Hz, 1H), 7.10 - 7.02 (m, 3H), 6.79 - 6.74 (m, 2H), 6.42 (d, J = 17.7 Hz, 1H), 3.75 (s, 3H).

^{13}**C NMR** (101 MHz, $CDCl_3$) δ: 160.4 (d, J = 2.6 Hz), 135.5 (d, J = 3.1 Hz), 134.7 (d, J = 9.2 Hz), 132.6 (d, J = 11.1 Hz), 131.9 (d, J = 5.8 Hz), 131.6 (d, J = 8.3 Hz), 130.4, 129.4 (d, J = 13.1 Hz), 125.6 (d, J = 3.7 Hz), 122.7 (d, J = 4.3 Hz), 121.2 (d, J = 2.3 Hz), 121.1 (d, J = 2.6 Hz), 117.8 (d, J = 82.6 Hz), 116.8 (d, J = 22.6 Hz), 114.9 (d, J = 2.1 Hz), 55.5, 42.6 (d, J = 45.5 Hz).

^{31}**P NMR** (162 MHz, $CDCl_3$) δ: 21.8 (d, J = 5.0 Hz).

^{19}**F NMR** (376 MHz, $CDCl_3$) δ: −78.3, −111.0 (d, J = 5.3 Hz).

HRMS (ESI]) was calculated for $[C_{32}H_{27}FOP]^+$ $[M]^+$: m/z 477.1778, found = 477.1765.

Note: The NMR's contain additional impurity peaks, compound was used for the next steps without further purifications.

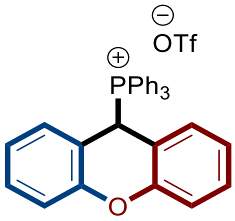

11d

Triphenyl(9H-xanthen-9-yl)phosphonium trifluoromethanesulfonate (11d):

Prepared according to general Procedure-A using aldehyde (**9d**) (0.73 mmol, 145 mg), product (**11d**) was isolated in (275 mg, 63% yield) as a white solid.

^{1}H NMR (400 MHz, $CDCl_3$) δ: 7.72 - 7.64 (m, 3H), 7.51 - 7.43 (m, 6H), 7.38 - 7.29 (m, 6H), 7.26 - 7.16 (m, 4H), 6.89 - 6.82 (m, 3H), 6.79 (dt, J = 8.2, 1.1 Hz, 2H).

^{13}C NMR (101 MHz, $CDCl_3$) δ: 154.5 (d, J = 5.4 Hz), 135.2 (d, J = 3.0 Hz), 135.0 (d, J = 8.8 Hz), 131.4 (d, J = 4.5 Hz), 130.6 (d, J = 3.9 Hz), 129.9 (d, J = 12.1 Hz), 124.4 (d, J = 3.1 Hz), 117.1 (d, J = 3.5 Hz), 116.2 (d, J = 80.5 Hz), 114.0 (d, J = 4.7 Hz), 41.1 (d, J = 41.9 Hz).

^{31}P NMR (162 MHz, $CDCl_3$) δ: 20.9.

^{19}F NMR (376 MHz, $CDCl_3$) δ: −78.1.

HRMS (ESI) was calculated for $[C_{31}H_{24}OP]^+$ [M]+: m/z 443.1559, found = 443.1548.

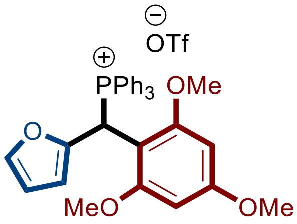

11e

(furan-2-yl(2,4,6-trimethoxyphenyl)methyl)triphenylphosphonium trifluoromethanesulfonate (11e):

Prepared according to general Procedure-A using aldehyde (**9e**) (1.04 mmol, 100 mg) and arene (**10a**) (1.04 mmol, 175 mg), product (**11e**) was isolated in (585 mg, 89% yield) as a white solid.

^{1}H NMR (400 MHz, $CDCl_3$) δ: 7.81 - 7.71 (m, 3H), 7.62 - 7.53 (m, 6H), 7.39 - 7.29 (m, 6H), 7.20 (ddd, J = 2.7, 1.8, 0.9 Hz, 1H), 6.66 (dd, J = 17.0, 1.3 Hz, 1H), 6.33 (ddd, J = 3.0, 1.9, 0.9 Hz, 1H), 6.11 (tt, J = 3.7, 1.1 Hz, 1H), 6.02 (s, 2H), 3.81 (s, 3H), 3.46 (s, 6H).

^{13}C NMR (101 MHz, $CDCl_3$) δ: 163.3 (d, J = 2.4 Hz), 158.5 (d, J = 5.4 Hz), 145.2 (d, J = 6.6 Hz), 142.9 (d, J = 3.3 Hz), 134.9 (d, J = 3.0 Hz), 133.9 (d, J = 9.2 Hz), 129.9 (d, J = 12.3 Hz), 119.6 (d,

J = 82.6 Hz), 111.9 (d, J = 7.0 Hz), 111.7 (d, J = 2.7 Hz), 98.9 (d, J = 5.1 Hz), 91.1 (d, J = 1.8 Hz), 55.9, 55.6, 36.7 (d, J = 47.6 Hz).

^{31}P NMR (162 MHz, $CDCl_3$) δ: 22.9.

^{19}F NMR (376 MHz, $CDCl_3$) δ: −78.1.

HRMS (ESI) was calculated for $[C_{32}H_{30}O_4P]^+$ $[M]^+$: m/z 509.1876, found = 509.1859.

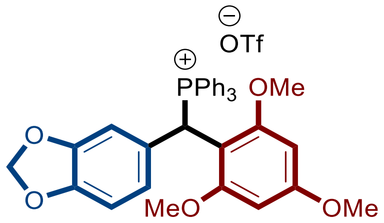

11f

(benzo[d][1,3]dioxol-5-yl(2,4,6-trimethoxyphenyl)methyl)triphenylphosphonium trifluoromethanesulfonate (11f):

Prepared according to general Procedure-A using aldehyde (**9f**) (0.73 mmol, 109 mg) and arene (**10a**) (0.73 mmol, 122 mg), product (**11f**) was isolated in (415 mg, 80% yield) as a white solid.

^{1}H NMR (400 MHz, $CDCl_3$) δ: 7.82 - 7.73 (m, 3H), 7.66 - 7.56 (m, 6H), 7.41 - 7.30 (m, 6H), 6.63 - 6.56 (m, 1H), 6.48 - 6.42 (m, 3H), 6.12 (s, 2H), 5.93 (d, J = 1.3 Hz, 2H), 3.84 (s, 3H), 3.47 (brs, 6H).

^{13}C NMR (101 MHz, $CDCl_3$): δ 162.9 (d, J = 1.2 Hz), 158.4 (d, J = 5.8 Hz), 148.2 (d, J = 2.5 Hz), 148.1 (d, J = 3.3 Hz), 134.8 (d, J = 2.9 Hz), 134.2 (d, J = 8.7 Hz), 130.1 (d, J = 12.1 Hz), 125.2 (d, J = 3.4 Hz), 124.1 (d, J = 5.8 Hz), 120.2 (d, J = 82.9 Hz), 110.3 (d, J = 4.4 Hz), 108.6 (d, J = 2.9 Hz), 102.3 (d, J = 3.5 Hz), 101.7, 91.3, 55.9, 41.5 (d, J = 47.4 Hz).

^{31}P NMR (162 MHz, $CDCl_3$): δ 23.4.

^{19}F NMR (376 MHz, $CDCl_3$): δ −78.1.

HRMS (ESI) was calculated for $[C_{35}H_{32}O_5P+H]^+$ $[M+H]^+$: m/z 564.2060, found = 564.1901.

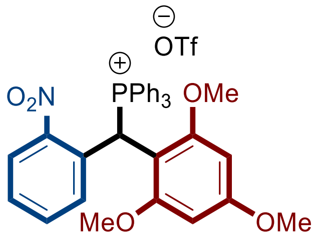

11g

(2-nitrophenyl)(2,4,6-trimethoxyphenyl)methyl)triphenylphosphonium trifluoromethanesulfonate (11g):

Prepared according to general Procedure-A using aldehyde (**9g**) (0.73 mmol, 110 mg) and arene (**10a**) (0.73 mmol, 122 mg), product (**11g**) was isolated in (432 mg, 83% yield) as a white solid.

^{1}H NMR (400 MHz, $CDCl_3$) δ: 7.74 - 7.69 (m, 5H), 7.66 - 7.60 (m, 2H), 7.58 - 7.53 (m, 6H), 7.49 - 7.42 (m, 7H), 6.07 (s, 2H), 3.82 (s, 3H), 3.49 (s, 6H).

^{13}C NMR (101 MHz, $CDCl_3$) δ: 163.6 (d, J = 2.0 Hz), 158.7 (d, J = 5.4 Hz), 148.2 (d, J = 6.5 Hz), 134.6 (d, J = 3.0 Hz), 134.1 (d, J = 9.0 Hz), 133.6 (d, J = 4.9 Hz), 130.0 (d, J = 2.2 Hz), 129.9, 129.8, 128.2, 120.2 (d, J = 83.5 Hz), 99.8 (d, J = 3.6 Hz), 91.6, 55.9, 55.6, 35.0 (d, J = 51.1 Hz).

^{31}P NMR (162 MHz, $CDCl_3$) δ: 26.3.

^{19}F NMR (376 MHz, $CDCl_3$) δ: −78.1.

HRMS (ESI) was calculated for $[C_{34}H_{31}NO_5P]^+$ $[M]^+$: m/z 564.1934 found = 564.1911.

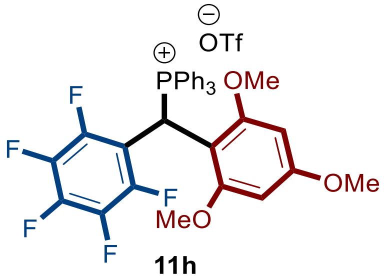

11h

((perfluorophenyl)(2,4,6-trimethoxyphenyl)methyl)triphenylphosphonium trifluoromethanesulfonate (11h):

Prepared according to general Procedure-A using aldehyde (**9h**) (0.73 mmol, 144 mg) and arene (**10a**) (0.73 mmol, 122 mg), product (**11h**) was isolated in (360 mg, 65% yield) as a white solid.

^{1}H NMR (400 MHz, $CDCl_3$) δ: 7.84 - 7.71 (m, 3H), 7.71 - 7.58 (m, 12H), 6.89 (d, J = 19.2 Hz, 1H), 5.97 (s, 2H), 3.81 (s, 3H), 3.30 (s, 6H).

^{13}C NMR (101 MHz, $CDCl_3$) δ: 163.6 (d, J = 2.1 Hz), 158.7 (d, J = 4.8 Hz), 135.0 (d, J = 3.3 Hz), 134.0 (d, J = 9.3 Hz), 130.0 (d, J = 12.4 Hz), 119.4 (d, J = 84.7 Hz), 91.0, 55.8, 55.3.

^{31}P NMR (162 MHz, $CDCl_3$) δ: 22.6.

^{19}F NMR (376 MHz, $CDCl_3$) δ: −78.2, −133.6 (d, J = 17.7 Hz), −151.7 (t, J = 21.1 Hz), −160.6 - −160.8 (m).

HRMS (ESI) was calculated for $[C_{34}H_{27}F_5O_3P]^+$ $[M]^+$: m/z 609.1612, found = 609.1604.

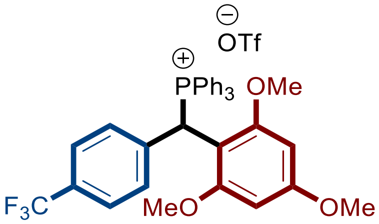

11i

triphenyl((4-(trifluoromethyl)phenyl)(2,4,6-trimethoxyphenyl)methyl)phosphonium trifluoromethanesulfonate (11i):

Prepared according to general Procedure-A using aldehyde (**9i**) (0.73 mmol, 127 mg) and arene (**10a**) (0.73 mmol, 122 mg), product (**11i**) was isolated in (284.7 mg, 53% yield) as a white solid.

^{1}H NMR (400 MHz, $CDCl_3$) δ 7.84 - 7.74 (m, 3H), 7.67 - 7.55 (m, 6H), 7.44 (d, J = 8.2 Hz, 2H), 7.39 - 7.30 (m, 5H), 7.16 (dd, J = 8.6, 2.1 Hz, 2H), 6.64 (d, J = 19.8 Hz, 1H), 6.10 (d, J = 0.8 Hz, 2H), 3.82 (s, 3H), 3.47 (s, 6H).

^{13}C NMR (101 MHz, $CDCl_3$) δ 163.2 (d, J = 1.8 Hz), 158.5 (d, J = 5.6 Hz), 136.7, 135.1 (d, J = 3.1 Hz), 134.1 (d, J = 9.0 Hz), 130.4 (d, J = 5.0 Hz), 130.2 (d, J = 12.2 Hz), 125.8 (q, J = 2.8 Hz), 122.8, 119.9, 119.1, 100.8 (d, J = 4.1 Hz), 91.3, 55.8, 55.6, 40.8 (d, J = 48.0 Hz).

^{31}P NMR (162 MHz, $CDCl_3$) δ 24.6.

^{19}F NMR (376 MHz, $CDCl_3$) δ −62.7, −78.2.

HRMS (ESI) was calculated for $[C_{35}H_{31}F_3O_3P]^+$ $[M]^+$: m/z 587.1957, found = 587.1942.

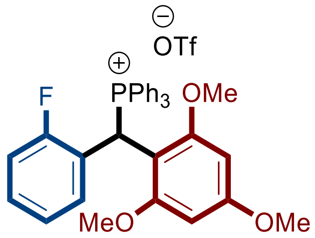

11j

((2-fluorophenyl)(2,4,6-trimethoxyphenyl)methyl)triphenylphosphonium trifluoromethanesulfonate (11j):

Prepared according to general Procedure-A using aldehyde (**9c**) (8.06 mmol, 1.0 g) and arene (**10a**) (8.06 mmol, 1.14 g), product (**11j**) was isolated in (5.5 g, 96% yield) as a white solid.

^{1}H NMR (400 MHz, $CDCl_3$) δ: 7.75 - 7.68 (m, 3H), 7.57 - 7.47 (m, 6H), 7.39 - 7.30 (m, 6H), 7.25 - 7.13 (m, 2H), 7.12 - 7.01 (m, 2H), 6.65 (dd, J = 11.2, 8.1 Hz, 1H), 6.08 (s, 2H), 3.82 (s, 3H), 3.53 (s, 6H).

^{13}C NMR (101 MHz, $CDCl_3$) δ: 163.31 (d, J = 2.1 Hz), 158.4 (d, J = 5.7 Hz), 134.6 (d, J = 3.0 Hz), 134.0 (d, J = 1.7 Hz), 133.9 (d, J = 1.7 Hz), 131.3 – 131.1 (m), 130.8 (d, J = 3.1 Hz), 130.7 (d, J = 2.9 Hz), 129.6 (d, J = 12.2 Hz), 123.7 (d, J = 233.2 Hz), 119.8 (d, J = 82.9 Hz), 115.1, 114.9, 99.4 (d, J = 4.0 Hz), 91.4 (d, J = 1.7 Hz), 55.8 (d, J = 15.0 Hz), 32.8 (d, J = 47.3 Hz).

^{31}P NMR (162 MHz, $CDCl_3$) δ: 28.1 (d, J = 4.7 Hz).

^{19}F NMR (376 MHz, $CDCl_3$) δ: −78.1, −106.7 (d, J = 4.3 Hz).

HRMS (ESI) was calculated for $[C_{34}H_{31}FO_3P]^+$ $[M]^+$: m/z 537.1989, found = 537.1975.

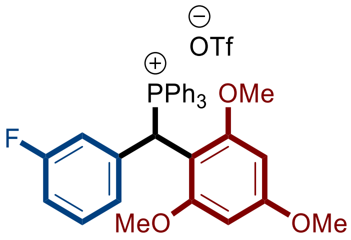

11k

((3-fluorophenyl)(2,4,6-trimethoxyphenyl)methyl)triphenylphosphonium trifluoromethanesulfonate (11k):

Prepared according to general Procedure-A using aldehyde (**9j**) (0.80 mmol, 100 mg) and arene (**10a**) (0.80 mmol, 135 mg), product (**11k**) was isolated in (520 mg, 94% yield) as a white solid.

^{1}H NMR (400 MHz, $CDCl_3$) δ: 7.82 - 7.73 (m, 3H), 7.60 (td, J = 7.9, 3.6 Hz, 6H), 7.34 (ddd, J = 12.1, 8.5, 1.3 Hz, 6H), 7.17 (td, J = 8.0, 5.9 Hz, 1H), 7.02 - 6.93 (m, 1H), 6.78 (dt, J = 7.9, 2.2 Hz, 1H), 6.69 (dq, J = 10.3, 2.3 Hz, 1H), 6.58 (d, J = 19.4 Hz, 1H), 6.11 (s, 2H), 3.83 (s, 3H), 3.48 (s, 6H).

^{13}C NMR (101 MHz, $CDCl_3$) δ: 163.9 (d, J = 2.6 Hz), 163.1, 161.4 (d, J = 2.7 Hz), 158.6 (d, J = 5.6 Hz), 135.0 (d, J = 2.9 Hz), 134.7 (d, J = 2.5 Hz), 134.6 (d, J = 2.5 Hz), 134.1 (d, J = 8.9 Hz), 130.6 (d, J = 2.3 Hz), 130.5 (d, J = 2.2 Hz), 130.2 (d, J = 12.2 Hz), 125.9 – 125.6 (m), 121.0 (d, J = 321.0 Hz), 119.7 (d, J = 83.2 Hz), 117.2 (d, J = 4.8 Hz), 116.9 (d, J = 4.8 Hz), 116.0 (d, J = 2.6 Hz), 115.8 (d, J = 2.7 Hz), 101.1 (d, J = 4.2 Hz), 91.3, 55.9, 55.6, 40.8 (d, J = 49.1 Hz).

^{31}P NMR (162 MHz, $CDCl_3$) δ: 24.4.

^{19}F NMR (376 MHz, $CDCl_3$) δ: −78.1, −110.7 (d, J = 2.3 Hz).

HRMS (ESI) was calculated for $[C_{34}H_{31}FO_3P]^+$ $[M]^+$: m/z 537.1989, found = 537.1965.

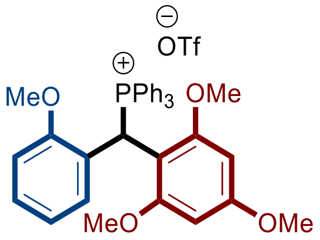

11l

((2-methoxyphenyl)(2,4,6-trimethoxyphenyl)methyl)triphenylphosphonium trifluoromethanesulfonate (11l):

Prepared according to general Procedure-A using aldehyde (**9k**) (0.73 mmol, 100 mg) and arene (1**0a**) (0.73 mmol, 123 mg), product (**11l**) was isolated in (472 mg, 92% yield) as a white solid.

^{1}H NMR (400 MHz, $CDCl_3$) δ: 7.81 - 7.72 (m, 3H), 7.64 - 7.55 (m, 6H), 7.39 - 7.28 (m, 6H), 7.10 (t, J = 8.1 Hz, 1H), 6.81 (d, J = 8.4 Hz, 1H), 6.61 - 6.47 (m, 3H), 6.12 (s, 2H), 3.84 (s, 3H), 3.54 (s, 3H), 3.49 (s, 6H).

^{13}C NMR (101 MHz, $CDCl_3$) δ: 162.9 (d, J = 1.5 Hz), 159.8 (d, J = 2.5 Hz), 158.6 (d, J = 5.8 Hz), 134.9 (d, J = 3.0 Hz), 134.2 (d, J = 8.8 Hz), 133.3 (d, J = 3.1 Hz), 130.1 (d, J = 12.2 Hz), 122.3 (d, J = 5.0 Hz), 121.0 (d, J = 320.8 Hz), 120.1 (d, J = 83.1 Hz), 115.8 (d, J = 4.9 Hz), 114.4 (d, J = 3.1 Hz), 101.8 (d, J = 3.9 Hz), 91.3, 55.9, 55.2, 41.4 (d, J = 47.4 Hz).

^{31}P NMR (162 MHz, $CDCl_3$) δ: 23.9.

^{19}F NMR (376 MHz, $CDCl_3$) δ: −78.1.

HRMS (ESI) was calculated for $[C_{35}H_{34}O_4P]^+$ $[M]^+$: m/z 549.2189, found = 549.2172.

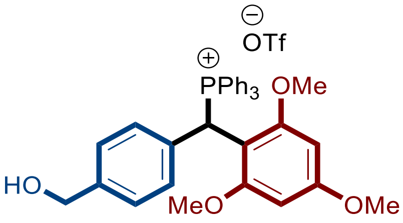

11m

((4-(hydroxymethyl)phenyl)(2,4,6-trimethoxyphenyl)methyl)triphenylphosphonium trifluoromethanesulfonate (11m):

Prepared according to general Procedure-A using aldehyde (**9l**) (0.89 mmol, 159 mg) and arene (**10a**) (0.89 mmol, 150 mg), product (**11m**) was isolated in (378 mg, 60% yield) as a white solid.

^{1}H NMR (400 MHz, CD_3CN) δ: 7.82 - 7.75 (m, 3H), 7.62 - 7.55 (m, 6H), 7.48 - 7.39 (m, 6H), 7.19 - 7.14 (m, 2H), 7.06 (dd, J = 8.3, 2.2 Hz, 2H), 6.64 (d, J = 19.3 Hz, 1H), 6.18 (d, J = 0.9 Hz, 2H), 4.52 (d, J = 2.0 Hz, 2H), 3.80 (s, 3H), 3.48 (s, 6H).

^{13}C NMR (101 MHz, CD_3CN) δ: 163.6 (d, J = 1.6 Hz), 159.4 (d, J = 5.8 Hz), 143.9 (d, J = 3.3 Hz), 135.4 (d, J = 3.0 Hz), 135.3 (d, J = 8.9 Hz), 131.9 (d, J = 3.0 Hz), 130.9 (d, J = 5.0 Hz), 130.6 (d, J = 12.1 Hz), 127.8 (d, J = 2.5 Hz), 121.1 (d, J = 83.2 Hz), 103.3 (d, J = 3.7 Hz), 92.2, 63.9, 56.3, 41.5 (d, J = 47.7 Hz).

^{31}P NMR (162 MHz, CD_3CN) δ: 24.4.

^{19}F NMR (376 MHz, CD_3CN) δ: −79.3.

HRMS (ESI) was calculated for $[C_{35}H_{34}O_4P]^+$ $[M]^+$: m/z 549.2189, found = 549.2172.

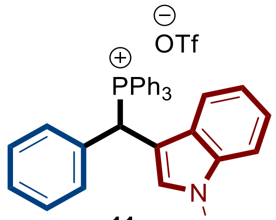

11n

((1-methyl-1H-indol-3-yl)(phenyl)methyl)triphenylphosphonium trifluoromethanesulfonate (11n):

Prepared according to general Procedure-A using aldehyde (**9p**) (0.73 mmol, 82 mg) and arene (**10d**) (0.73 mmol, 95 mg), product (**11n**) was isolated in (400 mg, 86% yield) as a pink solid.

^{1}H NMR (400 MHz, $CDCl_3$) δ: 7.78 - 7.74 (m, 3H), 7.61 - 7.54 (m, 12H), 7.41 - 7.36 (m, 1H), 7.31 - 7.27 (m, 3H), 7.24 - 7.16 (m, 4H), 7.06 - 7.00 (m, 1H), 6.76 - 6.68 (m, 2H), 3.68 (s, 3H).

^{13}C NMR (101 MHz, $CDCl_3$) δ: 136.7, 135.2 (d, J = 3.0 Hz), 134.9 (d, J = 9.1 Hz), 132.6, 130.8 (d, J = 5.8 Hz), 130.2 (d, J = 12.2 Hz), 129.0 (d, J = 2.4 Hz), 128.9 (d, J = 3.2 Hz), 127.1 (d, J = 7.5 Hz), 123.0, 122.3, 120.6, 119.6, 118.8, 118.1, 109.9, 105.4, 41.5 (d, J = 43.2 Hz), 33.3.

^{31}P NMR (162 MHz, $CDCl_3$) δ: 20.9.

^{19}F NMR (376 MHz, $CDCl_3$) δ: −78.1.

HRMS (ESI) was calculated for $[C_{34}H_{29}NP]^+$ $[M]^+$: m/z 482.2032, found = 482.2024.

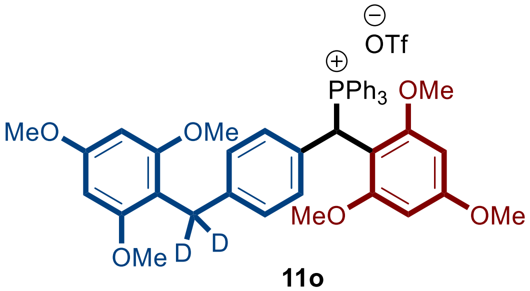

11o

triphenyl((2,4,6-trimethoxyphenyl)(4-((2,4,6-trimethoxyphenyl)methyl-d2)phenyl)methyl)phosphonium trifluoromethanesulfonate (11o):

Prepared according to general Procedure-A using aldehyde (**9n**) (0.17 mmol, 51 mg) and arene (**10a**) (0.17 mmol, 30 mg), product (**11o**) was isolated in (114 mg, 76% yield) as a white solid. 77% Deuterium-incorporation.

^{1}H NMR (400 MHz, $CDCl_3$) δ: 7.78 - 7.72 (m, 3H), 7.58 - 7.53 (m, 6H), 7.32 - 7.24 (m, 6H), 7.07 - 7.02 (m, 2H), 6.82 - 6.75 (m, 2H), 6.50 (d, J = 18.6 Hz, 1H), 6.11 (s, 2H), 6.10 (d, J = 0.8 Hz, 2H), 3.82 (s, 3H), 3.81 - 3.79 (m, 0.47H, 77% labeled), 3.79 (s, 3H), 3.76 (s, 6H), 3.46 (brs, 6H).
^{13}C NMR (101 MHz, $CDCl_3$) δ: 162.7 (d, J = 1.8 Hz), 159.9, 158.8, 158.5 (d, J = 5.8 Hz), 143.5 (t, J = 3.7 Hz), 134.7 (d, J = 3.0 Hz), 134.2 (d, J = 8.7 Hz), 129.9 (d, J = 12.0 Hz), 129.6 (d, J = 4.9 Hz), 129.2 (d, J = 2.7 Hz), 128.3 (d, J = 3.5 Hz), 122.6, 120.4 (d, J = 82.7 Hz), 119.4, 109.6, 102.2 (d, J = 3.8 Hz), 91.2, 90.7, 55.9, 55.8, 55.5, 41.1 (d, J = 46.6 Hz).
^{31}P NMR (162 MHz, $CDCl_3$) δ: 23.5.
^{19}F NMR (376 MHz, $CDCl_3$) δ: −78.1.
HRMS (ESI) was calculated for $[C_{44}H_{42}D_2O_6P]^+$ $[M]^+$: m/z 701.2995, found = 701.2964.

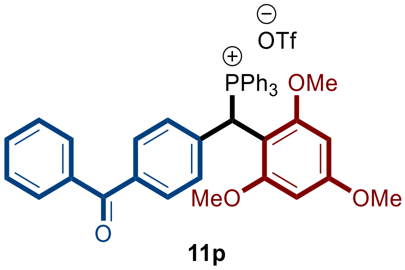

11p

(4-benzoylphenyl)(2,4,6-trimethoxyphenyl)methyl)triphenylphosphonium trifluoromethanesulfonate (11p):

Prepared according to general Procedure-A using aldehyde (**9o**) (0.73 mmol, 22 mg) and arene (**10a**) (0.73 mmol, 17 mg), product (**11p**) was isolated in (420 mg, 72% yield) as a white solid.
^{1}H NMR (400 MHz, $CDCl_3$) δ: 7.81 - 7.76 (m, 3H), 7.74 - 7.70 (m, 2H), 7.65 - 7.57 (m, 9H), 7.49 - 7.44 (m, 2H), 7.42 - 7.34 (m, 6H), 7.16(dd, J = 2.1, 0.6 Hz, 2H), 6.69 (d, J = 19.8 Hz, 1H), 6.13 (d, J = 0.8 Hz, 2H), 3.85 (s, 3H), 3.51 (brs, 6H).
^{13}C NMR (101 MHz, $CDCl_3$) δ: 195.9, 163.2 (d, J = 1.8 Hz), 158.7 (d, J = 5.6 Hz), 137.7 (d, J = 2.9 Hz), 136.9 (d, J = 2.5 Hz), 135.1 (d, J = 3.1 Hz), 134.3 (d, J = 9.0 Hz), 133.0, 132.6 (d, J = 11.1 Hz), 130.5 (d, J = 2.4 Hz), 130.3, 130.2, 130.1, 130.0 (d, J = 4.9 Hz), 129.4 (d, J = 13.1 Hz), 128.6, 101.0 (d, J = 4.1 Hz), 91.4, 55.9, 55.8 – 55.4 (m), 41.1 (d, J = 47.6 Hz).

^{31}**P NMR** (162 MHz, $CDCl_3$) δ: 24.7.

^{19}**F NMR** (376 MHz, $CDCl_3$) δ: −78.1.

HRMS (ESI) was calculated for $[C_{41}H_{36}O_4P]^+$ $[M]^+$: m/z 623.2345, found = 623.2316.

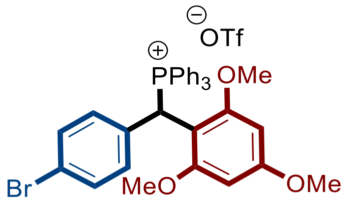

11w

((4-bromophenyl)(2,4,6-trimethoxyphenyl)methyl)triphenylphosphonium trifluoromethanesulfonate (11w):

Prepared according to general Procedure-A using aldehyde (**9l**) (0.73 mmol, 143 mg) and arene (**10a**) (0.73 mmol, 122 mg), product (**11w**) was isolated in (450 mg, 82% yield) as a white solid.

^{1}**H NMR** (400 MHz, $CDCl_3$) δ: 7.80 - 7.72 (m, 3H), 7.64 - 7.56 (m, 6H), 7.38 - 7.27 (m, 8H), 6.91 - 6.85 (m, 2H), 6.50 (d, *J* = 19.3 Hz, 1H), 6.10 (s, 2H), 3.82 (s, 3H), 3.59 - 3.34 (m, 6H).

^{13}**C NMR** (101 MHz, $CDCl_3$) δ: 158.5 (d, *J* = 5.6 Hz), 134.9 (d, *J* = 3.1 Hz), 134.1 (d, *J* = 8.9 Hz), 132.1 (d, *J* = 2.6 Hz), 131.7 (d, *J* = 4.9 Hz), 131.3 (d, *J* = 2.8 Hz), 130.1 (d, *J* = 12.2 Hz), 123.0 (d, *J* = 3.8 Hz), 40.8 (d, *J* = 47.9 Hz).

^{31}**P NMR** (162 MHz, $CDCl_3$) δ: 23.8.

^{19}**F NMR** (376 MHz, $CDCl_3$) δ: −78.0.

HRMS (ESI) was calculated for $[C_{34}H_{31}BrO_3P]^+$ $[M]^+$: m/z 597.1188, found = 597.1165.

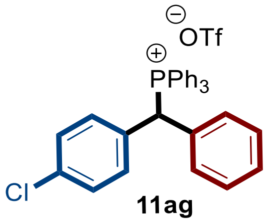

11ag

((4-chlorophenyl)(phenyl)methyl)triphenylphosphonium trifluoromethanesulfonate (11ag):

Prepared according to general Procedure-A using aldehyde (**9q**) (0.73 mmol, 102 mg) and arene (**10e**) (0.73 mmol, 57 mg), product (**11ag**) was isolated in (380 mg, 62% yield) as a white solid.

^{1}H NMR (400 MHz, $CDCl_3$) δ 7.80 - 7.73 (m, 3H), 7.66 - 7.55 (m, 12H), 7.31 - 7.19 (m, 9H), 7.01 (d, J = 18.0 Hz, 1H).

^{13}C NMR (101 MHz, $CDCl_3$) δ 135.2 (d, J = 3.0 Hz), 134.9 (d, J = 9.1 Hz), 132.7 (d, J = 4.1 Hz), 132.3 (d, J = 6.6 Hz), 131.5 (d, J = 4.2 Hz), 130.9 (d, J = 6.9 Hz), 130.3 (d, J = 12.3 Hz), 129.4 (t, J = 2.1 Hz), 129.2 (d, J = 2.5 Hz), 118.1 (d, J = 82.4 Hz), 46.3 (d, J = 43.5 Hz).

^{31}P NMR (162 MHz, $CDCl_3$) δ: 21.9.

^{19}F NMR (376 MHz, $CDCl_3$) δ: -78.1.

HRMS (ESI) was calculated for $[C_{31}H_{25}ClP]^+$ $[M]^+$: m/z 463.1376, found = 463.1359.

2.3. General Procedure-B and Characterization for Diarylmethanes products (12).

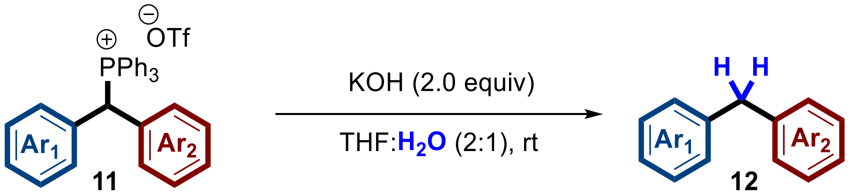

A 7 mL screw-cap vial containing a Teflon-coated magnetic stirring bar was charged with benzhydryl phosphonium salt **11** (0.2 mmol, 1.0 equivalent), 2 mL of THF, and 1 mL of water. The mixture was stirred at room temperature for five minutes. Powder KOH (0.4 mmol, 2.0 equivalents) was then added in a single portion. The progress of the reaction was monitored using thin-layer chromatography (TLC). The reaction mixture was quenched with H_2O, and the aqueous phase was extracted with EtOAc (2 x 5 mL). The combined organic phases were dried over $NaSO_4$, filtered and concentrated under reduced pressure (by evaporator). The crude material was purified by column chromatography, yielding the desired product (**12**).

Table - S2: Optimization table for Diarylmethanes (**12**) preparation.

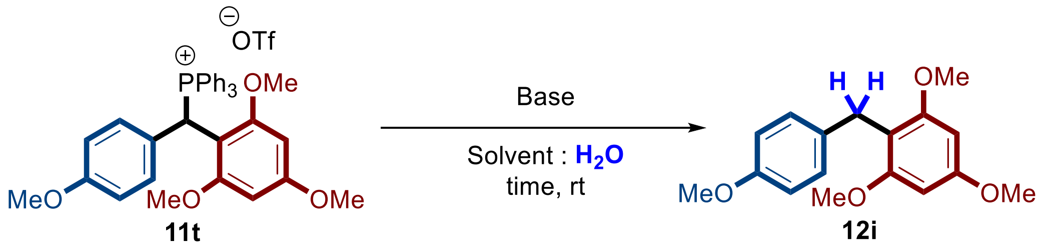

Entry	Solvent	H_2O	Base (Equiv.)	Time (hours)	2a (%)[b]
1.	CH_2Cl_2	20 eq.	DBU (1.1)	72	NR
2.	THF	20 eq.	DBU (1.1)	48	36
3.	THF	20 eq.	DABCO (1.0)	24	NR
4.	THF	20 eq.	DABCO (1.5)	24	NR
5.	THF	20 eq.	DMAP (1.1)	24	NR
6.	THF	20 eq.	Et_3N (1.1)	24	NR
7.	THF	20 eq.	Et_3N (1.5)	24	NR
8.	THF	20 eq.	Cs_2CO_3 (1.5)	24	NR
9.	THF	20 eq.	NaOH (2.0)	24	40
10.	THF	20 eq.	KOH (1.1)	24	32
11.	THF	1 mL	KOH (1.5)	48	84
**12.**	**THF**	**1 mL**	**KOH (2.0)**	**3**	**87**
13.	THF	1 mL	KOH (3.0)	3	60
14.	THF	1 mL	KOH (5.0)	3	68
15.	MeCN	1 mL	KOH (2.0)	72	47
16.	CH_2Cl_2	1 mL	KOH (2.0)	60	16
17.	THF	-	*Aq.* 1M KOH (2.0)	60	54[c]
18.	THF	-	*Aq.* 1M KOH (3.0)	15	46[c]
19.	THF	-	*Aq.* 1M KOH (5.0)	7	39[c]

Reaction conditions: [a] All reactions were carried out using 0.14 mmol of benzhydryl phosphonium salt **11t** at rt. [b] All reported yields are isolated yields. [c] The reaction was carried out in a stock solution of KOH in water. NR = No reaction was observed at all.

Table - S3: Scope of the diarylmethanes preparation (**12**).

Entry	Phosphonium Salts (11)	Diarylmethanes (12)	Time	Yield[a]
1.	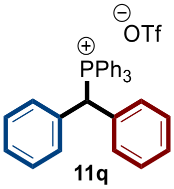 **11q**	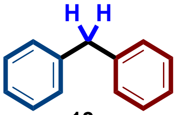 **12a**	1h	82%
2.	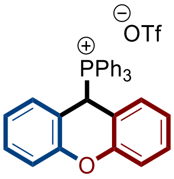 **11d**	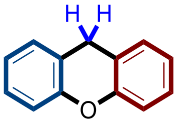 **12b**	1h	96%
3.	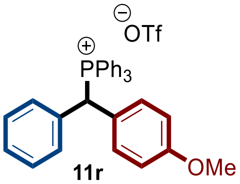 **11r**	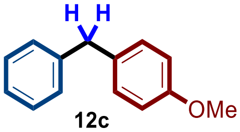 **12c**	1h	71%
4.	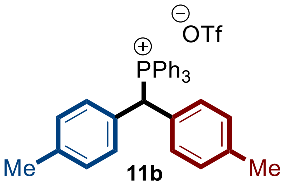 **11b**	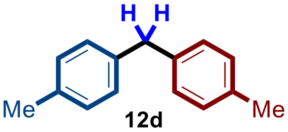 **12d**	1h	96%
5.	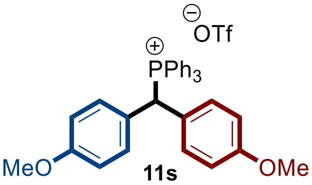 **11s**	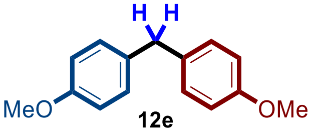 **12e**	1h	87%
6.	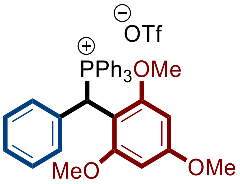 **11x**	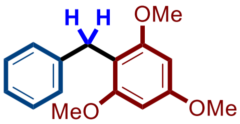 **12f**	1h	96%

7.	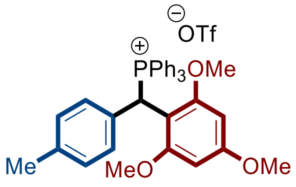 **11ad**	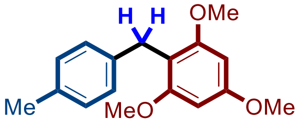 **12g**	5h	95%
8.	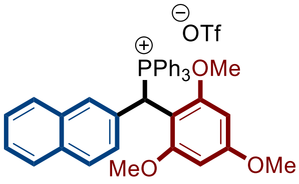 **11af**	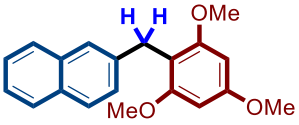 **12h**	5h	86%
9.	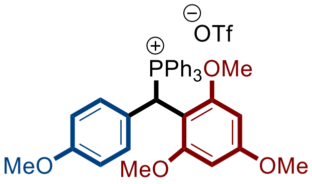 **11t**	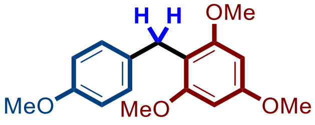 **12i**	3h	87%
10.	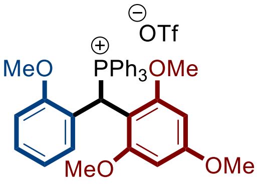 **11l**	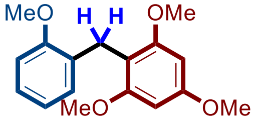 **12j**	5h	73%
11.	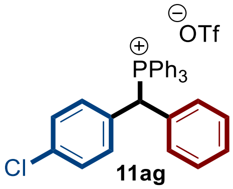 **11ag**	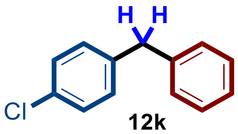 **12k**	1h	96%
12.	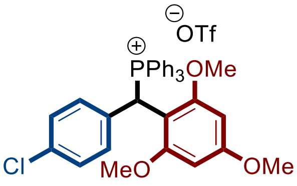 **11z**	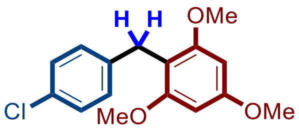 **12l**	5h	96%
13.	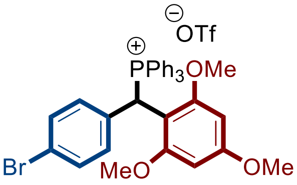 **11w**	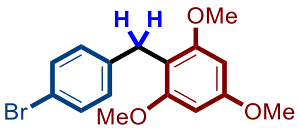 **12m**	5h	80%

14.	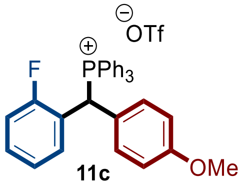 **11c**	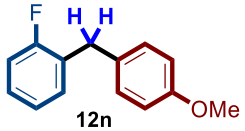 **12n**	5h	73%
15.	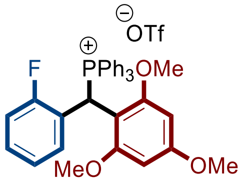 **11j**	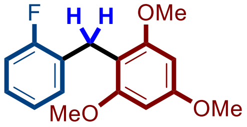 **12o**	5h	87%
16.	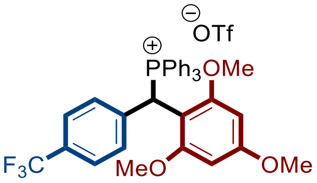 **11i**	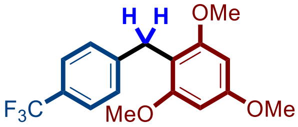 **12p**	5h	82%
17.	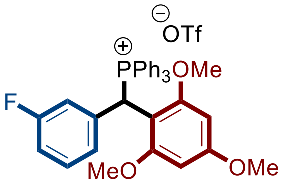 **11k**	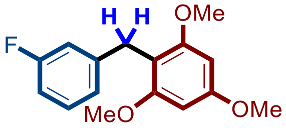 **12q**	5h	72%
18.	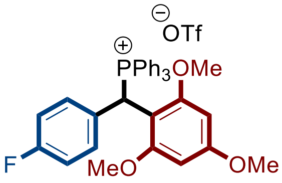 **11y**	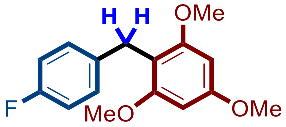 **12r**	5h	90%
19.	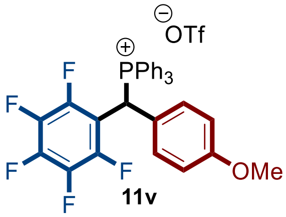 **11v**	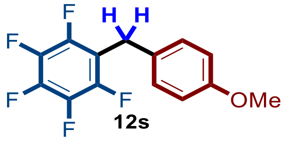 **12s**	5h	83%
20.	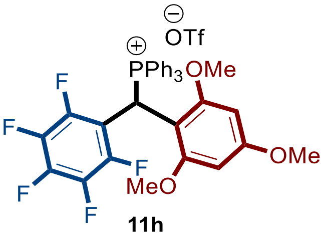 **11h**	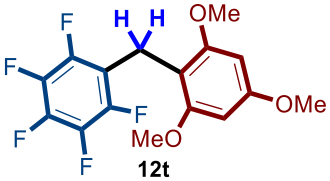 **12t**	5h	60%

21.	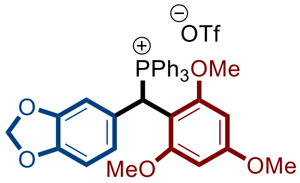 **11f**	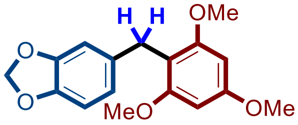 **12u**	5h	90%
22.	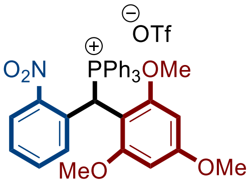 **11g**	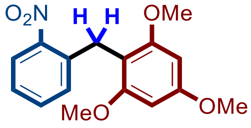 **12v**	5h	80%
23.	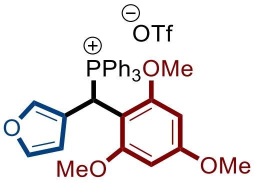 **11ah**	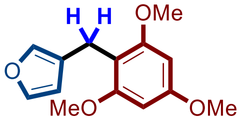 **12w**	5h	80%
24.	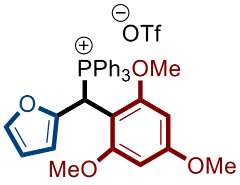 **11e**	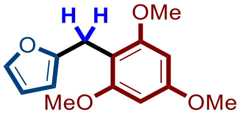 **12x**	5h	67%
25.	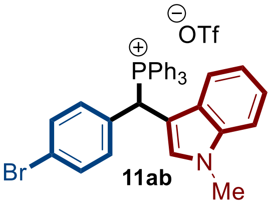 **11ab**	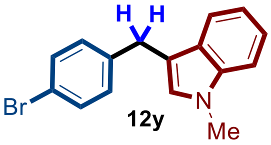 **12y**	5h	72%
26.	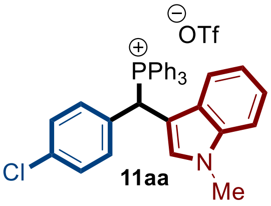 **11aa**	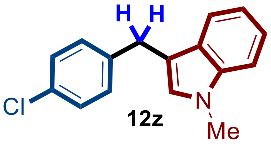 **12z**	5h	70%
27.	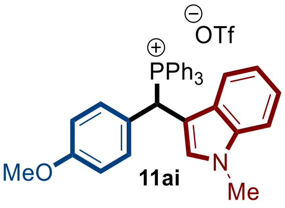 **11ai**	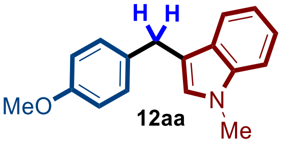 **12aa**	5h	40%

28.	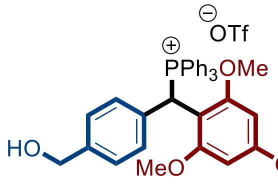 **11m**	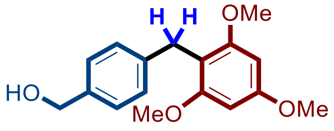 **12ab**	5h	68%
29.	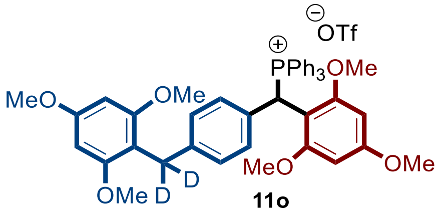 **11o**	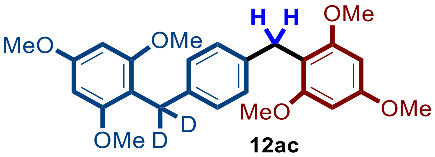 **12ac**	5h	71%
30.	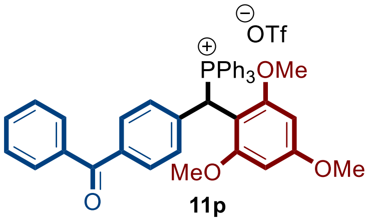 **11p**	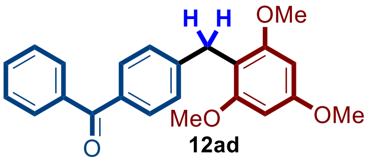 **12ad**	5h	84%

[a] Isolated yield.

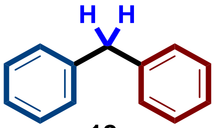

12a

Diphenylmethane (12a):

Prepared according to general Procedure-B using phosphonium salt (**11q**) (0.2 mmol, 115 mg) product (**12a**) was isolated in (28 mg, 82% yield) as a colorless oil.

R_f = 0.45 (5% EtOAc in hexane).

^{1}H NMR (400 MHz, $CDCl_3$) δ: 7.32 - 7.27 (m, 4H), 7.23 - 7.18 (m, 6H), 4.00 (s, 2H).

The spectral data are consistent with those reported in the literature. [9]

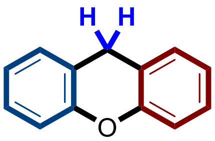

12b

9H-xanthene (12b):

Prepared according to general Procedure-B using phosphonium salt (**11d**) (0.16 mmol, 100 mg) product (**12b**) was isolated in (28 mg, 96% yield) as a colorless oil.

R_f = 0.43 (5% EtOAc in hexane).

^{1}H NMR (400 MHz, $CDCl_3$) δ: 7.23 - 7.16 (m, 4H), 7.07 - 7.01 (m, 4H), 4.06 (s, 2H).

The spectral data are consistent with those reported in the literature.[10]

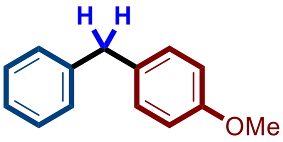

12c

1-benzyl-4-methoxybenzene (12c):

Prepared according to general Procedure-B using phosphonium salt (**11r**) (0.2 mmol, 123 mg) product (**12c**) was isolated in (28 mg, 71% yield) as a colorless oil.

R_f = 0.40 (5% EtOAc in hexane).

^{1}H NMR (400 MHz, $CDCl_3$) δ: 7.32 - 7.27 (m, 2H), 7.25 - 7.15 (m, 3H), 7.15 - 7.08 (m, 2H), 6.87 - 6.82 (m, 2H), 3.94 (s, 2H), 3.79 (s, 3H).

The spectral data are consistent with those reported in the literature.[9]

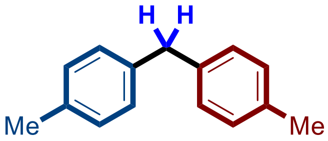

12d

Di-p-tolylmethane (12d):

Prepared according to general Procedure-B using phosphonium salt (**11b**) (0.2 mmol, 121 mg) product (**12d**) was isolated in (38 mg, 96% yield) as a colorless oil.

R_f = 0.41 (5% EtOAc in hexane).

¹H NMR (400 MHz, $CDCl_3$) δ: δ 7.13 - 7.08 (m, 8H), 3.93 (s, 2H), 2.34 (s, 6H).

The spectral data are consistent with those reported in the literature.[11]

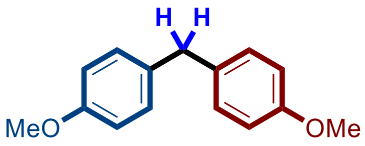

12e

bis(4-methoxyphenyl)methane (12e):

Prepared according to general Procedure-B using phosphonium salt (**11s**) (0.17 mmol, 110 mg) product (**12e**) was isolated in (34 mg, 87% yield) as a colorless oil.

R_f = 0.39 (5% EtOAc in hexane).

¹H NMR (400 MHz, $CDCl_3$) δ: 7.11 - 7.07 (m, 4H), 6.85 - 6.81 (m, 4H), 3.87 (s, 2H), 3.78 (s, 6H).

The spectral data are consistent with those reported in the literature.[9]

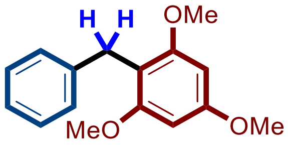

12f

2-benzyl-1,3,5-trimethoxybenzene (12f):

Prepared according to general Procedure-B using phosphonium salt (**11x**) (0.16 mmol, 105 mg) product (**12f**) was isolated in (39 mg, 96% yield) as a colorless oil.

R_f = 0.40 (5% EtOAc in hexane).

¹H NMR (400 MHz, $CDCl_3$) δ: 7.26 - 7.20 (m, 4H), 7.15 - 7.10 (m, 1H), 6.17 (s, 2H), 3.95 (s, 2H), 3.82 (s, 3H), 3.80 (s, 6H).

The spectral data are consistent with those reported in the literature.[12]

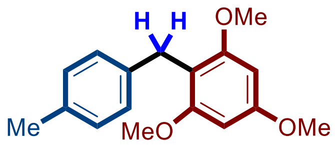

12g

1,3,5-trimethoxy-2-(4-methylbenzyl)benzene (12g):

Prepared according to general Procedure-B using phosphonium salt (**11ad**) (0.2 mmol, 136 mg) product (**12g**) was isolated in (52 mg, 95% yield) as a colorless solid.

R_f = 0.40 (5% EtOAc in hexane).

^{1}H NMR (400 MHz, $CDCl_3$) δ: 7.12 (d, J = 7.6 Hz, 2H), 7.02 (d, J = 7.6 Hz, 2H), 6.15 (s, 2H), 3.90 (s, 2H), 3.81 (s, 3H), 3.79 (s, 6H), 2.28 (s, 3H).

The spectral data are consistent with those reported in the literature.[13]

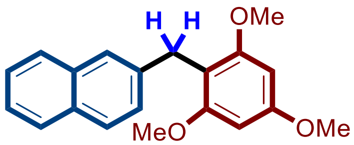

12h

2-(2,4,6-trimethoxybenzyl)naphthalene (12h):

Prepared according to general Procedure-B using phosphonium salt (**11af**) (0.2 mmol, 143 mg) product (**12h**) was isolated in (53 mg, 86% yield) as a colorless solid.

R_f = 0.48 (5% EtOAc in hexane).

^{1}H NMR (400 MHz, $CDCl_3$) δ: 8.36 (dd, J = 8.5, 1.1 Hz, 1H), 7.88 (dd, J = 8.2, 1.4 Hz, 1H), 7.69 (d, J = 8.2 Hz, 1H), 7.57 (ddd, J = 8.4, 6.7, 1.4 Hz, 1H), 7.51 (ddd, J = 8.1, 6.8, 1.2 Hz, 1H), 7.32 (dd, J = 8.2, 7.1 Hz, 1H), 6.97 (dq, J = 7.3, 1.2 Hz, 1H), 6.26 (s, 2H), 4.43 (s, 2H), 3.88 (s, 3H), 3.75 (s, 6H).

The spectral data are consistent with those reported in the literature.[14]

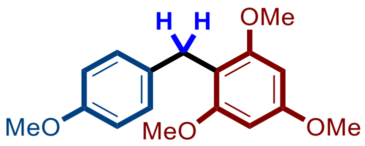

12i

1,3,5-trimethoxy-2-(4-methoxybenzyl)benzene (12i):

Prepared according to general Procedure-B using phosphonium salt (**11t**) (0.14 mmol, 100 mg) product (**12i**) was isolated in (34 mg, 87% yield) as a colorless oil. A 1.01-gram scale of **11t** reaction gave product **12i** (355 mg, 85% yield).

R_f = 0.54 (5% EtOAc in hexane).

^{1}H NMR (400 MHz, $CDCl_3$) δ: 7.16 (d, *J* = 8.7 Hz, 2H), 6.76 (d, *J* = 8.7 Hz, 2H), 6.15 (s, 2H), 3.87 (s, 2H), 3.81 (s, 3H), 3.79 (s, 6H), 3.75 (s, 3H).

The spectral data are consistent with those reported in the literature. [15]

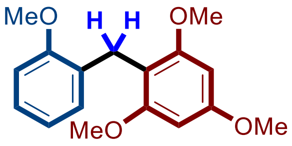

12j

1,3,5-trimethoxy-2-(2-methoxybenzyl)benzene (12j)

Prepared according to general Procedure-B using phosphonium salt (**11l**) (0.2 mmol, 150 mg) product (**12j**) was isolated in (41 mg, 73% yield) as a colorless oil.

R_f = 0.42 (5% EtOAc in hexane).

^{1}H NMR (400 MHz, $CDCl_3$) δ: 7.14 (t, *J* = 7.9 Hz, 1H), 6.86 - 6.80 (m, 2H), 6.70 - 6.65 (m, 1H), 6.16 (s, 2H), 3.92 (s, 2H), 3.81 (s, 3H), 3.79 (s, 6H), 3.76 (s, 3H).

^{13}C NMR (101 MHz, $CDCl_3$) δ: 159.8, 159.4, 158.9, 144.0, 128.9, 121.1, 114.5, 110.5, 110.1, 90.7, 55.8, 55.4, 55.1, 28.4.

HRMS (ESI) was calculated for $[C_{17}H_{20}O_4 + H]^+$ $[M + H]^+$: m/z 289.1434, found = 289.1423.

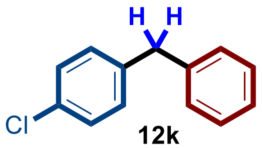

12k

1-benzyl-4-chlorobenzene (12k):

Prepared according to general Procedure-B using phosphonium salt (**11ag**) (0.17 mmol, 104 mg) product (**12k**) was isolated in (33 mg, 96% yield) as a colorless oil.

R_f = 0.43 (5% EtOAc in hexane).

^{1}H NMR (400 MHz, $CDCl_3$) δ: 7.33 - 7.26 (m, 3H), 7.26 - 7.19 (m, 2H), 7.19 - 7.15 (m, 2H), 7.14 - 7.10 (m, 2H), 3.96 (s, 2H).

The spectral data are consistent with those reported in the literature. [16]

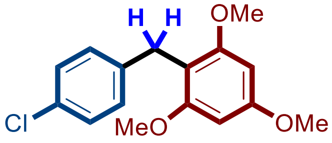

12l

2-(4-chlorobenzyl)-1,3,5-trimethoxybenzene (12l):

Prepared according to general Procedure-B using phosphonium salt (**11z**) (0.2 mmol, 140 mg) product (**12l**) was isolated in (56 mg, 96% yield) as a colorless solid.

R_f = 0.40 (5% EtOAc in hexane).

^{1}H NMR (400 MHz, $CDCl_3$) δ: 7.17 (s, 4H), 6.16 (s, 2H), 3.89 (s, 2H), 3.82 (s, 3H), 3.79 (s, 6H).

^{13}C NMR (101 MHz, $CDCl_3$) δ: 159.9, 158.9, 140.9, 130.9, 129.9, 128.1, 109.8, 90.7, 55.8, 55.4, 27.8.

HRMS (ESI) was calculated for $[C_{16}H_{17}ClO_3+H]^+$ $[M+H]^+$: m/z 293.0939, found = 293.0931.

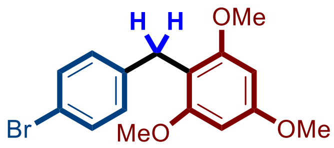

12m

2-(4-bromobenzyl)-1,3,5-trimethoxybenzene (12m):

Prepared according to general Procedure-B using phosphonium salt (**11w**) (0.2 mmol, 149 mg) product (**12m**) was isolated in (54 mg, 80% yield) as a colorless solid.

R_f = 0.4 (5% EtOAc in hexane).

^{1}H NMR (400 MHz, $CDCl_3$) δ: 7.35 - 7.30 (m, 2H), 7.14 - 7.09 (m, 2H), 6.16 (s, 2H), 3.88 (s, 2H), 3.82 (s, 3H), 3.79 (s, 6H).

^{13}C NMR (101 MHz, $CDCl_3$) δ: 159.9, 158.8, 141.4, 131.0, 130.3, 119.0, 109.7, 90.7, 55.8, 55.4, 27.9.

HRMS (ESI) was calculated for $[C_{16}H_{17}BrO_3+H]^+$ $[M+H]^+$: m/z 337.0433, found = 337.0425.

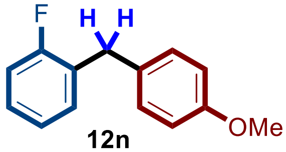

12n

1-fluoro-2-(4-methoxybenzyl)benzene (12n):

Prepared according to general Procedure-B using phosphonium salt (**11c**) (0.2 mmol, 143 mg) product (**12n**) was isolated in (36 mg, 73% yield) as a colorless oil.

R_f = 0.38 (5% EtOAc in hexane).

^{1}H NMR (400 MHz, $CDCl_3$) δ: 7.22 - 7.12 (m, 4H), 7.08 - 7.01 (m, 2H), 6.87 - 6.82 (m, 2H), 3.96 (s, 2H), 3.79 (s, 3H).

The spectral data are consistent with those reported in the literature. [17]

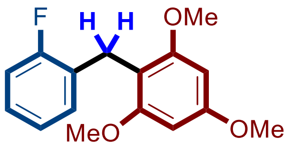

12o

2-(2-fluorobenzyl)-1,3,5-trimethoxybenzene (12o):

Prepared according to general Procedure-B using phosphonium salt (**11j**) (0.14 mmol, 98 mg) product (**12o**) was isolated in (27 mg, 87% yield) as a colorless oil.

R_f = 0.4 (5% EtOAc in hexane).

^{1}H NMR (400 MHz, $CDCl_3$) δ: 7.13 - 7.06 (m, 1H), 7.01 - 6.95 (m, 1H), 6.94 - 6.88 (m, 2H), 6.18 (s, 2H), 3.97 (s, 2H), 3.83 (s, 3H), 3.76 (s, 3H).

^{13}C NMR (101 MHz, $CDCl_3$) δ: 161.2 (d, J = 244.8 Hz), 160.1, 159.3, 129.8 (d, J = 4.8 Hz), 128.8 (d, J = 15.7 Hz), 126.8 (d, J = 7.9 Hz), 123.6 (d, J = 3.5 Hz), 114.7 (d, J = 22.3 Hz), 108.3, 90.7, 55.8, 55.4, 21.0 (d, J = 4.3 Hz).

^{19}F NMR (376 MHz, $CDCl_3$) δ: −118.3.

^{31}P NMR (162 MHz, $CDCl_3$) δ: 29.2.

HRMS (ESI) was calculated for $[C_{16}H_{17}FO_3 + H]^+$ $[M + H]^+$: m/z 277.1234, found = 277.1224.

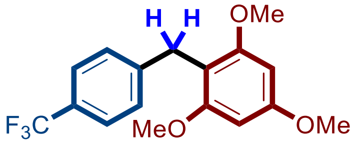

12p

1,3,5-trimethoxy-2-(4-(trifluoromethyl)benzyl)benzene (12p):

Prepared according to general Procedure-B using phosphonium salt (**11i**) (0.2 mmol, 147 mg) product (**12p**) was isolated in (37 mg, 82% yield) as a colorless solid.

R_f = 0.40 (5% EtOAc in hexane).

^{1}H NMR (400 MHz, $CDCl_3$) δ: 7.46 (d, J = 8.0 Hz, 2H), 7.33 (d, J = 7.9 Hz, 2H), 6.16 (s, 2H), 3.98 (s, 2H), 3.82 (s, 3H), 3.80 (s, 6H).

^{13}C NMR (101 MHz, $CDCl_3$) δ: 160.1, 158.9, 146.6, 128.8, 124.9 (q, J = 3.8 Hz), 109.3, 90.7, 55.8, 55.4, 28.4.

^{19}F NMR (376 MHz, $CDCl_3$) δ: −62.1.

HRMS (ESI) was calculated for $[C_{17}H_{17}F_3O_3+H]^+$ $[M+H]^+$: m/z 327.1202, found = 327.1190.

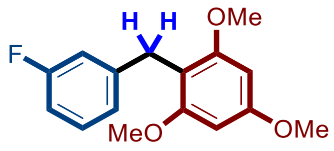

12q

2-(3-fluorobenzyl)-1,3,5-trimethoxybenzene (12q):

Prepared according to general Procedure-B using phosphonium salt (**1k**) (0.2 mmol, 159 mg) product (**12q**) was isolated in (40 mg, 72% yield) as a colorless oil.

R_f = 0.38 (5% EtOAc in hexane).

^{1}H NMR (400 MHz, $CDCl_3$) δ: 7.21 - 7.11 (m, 1H), 7.05 - 6.98 (m, 1H), 6.96 - 6.87 (m, 1H), 6.85 - 6.75 (m, 1H), 6.15 (s, 2H), 3.92 (s, 2H), 3.81 (s, 3H), 3.79 (s, 6H).

^{13}C NMR (101 MHz, $CDCl_3$) δ: 162.9 (d, J = 243.8 Hz), 159.9, 158.9, 145.1 (d, J = 7.2 Hz), 129.3 (d, J = 8.3 Hz), 124.2 (d, J = 2.7 Hz), 115.3 (d, J = 21.0 Hz), 112.2 (d, J = 21.2 Hz), 109.6, 90.7, 55.8, 55.4, 28.2 (d, J = 1.8 Hz).

^{19}F NMR (376 MHz $CDCl_3$) δ: −114.6.

HRMS (ESI) was calculated for $[C_{16}H_{17}FO_3 + H]^+$ $[M + H]^+$: m/z 277.1234, found = 277.1223.

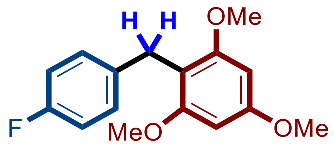

12r

2-(4-fluorobenzyl)-1,3,5-trimethoxybenzene (12r):

Prepared according to general Procedure-B using phosphonium salt (**11y**) (0.2 mmol, 137 mg) product (**12r**) was isolated in (50 mg, 90% yield) as a colorless solid.

R_f = 0.35 (5% EtOAc in hexane).

^{1}H NMR (400 MHz, $CDCl_3$) δ: 7.23 - 7.17 (m, 2H), 6.94 - 6.85 (m, 2H), 6.17 (s, 2H), 3.91 (s, 2H), 3.82 (s, 3H), 3.80 (s, 6H).

^{13}C NMR (101 MHz, $CDCl_3$) δ 162.3, 159.9, 159.8, 158.8, 138.0 (d, J = 3.0 Hz), 129.8 (d, J = 7.7 Hz), 114.7 (d, J = 20.9 Hz), 110.3, 90.7, 55.8, 55.4, 27.6.

^{19}F NMR (376 MHz, $CDCl_3$) δ: −118.8.

The spectral data are consistent with those reported in the literature.[18]

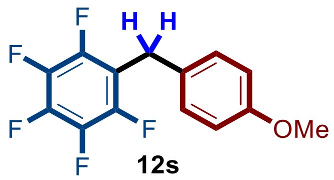

12s

1,2,3,4,5-pentafluoro-6-(4-methoxybenzyl)benzene (12s):

Prepared according to general Procedure-B using phosphonium salt (**11v**) (0.2 mmol, 143 mg) product (**12s**) was isolated in (48 mg, 83% yield) as a colorless oil.

R_f = 0.44 (5% EtOAc in hexane).

^{1}**H NMR** (400 MHz, $CDCl_3$) δ: 7.21 - 7.13 (m, 2H), 6.86 - 6.81 (m, 2H), 3.96 (t, J = 2.1 Hz, 2H), 3.78 (s, 3H).

The spectral data are consistent with those reported in the literature.[19]

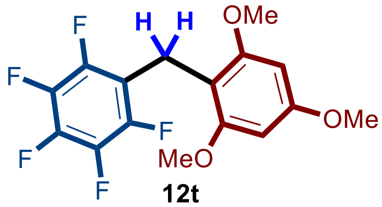

12t

1,2,3,4,5-pentafluoro-6-(2,4,6-trimethoxybenzyl)benzene (12t):

Prepared according to general Procedure-B using phosphonium salt (**11h**) (0.2 mmol, 151 mg) product (**12t**) was isolated in (42 mg, 60% yield) as a colorless solid.

R_f = 0.43 (5% EtOAc in hexane).

^{1}**H NMR** (400 MHz, $CDCl_3$) δ: 6.10 (s, 2H), 3.95 (s, 2H), 3.80 (s, 3H), 3.77 (s, 6H).

^{13}**C NMR** (101 MHz, $CDCl_3$) δ: 160.4, 159.0, 132.4 (d, J = 10.7 Hz), 128.6 (d, J = 12.6 Hz), 106.4, 90.6, 55.8, 55.4, 29.8.

^{19}**F NMR** (376 MHz, $CDCl_3$) δ: −142.9 (dd, J = 14.7, 7.2 Hz), −159.7 (t, J = 20.9 Hz), −164.5 - −164.9 (m).

HRMS (ESI) was calculated for $[C_{16}H_{13}F_5O_3+H]^+$ $[M+H]^+$: m/z 349.0857, found = 349.0852.

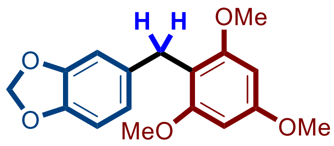

12u

5-(2,4,6-trimethoxybenzyl)benzo[d][1,3]dioxole (12u):

Prepared according to general Procedure-B using phosphonium salt (**11f**) (0.2 mmol, 142 mg) product (**12u**) was isolated in (54 mg, 90% yield) as a colorless solid.

R_f = 0.35 (5% EtOAc in hexane).

^{1}H NMR (400 MHz, $CDCl_3$) δ: 6.78 (dd, J = 1.7, 0.5 Hz, 1H), 6.76 - 6.72 (m, 1H), 6.68 (dd, J = 7.9, 0.4 Hz, 1H), 6.16 (s, 2H), 5.87 (s, 2H), 3.86 (s, 2H), 3.82 (s, 3H), 3.81 (s, 6H).

^{13}C NMR (101 MHz, $CDCl_3$) δ: 159.7, 158.8, 147.3, 145.2, 136.3, 121.2, 110.5, 109.2, 107.9, 100.6, 90.7, 55.8, 55.4, 28.0.

HRMS (ESI) was calculated for $[C_{17}H_{18}O_5+H]^+$ $[M+H]^+$: m/z 303.1227, found = 303.1212.

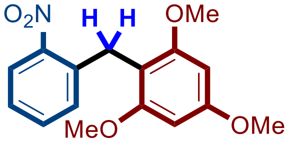

12v

1,3,5-trimethoxy-2-(2-nitrobenzyl)benzene (12v):

Prepared according to general Procedure-B using phosphonium salt (**11g**) (0.2 mmol, 142 mg) product (**12v**) was isolated in (48 mg, 80% yield) as a yellow liquid.

R_f = 0.32 (10% EtOAc in hexane).

^{1}H NMR (400 MHz, $CDCl_3$) δ: 7.78 (dd, J = 8.1, 1.4 Hz, 1H), 7.35 (td, J = 7.6, 1.4 Hz, 1H), 7.28 - 7.18 (m, 1H), 7.15 (d, J = 6.4 Hz, 1H), 6.13 (s, 2H), 4.22 (s, 2H), 3.81 (s, 3H), 3.73 (s, 6H).

^{13}C NMR (101 MHz, $CDCl_3$) δ: 160.3, 158.9, 150.1, 136.9, 132.4, 130.8, 126.1, 123.9, 108.3, 90.5, 55.7, 55.4, 24.4.

The spectral data are consistent with those reported in the literature.[18]

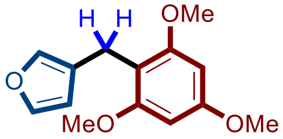

12w

3-(2,4,6-trimethoxybenzyl)furan (12w):

Prepared according to general Procedure-B using phosphonium salt (**11ah**) (0.2 mmol, 131 mg) product (**12w**) was isolated in (40 mg, 80% yield) as a colorless solid.

R_f = 0.24 (5% EtOAc in hexane).

^{1}H NMR (400 MHz, $CDCl_3$) δ: 7.28 - 7.25 (m, 1H), 7.17 (dq, J = 1.9, 1.0 Hz, 1H), 6.30 (dd, J = 1.9, 0.8 Hz, 1H), 6.15 (s, 2H), 3.82 (s, 6H), 3.81 (s, 3H), 3.70 (d, J = 1.0 Hz, 2H).

^{13}C NMR (101 MHz, $CDCl_3$) δ: 158.7, 157.7, 141.2, 138.3, 123.9, 110.7, 109.0, 89.7, 54.8, 54.4, 16.9.

HRMS (ESI) was calculated for $[C_{14}H_{16}O_4+H]^+$ $[M+H]^+$: m/z 294.1121, found = 249.1115.

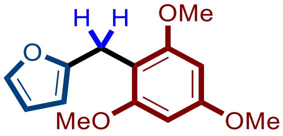

12x

2-(2,4,6-trimethoxybenzyl)furan (12x):

Prepared according to general Procedure-B using phosphonium salt (**11e**) (0.2 mmol, 134 mg) product (**12x**) was isolated in (34 mg, 67% yield) as a colorless oil.

R_f = 0.24 (5% EtOAc in hexane).

^{1}H NMR (400 MHz, $CDCl_3$) δ: 7.18 - 7.15 (m, 1H), 6.15 - 6.11 (m, 1H), 6.07 (s, 2H), 5.73 - 5.70 (m, 1H), 3.84 (s, 2H), 3.72 (s, 3H), 3.70 (s, 6H).

^{13}C NMR (101 MHz, $CDCl_3$) δ: 160.0, 159.1, 155.8, 140.4, 110.2, 107.3, 104.6, 90.8, 55.9, 55.4, 21.6.

HRMS (ESI) was calculated for $[C_{14}H_{16}O_4 + H]^+$ $[M + H]^+$: m/z 249.1121, found = 249.1113.

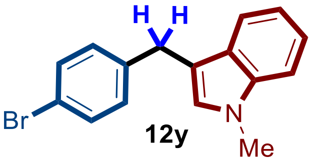

12y

3-(4-bromobenzyl)-1-methyl-1H-indole (12y):

Prepared according to general Procedure-B using phosphonium salt (**11ab**) (0.2 mmol, 142 mg) product (**12y**) was isolated in (43 mg, 72% yield) as a pink solid.

R_f = 0.54 (5% EtOAc in hexane).

^{1}H NMR (400 MHz, $CDCl_3$) δ: 7.38 (dt, *J* = 7.9, 1.0 Hz, 1H), 7.33 - 7.26 (m, 2H), 7.27 - 7.22 (m, 1H), 7.20 (dt, *J* = 8.3, 1.0 Hz, 1H), 7.18 - 7.09 (m, 1H), 7.09 - 7.02 (m, 2H), 6.98 (ddd, *J* = 8.0, 6.9, 1.1 Hz, 1H), 6.66 (s, 1H), 3.96 (s, 2H), 3.64 (s, 3H).

The spectral data are consistent with those reported in the literature.[20]

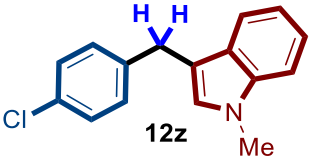

12z

3-(4-chlorobenzyl)-1-methyl-1H-indole (12z):

Prepared according to general Procedure-B using phosphonium salt (**11aa**) (0.2 mmol, 133 mg) product (**12z**) was isolated in (35 mg, 70% yield) as a colorless solid.

R_f = 0.54 (5% EtOAc in hexane).

^{1}H NMR (400 MHz, $CDCl_3$) δ: 7.45 (dt, *J* = 7.9, 1.0 Hz, 1H), 7.32 - 7.26 (m, 1H), 7.23 - 7.15 (m, 5H), 7.05 (ddd, *J* = 8.0, 6.9, 1.1 Hz, 1H), 6.73 (s, 1H), 4.04 (s, 2H), 3.71 (s, 3H).

The spectral data are consistent with those reported in the literature.[20]

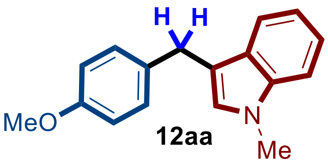

12aa

3-(4-methoxybenzyl)-1-methyl-1H-indole (12aa):

Prepared according to general Procedure-B using phosphonium salt (**11ai**) (0.16 mmol, 112 mg) product (**12aa**) was isolated in (17 mg, 40% yield) as a brown oil.

R_f = 0.54 (5% EtOAc in hexane).

^{1}H NMR (400 MHz, CD_3CN) δ: 7.45 (tt, J = 8.0, 1.0 Hz, 1H), 7.32 (tt, J = 8.2, 0.9 Hz, 1H), 7.22 - 7.20 (m, 1H), 7.20 - 7.12 (m, 2H), 7.00 (ddt, J = 8.0, 7.0, 1.0 Hz, 1H), 6.93 (s, 1H), 6.82 (tt, 2H), 4.00 (s, 2H), 3.73 (s, 3H), 3.71 (s, 3H).

The spectral data are consistent with those reported in the literature. [21]

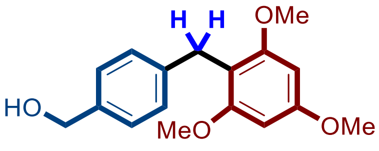

12ab

(4-(2,4,6-trimethoxybenzyl)phenyl)methanol (12ab):

Prepared according to general Procedure-B using phosphonium salt (**11m**) (0.2 mmol, 140 mg) product (**12ab**) was isolated in (40 mg, 68% yield) as a colorless oil.

R_f = 0.43 (30% EtOAc in hexane).

H NMR (400 MHz, $CDCl_3$) δ: 7.25 - 7.18 (m, 4H), 6.16 (s, 2H), 4.60 (s, 2H), 3.94 (s, 2H), 3.81 (s, 3H), 3.79 (s, 6H).

^{13}C NMR (101 MHz, $CDCl_3$) δ: 159.8, 158.9, 141.9, 137.8, 128.7, 127.0, 110.2, 90.7, 65.5, 55.8, 55.4, 28.1.

HRMS (ESI) was calculated for $[C_{17}H_{20}O_4 + H]^+$ $[M + H]^+$: m/z 289.1434, found = 289.1424.

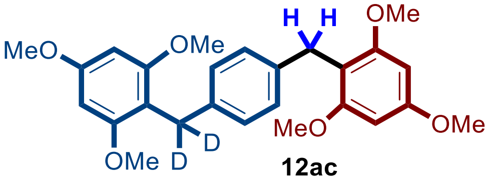

1,3,5-trimethoxy-2-((4-(2,4,6-trimethoxybenzyl)phenyl)methyl-d_2)benzene (12ac):

Prepared according to general Procedure-B using phosphonium salt (**11o**) (0.03 mmol, 30 mg) product (**12ac**) was isolated in (10 mg, 71% yield) as a colorless oil.

R_f = 0.3 (5% EtOAc in hexane).

^{1}H NMR (400 MHz, $CDCl_3$) δ: 7.07 (s, 4H), 6.12 (s, 4H), 3.85 (s, 2H), 3.79 (s, 6H), 3.76 (s, 12H).

^{13}C NMR (101 MHz, $CDCl_3$) δ: 159.6, 158.9, 139.0, 128.2, 128.1, 90.7, 55.8, 55.4, 27.9.

HRMS (ESI) was calculated for $[C_{26}H_{28}D_2O_6 + H]^+$ $[M + H]^+$: m/z 441.2240, found = 441.2223.

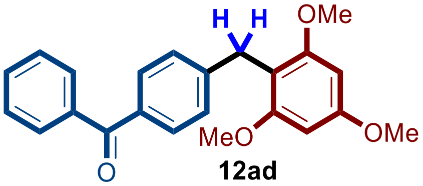

phenyl(4-(2,4,6-trimethoxybenzyl)phenyl)methanone (12ad):

Prepared according to general Procedure-B using phosphonium salt (**11p**) (0.03 mmol, 23 mg) product (**12ad**) was isolated in (9 mg, 84% yield) as a colorless oil.

R_f = 0.5 (10% EtOAc in hexane).

^{1}H NMR (400 MHz, $CDCl_3$) δ: 7.77 (d, J = 7.4 Hz, 2H), 7.68 (d, J = 8.0 Hz, 2H), 7.55 (t, J = 7.4 Hz, 1H), 7.45 (t, J = 7.5 Hz, 2H), 7.32 (d, J = 8.0 Hz, 2H), 6.16 (s, 2H), 4.00 (s, 2H), 3.82 (s, 3H), 3.80 (s, 6H).

^{13}C NMR (101 MHz, $CDCl_3$) δ: 196.8, 160.1, 158.9, 147.9, 138.2, 134.8, 132.1, 130.3, 130.1, 128.5, 128.2, 109.4, 90.7, 55.8, 55.5, 28.6.

HRMS (ESI) was calculated for $[C_{23}H_{22}O_4 + H]^+$ $[M + H]^+$: m/z 363.1590, found = 363.1577.

2.4. NMR analysis for the reduction reaction of benzhydryl phosphonium salt (11a$_d$) giving product (12i).

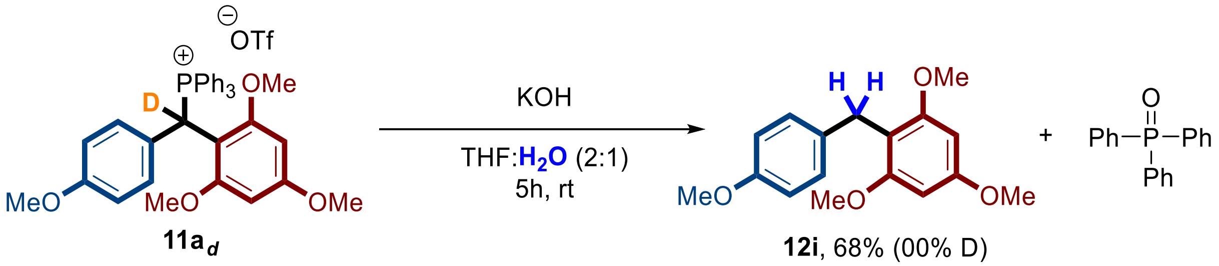

Observations:

When applying the reduction reaction conditions [KOH in THF:H_2O] with the deuterium incorporated benzhydryl phosphonium salt **(11a$_d$)**, we observed the disappearance of the deuterium and replaced by proton as can be seen in the ^{1}H-NMR. Suggesting that the reaction goes throw the formation of ylide. Crude ^{31}P-NMR spectrum for the reaction shows the complete disappearance of the 23.6 ppm peak associated with the starting benzhydryl phosphonium salt **(11a$_d$)**, and the presence of only 31.9 ppm peak which is associated with triphenyl phosphonium oxide (Ph_3P=O).

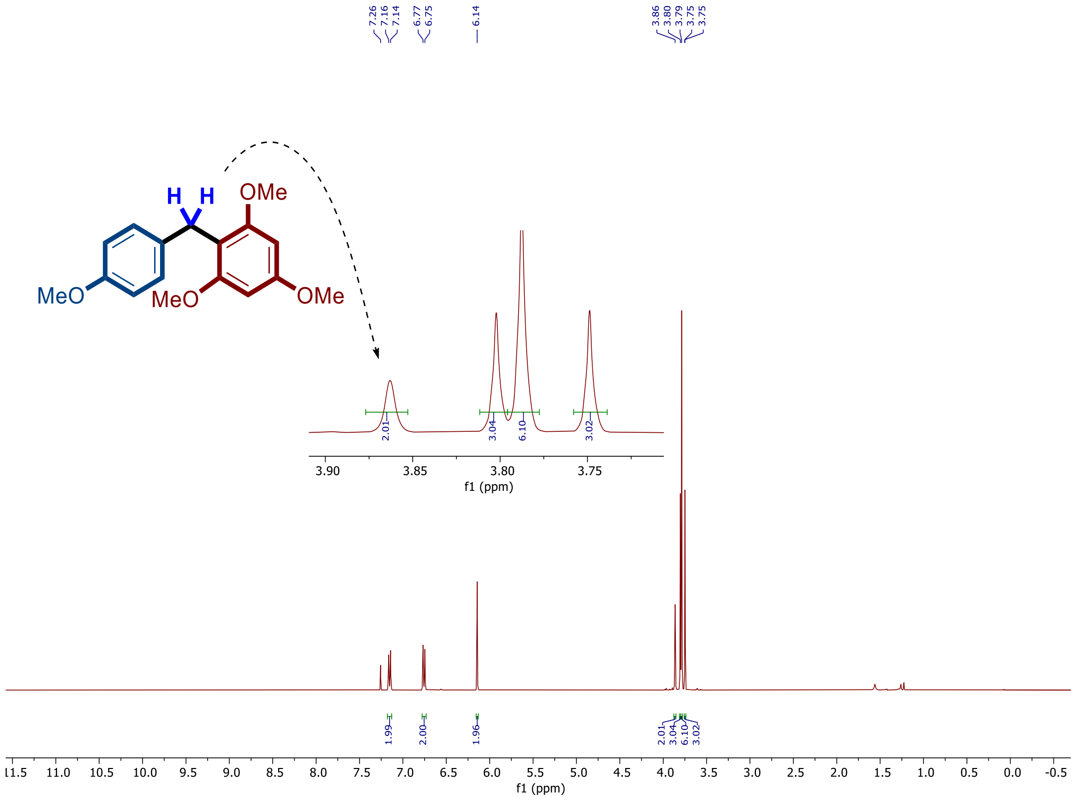

^{1}H NMR (400 MHz, $CDCl_3$) of compound (**12i**)

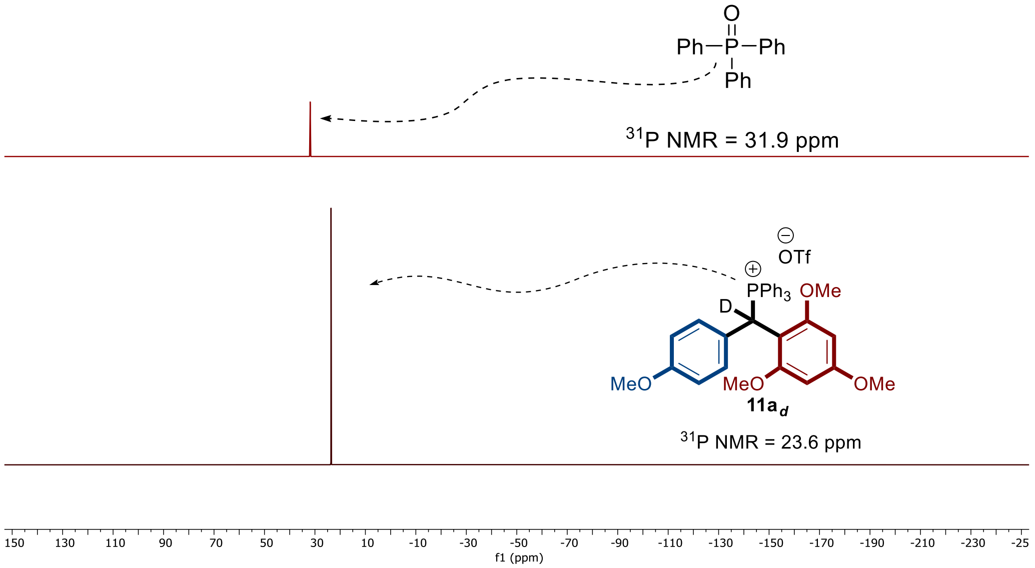

Crude ^{31}P-NMR spectrum (162 MHz, THF:H_2O**)** of the reaction after 1 h of **11a*d***

2.5. General Procedure-C and Characterization for Diarylmethanes-D_2 Products (13)

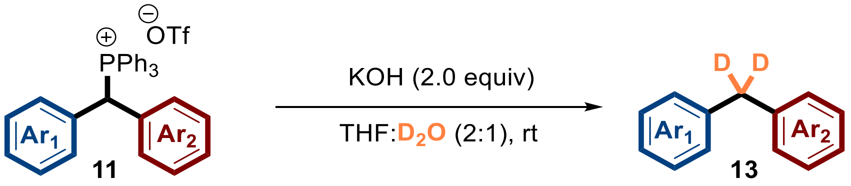

A 7 mL screw-cap vial containing a Teflon-coated magnetic stirring bar was charged with benzhydryl phosphonium salt **11** (0.2 mmol, 1.0 equivalent), 2 mL of THF, and 1 mL of D_2O. The mixture was stirred at room temperature for five minutes. Powder KOH (0.4 mmol, 2.0 equivalents) was then added in a single portion. The progress of the reaction was monitored using thin-layer chromatography (TLC). The reaction mixture was quenched with D_2O, and the aqueous phase was extracted with EtOAc (2 x 5 mL). The combined organic phases were dried over $NaSO_4$, filtered and concentrated under reduced pressure (by evaporator). The crude material was purified by column chromatography, yielding the desired product (**13**).

Notes: (1) The percentage of deuterium (D%) incorporation for all the deuterated molecules were determined by ^{1}H-NMR. (2) Products **13n** and **13x** were analyzed with ^{2}H-NMR.

Table - S4: Scope of the reduction reaction of deuterated products (**13**).

Entry	Phosphonium Salts (11)	Diarylmethanes-D_2 (13)	Time	Yield[a] (D % incorporation)
1.	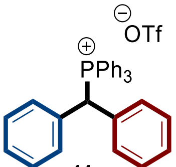 **11q**	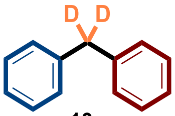 **13a**	1h	96% (96% D)
2.	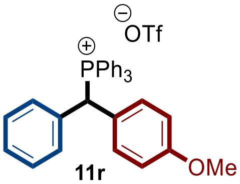 **11r**	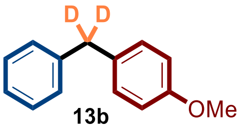 **13b**	1h	86% (96% D)

3.	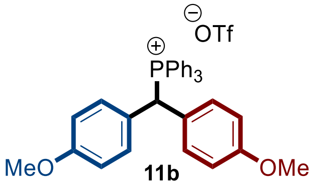 **11b**	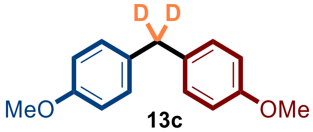 **13c**	5h	64% (96% D)
4.	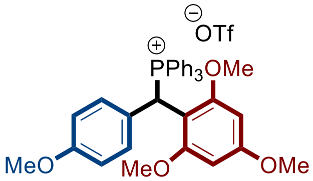 **11t**	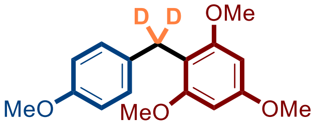 **13d**	5h	89% (96% D)
5.	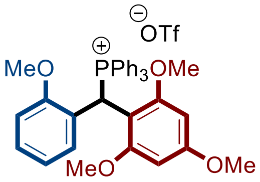 **11l**	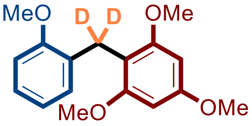 **13e**	1h	89% (96% D)
6.	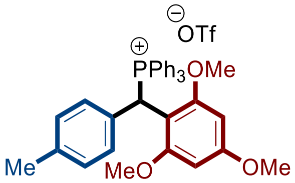 **11ad**	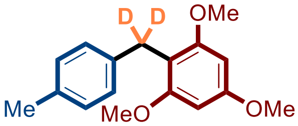 **13f**	5h	82% (96% D)
7.	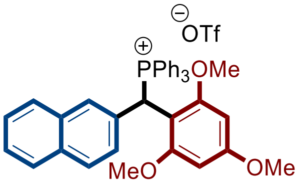 **11af**	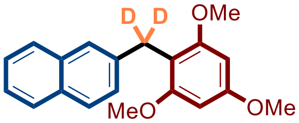 **13g**	5h	80% (97% D)
8.	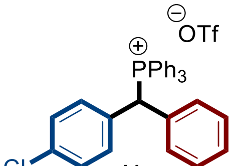 **11ag**	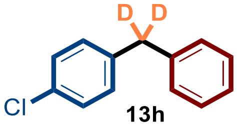 **13h**	1h	83% (96% D)
9.	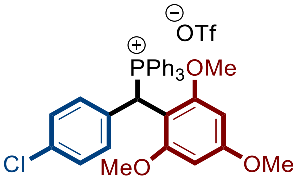 **11z**	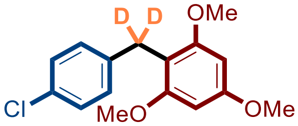 **13i**	5h	90% (97% D)

10.	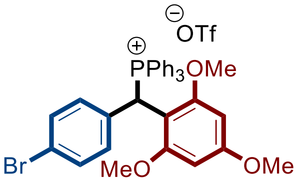 **11w**	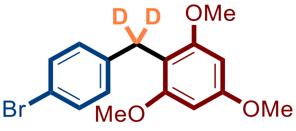 **13j**	5h	67% (97% D)
11.	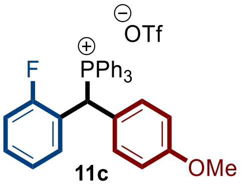 **11c**	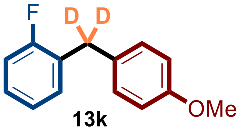 **13k**	3h	74% (94% D)
12.	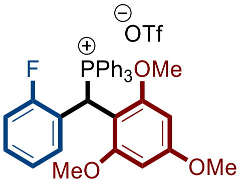 **11j**	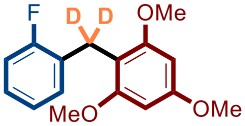 **13l**	5h	87% (93% D)
13.	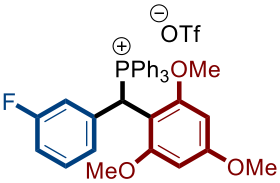 **11k**	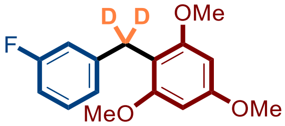 **13m**	1h	87% (94% D)
14.	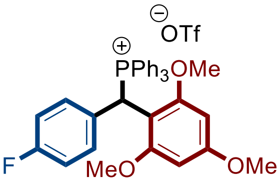 **11y**	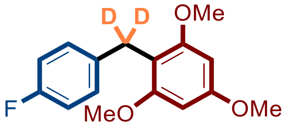 **13n**	5h	90% (99% D)
15.	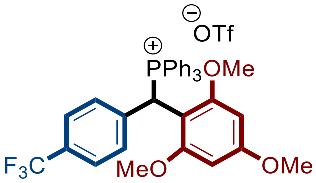 **11i**	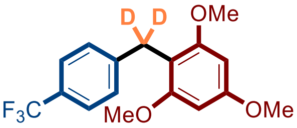 **13o**	5h	80% (99% D)
16.	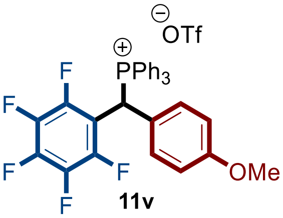 **11v**	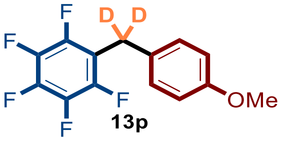 **13p**	3 days	96% (94% D)

17.	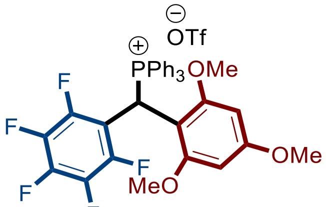 **11h**	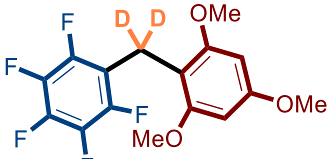 **13q**	5h	90% (97% D)
18.	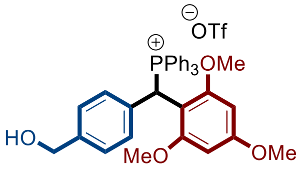 **11m**	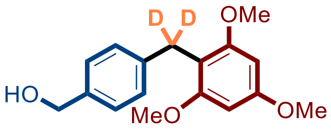 **13r**	2h	85% (96% D)
19.	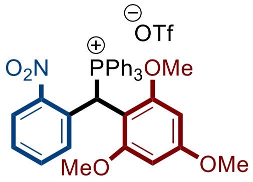 **11g**	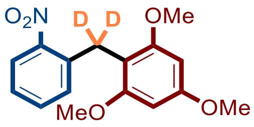 **13s**	5h	75% (95% D)
20.	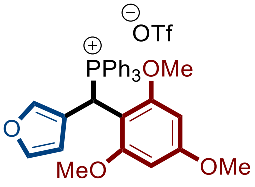 **11ah**	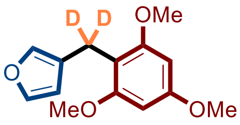 **13t**	5h	50% (99% D)
21.	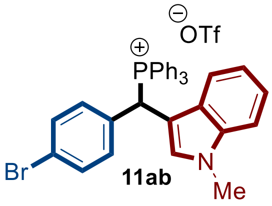 **11ab**	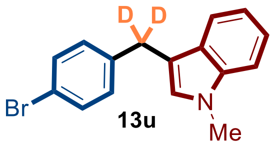 **13u**	5h	71% (97% D)
22.	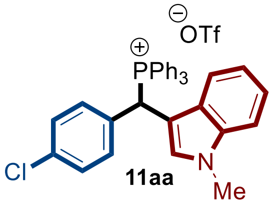 **11aa**	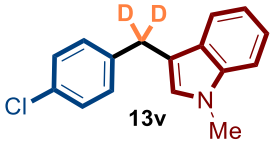 **13v**	5h	60% (97% D)
23.	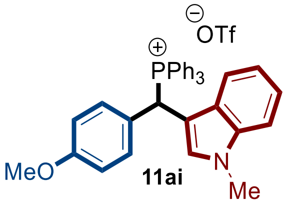 **11ai**	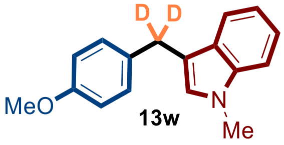 **13w**	3h	45% (96% D)

24.	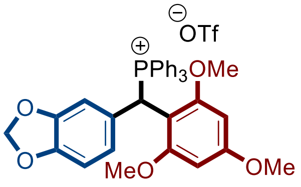<br>**11f**	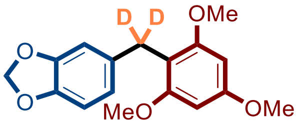<br>**13x**	5h	87%<br>(99% D)
25.	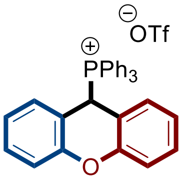<br>**11d**	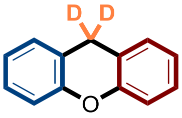<br>**13y**	5h	83%<br>(96% D)

[a] Isolated yield.

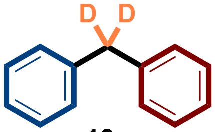

13a

Diphenylmethane-d2 (13a):

Prepared according to general Procedure-C using phosphonium salt (**11q**) (0.18 mmol, 159 mg) product (**13a**) was isolated in (29 mg, 96% yield) as a colorless oil. 96% Deuterium-incorporation. R_f = 0.43 (5% EtOAc in hexane).

^{1}**H NMR** (400 MHz, $CDCl_3$) δ: 7.23 - 7.16 (m, 4H), 7.14 - 7.07 (m, 6H), 3.88 (brs, 0.08H, 96% labeled).

The spectral data are consistent with those reported in the literature.[22]

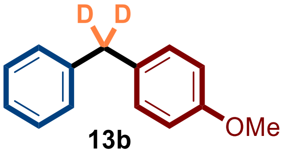

13b

1-methoxy-4-(phenylmethyl-d$_2$)benzene (13b):

Prepared according to general Procedure-C using phosphonium salt (**11r**) (0.3 mmol, 185 mg) product (**13b**) was isolated in (52 mg, 86% yield) as a colorless oil. 96% Deuterium-incorporation. R_f = 0.40 (5% EtOAc in hexane).

^{1}**H NMR** (400 MHz, $CDCl_3$) δ: 7.33 - 7.28 (m, 2H), 7.25 - 7.18 (m, 3H), 7.16 - 7.11 (m, 2H), 6.89 - 6.83 (m, 2H), 3.80 (s, 3H).

^{13}**C NMR** (101 MHz, $CDCl_3$) δ: 158.0, 141.5, 133.2, 129.9, 128.8, 128.5, 126.0, 113.9, 55.3, 40.3 (p, J = 19.3 Hz).

The spectral data are consistent with those reported in the literature.[22]

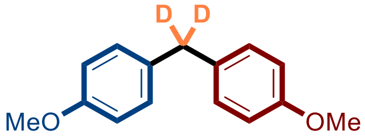

13c

Bis(4-methoxyphenyl)methane-d₂ (13c):

Prepared according to general Procedure-C using phosphonium salt (**11b**) (0.2 mmol, 148 mg) product (**13c**) was isolated in (34 mg, 64% yield) as a colorless oil. 96% Deuterium-incorporation.

R_f = 0.39 (5% EtOAc in hexane).

^{1}**H NMR** (400 MHz, $CDCl_3$) δ: 7.13 - 7.06 (m, 4H), 6.87 - 6.80 (m, 4H), 3.79 (s, 6H).

^{13}**C NMR** (101 MHz, $CDCl_3$) δ: 158.0, 133.8, 129.8, 113.9, 55.4.

The spectral data are consistent with those reported in the literature.[23]

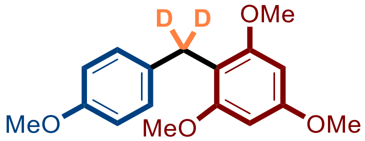

13d

1,3,5-trimethoxy-2-((4-methoxyphenyl)methyl-d₂)benzene (13d):

Prepared according to general Procedure-C using phosphonium salt (**11t**) (0.2 mmol, 144 mg) product (**13d**) was isolated in (68 mg, 89% yield) as a colorless oil. 96% Deuterium-incorporation.

A 1.0-gram scale of **11t** reaction gave product **13d** (357 mg, 86% yield, 94% D).

R_f = 0.54 (5% EtOAc in hexane).

^{1}**H NMR** (400 MHz, $CDCl_3$) δ: 7.21 - 7.16 (m, 2H), 6.81 - 6.76 (m, 2H), 6.17 (s, 2H), 3.90 (brs, 0.08H, 96% labeled), 3.82 (s, 3H), 3.81 (s, 6H), 3.77 (s, 3H).

^{13}**C NMR** (101 MHz, $CDCl_3$) δ: 159.6, 158.9, 157.5, 134.5, 129.4, 113.5, 110.7, 90.7, 55.8, 55.4, 55.3, 27.1- 26.5 (m).

HRMS (ESI) was calculated for $[C_{17}H_{18}D_2O_4 + H]^+$ $[M + H]^+$: m/z 291.1559, found = 291.1548.

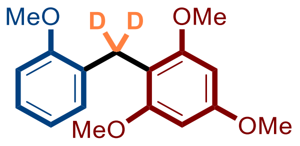

13e

1,3,5-trimethoxy-2-((2-methoxyphenyl)methyl-d₂)benzene (13e):

Prepared according to general Procedure-C using phosphonium salt (**11l**) (0.2 mmol, 150 mg) product (**13e**) was isolated in (52 mg, 89% yield) as a colorless oil. 96% Deuterium-incorporation. R_f = 0.53 (5% EtOAc in hexane).

^{1}H NMR (400 MHz, $CDCl_3$) δ: 7.16 - 7.11 (m, 1H), 6.87 - 6.80 (m, 2H), 6.67 (ddd, J = 8.2, 2.6, 1.0 Hz, 1H), 6.15 (s, 2H), 3.81 (s, 3H), 3.80 (s, 6H), 3.77 (s, 3H).

^{13}C NMR (101 MHz, $CDCl_3$) δ: 159.8, 159.4, 158.9, 143.9, 128.9, 121.1, 114.5, 110.5, 110.1, 90.7, 55.8, 55.4, 55.1.

HRMS (ESI) was calculated for $[C_{17}H_{18}D_2O_4 + H]^+$ $[M + H]^+$: m/z 291.1559, found = 291.1555.

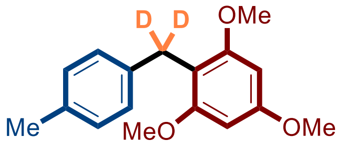

13f

1,3,5-trimethoxy-2-(p-tolylmethyl-d2)benzene (13f):

Prepared according to general Procedure-C using phosphonium salt (**11ad**) (0.2 mmol, 136 mg) product (**13f**) was isolated in (44 mg, 82% yield) as a yellow solid. 96% Deuterium-incorporation. R_f = 0.40 (5% EtOAc in hexane).

^{1}H NMR (400 MHz, $CDCl_3$) δ: 7.13 (d, J = 8.2 Hz, 2H), 7.03 (d, J = 8.2 Hz, 2H), 6.15 (s, 2H), 3.81 (s, 3H), 3.79 (s, 6H), 2.29 (s, 3H).

^{13}C NMR (101 MHz, $CDCl_3$) δ: 159.7, 159.0, 158.9, 139.2, 134.7, 128.8, 128.4, 110.5, 90.8, 90.7, 55.8, 55.4, 29.8, 27.5 - 27.2 (m), 21.1.

HRMS (ESI) was calculated for $[C_{17}H_{18}D_2O_3+H]^+$ $[M+H]^+$: m/z 275.1610, found = 275.1601.

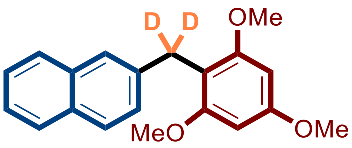

13g

2-((2,4,6-trimethoxyphenyl)methyl-d2)naphthalene (13g):

Prepared according to general Procedure-C using phosphonium salt (**11af**) (0.2 mmol, 143 mg) product (**13g**) was isolated in (46 mg, 80% yield) as a colorless solid. 97% Deuterium-incorporation.

R$_f$ = 0.48 (5% EtOAc in hexane).

^{1}H NMR (400 MHz, $CDCl_3$) δ; 8.36 (d, J = 8.4 Hz, 1H), 7.90 - 7.84 (m, 1H), 7.69 (dt, J = 8.4, 1.1 Hz, 1H), 7.57 (ddd, J = 8.4, 6.8, 1.4 Hz, 1H), 7.50 (ddd, J = 8.1, 6.8, 1.3 Hz, 1H), 7.32 (dd, J = 8.2, 7.1 Hz, 1H), 6.97 (dd, J = 7.2, 1.3 Hz, 1H), 6.25 (s, 2H), 3.88 (s, 3H), 3.75 (s, 6H).

^{13}C NMR (101 MHz, $CDCl_3$) δ: 160.1, 159.5, 137.4, 133.7, 132.5, 128.6, 126.1, 125.7, 125.5, 125.3, 124.3, 124.1, 108.7, 90.8, 55.8, 55.5, 24.9 (p, J = 21.3 Hz).

HRMS (ESI) was calculated for $[C_{20}H_{18}D_2O_3+H]^+$ $[M+H]^+$: m/z 311.1610, found = 311.1601.

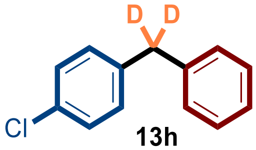

13h

1-chloro-4-(phenylmethyl-d2)benzene (13h):

Prepared according to general Procedure-C using phosphonium salt (**11ag**) (0.1 mmol, 110 mg) product (**13h**) was isolated in (31 mg, 83% yield) as a colorless oil. 96% Deuterium-incorporation.

R$_f$ = 0.54 (5% EtOAc in hexane).

^{1}H NMR (400 MHz, $CDCl_3$) δ: 7.23 - 7.16 (m, 3H), 7.16 - 7.10 (m, 2H), 7.09 - 7.05 (m, 2H), 7.04 - 7.00 (m, 2H), 3.95 (brs, 0.09H, 96% labeled).

^{13}C NMR (101 MHz, $CDCl_3$) δ: 140.6, 139.6, 132.0, 130.4, 128.9, 128.7, 126.4, 41.0 - 40.5 (m).

HRMS (ESI) was calculated for $[C_{13}H_9D_2Cl + H]^+$ $[M + H]^+$: m/z 205.0853, found = 205.0859.

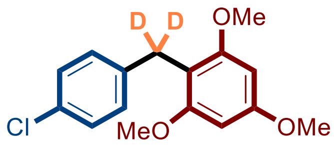

13i

2-((4-chlorophenyl)methyl-d2)-1,3,5-trimethoxybenzene (13i):

Prepared according to general Procedure-C using phosphonium salt (**11z**) (0.2 mmol, 140 mg) product (**13i**) was isolated in (56 mg, 90% yield) as a colorless solid. 97% Deuterium-incorporation.

R_f = 0.40 (5% EtOAc in hexane).

^{1}H NMR (400 MHz, $CDCl_3$) δ: 7.17 (s, 4H), 6.16 (s, 2H), 3.81 (s, 3H), 3.79 (s, 6H).

^{13}C NMR (101 MHz, $CDCl_3$) δ: 159.9, 158.8, 140.8, 130.9, 129.9, 128.1, 109.8, 90.7, 55.8, 55.4, 27.6 - 26.9 (m).

HRMS (ESI) was calculated for $[C_{16}H_{15}D_2ClO_3+H]^+$ $[M+H]^+$: m/z 295.1064, found = 295.1053.

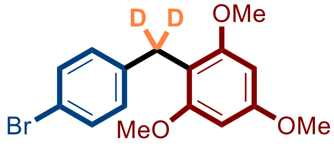

13j

2-((4-bromophenyl)methyl-d2)-1,3,5-trimethoxybenzene (13j):

Prepared according to general Procedure-C using phosphonium salt (**11w**) (0.2 mmol, 149 mg) product (**13j**) was isolated in (45 mg, 67% yield) as a colorless solid. 97% Deuterium-incorporation.

R_f = 0.4 (5% EtOAc in hexane).

^{1}H NMR (400 MHz, $CDCl_3$) δ: 7.34 - 7.29 (m, 2H), 7.13 - 7.08 (m, 2H), 6.14 (s, 2H), 3.86 (brs, 0.05H, 97% labeled), 3.81 (s, 3H), 3.78 (s, 6H),

^{13}C NMR (101 MHz, $CDCl_3$) δ: 160.0, 158.9, 141.4, 131.1, 130.4, 119.1, 109.8, 90.8, 55.8, 55.4, 27.6 - 27.1 (m).

HRMS (ESI) was calculated for $[C_{16}H_{15}D_2BrO_3+H]^+$ $[M+H]^+$: m/z 339.0559, found = 339.0555.

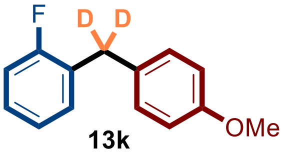

13k

1-fluoro-2-((4-methoxyphenyl)methyl-d2)benzene (13k):

Prepared according to general Procedure-C using phosphonium salt (**11c**) (0.2 mmol, 158 mg) product (**13k**) was isolated in (41 mg, 74% yield) as a colorless oil. 94% Deuterium-incorporation. R_f = 0.43 (5% EtOAc in hexane).

^{1}H NMR (400 MHz, $CDCl_3$) δ: 7.24 - 7.12 (m, 4H), 7.10 - 7.01 (m, 2H), 6.89 - 6.82 (m, 2H), 3.80 (s, 3H).

^{13}C NMR (101 MHz, $CDCl_3$) δ: 161.0 (d, J = 245.2 Hz), 158.2, 132.0, 131.0 (d, J = 4.7 Hz), 129.8, 127.9 (d, J = 8.0 Hz), 124.2 (d, J = 3.6 Hz), 115.4 (d, J = 22.0 Hz), 114.0, 55.3, 33.9 - 33.2 (m).

^{19}F NMR (376 MHz, $CDCl_3$) δ: −118.1.

HRMS (ESI) was calculated for $[C_{14}H_{11}D_2FO + H]^+$ $[M + H]^+$: m/z 219.1148, found = 219.1155.

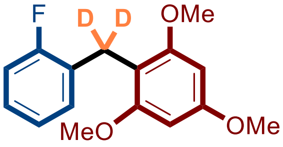

13l

2-((4-fluorophenyl)methyl-d$_2$)-1,3,5-trimethoxybenzene (13l):

Prepared according to general Procedure-C using phosphonium salt (**11j**) (0.3 mmol, 170 mg) product (**13l**) was isolated in (77 mg, 87% yield) as a colorless oil. 93% Deuterium-incorporation. R_f = 0.5 (5% EtOAc in hexane).

^{1}H NMR (400 MHz, $CDCl_3$) δ: 7.12 - 7.06 (m, 1H), 7.01 - 6.95 (m, 1H), 6.94 - 6.89 (m, 2H), 6.17 (s, 2H), 3.83 (s, 3H), 3.76 (s, 6H).

^{13}C NMR (100 MHz, $CDCl_3$) δ: 161.2 (d, J = 244.7 Hz), 160.0, 159.3, 129.9 (d, J = 4.8 Hz), 128.7 (d, J = 15.7 Hz), 126.8 (d, J = 7.9 Hz), 123.6 (d, J = 3.5 Hz), 114.7 (d, J = 22.3 Hz), 108.3, 90.7, 55.8, 55.4.

^{19}F NMR (376 MHz, $CDCl_3$) δ: −118.3.

HRMS (ESI) was calculated for $[C_{16}H_{15}D_2FO_3 + H]^+$ $[M + H]^+$: m/z 279.1360, found = 279.1346.

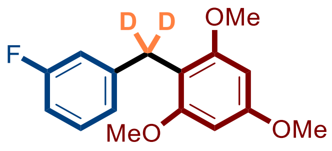

13m

2-((3-fluorophenyl)methyl-d_2)-1,3,5-trimethoxybenzene (13m):

Prepared according to general Procedure-C using phosphonium salt (**11k**) (0.3 mmol, 174 mg) product (**13m**) was isolated in (79 mg, 87% yield) as a colorless oil. 94% Deuterium-incorporation. R_f = 0.45 (5% EtOAc in hexane).

^{1}H NMR (400 MHz, $CDCl_3$) δ: 7.20 - 7.13 (m, 1H), 7.05 - 7.01 (m, 1H), 6.95 - 6.90 (m, 1H), 6.84 - 6.77 (m, 1H), 6.16 (s, 2H), 3.82 (s, 3H), 3.80 (s, 6H).

^{13}C NMR (101 MHz, $CDCl_3$) δ: 162.9 (d, J = 243.8 Hz), 159.9, 158.9, 145.0 (d, J = 7.2 Hz), 129.3 (d, J = 8.3 Hz), 124.2 (d, J = 2.6 Hz), 115.4 (d, J = 21.1 Hz), 112.2 (d, J = 21.2 Hz), 109.6, 55.8, 55.4.

^{19}F NMR (376 MHz, $CDCl_3$) δ: –114.6.

HRMS (ESI) was calculated for $[C_{16}H_{15}D_2FO_3 + H]^+$ $[M + H]^+$: m/z 279.1360, found = 279.1351.

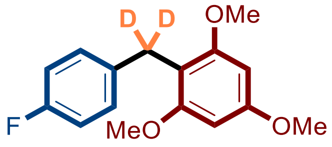

13n

2-((4-fluorophenyl)methyl-d2)-1,3,5-trimethoxybenzene (13n):

Prepared according to general Procedure-C using phosphonium salt (**11y**) (0.2 mmol, 137 mg) product (**13n**) was isolated in (50 mg, 90% yield) as a colorless solid. 99% Deuterium-incorporation. R_f = 0.48 (5% EtOAc in hexane).

^{1}H NMR (400 MHz, $CDCl_3$) δ: 7.21 (t, J = 6.3 Hz, 2H), 6.90 (td, J = 8.6, 1.8 Hz, 2H), 6.17 (s, 2H), 3.82 (s, 3H), 3.81 (s, 6H).

^{13}C NMR (101 MHz, $CDCl_3$) δ: 162.3, 159.9, 159.8, 158.8, 138.0, 137.9, 129.9, 129.8 (d, J = 7.7 Hz), 114.8, 114.6 (d, J = 20.8 Hz), 110.2, 90.7, 55.8, 55.4, 27.1 (p, J = 18.7 Hz).

^{19}F NMR (376 MHz, $CDCl_3$) δ: –118.7.

^{2}H NMR (77 MHz, $CDCl_3$) δ: 3.80.

HRMS (ESI) was calculated for $[C_{16}H_{15}D_2FO_3+H]^+$ $[M+H]^+$: m/z 279.1360, found = 279.1349.

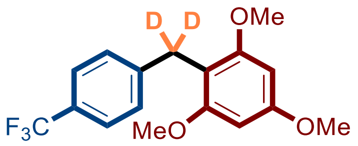

13o

1,3,5-trimethoxy-2-((4-(trifluoromethyl)phenyl)methyl-d2)benzene (13o):

Prepared according to general Procedure-C using phosphonium salt (**11i**) (0.2 mmol, 147 mg) product (**13o**) was isolated in (50 mg, 80% yield) as a colorless solid. 99% Deuterium-incorporation.

R_f = 0.40 (5% EtOAc in hexane).

^{1}H NMR (400 MHz, $CDCl_3$) δ: 7.49 - 7.42 (m, 2H), 7.37 - 7.30 (m, 2H), 6.15 (s, 2H), 3.81 (s, 3H), 3.79 (s, 6H).

^{13}C NMR (101 MHz, $CDCl_3$) δ: 160.1, 158.9, 146.5, 128.8, 125.0, 125.0 (q, J = 3.8 Hz), 124.9, 124.9, 124.9, 109.2, 90.7, 55.8, 55.4.

^{19}F NMR (376 MHz, $CDCl_3$) δ: −62.1.

HRMS (ESI) was calculated for $[C_{17}H_{15}D_2F_3O_3+H]^+$ $[M+H]^+$: m/z 329.1328, found = 329.1313.

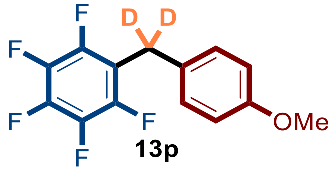

13p

1,2,3,4,5-pentafluoro-6-((4-methoxyphenyl)methyl-d₂)benzene (13p):

Prepared according to general Procedure-C using phosphonium salt (**11v**) (0.2 mmol, 140 mg) product (**13p**) was isolated in (56 mg, 96% yield) as a colorless oil. 94% Deuterium-incorporation.

R_f = 0.45 (5% EtOAc in hexane).

^{1}H NMR (400 MHz, $CDCl_3$) δ: 7.20 - 7.14 (m, 2H), 6.86 - 6.81 (m, 2H), 3.94 (brs, 0.12H, 94% labeled), 3.78 (s, 3H).

^{13}C NMR (101MHz, $CDCl_3$) δ: 158.7, 129.5, 114.3, 55.4.

^{19}F NMR (376 MHz, $CDCl_3$) δ: −143.5 - −143.8 (m), −157.3 - −157.5 (m), −162.3 - −162.6 (m).

HRMS (ESI) was calculated for $[C_{14}H_7D_2F_5O + H]^+$ $[M + H]^+$: m/z 291.0771, found = 291.0772.

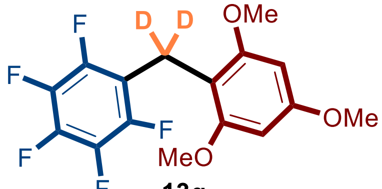

13q

1,2,3,4,5-pentafluoro-6-((2,4,6-trimethoxyphenyl)methyl-d2)benzene (13q):

Prepared according to general Procedure-C using phosphonium salt (**11h**) (0.2 mmol, 151 mg) product (**13q**) was isolated in (62 mg, 90% yield) as a colorless solid. 97 % Deuterium-incorporation.

R_f = 0.43 (5% EtOAc in hexane).

^{1}H NMR (400 MHz, $CDCl_3$) δ: 6.10 (s, 2H), 3.93 (brs, 0.06H, 97% labeled), 3.80 (s, 3H), 3.77 (s, 6H).

^{13}C NMR (101 MHz, $CDCl_3$) δ: 160.4, 159.1, 146.8, 144.4, 133.9 (d, J = 19.5 Hz), 131.2 – 127.1 (m), 106.4, 90.59, 77.3, 55.8, 55.4, 16.5 – 15.8 (m).

^{19}F NMR (376 MHz, $CDCl_3$) δ: −141.8 - −145.0 (m), −159.7 (t, J = 20.8 Hz), −164.1 - −165.9 (m).

HRMS (ESI) was calculated for $[C_{16}H_{11}D_2F_5O_3+H]^+$ $[M+H]^+$: m/z 351.0983, found = 351.0972.

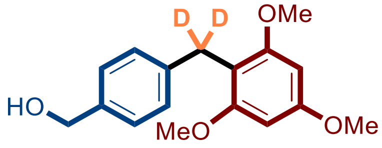

13r

(4-((2,4,6-trimethoxyphenyl)methyl-d$_2$)phenyl)methanol (13r):

Prepared according to general Procedure-C using phosphonium salt (**11m**) (0.2 mmol, 115 mg) product (**13r**) was isolated in (52 mg, 85% yield) as a colorless oil. 96% Deuterium-incorporation.

R_f = 0.43 (30% EtOAc in hexane).

^{1}H NMR (400 MHz, $CDCl_3$) δ: 7.17 - 7.10 (m, 4H), 6.06 (s, 2H), 4.51 (s, 2H), 3.72 (s, 3H), 3.70 (s, 6H).

^{13}C NMR (101 MHz, $CDCl_3$) δ: 159.8, 158.9, 141.9, 137.9, 128.7, 127.0, 110.1, 90.7, 65.5, 55.8, 55.4, 27.9 – 27.2 (m).

HRMS (ESI) was calculated for $[C_{17}H_{18}D_2O_4 + H]^+$ $[M + H]^+$: m/z 291.1559, found = 291.1554.

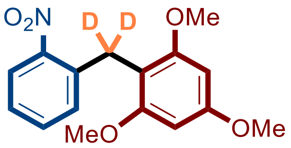

13s

1,3,5-trimethoxy-2-((2-nitrophenyl)methyl-d2)benzene (13s):

Prepared according to general Procedure-C using phosphonium salt (**11g**) (0.2 mmol, 142 mg) product (**13s**) was isolated in (45 mg, 75% yield) as a yellow solid. 95% Deuterium-incorporation.

R_f = 0.29 (5% EtOAc in hexane).

^{1}H NMR (400 MHz, $CDCl_3$) δ: 7.78 (dd, J = 8.2, 1.4 Hz, 1H), 7.35 (ddd, J = 7.8, 7.3, 1.4 Hz, 1H), 7.27 - 7.18 (m, 1H), 7.16 (dd, J = 7.8, 1.6 Hz, 1H), 6.13 (s, 2H), 4.20 (brs, 0.11H, 95% labeled) 3.81 (s, 3H), 3.73 (s, 6H).

^{13}C NMR (101 MHz, $CDCl_3$) δ: 160.3, 158.9, 150.1, 136.8, 132.4, 130.9, 126.2, 123.9, 108.2, 90.5, 77.4, 77.1, 76.8, 55.7.

HRMS (ESI) was calculated for $[C_{16}H_{15}D_2NO_5+H]^+$ $[M+H]^+$: m/z 306.1305, found = 306.1296.

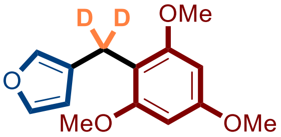

13t

3-((2,4,6-trimethoxyphenyl)methyl-d2)furan (13t):

Prepared according to general Procedure-C using phosphonium salt (**11ah**) (0.2 mmol, 131 mg) product (**13t**) was isolated in (25 mg, 50% yield) as a colorless solid. 99% Deuterium-incorporation.

R_f = 0.24 (5% EtOAc in hexane).

^{1}H NMR (400 MHz, $CDCl_3$) δ: 7.27 - 7.26 (m, 1H), 7.17 (dd, J = 1.6, 0.9 Hz, 1H), 6.30 (dd, J = 1.8, 0.9 Hz, 1H), 6.15 (s, 2H), 3.81 (s, 6H), 3.81 (s, 3H).

^{13}C NMR (101 MHz, $CDCl_3$) δ: 159.7, 158.7, 142.2, 139.3, 124.8, 111.7, 109.9, 90.7, 55.8, 55.4, 17.4 (p, J = 18.7 Hz).

HRMS (ESI) was calculated for $[C_{14}H_{14}D_2O_4+H]^+$ $[M+H]^+$: m/z 251.1246, found = 251.1236.

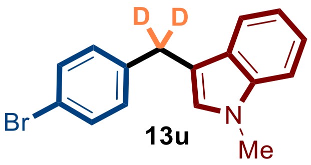

3-((4-bromophenyl)methyl-d2)-1-methyl-1H-indole (13u):

Prepared according to general Procedure-C using phosphonium salt (**11ab**) (0.2 mmol, 131 mg) product (**13u**) was isolated in (43 mg, 71% yield) as a yellow solid. 97% Deuterium-incorporation. R_f = 0.54 (5% EtOAc in hexane).

^{1}H NMR (400 MHz, $CDCl_3$) δ: 7.40 - 7.36 (m, 1H), 7.32 - 7.27 (m, 2H), 7.26 - 7.19 (m, 1H), 7.16 - 7.11 (m, 1H), 7.09 - 7.03 (m, 2H), 6.99 (ddd, J = 8.0, 6.9, 1.1 Hz, 1H), 6.66 (s, 1H), 3.94 (brs, 0.05H, 97% labeled), 3.64 (s, 3H).

^{13}C NMR (101 MHz, $CDCl_3$) δ: 140.5, 137.3, 131.5, 130.5, 127.8, 127.3, 121.8, 119.7, 119.2, 119.0, 113.6, 109.3, 30.3 (p, J = 20.8 Hz).

HRMS (ESI) was calculated for $[C_{16}H_{12}D_2BrN+H]^+$ $[M+H]^+$: m/z 302.0507, found = 302.0505.

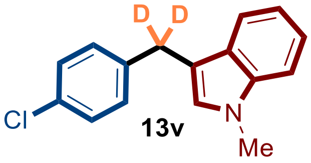

3-((4-chlorophenyl)methyl-d2)-1-methyl-1H-indole (13v):

Prepared according to general Procedure-C using phosphonium salt (**11aa**) (0.2 mmol, 133 mg) product (**13v**) was isolated in (30 mg, 60% yield) as a yellow solid. 97% Deuterium-incorporation. R_f = 0.54 (5% EtOAc in hexane).

^{1}H NMR (400 MHz, $CDCl_3$) δ: 7.48 (dt, J = 7.9, 1.0 Hz, 1H), 7.31 (dt, J = 8.2, 0.9 Hz, 1H), 7.28 - 7.20 (m, 5H), 7.09 (ddd, J = 8.0, 6.9, 1.1 Hz, 1H), 6.77 (s, 1H), 4.04 (brs, 0.05H, 97% labeled) 3.75 (s, 3H).

^{13}C NMR (101 MHz, $CDCl_3$) δ: 139.9, 137.3, 131.7, 130.1, 128.6, 128.5, 127.8, 127.3, 121.8, 119.2, 119.0, 113.7, 109.3, 32.7.

HRMS (ESI) was calculated for $[C_{16}H_{12}D_2ClN+H]^+$ $[M+H]^+$: m/z 258.1013, found = 258.1005.

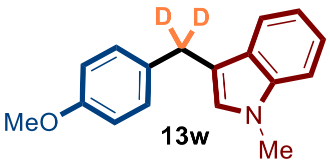

13w

3-((4-methoxyphenyl)methyl-d_2)-1-methyl-1H-indole (13w):

Prepared according to general Procedure-C using phosphonium salt (**11ai**) (0.16 mmol, 112 mg) product (**13w**) was isolated in (22 mg, 45% yield) as a colorless oil. 96% Deuterium-incorporation.

R_f = 0.35 (5% EtOAc in hexane).

^{1}H NMR (400 MHz, CD_3CN) δ: 7.46 (tt, J = 7.9, 1.0 Hz, 1H), 7.44 - 7.30 (m, 1H), 7.29 - 7.28 (m, 2H), 7.20 (dtd, J = 8.2, 7.0, 1.2 Hz, 1H), 7.01 (dtd, J = 8.0, 7.0, 1.0 Hz, 1H), 6.94 (s, 1H), 6.84 (t, J = 2.2 Hz, 1H), 6.81 (t, J = 2.1 Hz, 1H), 3.98 (brs, 0.08H, 96% labeled), 3.73 (s, 3H), 3.72 (s, 3H).

^{13}C NMR (101 MHz, CD_3CN) δ: 158.9, 138.3, 134.9, 130.4, 128.6, 128.2, 122.3, 119.9, 119.4, 118.3, 115.3, 114.6, 110.3, 55.8, 32.9, 30.6 - 30.2 (m).

HRMS (ESI) was calculated for $[C_{17}H_{15}D_2NO + H]^+$ $[M + H]^+$: m/z 254.1504, found = 254.1505.

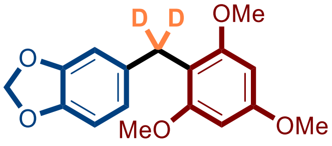

13x

5-((2,4,6-trimethoxyphenyl)methyl-d2)benzo[d][1,3]dioxole (13x):

Prepared according to general Procedure-C using phosphonium salt (**11f**) (0.2 mmol, 142 mg) product (**13x**) was isolated in (53 mg, 87% yield) as a colorless solid. 99% Deuterium-incorporation.

R_f = 0.35 (5% EtOAc in hexane).

^{1}H NMR (400 MHz, $CDCl_3$) δ: 6.78 (d, J = 1.5 Hz, 1H), 6.74 (dd, J = 7.9, 1.7 Hz, 1H), 6.70 - 6.65 (m, 1H), 6.15 (s, 2H), 5.89 - 5.85 (m, 2H), 3.81 (d, J = 2.7 Hz, 3H), 3.81 (s, 6H).

^{13}C NMR (101 MHz, $CDCl_3$) δ: 159.8, 158.8, 147.3, 145.2, 136.9, 121.2, 110.5, 109.3, 107.9, 100.6, 90.7, 55.8, 55.4, 27.4 (p, J = 18.8 Hz).

^{2}H NMR (77 MHz, $CDCl_3$) δ: 3.77.

HRMS (ESI) was calculated for $[C_{17}H_{16}D_2O_5+H]^+$ $[M+H]^+$: m/z 305.1352, found = 305.1337.

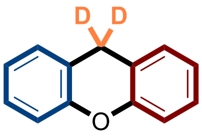

13y

9H-xanthene-9,9-d₂ (13y):

Prepared according to general Procedure-C using phosphonium salt (**11d**) (0.2 mmol, 125 mg) product (**13y**) was isolated in (30 mg, 83% yield) as a colorless solid. 96% Deuterium-incorporation.

R_f = 0.45 (5% EtOAc in hexane).

^{1}H NMR (500 MHz, $CDCl_3$) δ 7.24 - 7.14 (m, 4H), 7.07 - 7.02 (m, 4H), 4.04 (brs, 0.08, 96%).

^{13}C NMR (126 MHz, $CDCl_3$) δ 152.1, 129.0, 127.8, 123.1, 120.6, 116.6, 27.3 (p, *J* = 20.0 Hz).

HRMS (ESI) was calculated for $[C_{13}H_8D_2O+H]^+$ $[M+H]^+$: m/z 185.0929, found = 185.0941.

2.6. Sequential alkylation deuterolysis and hydrolysis for the synthesis of compound (12ac):

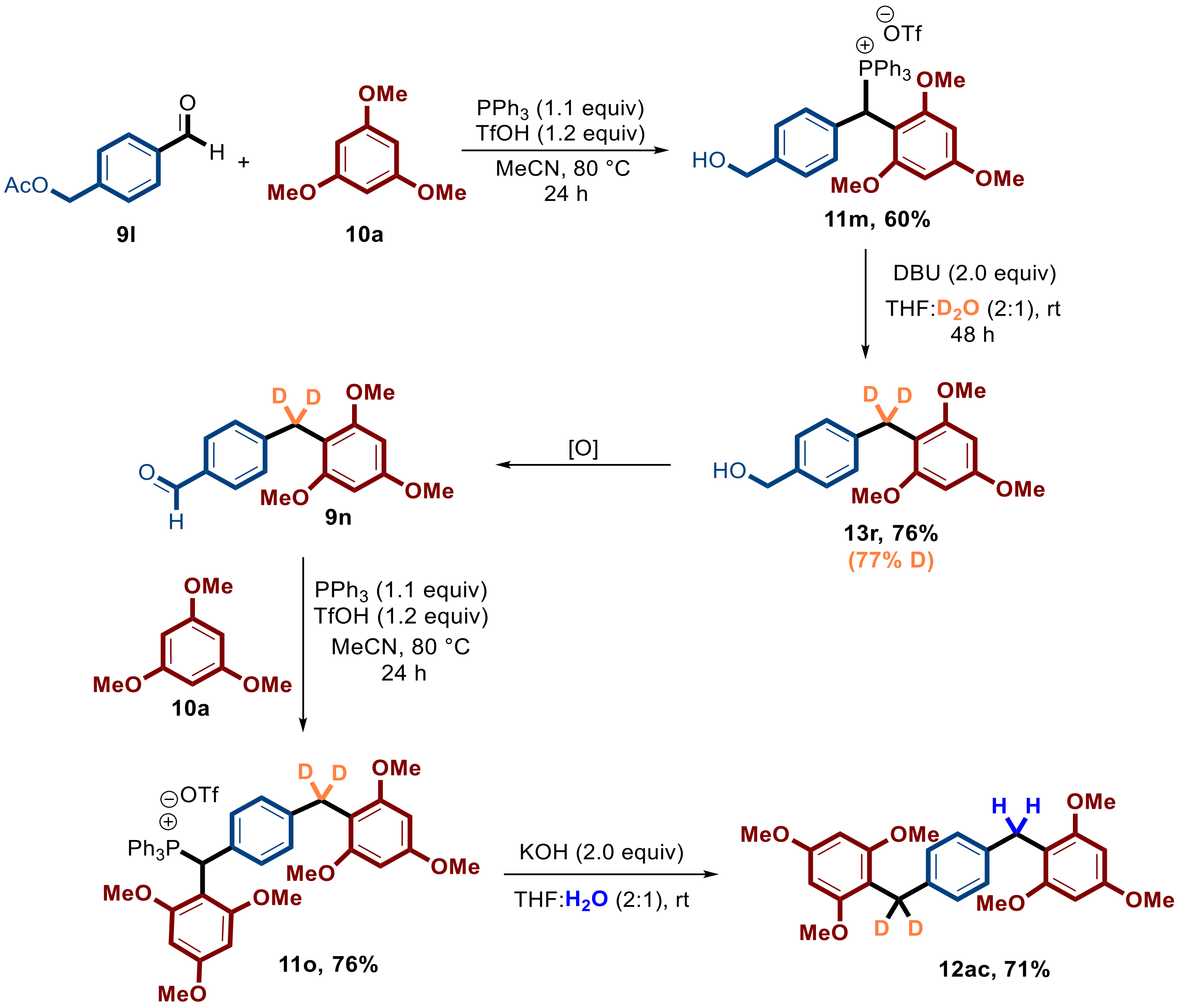

Discussion:

A sequential and multiple functionalization was applied. First and alkylation for aldehyde **9i** with **10a** that yielded **11m** with a 60% yield according to general Procedure-A. Then, this **11m** phosphonium salt was subjected into the first deuterolysis to give product **13r** in 76% yield and 77% D incorporation using general Procedure-C, and then was oxidize to yield the D-incorporated aldehyde **9n**. Another alkylation reaction was performed for **9n** aldehyde along with **10a** in the same manner as general Procedure-A to give the phosphonium salt **11o** in 76%. The last step was converting **11o** using general Procedure-B to yield the hydrolysis product **12ac** in 71% yield.

2.7. General Procedure-D and Characterization for Diarylketones Products (14)

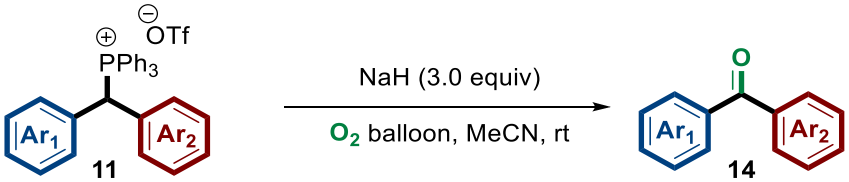

Inside a 10 mL vial, phosphonium salt **11** (0.2 mmol, 1 equiv) was added under an oxygen atmosphere (O_2 balloon) and dissolved in 3 mL of CH_3CN (previously degassed with an O_2 balloon for 10 minutes). Then, sodium hydride (NaH) (3 equiv, 15 mg) was added in one portion, and the reaction mixture was stirred for 5 hours. Finally, the reaction was quenched by the addition of EtOAc, and the solvents were evaporated under reduced pressure (Evaporator), yielding the crude compound. The crude product was further purified by short column chromatography to afford the desired product (**14**).

Table - S4: Scope of the for diarylketones Products (**14**):

Entry	Phosphonium Salts (11)	Diarylketone (14)	Yield[a]
1.	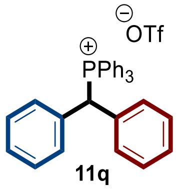 11q	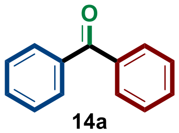 14a	92%
2.	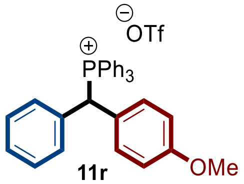 11r	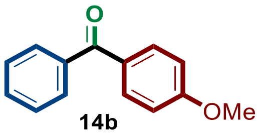 14b	80%
3.	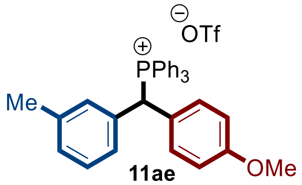 11ae	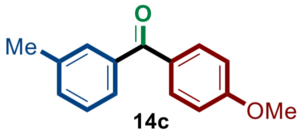 14c	90%

4.	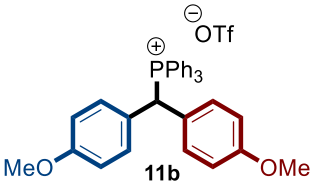 **11b**	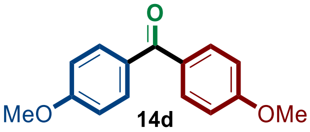 **14d**	84%
5.	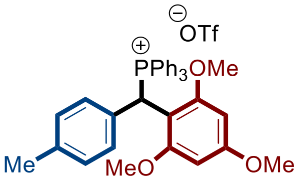 **11ad**	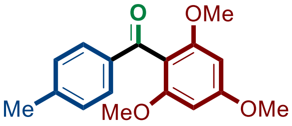 **14e**	55%
6.	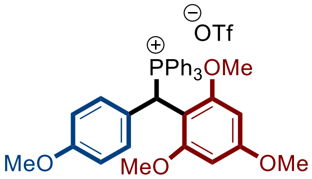 **11t**	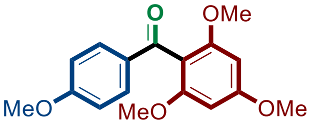 **14f**	64%
7.	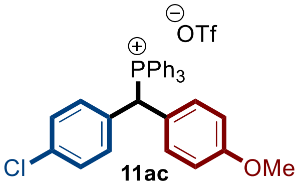 **11ac**	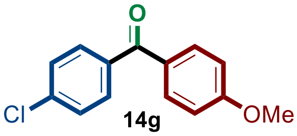 **14g**	59%
8.	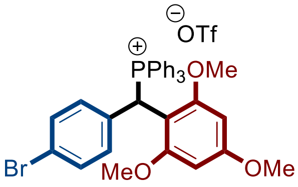 **11w**	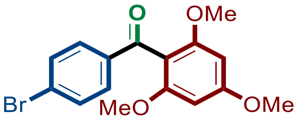 **14h**	54%
9.	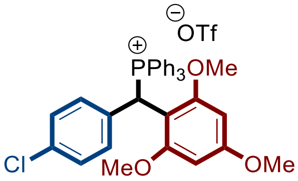 **11z**	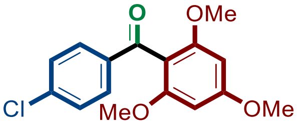 **14i**	82%
10.	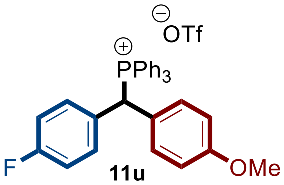 **11u**	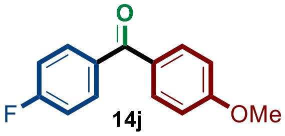 **14j**	83%

11.	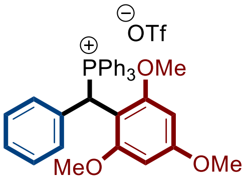 11x	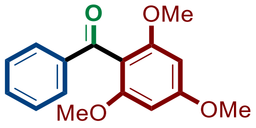 14k	50%
12.	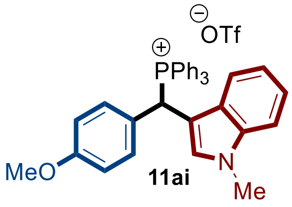 11ai	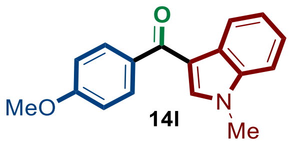 14l	70%
13.	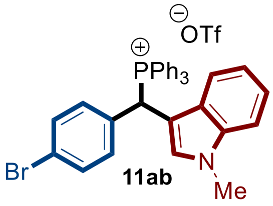 11ab	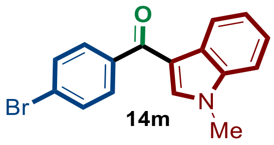 14m	58%
14.	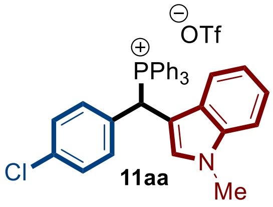 11aa	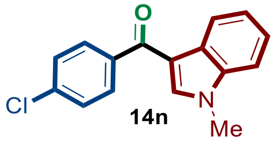 14n	55%
15.	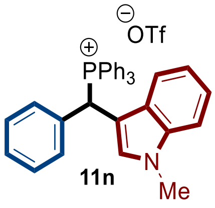 11n	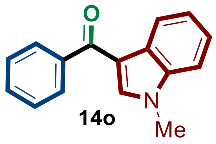 14o	71%
16.	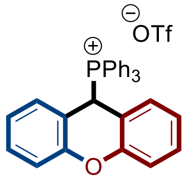 11d	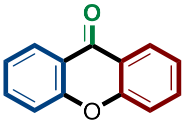 14p	95%

[a] Isolated yield.

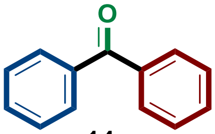

14a

benzophenone (14a):

Prepared according to general Procedure-D using phosphine salt (**11q**) (0.2 mmol, 115 mg) product (**14a**) was isolated in (34 mg, 92% yield) as a colorless solid.

R_f = 0.6 (10% EtOAc in hexane).

^{1}H NMR (400 MHz, $CDCl_3$) δ: 7.81 (d, J = 6.9 Hz, 4H), 7.63 - 7.54 (m, 2H), 7.52 - 7.44 (m, 4H).

The spectral data are consistent with those reported in the literature.[24]

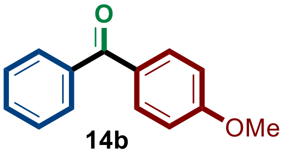

14b

(4-methoxyphenyl)(phenyl)methanone (14b):

Prepared according to general Procedure-D using phosphonium salt (**11r**) (0.2 mmol, 121 mg) product (**14b**) was isolated in (34 mg, 80% yield) as a colorless solid.

R_f = 0.55 (10% EtOAc in hexane).

^{1}H NMR (400 MHz, $CDCl_3$) δ: 7.86 - 7.79 (m, 2H), 7.78 - 7.72 (m, 2H), 7.60 - 7.52 (m, 1H), 7.50 - 7.40 (m, 2H), 6.99 - 6.94 (m, 2H), 3.88 (s, 3H).

The spectral data are consistent with those reported in the literature.[24]

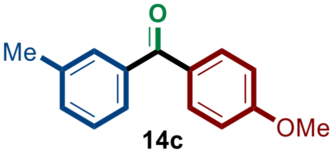

14c

(4-methoxyphenyl)(m-tolyl)methanone (14c):

Prepared according to general Procedure-D using phosphonium salt (**11ae**) (0.2 mmol, 124 mg) product (**14c**) was isolated in (41 mg, 90% yield) as a colorless solid.

R_f = 0.51 (10% EtOAc in hexane).

^{1}H NMR (400 MHz, $CDCl_3$) δ: 7.83 (d, J = 8.4 Hz, 2H), 7.59 - 7.49 (m, 2H), 7.41 - 7.30 (m, 2H), 6.96 (d, J = 8.4 Hz, 2H), 3.89 (s, 3H), 2.42 (s, 3H).

The spectral data are consistent with those reported in the literature.[25]

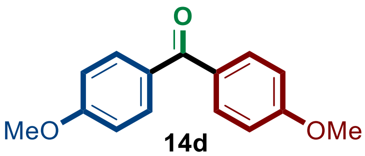

14d

bis(4-methoxyphenyl)methanone (14d):

Prepared according to general Procedure-D using phosphonium salt (**11b**) (0.2 mmol, 127 mg) product (**14d**) was isolated in (41 mg, 84% yield) as a colorless solid.

R_f = 0.49 (10% EtOAc in hexane).

^{1}H NMR (400 MHz, $CDCl_3$) δ: 7.82 - 7.75 (m, 4H), 6.97 - 6.93 (m, 4H), 3.89 (s, 6H).

The spectral data are consistent with those reported in the literature.[26]

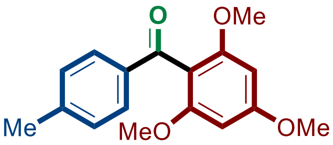

14e

p-tolyl(2,4,6-trimethoxyphenyl)methanone (14e):

Prepared according to general Procedure-D using phosphonium salt (**11ad**) (0.2 mmol, 136 mg) product (**14e**) was isolated in (32 mg, 55% yield) as a colorless solid.

R_f = 0.35 (20% EtOAc in hexane).

^{1}H NMR (400 MHz, $CDCl_3$) δ: 7.74 (d, J = 8.2 Hz, 2H), 7.21 (d, J = 8.6 Hz, 2H), 6.17 (s, 2H), 3.86 (s, 3H), 3.68 (s, 6H), 2.40 (s, 3H).

The spectral data are consistent with those reported in the literature.[27]

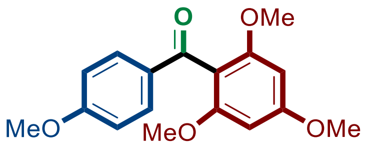

14f

(4-methoxyphenyl)(2,4,6-trimethoxyphenyl)methanone (14f):

Prepared according to general Procedure-D using phosphonium salt (**11t**) (0.2 mmol, 139 mg) product (**14f**) was isolated in (39 mg, 64% yield) as a colorless solid.

R_f = 0.34 (30% EtOAc in hexane).

^{1}H NMR (400 MHz, $CDCl_3$) δ: 7.82 (d, J = 9.0 Hz, 2H), 6.89 (d, J = 9.0 Hz, 2H), 6.17 (s, 2H), 3.86 (s, 3H), 3.85 (s, 3H), 3.69 (s, 6H).

The spectral data are consistent with those reported in the literature.[28]

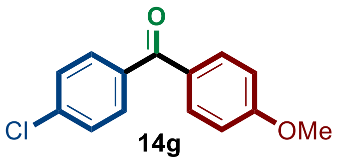

14g

(4-chlorophenyl)(4-methoxyphenyl)methanone (14g):

Prepared according to general Procedure-D using phosphonium salt (**11ac**) (0.2 mmol, 128 mg) product (**14g**) was isolated in (30 mg, 59% yield) as a colorless solid.

R_f = 0.56 (15% EtOAc in hexane).

^{1}H NMR (400 MHz, $CDCl_3$) δ: 7.83 - 7.76 (m, 2H), 7.74 - 7.67 (m, 2H), 7.49 - 7.42 (m, 2H), 7.01 - 6.94 (m, 2H), 3.89 (s, 3H).

The spectral data are consistent with those reported in the literature.[26]

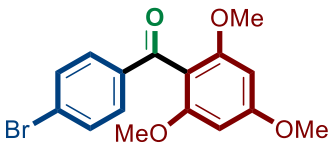

14h

(4-bromophenyl)(2,4,6-trimethoxyphenyl)methanone (14h):

Prepared according to general Procedure-D using phosphonium salt (**11w**) (0.2 mmol, 149 mg) product (**14h**) was isolated in (38 mg, 54% yield) as a yellow solid.

$R_f = 0.32$ (20% EtOAc in hexane).

^{1}H NMR (400 MHz, $CDCl_3$) δ: 7.73 - 7.66 (m, 2H), 7.58 - 7.51 (m, 2H), 6.16 (s, 2H), 3.86 (s, 3H), 3.69 (s, 6H).

The spectral data are consistent with those reported in the literature.[27]

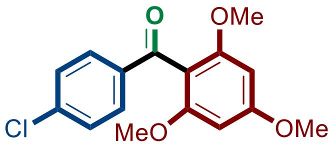

14i

(4-chlorophenyl)(2,4,6-trimethoxyphenyl)methanone (14i):

Prepared according to general Procedure-D using phosphonium salt (**11z**) (0.2 mmol, 140 mg) product (**14i**) was isolated in (50 mg, 82% yield) as a colorless solid.

$R_f = 0.27$ (15% EtOAc in hexane).

^{1}H NMR (400 MHz, $CDCl_3$) δ: 7.77 (d, J = 8.6 Hz, 2H), 7.38 (d, J = 8.4 Hz, 2H), 6.16 (s, 2H), 3.86 (s, 3H), 3.68 (s, 6H).

^{13}C NMR (126 MHz, $CDCl_3$) δ: 193.8, 162.8, 158.9, 139.4, 136.8, 130.9, 128.7, 110.5, 90.8, 55.96, 55.6.

HRMS (ESI) was calculated for $[C_{16}H_{15}ClO_4 + H]^+$ $[M + H]^+$: m/z 307.0731, found = 307.0725.

Note: The crude reaction mixture of (**14i**) was submitted to ^{31}P-NMR analysis. (see page S185)

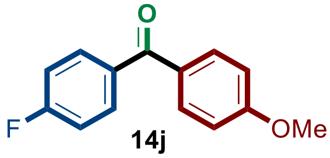

14j

(4-fluorophenyl)(4-methoxyphenyl)methanone (14j):

Prepared according to general Procedure-D using phosphonium salt (**11u**) (0.2 mmol, 125 mg) product (**14j**) was isolated in (39 mg, 83% yield) as a colorless solid.

$R_f = 0.52$ (15% EtOAc in hexane).

^{1}H NMR (400 MHz, $CDCl_3$) δ: 7.83 - 7.74 (m, 4H), 7.15 (t, J = 8.6 Hz, 2H), 6.97 (d, J = 8.9 Hz, 2H), 3.89 (s, 3H).

The spectral data are consistent with those reported in the literature.[26]

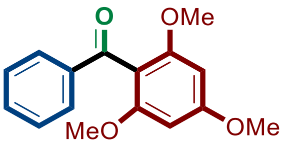

14k

phenyl(2,4,6-trimethoxyphenyl)methanone (14k):

Prepared according to general Procedure-D using phosphonium salt (**11x**) (0.2 mmol, 142 mg) product (**14k**) was isolated in (27 mg, 50% yield) as a white solid.

R_f = 0.29 (20% EtOAc in hexane).

^{1}H NMR (400 MHz, $CDCl_3$) δ: 7.86 - 7.82 (m, 2H), 7.55 - 7.50 (m, 1H), 7.44 - 7.39 (m, 2H), 6.17 (s, 2H), 3.86 (s, 3H), 3.68 (s, 6H).

^{13}C NMR (126 MHz, $CDCl_3$) δ: 195.1, 162.5, 158.9, 138.3, 133.0, 129.5, 128.4, 111.1, 90.8, 55.9, 55.6.

HRMS (ESI) was calculated for $[C_{16}H_{16}O_4+H]^+$ $[M+H]^+$: m/z 273.1121, found = 273.11145.

The spectral data are consistent with those reported in the literature.[26]

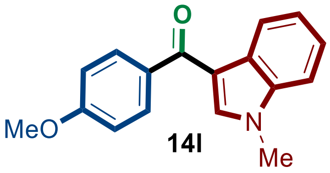

14l

(4-methoxyphenyl)(1-methyl-1H-indol-3-yl)methanone (14l):

Prepared according to general Procedure-D using phosphonium salt (**11ai**) (0.2 mmol, 132 mg) product (**14l**) was isolated in (37 mg, 70% yield) as a yellow oil.

R_f = 0.24 (20% EtOAc in hexane).

^{1}H NMR (400 MHz, $CDCl_3$) δ: 8.40 - 8.34 (m, 1H), 7.85 (d, J = 8.8 Hz, 2H), 7.56 (s, 1H), 7.41 - 7.30 (m, 3H), 6.99 (d, J = 8.8 Hz, 2H), 3.90 (s, 3H), 3.86 (s, 3H).

The spectral data are consistent with those reported in the literature.[29]

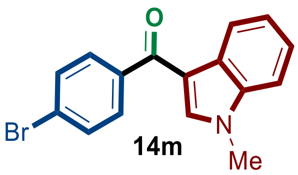

14m

(4-bromophenyl)(1-methyl-1H-indol-3-yl)methanone (14m):

Prepared according to general Procedure-D using phosphonium salt (**11ab**) (0.2 mmol, 142 mg) product (**14m**) was isolated in (37 mg, 58% yield) as a yellow solid.

R_f = 0.25 (20% EtOAc in hexane).

^{1}H NMR (400 MHz, $CDCl_3$) δ: 8.41 - 8.36 (m, 1H), 7.74 - 7.67 (m, 2H), 7.65 - 7.60 (m, 2H), 7.51 (s, 1H), 7.40 - 7.33 (m, 3H), 3.86 (s, 3H).

The spectral data are consistent with those reported in the literature. [29]

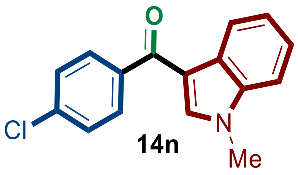

14n

(4-chlorophenyl)(1-methyl-1H-indol-3-yl)methanone (14n):

Prepared according to general Procedure-D using phosphonium salt (**11aa**) (0.2 mmol, 133 mg) product (**14n**) was isolated in (30 mg, 55% yield) as a yellow liquid.

R_f = 0.25 (20% EtOAc in hexane).

^{1}H NMR (400 MHz, $CDCl_3$) δ: 8.41 - 8.36 (m, 1H), 7.80 - 7.73 (m, 2H), 7.52 (s, 1H), 7.48 - 7.44 (m, 2H), 7.40 - 7.33 (m, 3H), 3.87 (s, 3H).

The spectral data are consistent with those reported in the literature.[29]

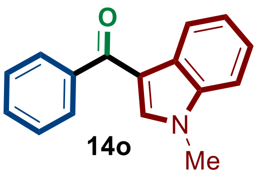

14o

(1-methyl-1H-indol-3-yl)(phenyl)methanone (14o):

Prepared according to general Procedure-D using phosphonium salt (**11n**) (0.2 mmol, 135 mg) product (**14o**) was isolated in (34 mg, 71% yield) as a yellow oil.

R_f = 0.29 (20% EtOAc in hexane).

^{1}H NMR (400 MHz, $CDCl_3$) δ: 8.46 - 8.40 (m, 1H), 7.85 - 7.79 (m, 2H), 7.58 - 7.45 (m, 4H), 7.41 - 7.32 (m, 3H), 3.85 (s, 3H).

^{13}C NMR (126 MHz, $CDCl_3$) δ: 190.9, 141.1, 138.0, 137.7, 131.2, 128.8, 128.4, 127.3, 123.8, 122.9, 122.8, 115.8, 109.7, 33.7.

HRMS (ESI) was calculated for $[C_{16}H_{13}NO+H]^+$ $[M+H]^+$: m/z 236.1069, found = 236.1064.

The spectral data are consistent with those reported in the literature.[29]

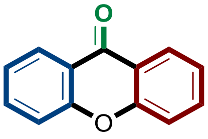

14p

9H-xanthen-9-one (14p):

Prepared according to general Procedure-D using phosphonium salt (**11d**) (0.2 mmol, 88 mg) product (**14p**) was isolated in (37 mg, 95% yield) as a yellowish solid.

R_f = 0.48 (10% EtOAc in hexane).

^{1}H NMR (400 MHz, $CDCl_3$) δ 8.35 (ddd, J = 8.0, 1.8, 0.5 Hz, 2H), 7.74 (ddd, J = 8.5, 7.1, 1.7 Hz, 2H), 7.51 (ddd, J = 8.5, 1.1, 0.5 Hz, 2H), 7.43 - 7.35 (m, 2H).

The spectral data are consistent with those reported in the literature. [30]

Progress for the oxidation reaction of benzhydryl phosphonium salt 11z to produce 14i:

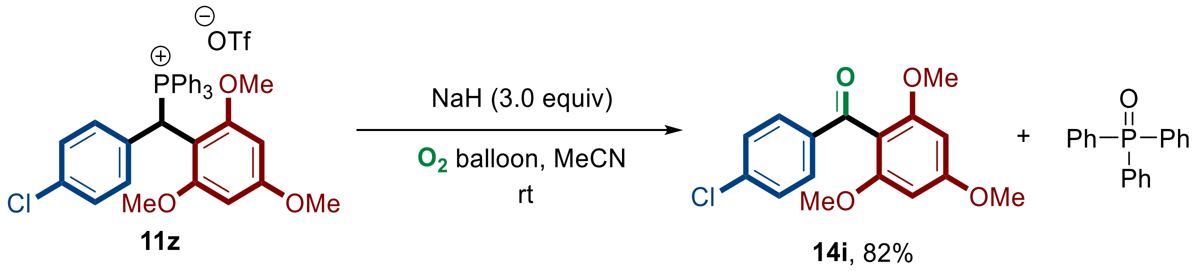

Inside a 10 mL vial, phosphonium salt **11z** (0.2 mmol, 1 equiv) was added under an oxygen atmosphere (O_2 balloon) and dissolved in 3 mL of CH_3CN (previously degassed with an O_2 balloon for 10 minutes). Then, sodium hydride (NaH) (3 equiv, 15 mg) was added in one portion, and the reaction mixture was stirred for 5 hours. Finally, the reaction was quenched by the addition of EtOAc, and the solvents were evaporated under reduced pressure (evaporator), and the crude mixture was analyzed by NMR.

Observation: Crude P-NMR analysis showed the disappearance of 23.9 ppm peak which is associated with starting phosphonium salt **11z**. And the appearance of 29.9 ppm peak which is associated with triphenyl phosphonium oxide ($Ph_3P{=}O$)

^{31}P NMR spectrum (162 MHz, $CDCl_3$) of the crude reaction mixture of **14i**:

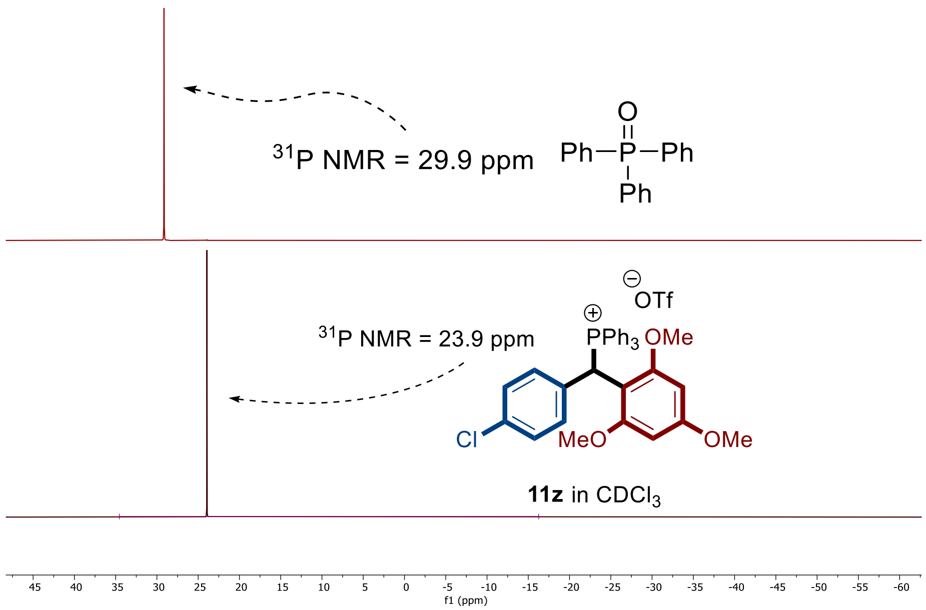

2.8. Control experiment for the oxidation reaction:

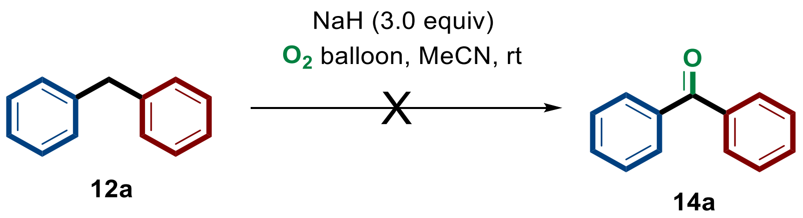

Inside a 10 mL vial, diphenylmethane **12a** (0.2 mmol, 1 equiv) was added under an oxygen atmosphere (O_2 balloon) and dissolved in 3 mL of CH_3CN (previously degassed with an O_2 balloon for 10 minutes). Then, sodium hydride (NaH) (3 equiv, 15 mg) was added in one portion, and the reaction mixture was stirred for 5 hours. Finally, the reaction was quenched by the addition of EtOAc, and the solvents were evaporated under reduced pressure (Evaporator), yielding the crude compound, that was analyzed using ^{1}H-NMR.

Result and Discussion:

To determine whether product **14** is obtained through a direct oxidation of phosphonium salt **11** or whether it undergoes a reduction step to form product **12**, which is then oxidized to yield product **14**, we subjected product **12a** to the same oxidation conditions: 3 equivalents of NaH, an oxygen balloon, and MeCN as the solvent at room temperature for 5 hours. The result, showing no conversion of **12a** into **14a**, suggests that the direct oxidation of **11** with molecular oxygen is the most likely mechanism taking place.

2. NMR Spectra

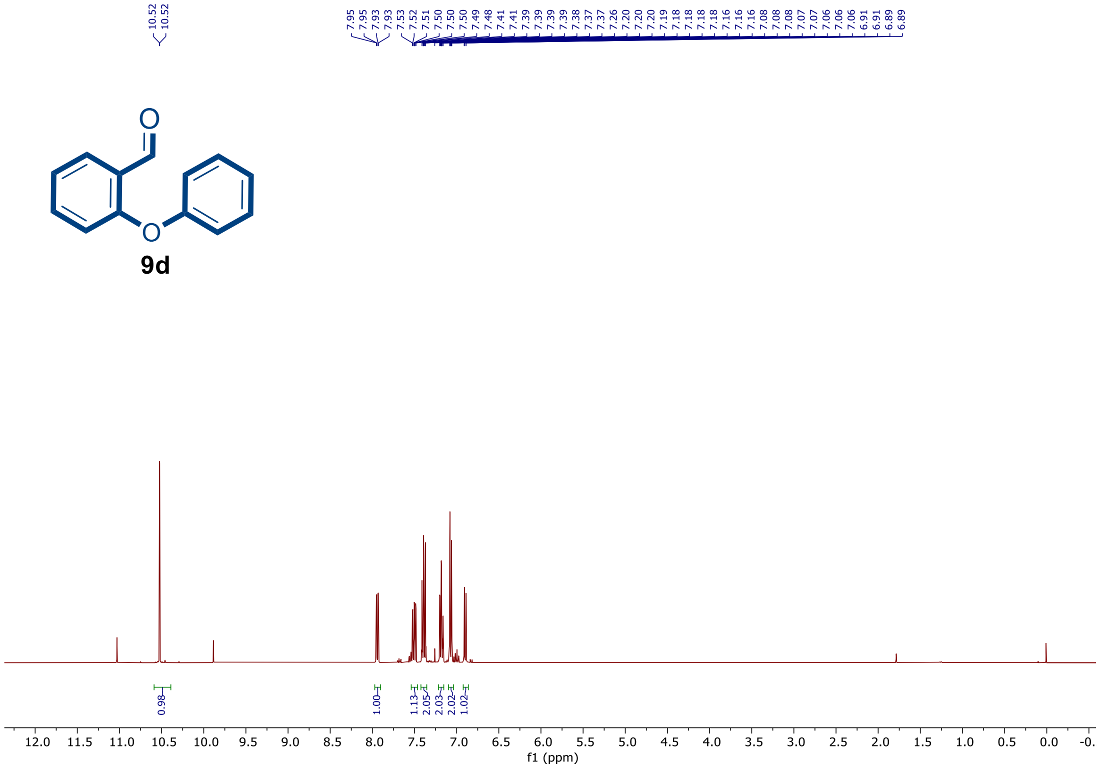

^{1}H NMR (400 MHz, $CDCl_3$) of compound (**9d**)

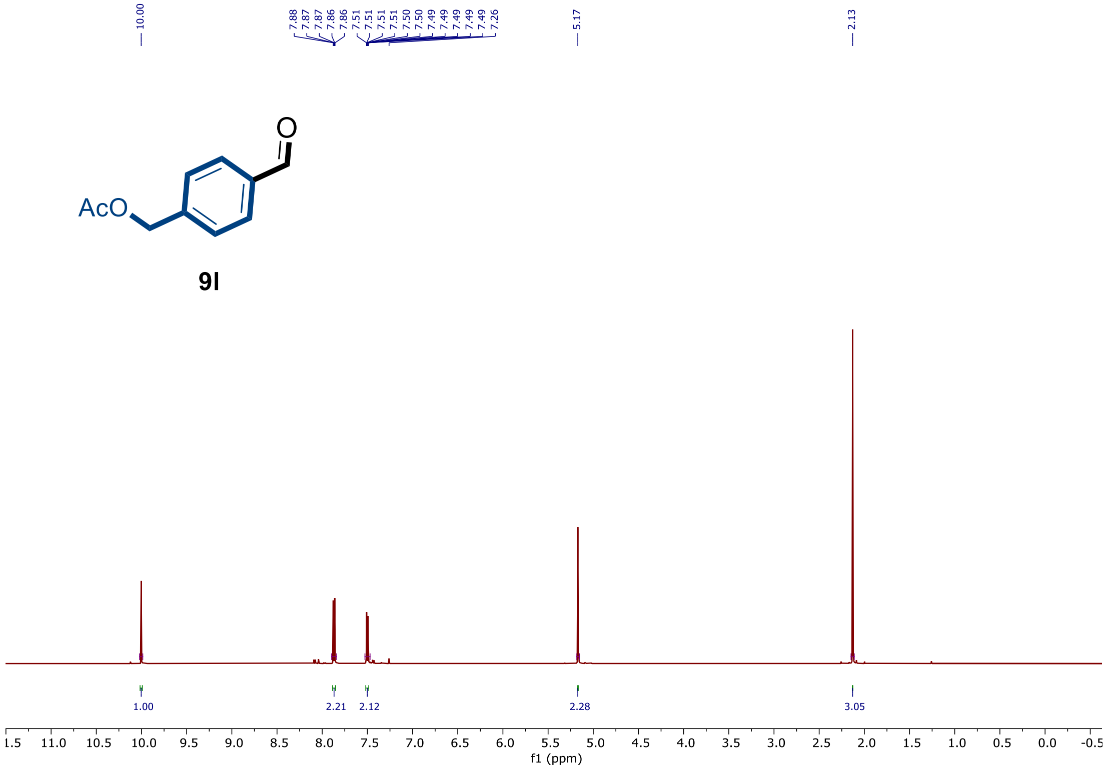

^{1}H NMR (400 MHz, $CDCl_3$) of compound (**9l**)

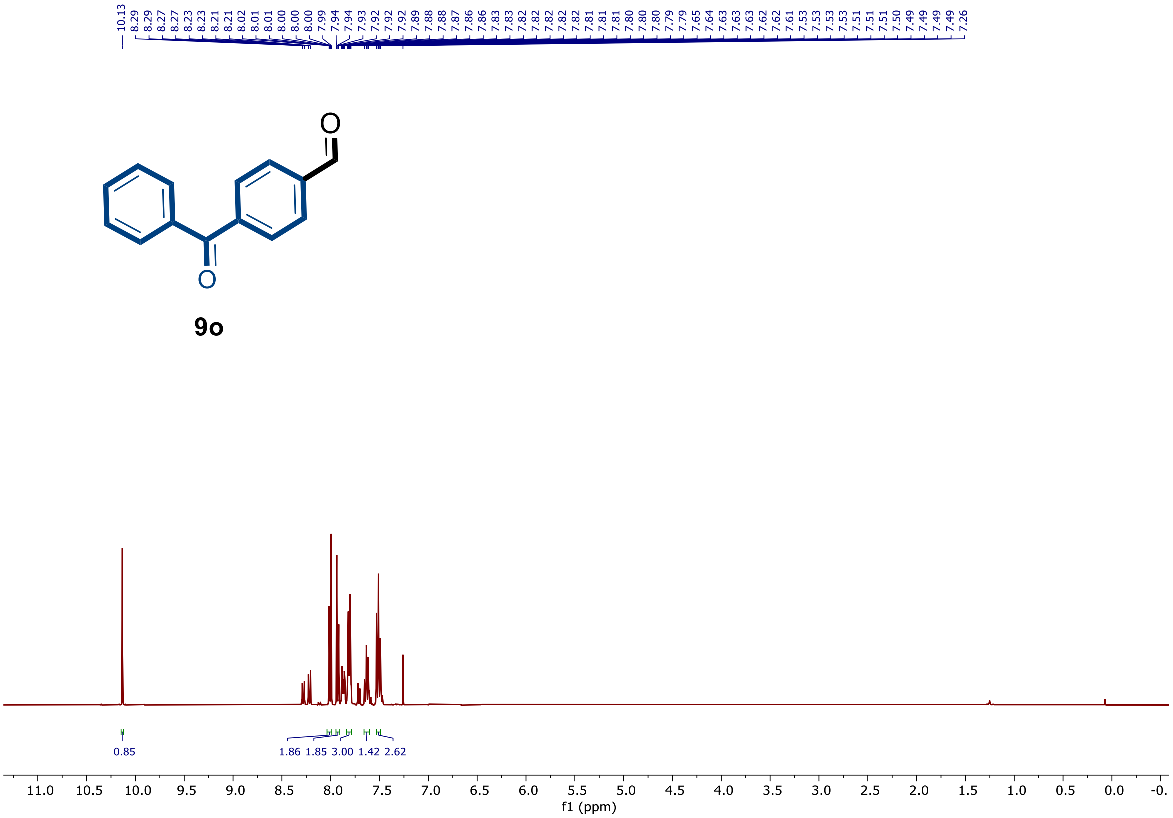

^{1}H NMR (400 MHz, $CDCl_3$) of compound (**9o**)

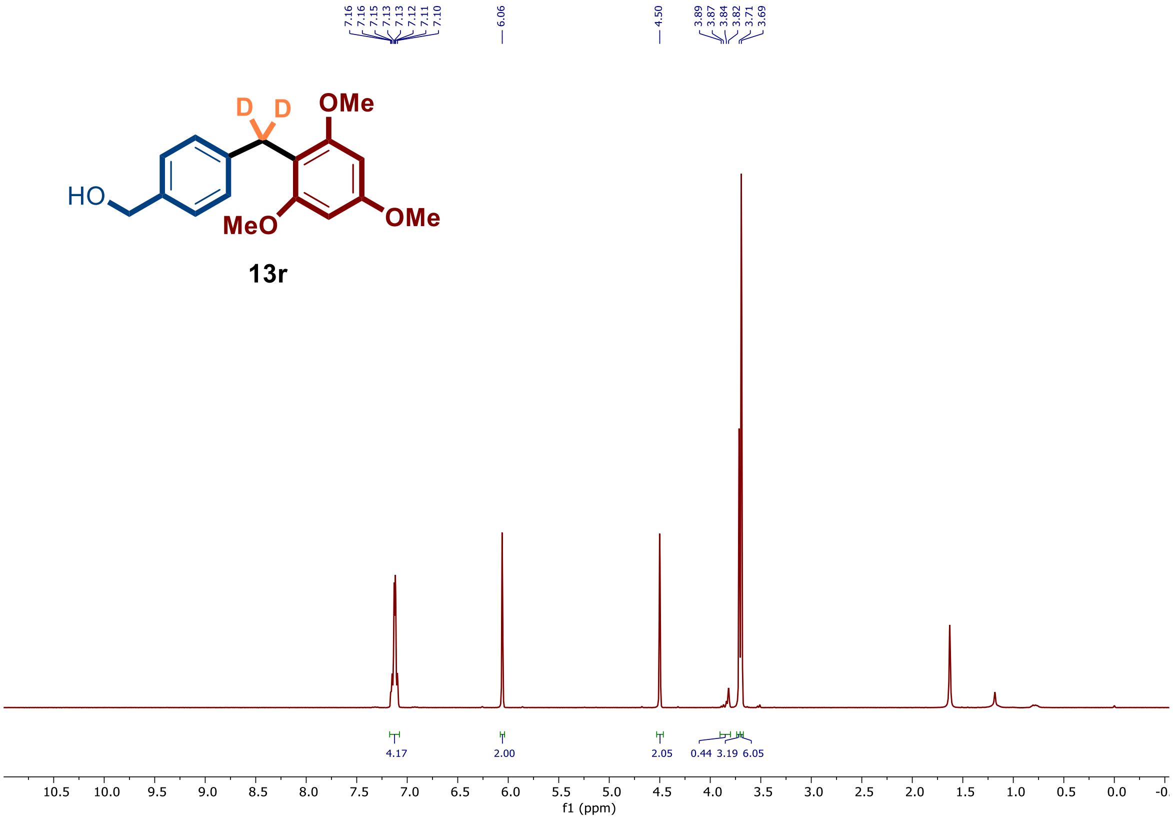

^{1}H NMR (400 MHz, $CDCl_3$) of compound (**13r**)

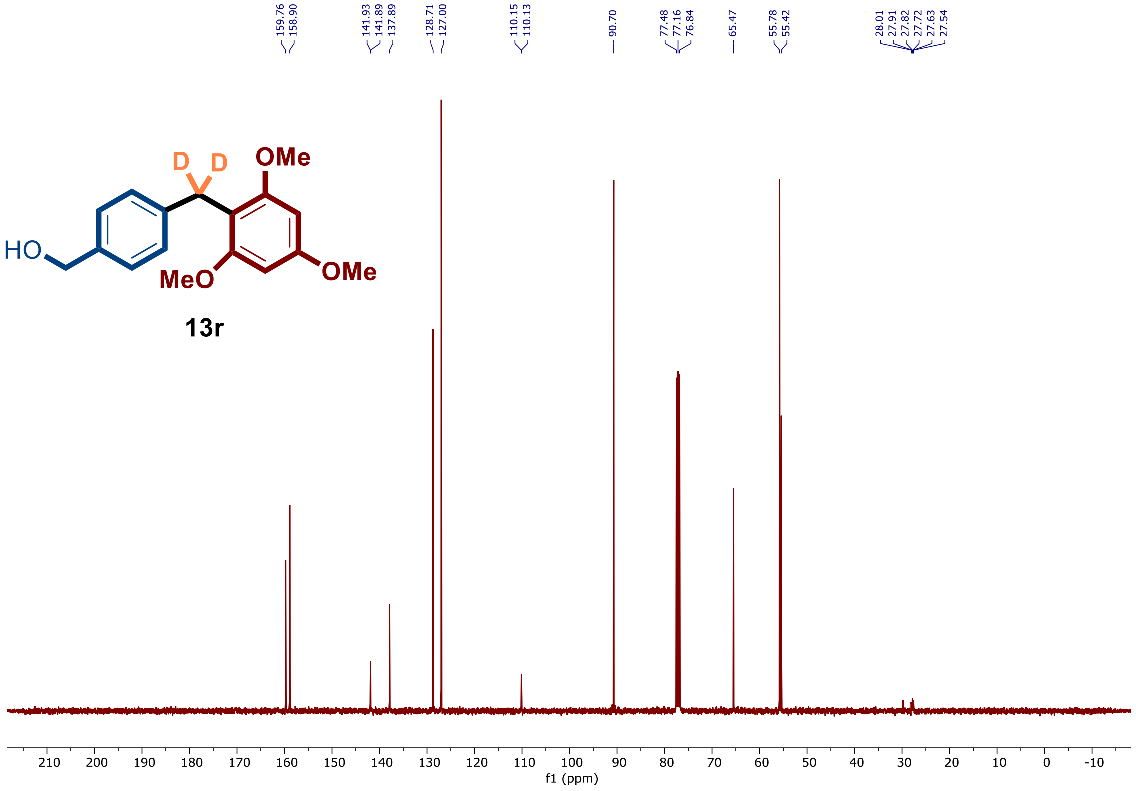

^{13}C NMR (101 MHz, $CDCl_3$) of compound (**13r**)

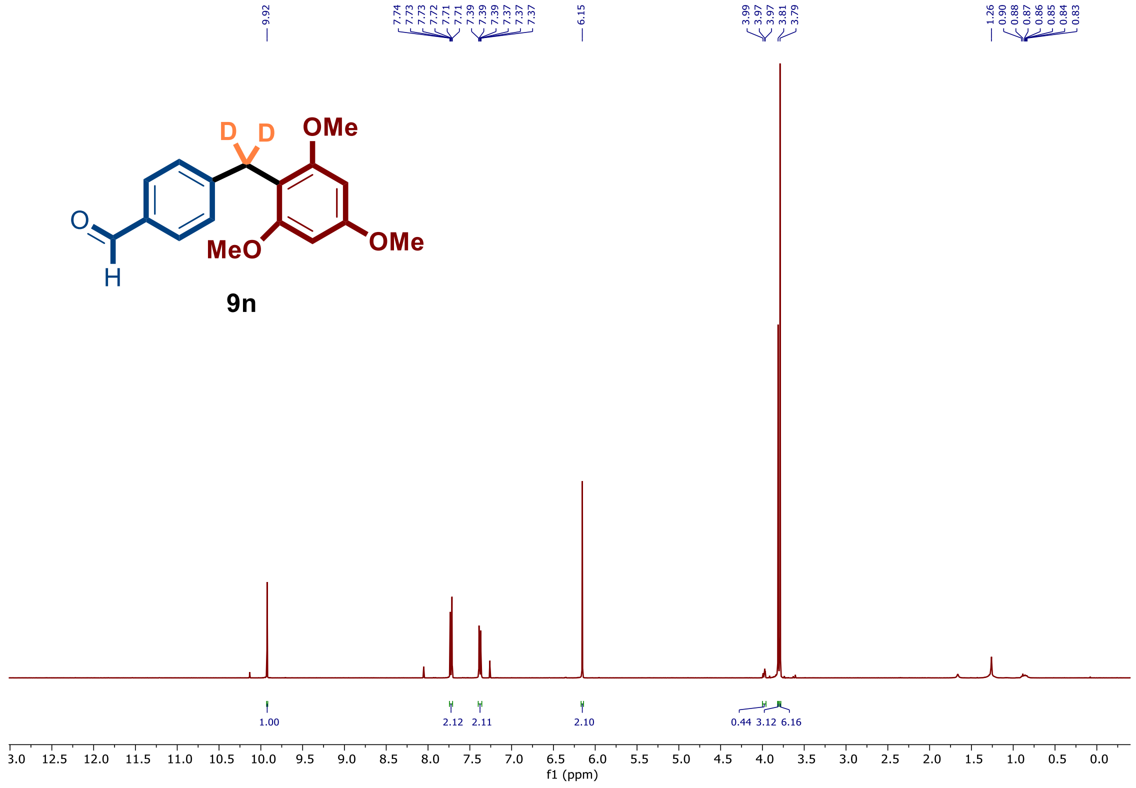

^{1}H NMR (400 MHz, $CDCl_3$) of compound (**9n**)

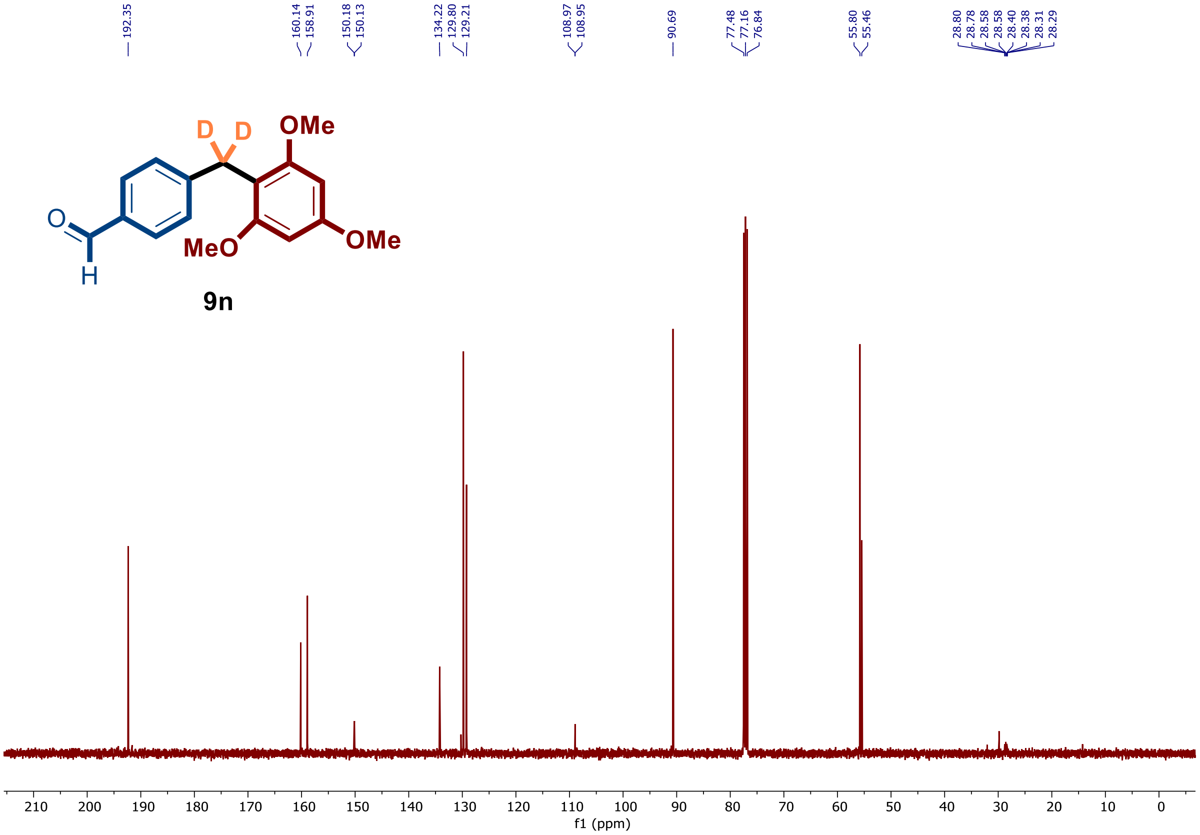

^{13}C NMR (101 MHz, $CDCl_3$) of compound (**9n**)

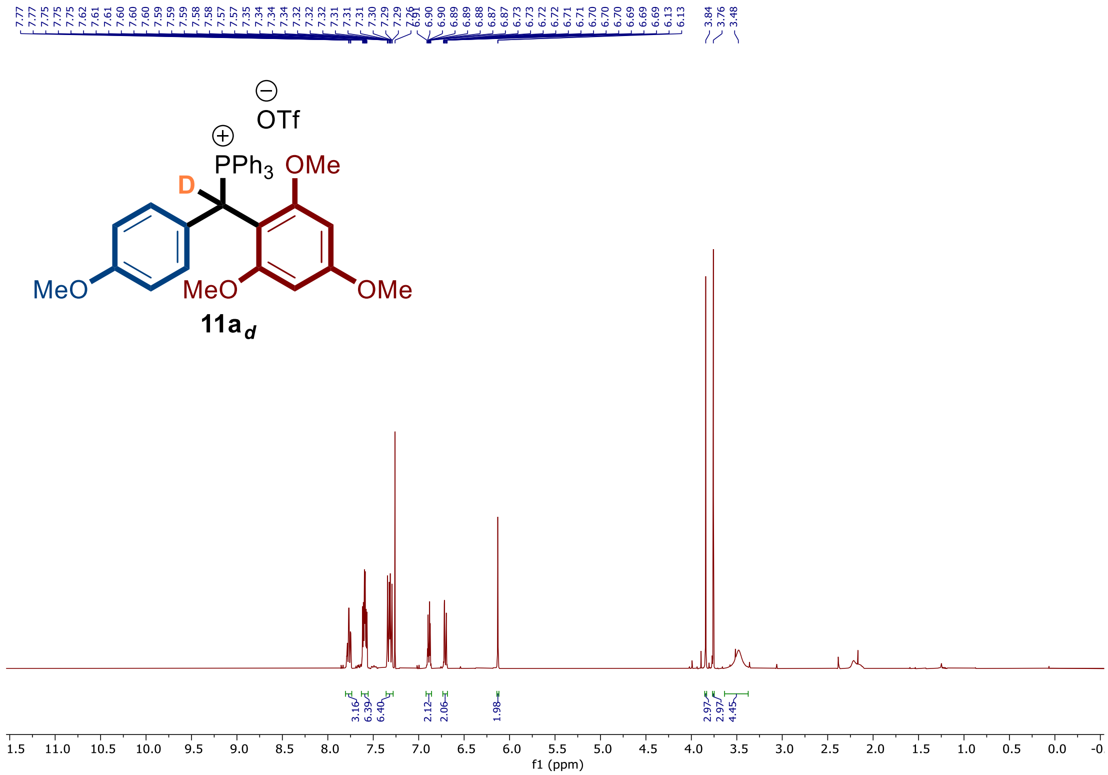

^{1}H NMR (400 MHz, $CDCl_3$) of compound (**11a*d***)

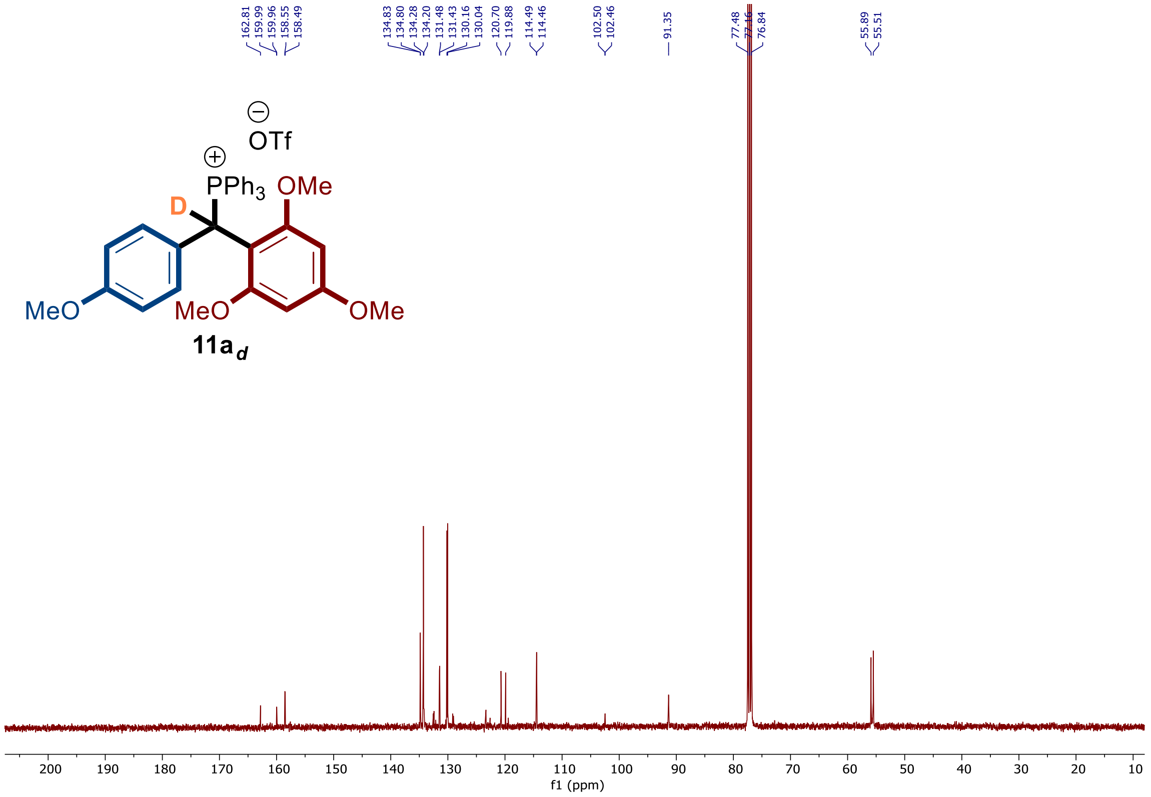

^{13}C NMR (101 MHz, $CDCl_3$) of compound (**11a*d***)

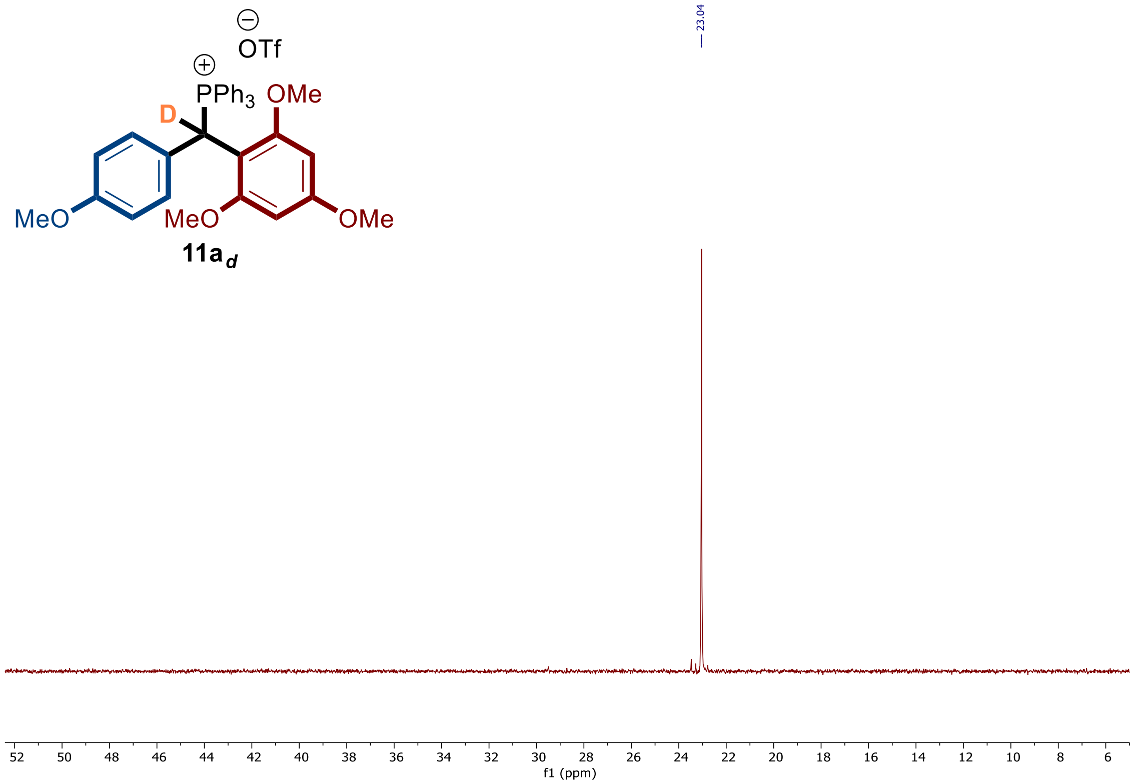

^{31}P NMR (162 MHz, $CDCl_3$) of compound (**11a*d***)

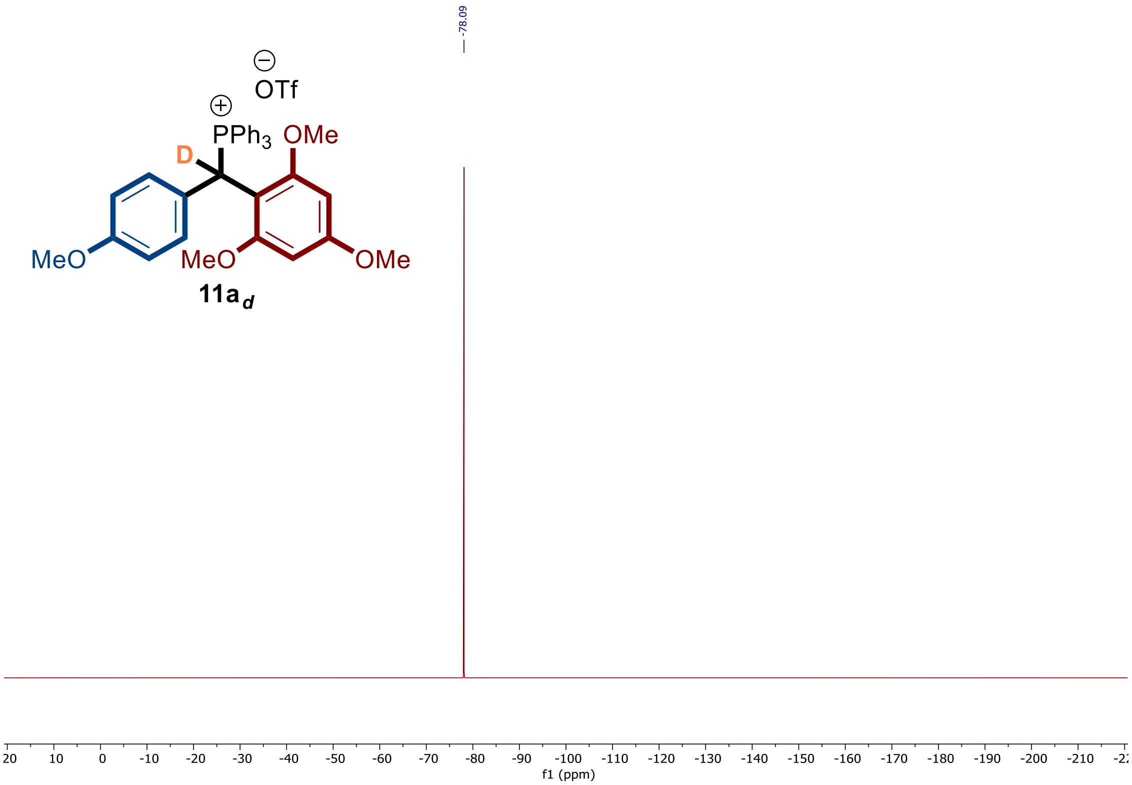

^{19}F NMR (376 MHz, $CDCl_3$) of compound (**11a$_d$**)

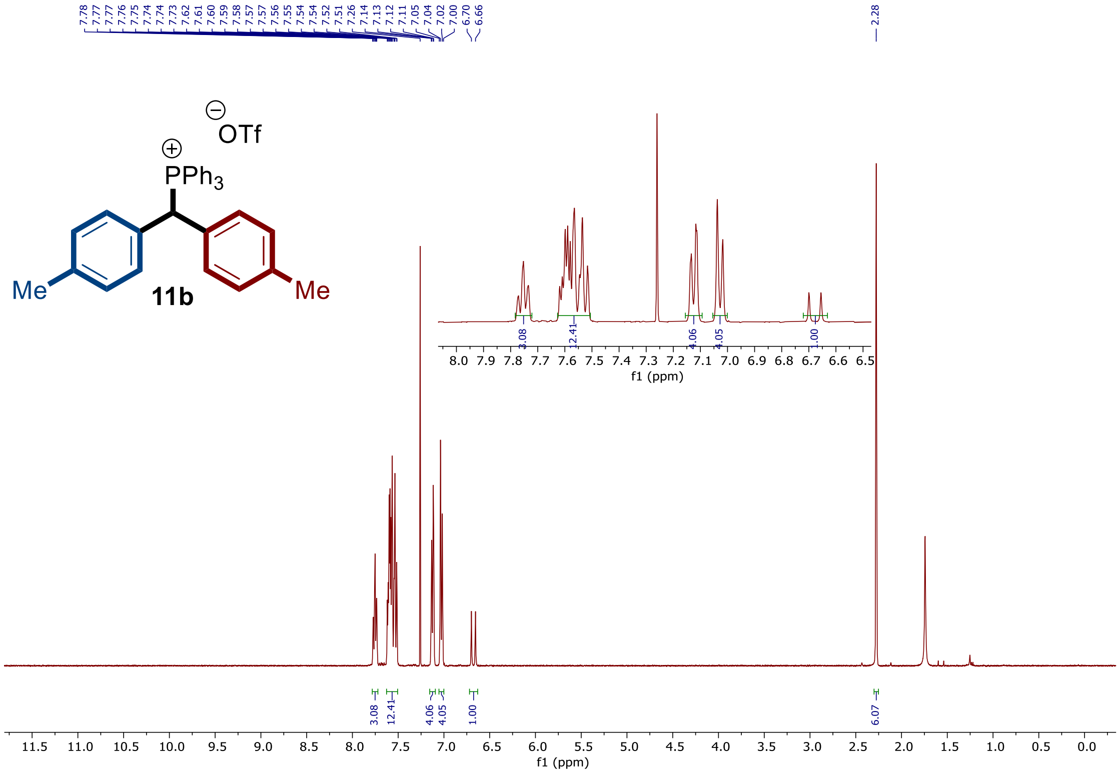

^{1}H NMR (400 MHz, $CDCl_3$) of compound (**11b**)

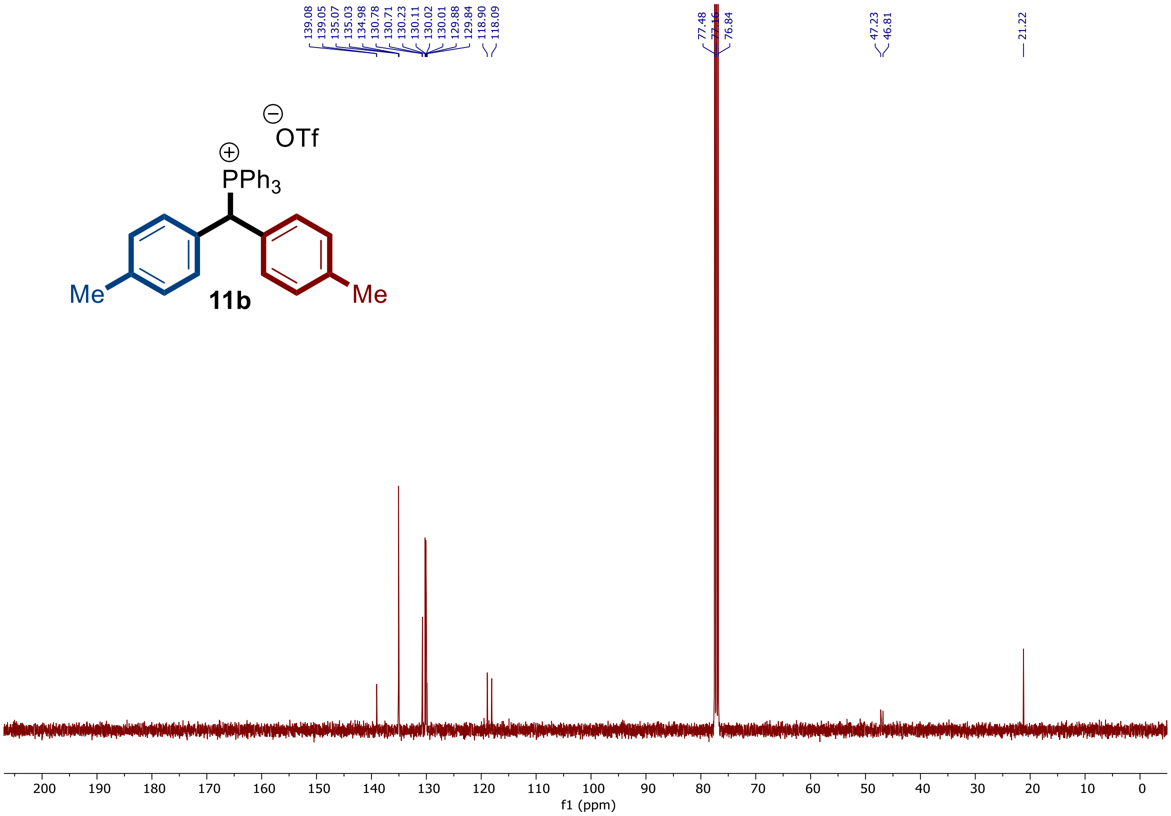

^{13}C NMR (101 MHz, $CDCl_3$) of compound (**11b**)

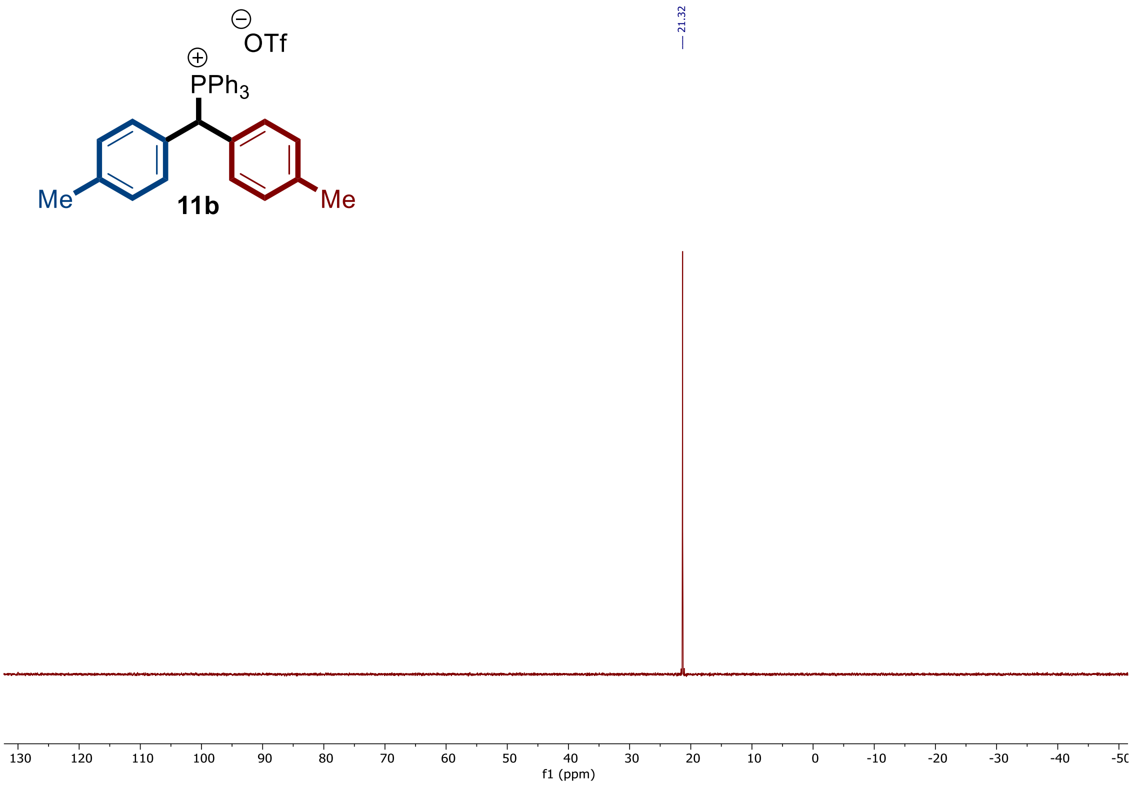

^{31}P NMR (162 MHz, $CDCl_3$) of compound (**11b**)

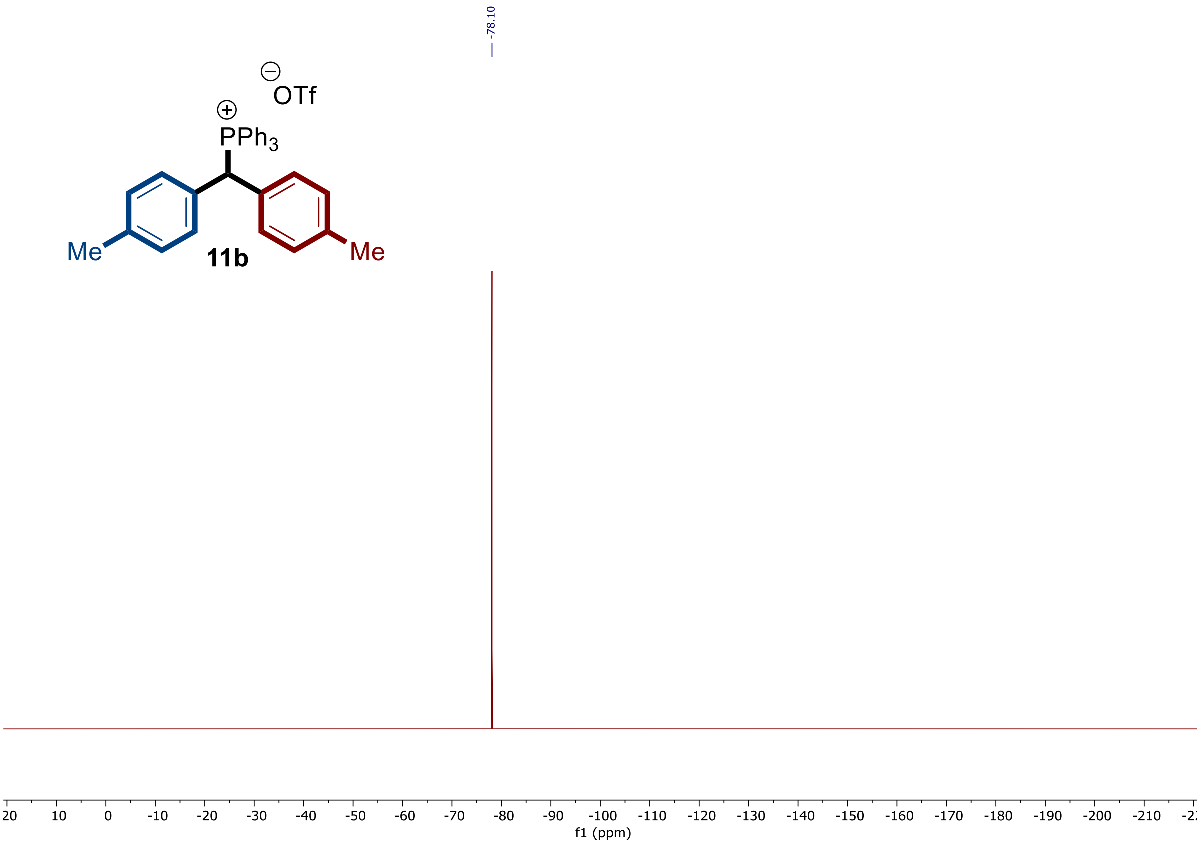

^{19}F NMR (376 MHz, CDCl$_3$) of compound (**11b**)

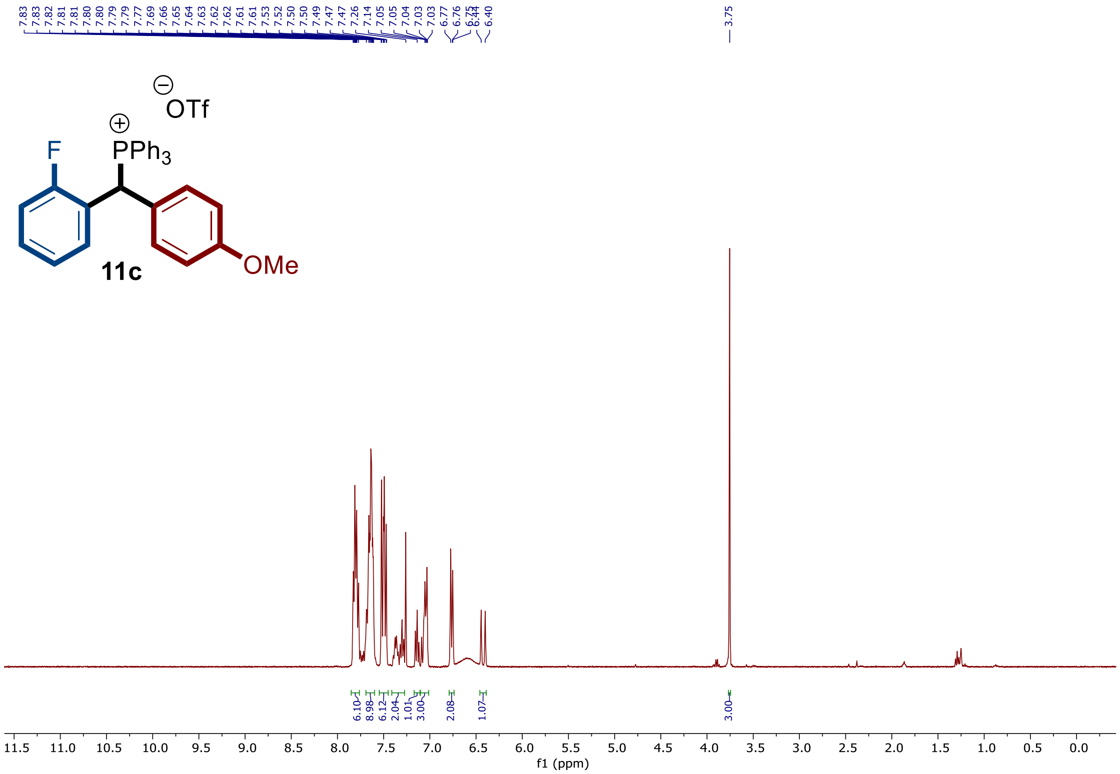

^{1}H NMR (400 MHz, CDCl$_3$) of compound (**11c**)

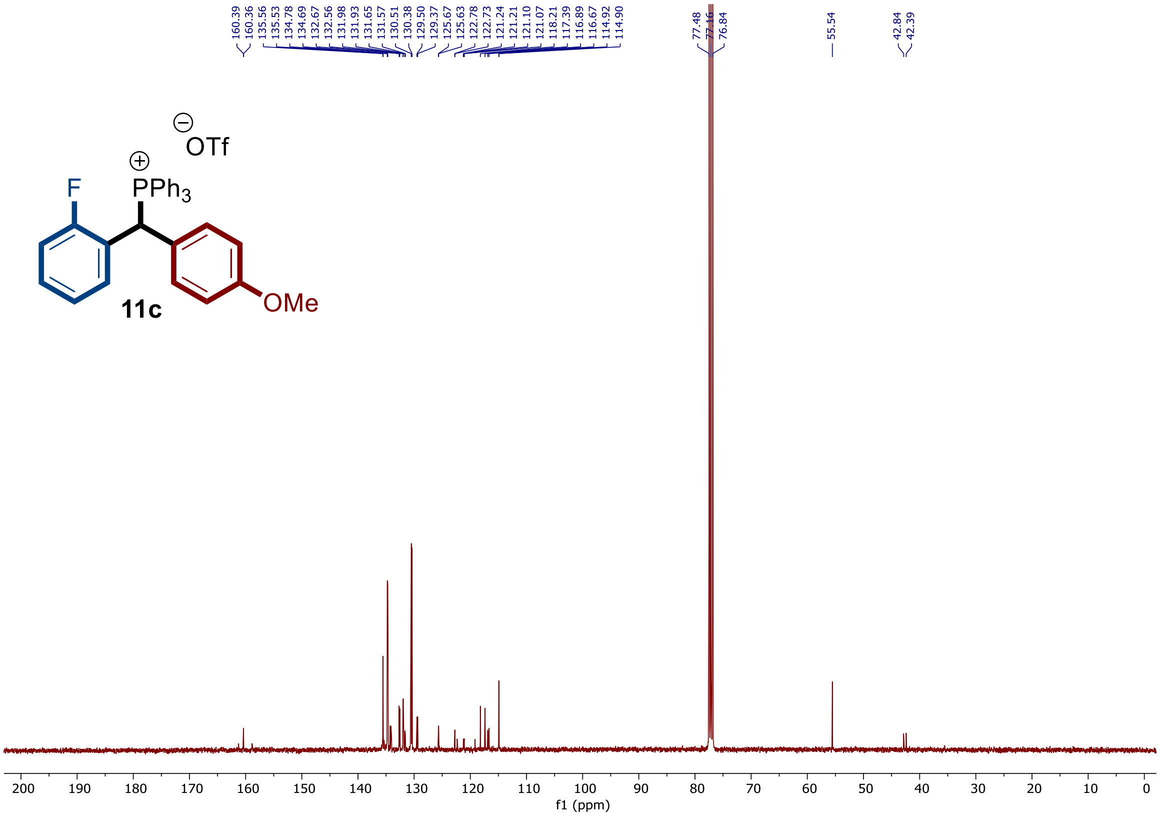

^{13}C NMR (101 MHz, $CDCl_3$) of compound (**11c**)

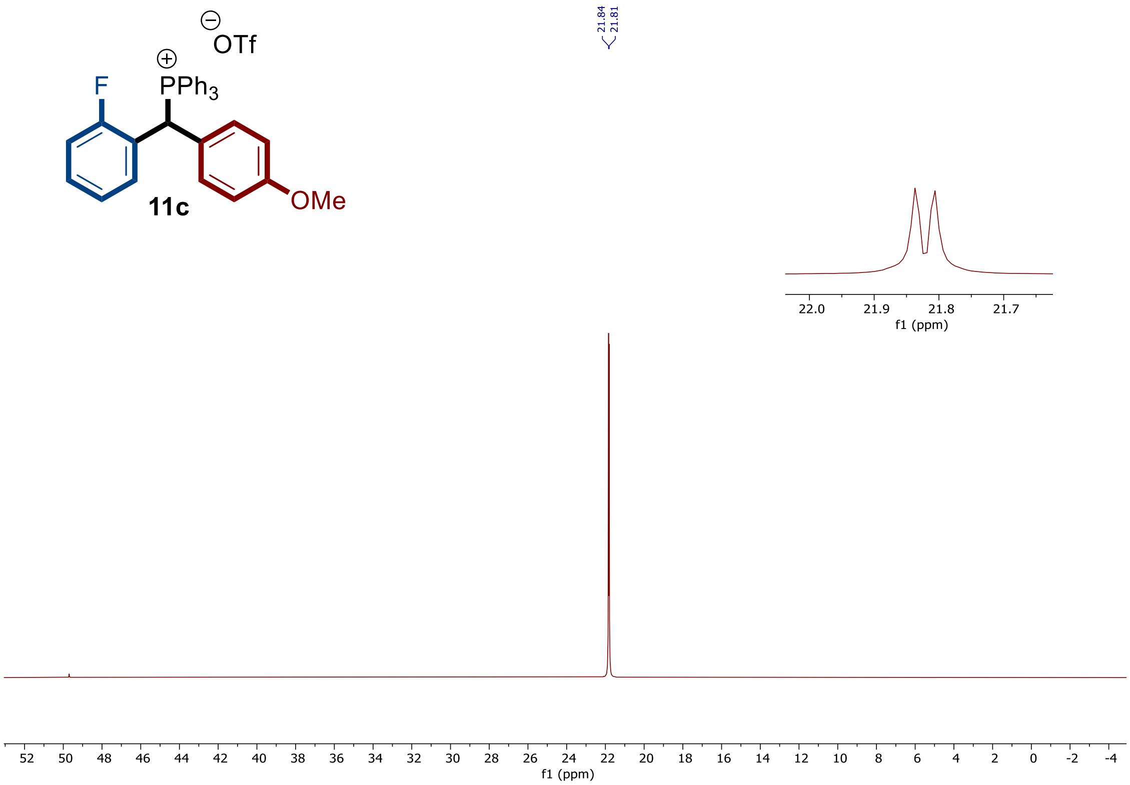

^{31}P NMR (162 MHz, $CDCl_3$) of compound (**11c**)

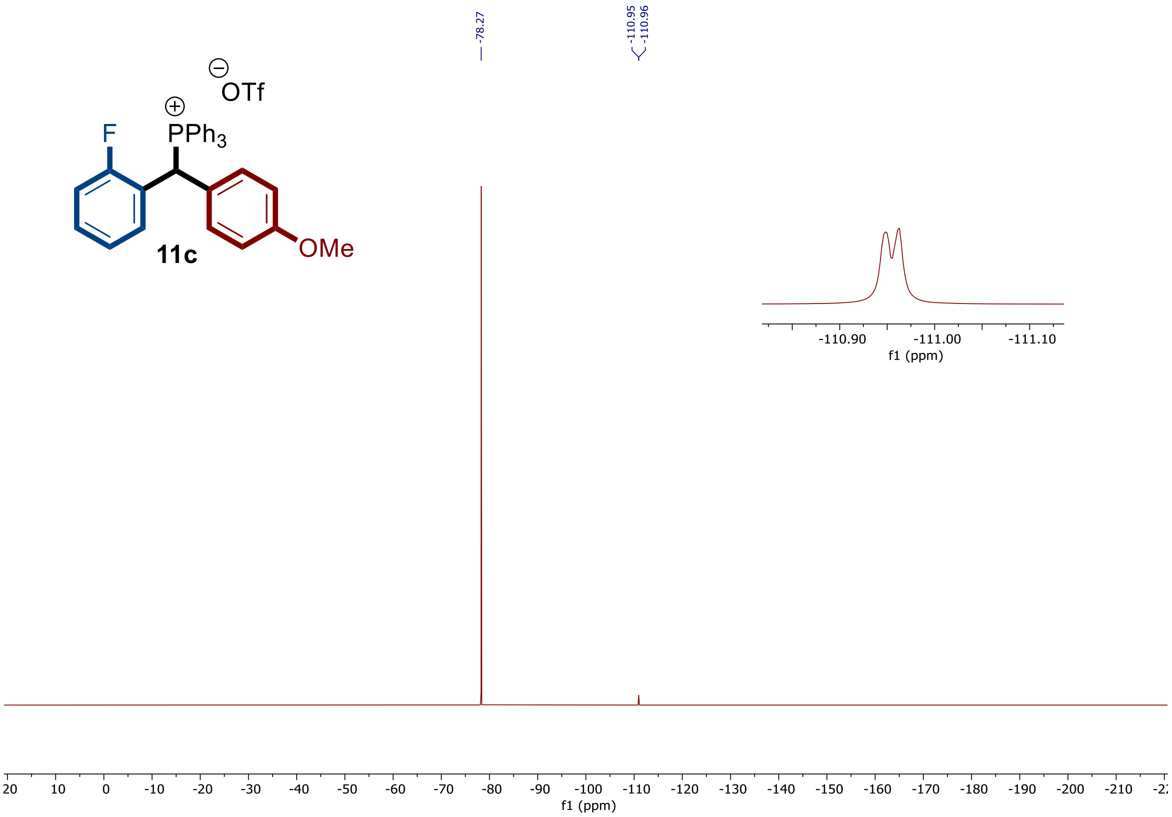

^{19}F NMR (376 MHz, $CDCl_3$) of compound (**11c**)

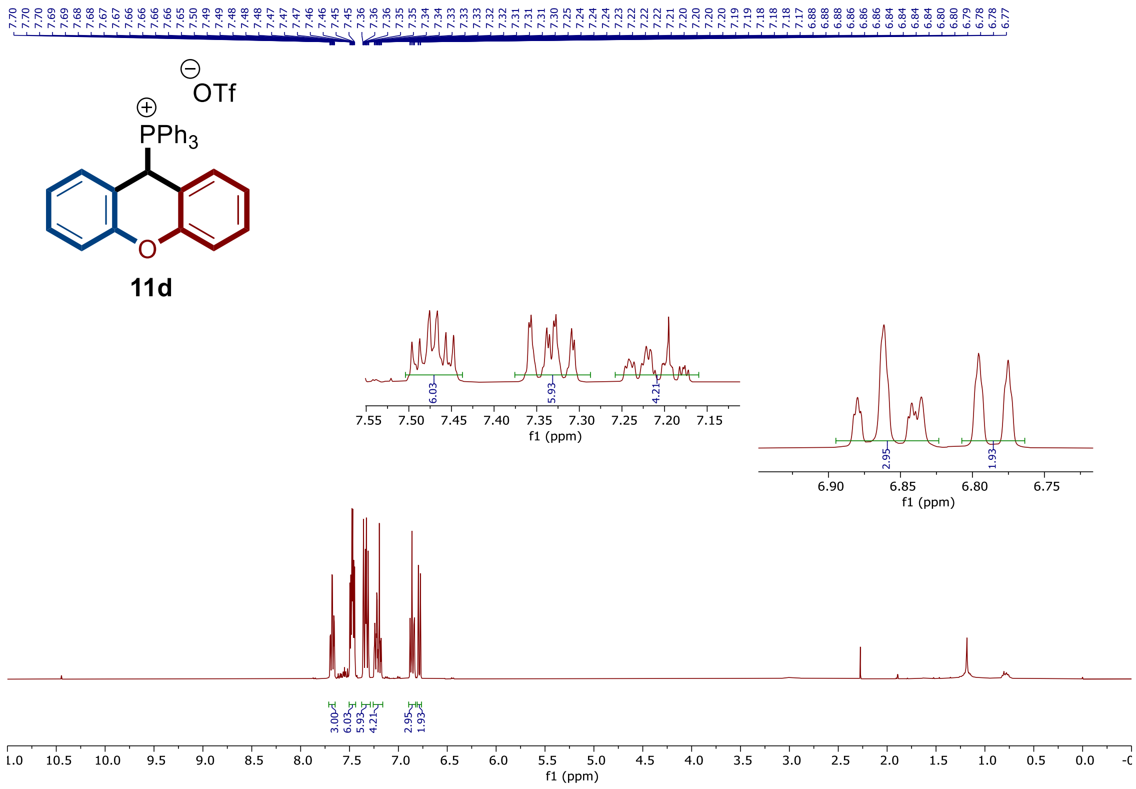

^{1}H NMR (400 MHz, $CDCl_3$) of compound (**11d**)

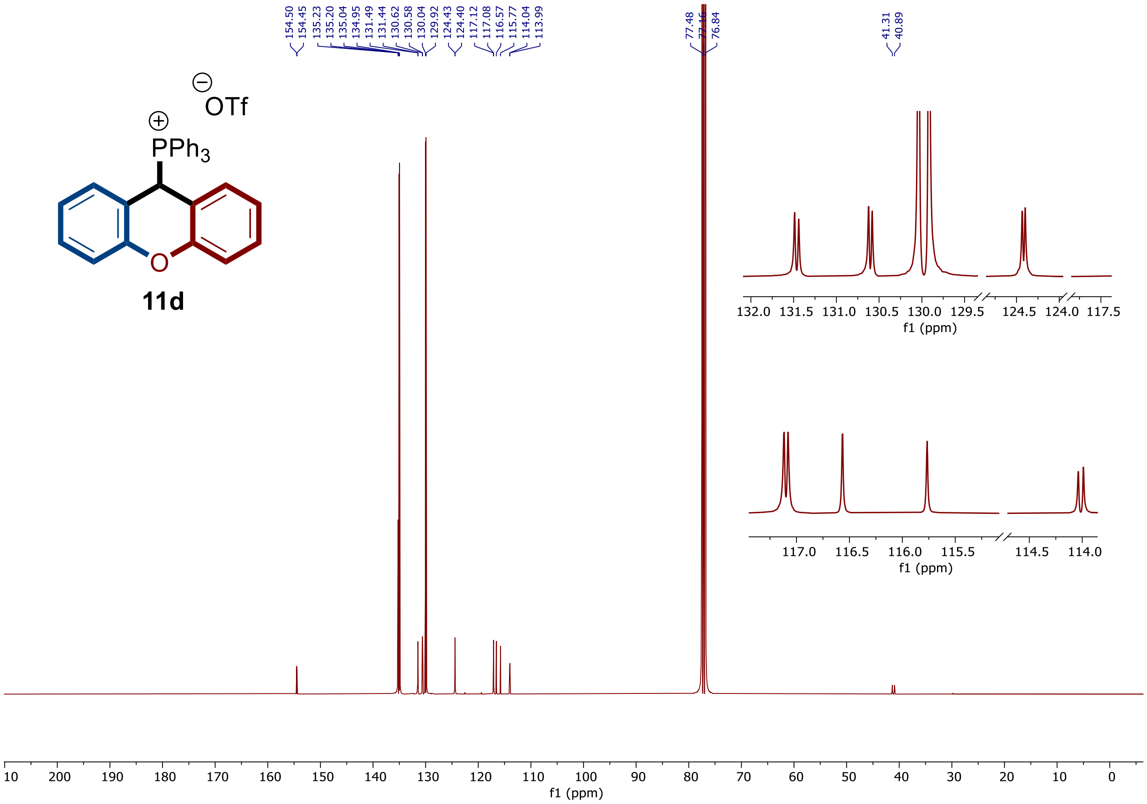

^{13}C NMR (101 MHz, $CDCl_3$) of compound (**11d**)

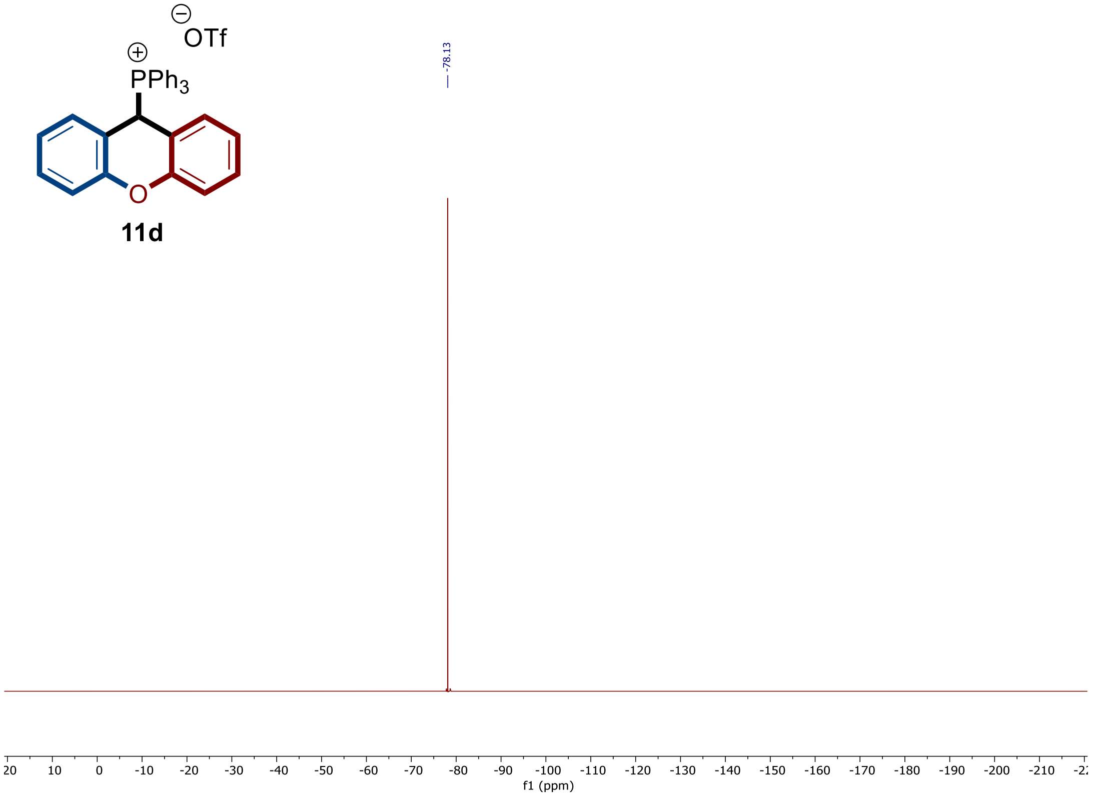

^{19}F NMR (376 MHz, $CDCl_3$) of compound (**11d**)

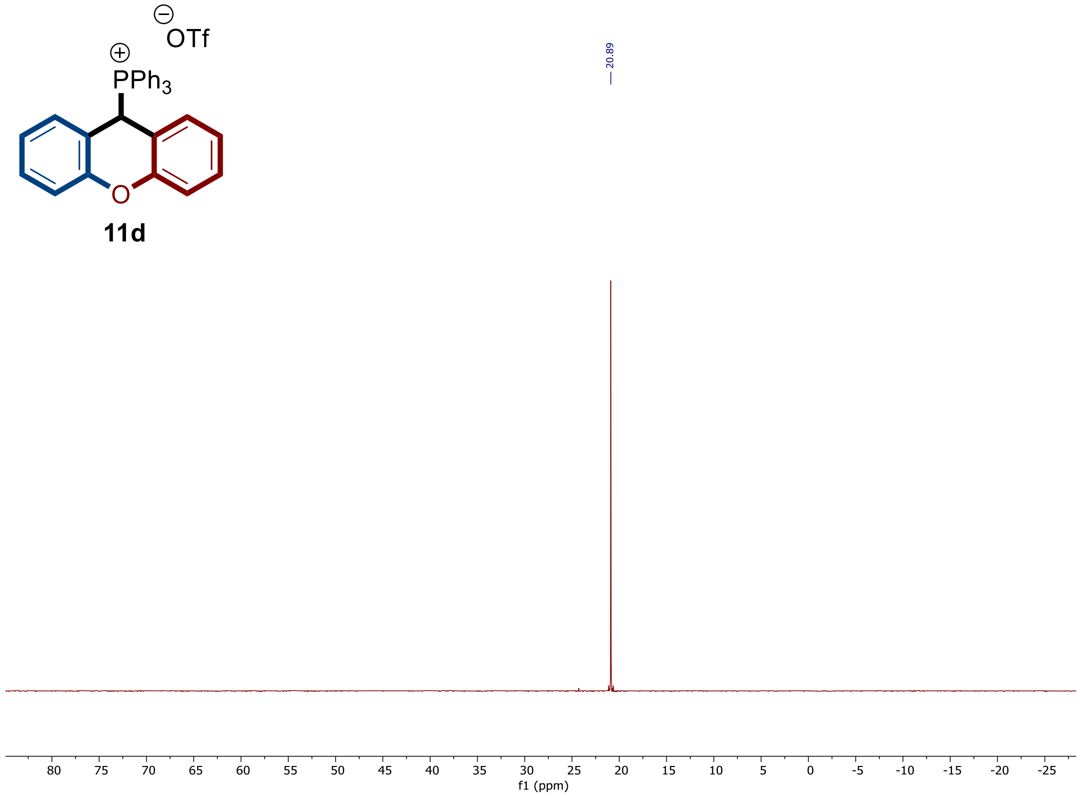

^{31}P NMR (162 MHz, CDCl$_3$) of compound (**11d**)

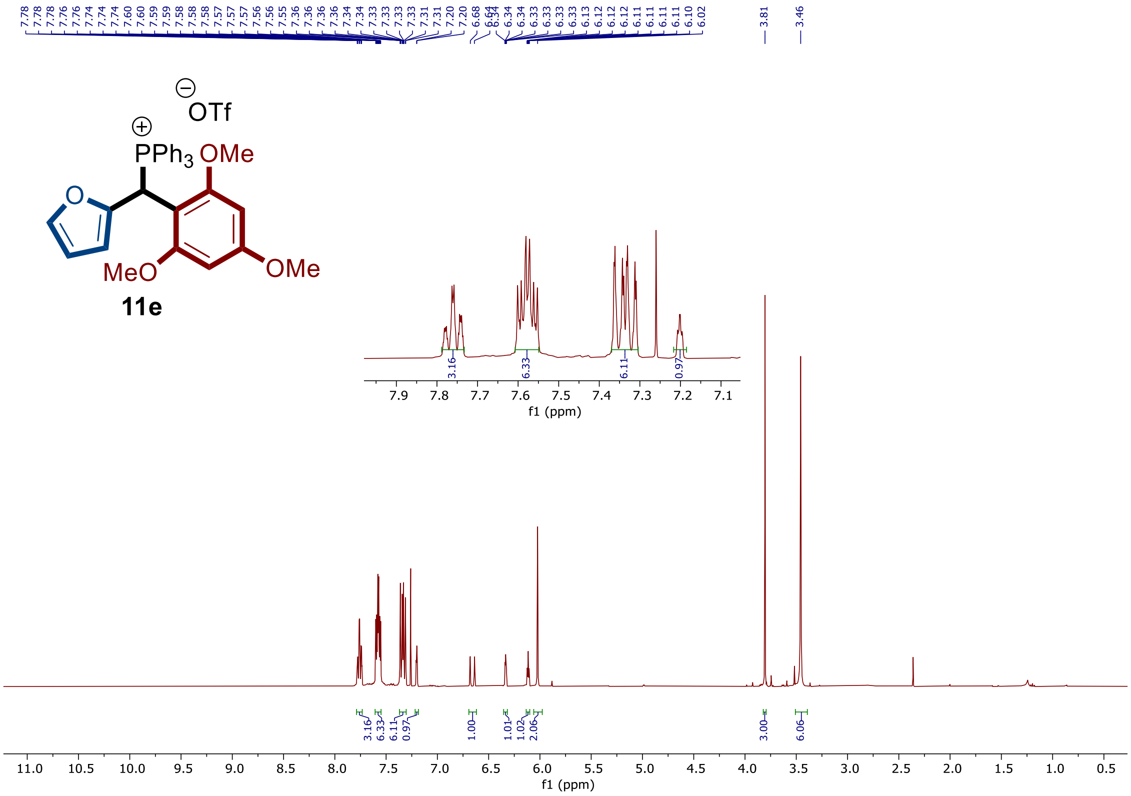

^{1}H NMR (400 MHz, CDCl$_3$) of compound (**11e**)

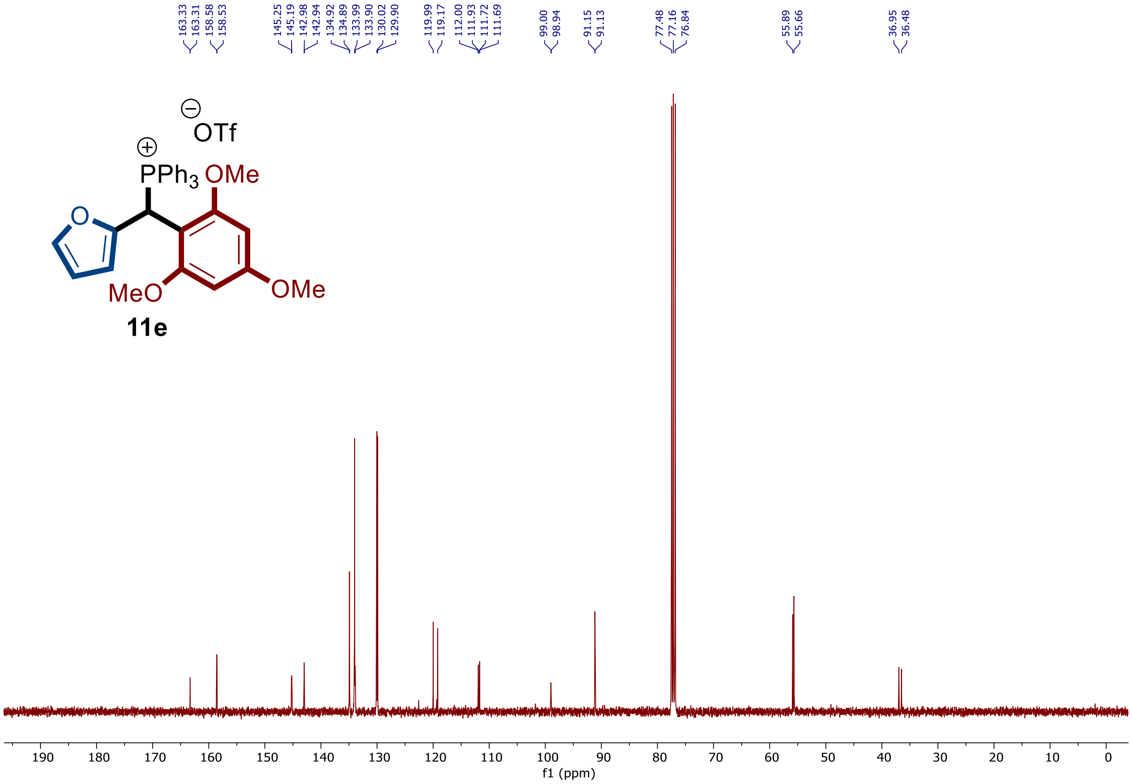

^{13}C NMR (101 MHz, $CDCl_3$) of compound (**11e**)

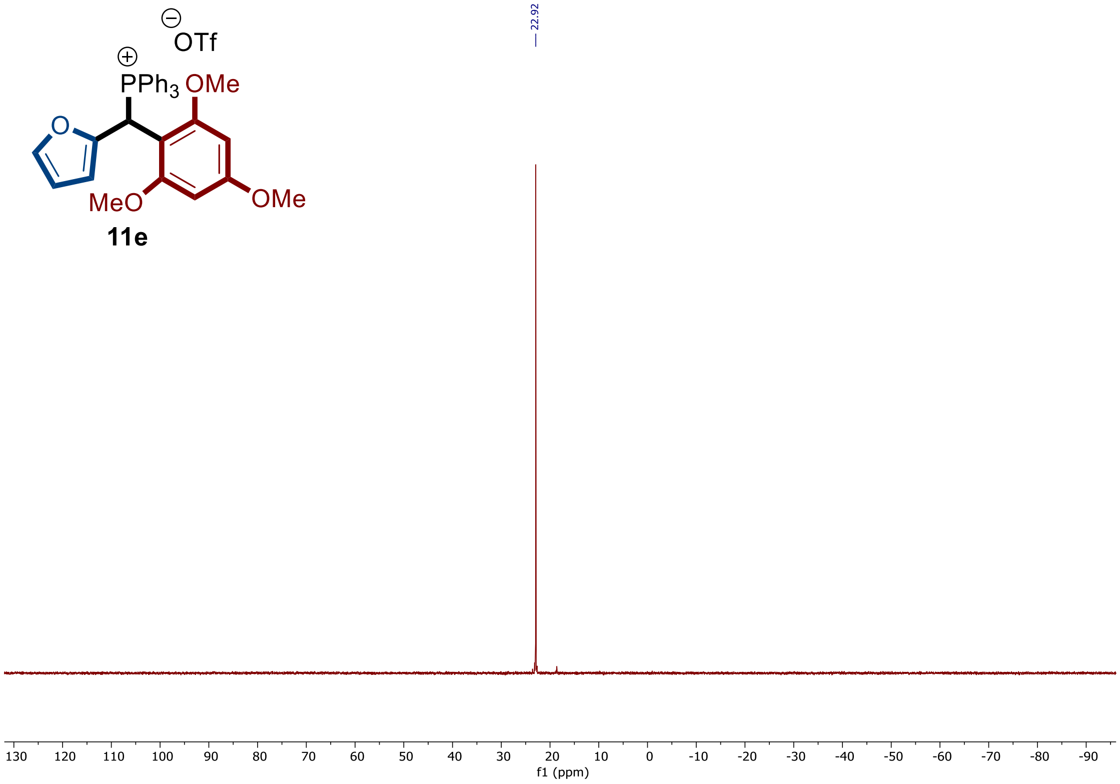

^{31}P NMR (162 MHz, $CDCl_3$) of compound (**11e**)

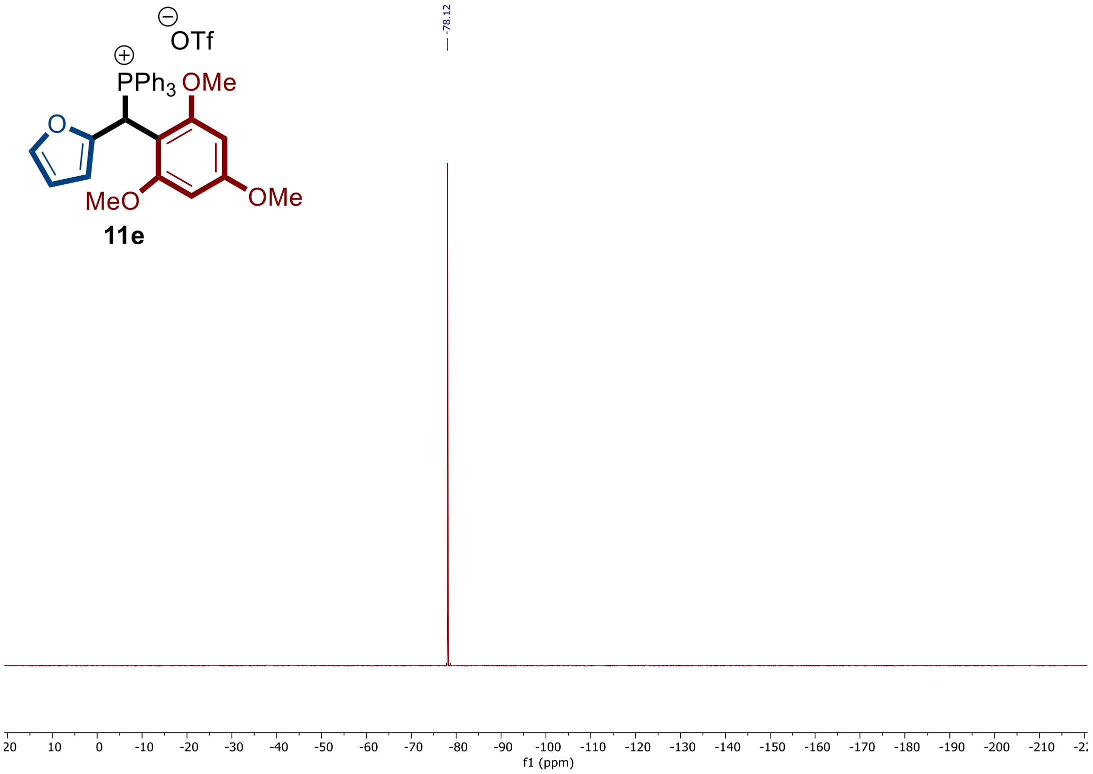

^{19}F NMR (376 MHz, CDCl$_3$) of compound (**11e**)

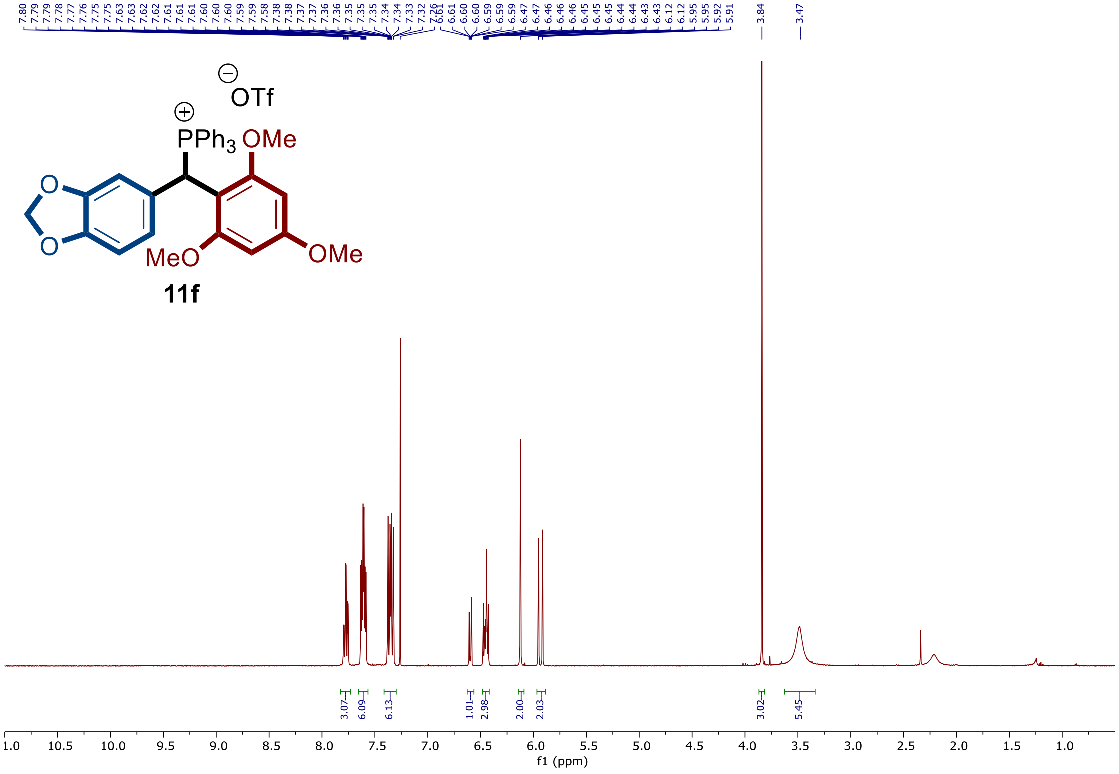

^{1}H NMR (400 MHz, CDCl$_3$) of compound (**11f**)

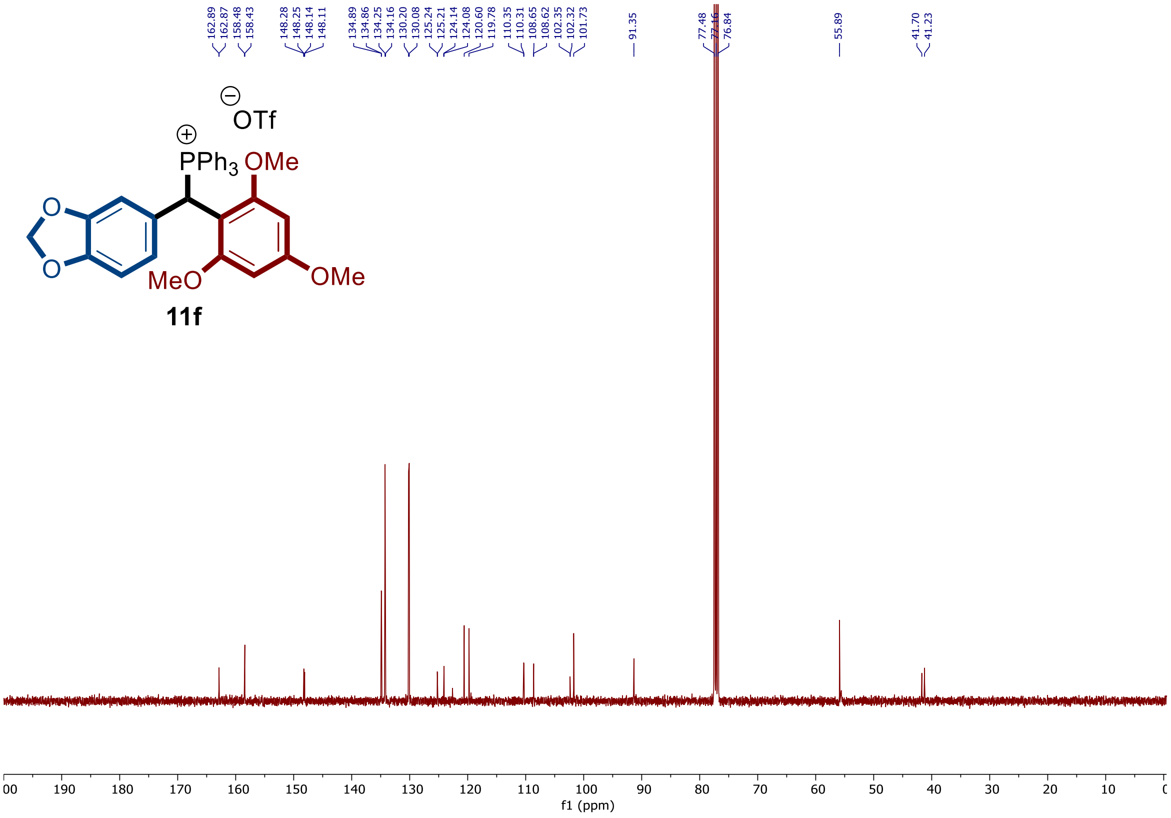

^{13}C NMR (101 MHz, $CDCl_3$) of compound (**11f**)

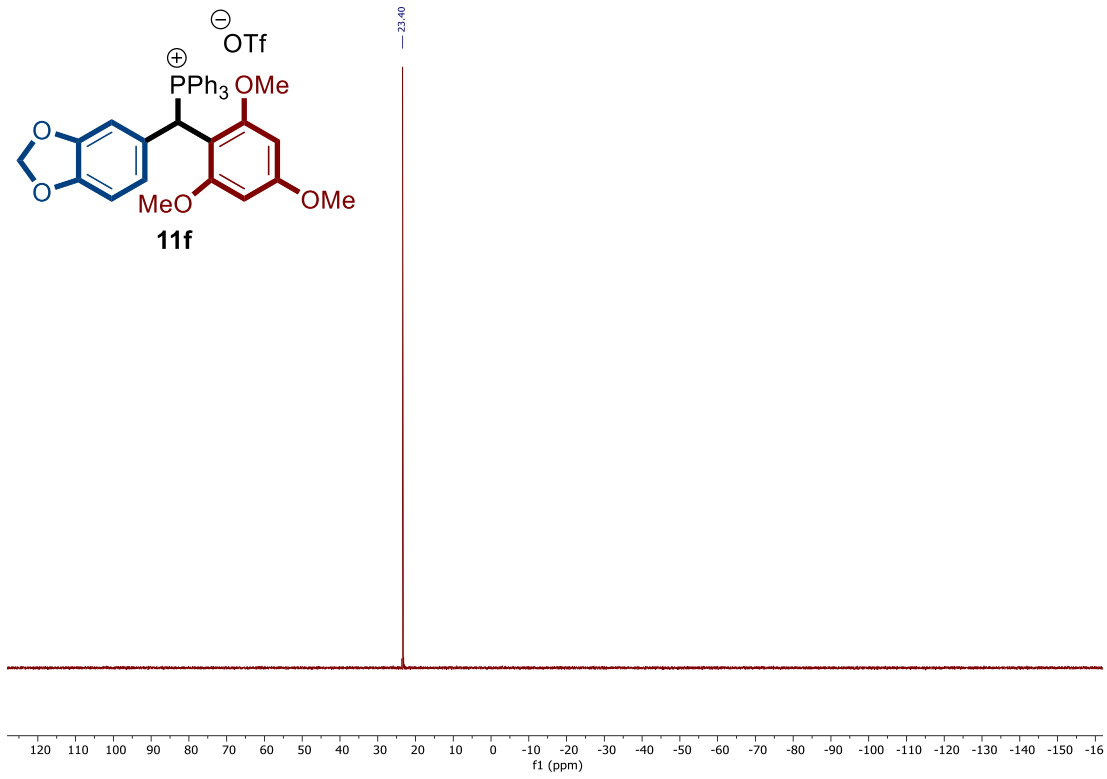

^{31}P NMR (162 MHz, $CDCl_3$) of compound (**11f**)

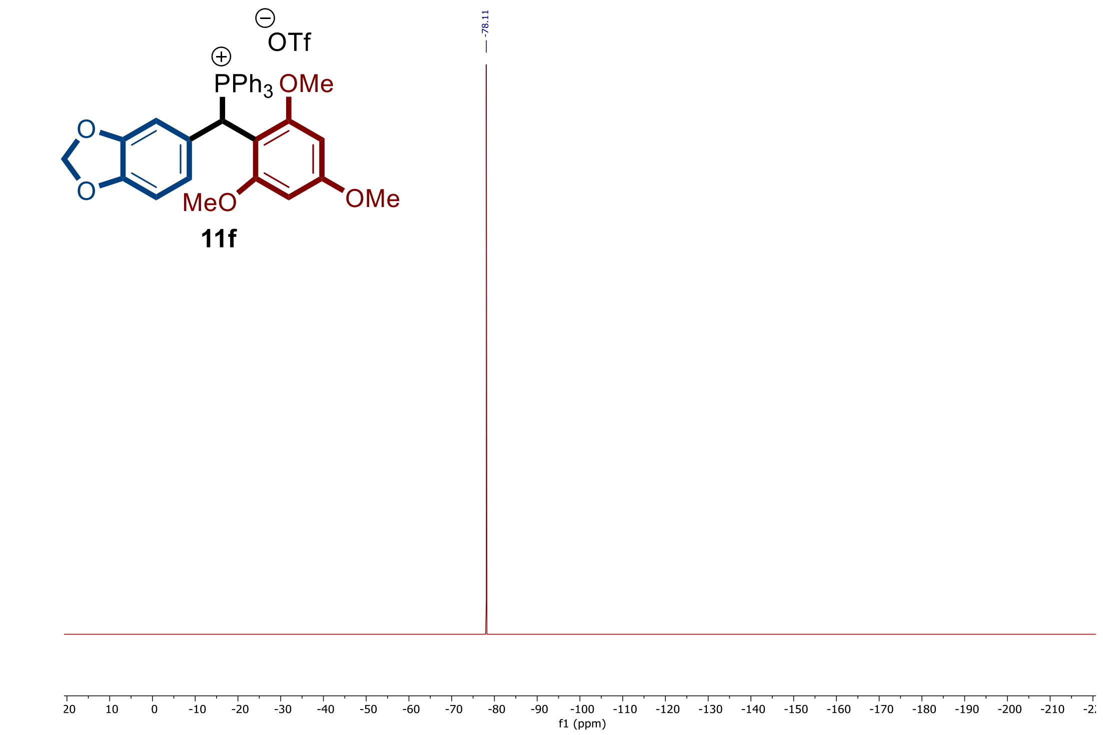

^{19}F NMR (376 MHz, $CDCl_3$) of compound (**11f**)

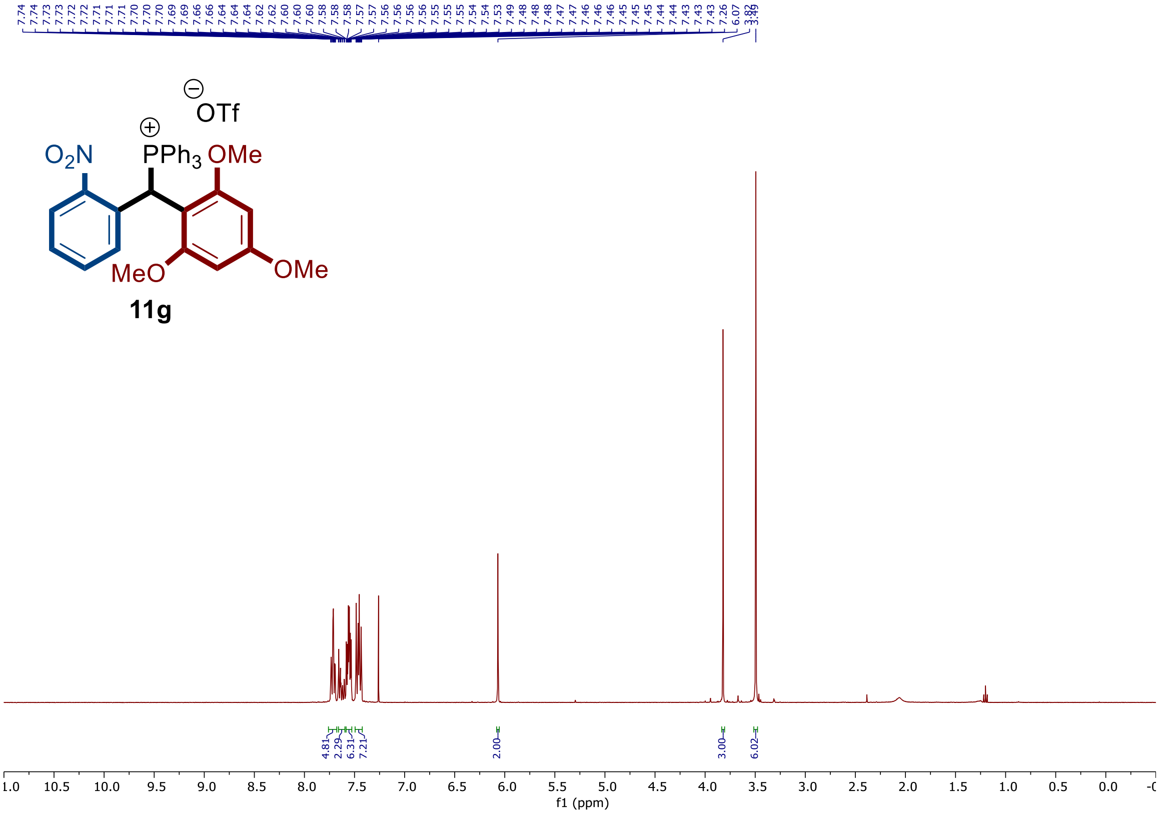

^{1}H NMR (400 MHz, $CDCl_3$) of compound (**11g**)

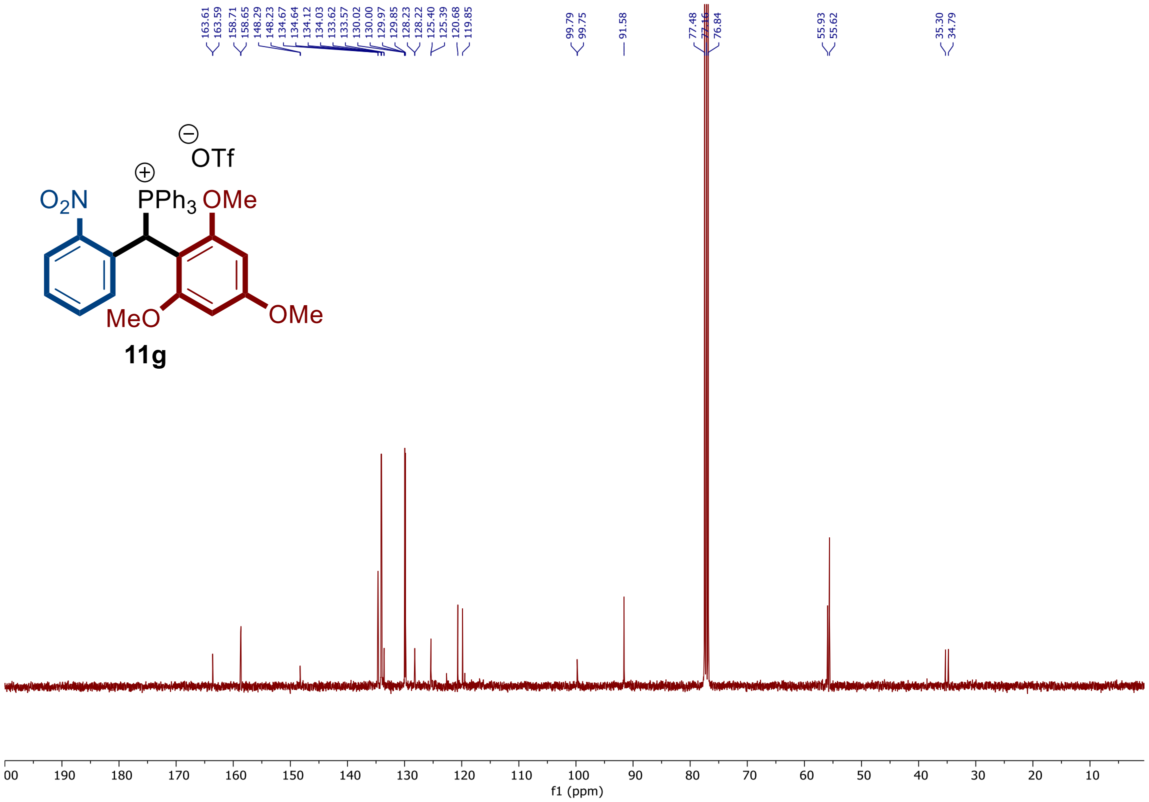

^{13}C NMR (101 MHz, $CDCl_3$) of compound (**11g**)

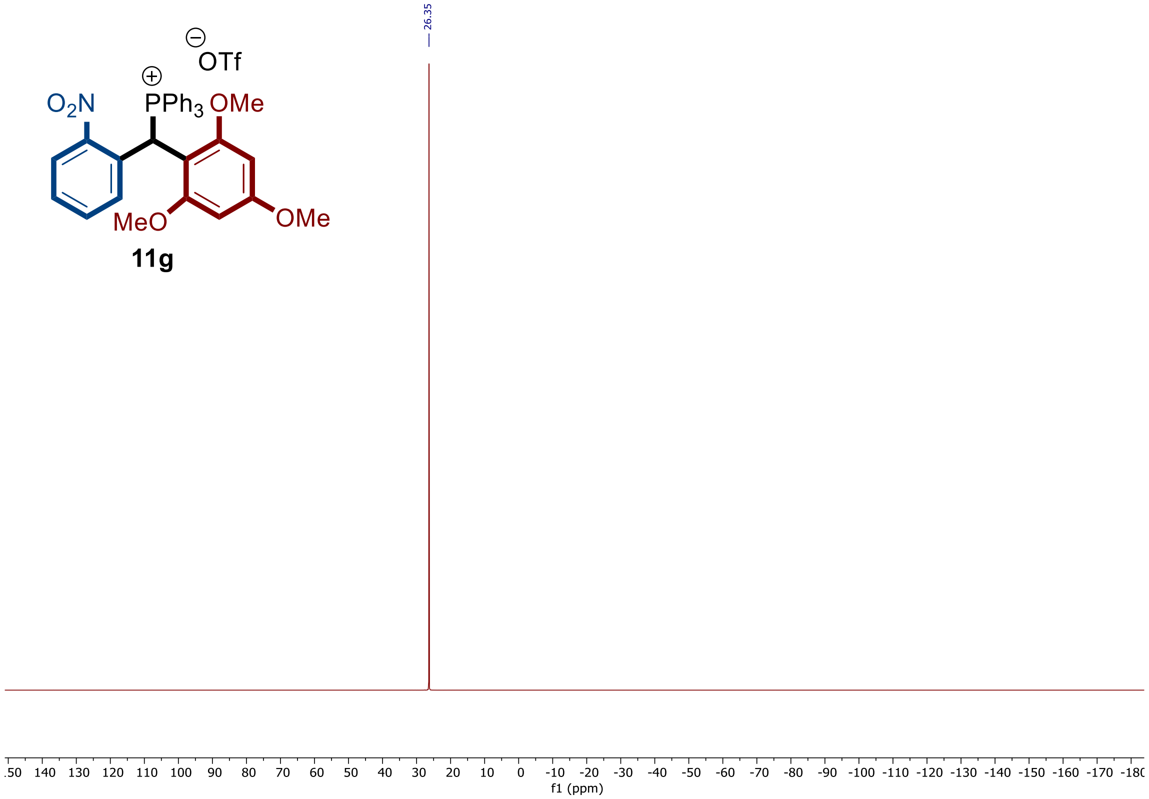

^{31}P NMR (162 MHz, $CDCl_3$) of compound (**11g**)

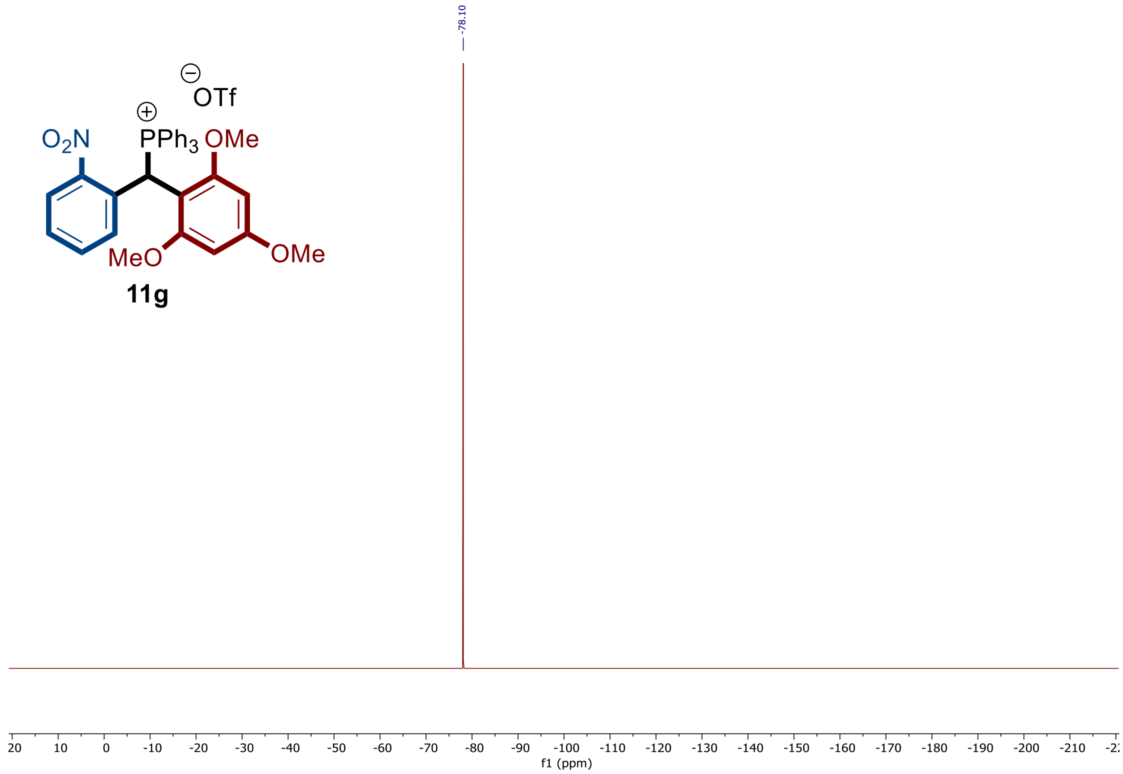

^{19}F NMR (376 MHz, $CDCl_3$) of compound (**11g**)

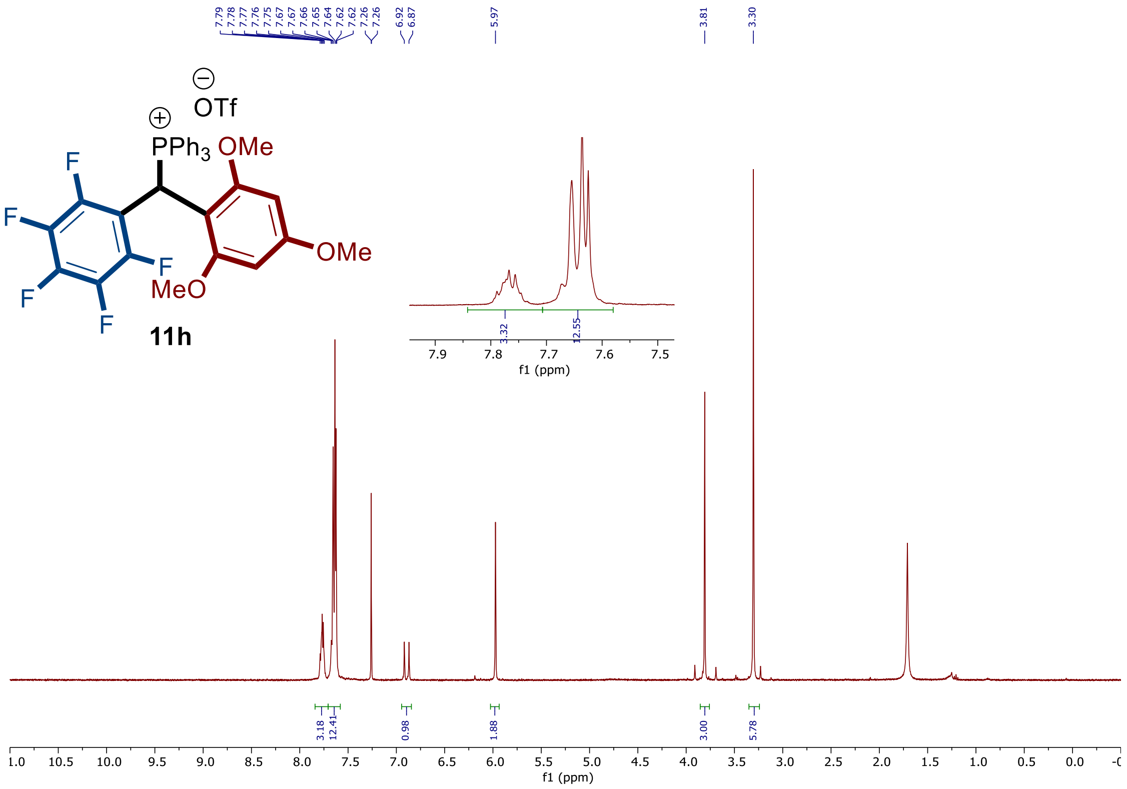

^{1}H NMR (400 MHz, $CDCl_3$) of compound (**11h**)

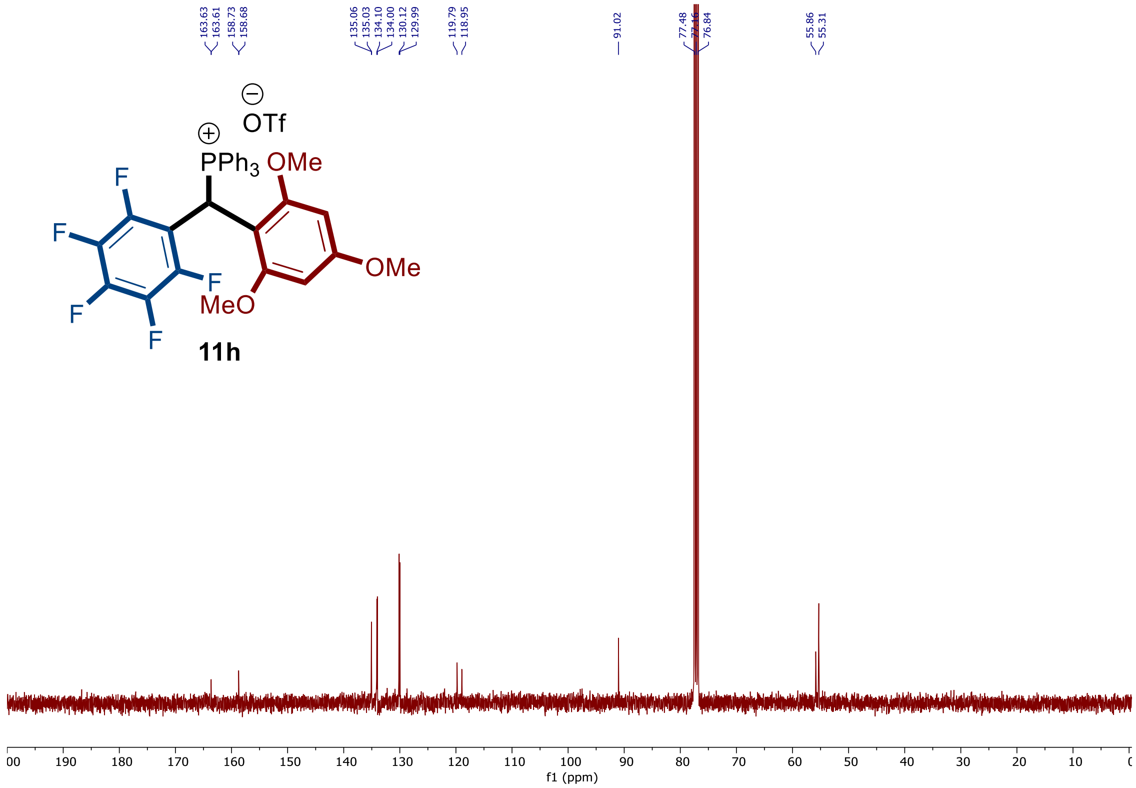

^{13}C NMR (101 MHz, $CDCl_3$) of compound (**11h**)

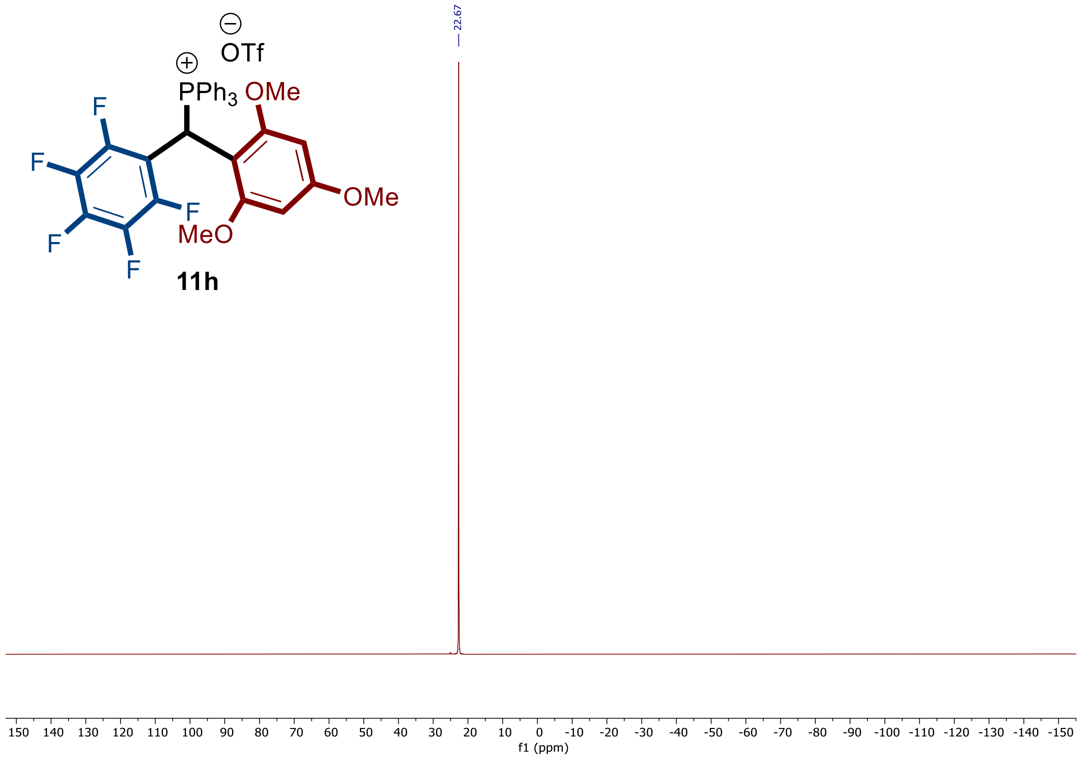

^{31}P NMR (162 MHz, $CDCl_3$) of compound (**11h**)

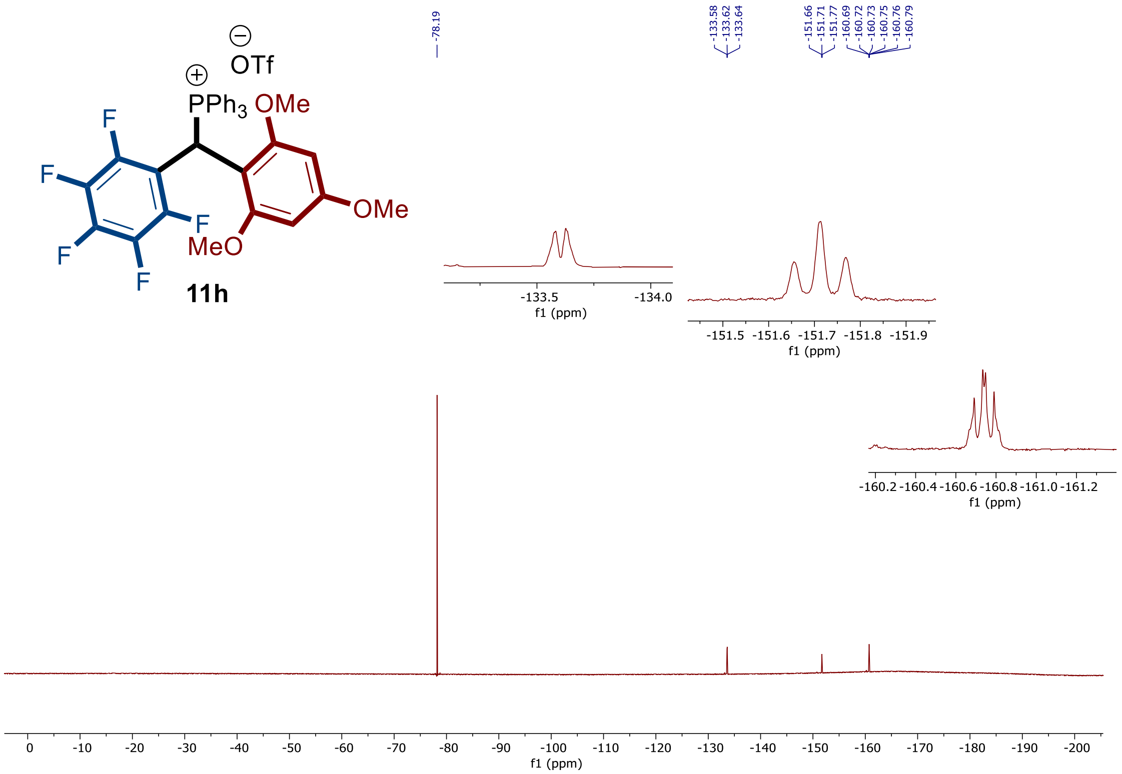

^{19}F NMR (376 MHz, $CDCl_3$) of compound (**11h**)

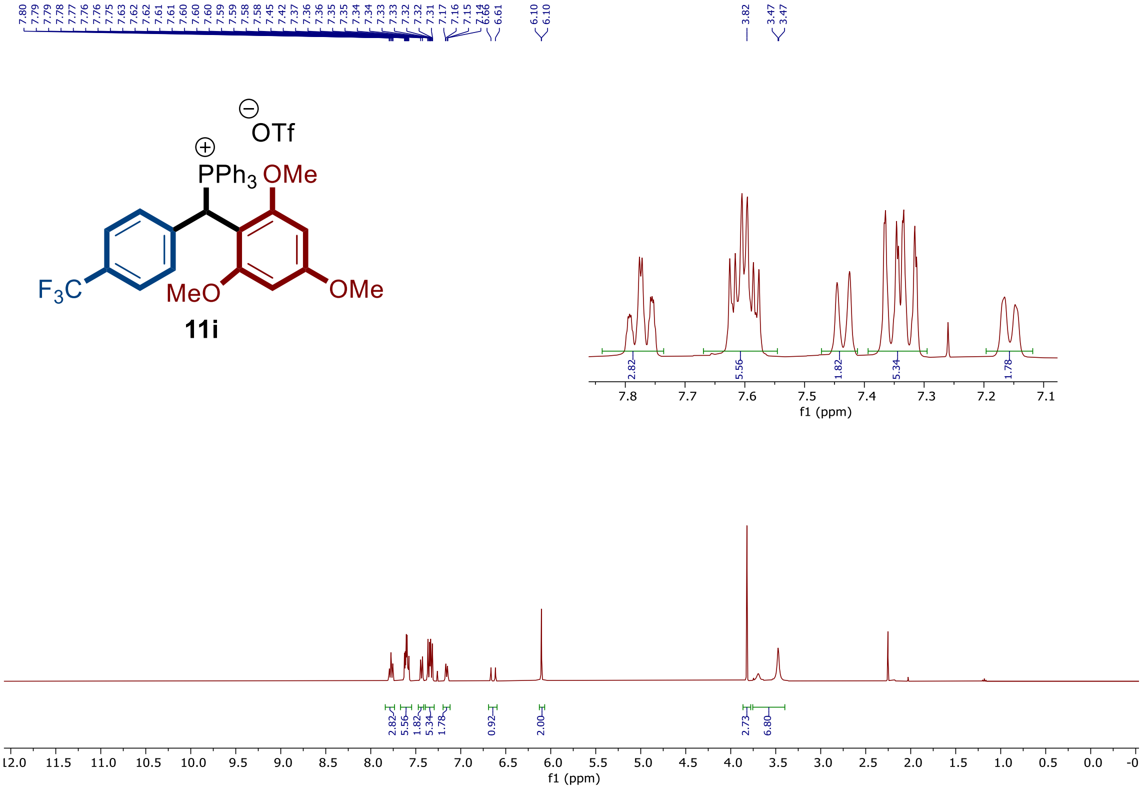

^{1}H NMR (400 MHz, $CDCl_3$) of compound (**11i**)

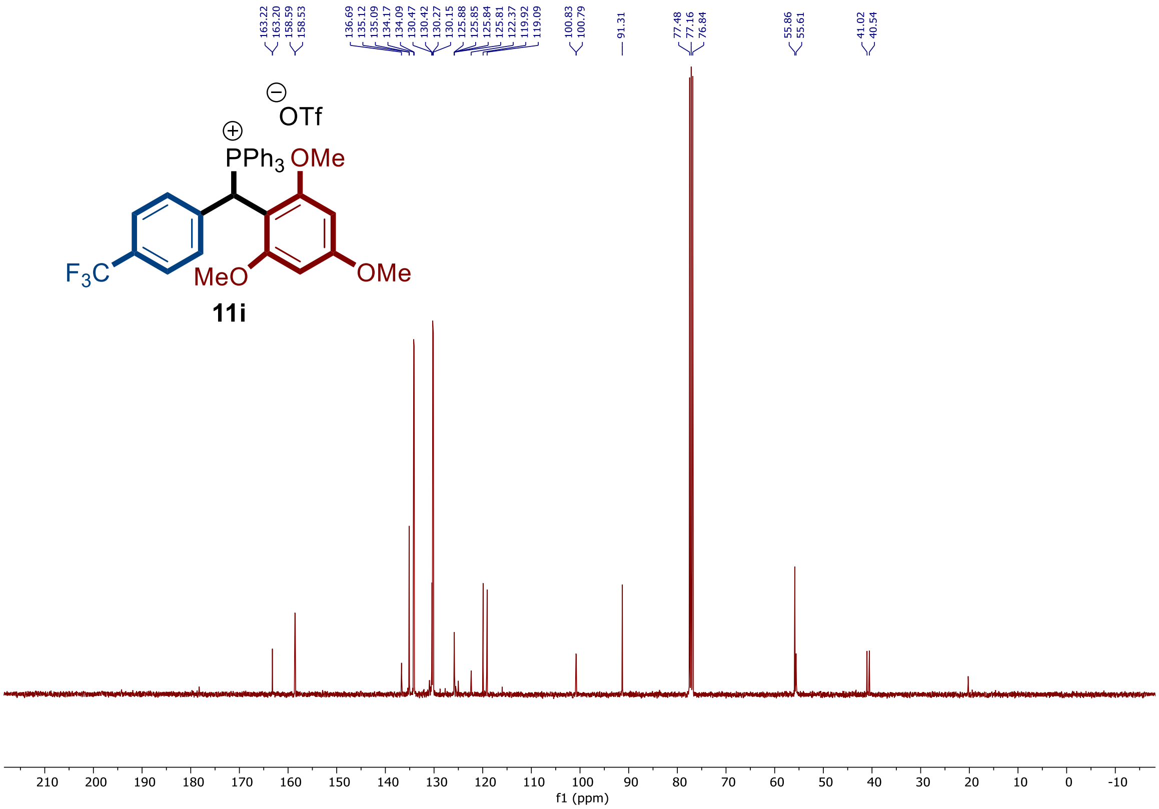

^{13}C NMR (101 MHz, $CDCl_3$) of compound (**11i**)

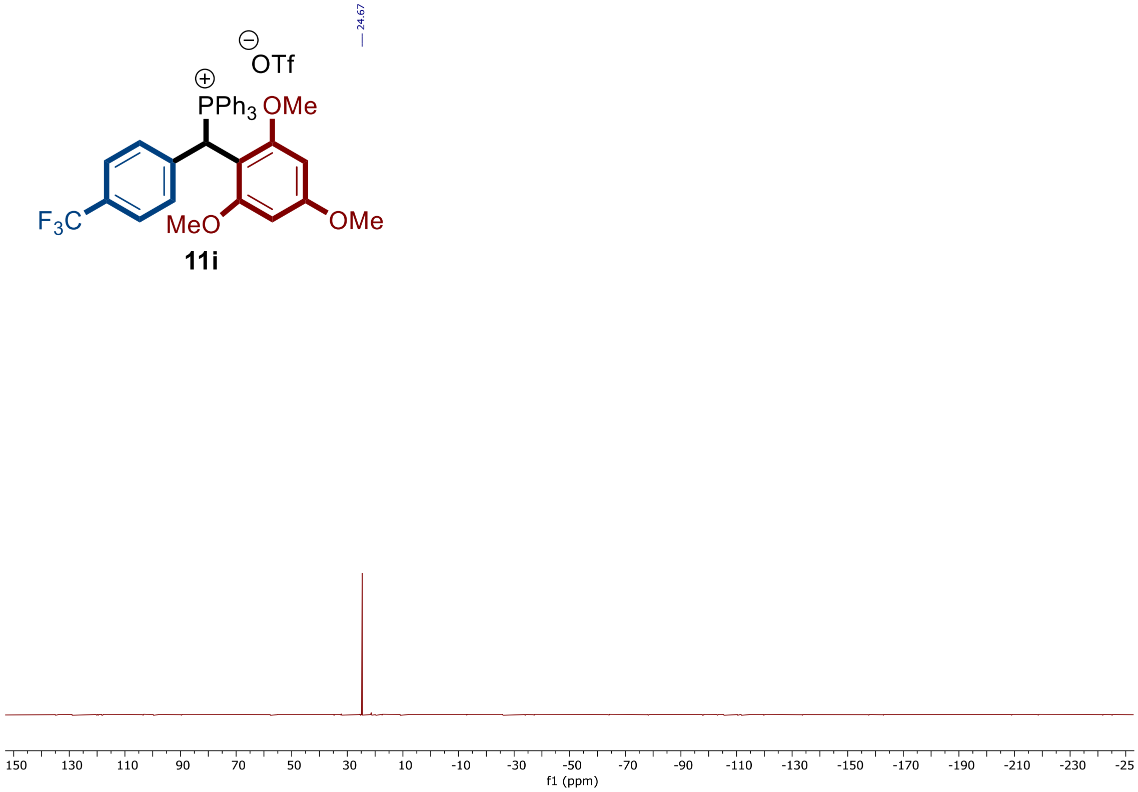

^{31}P NMR (162 MHz, $CDCl_3$) of compound (**11i**)

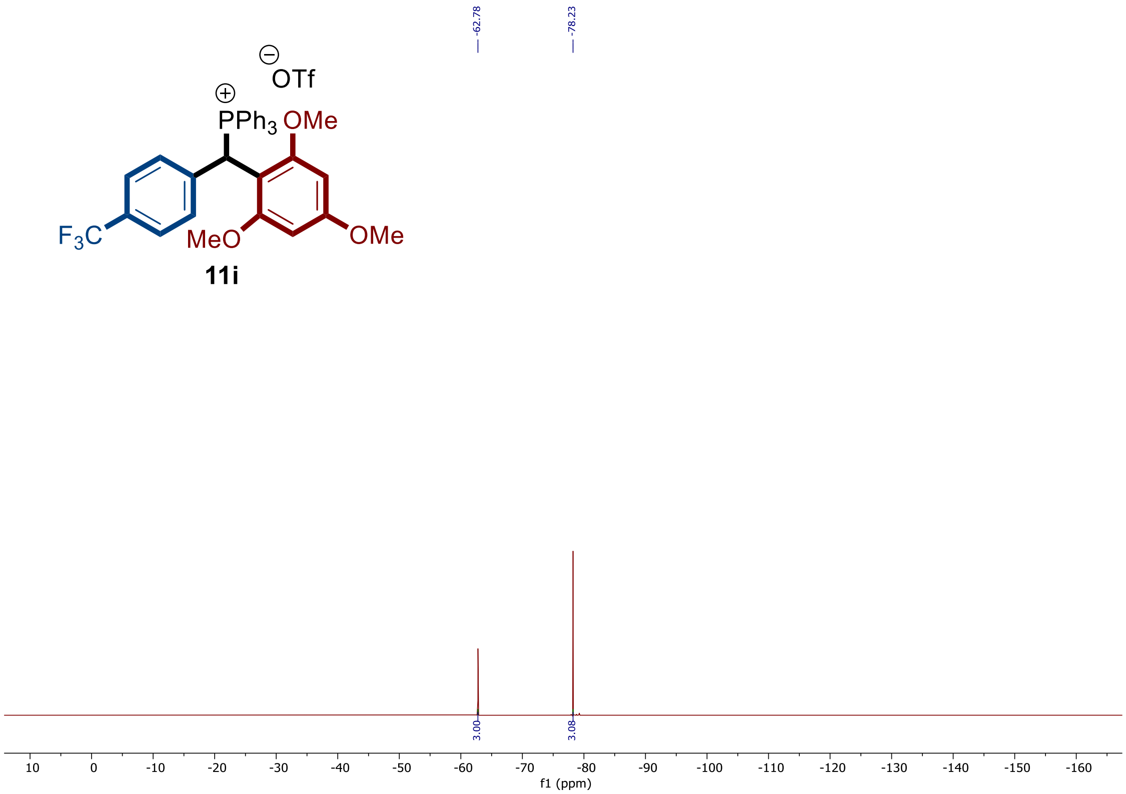

^{19}F NMR (376 MHz, CDCl$_3$) of compound (**11i**)

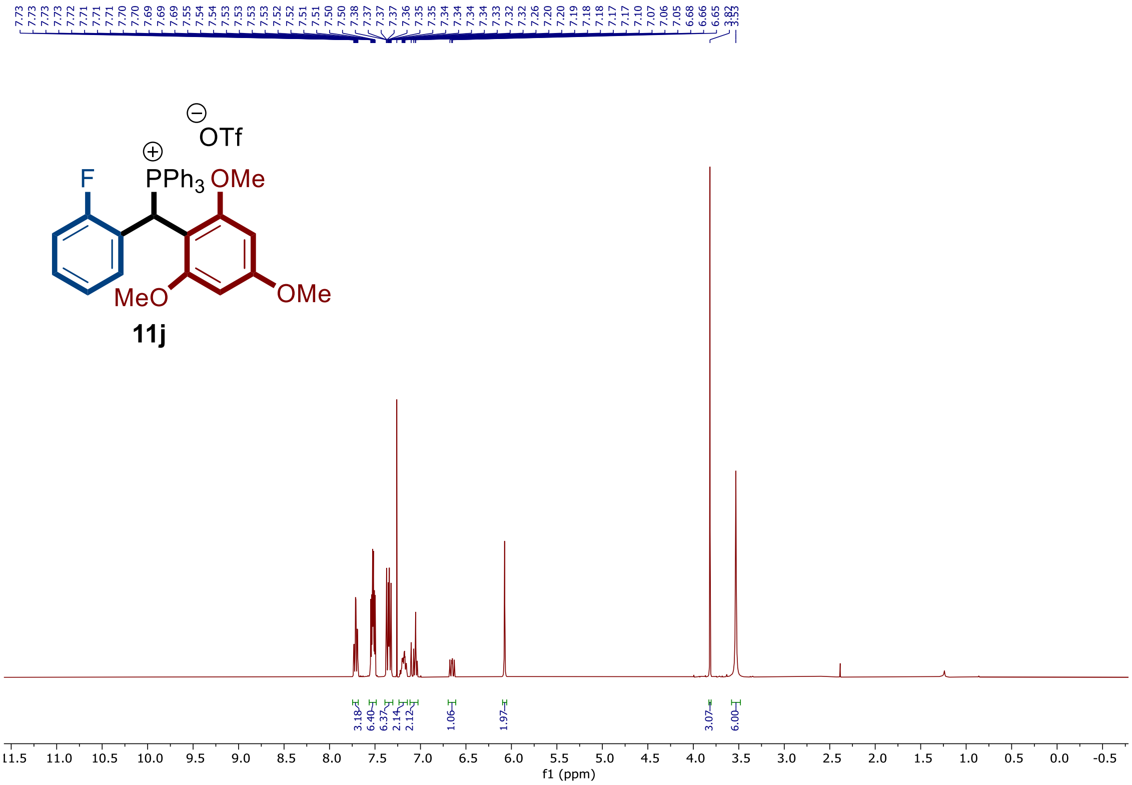

^{1}H NMR (400 MHz, CDCl$_3$) of compound (**11j**)

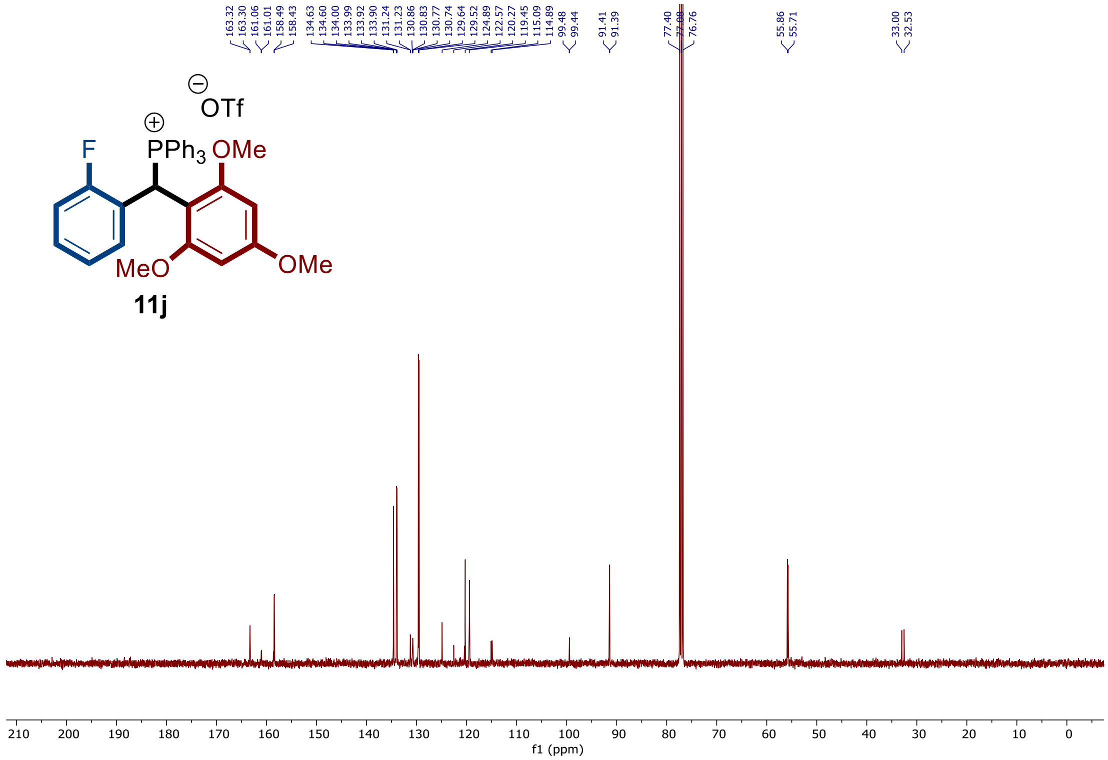

^{13}C NMR (101 MHz, $CDCl_3$) of compound (**11j**)

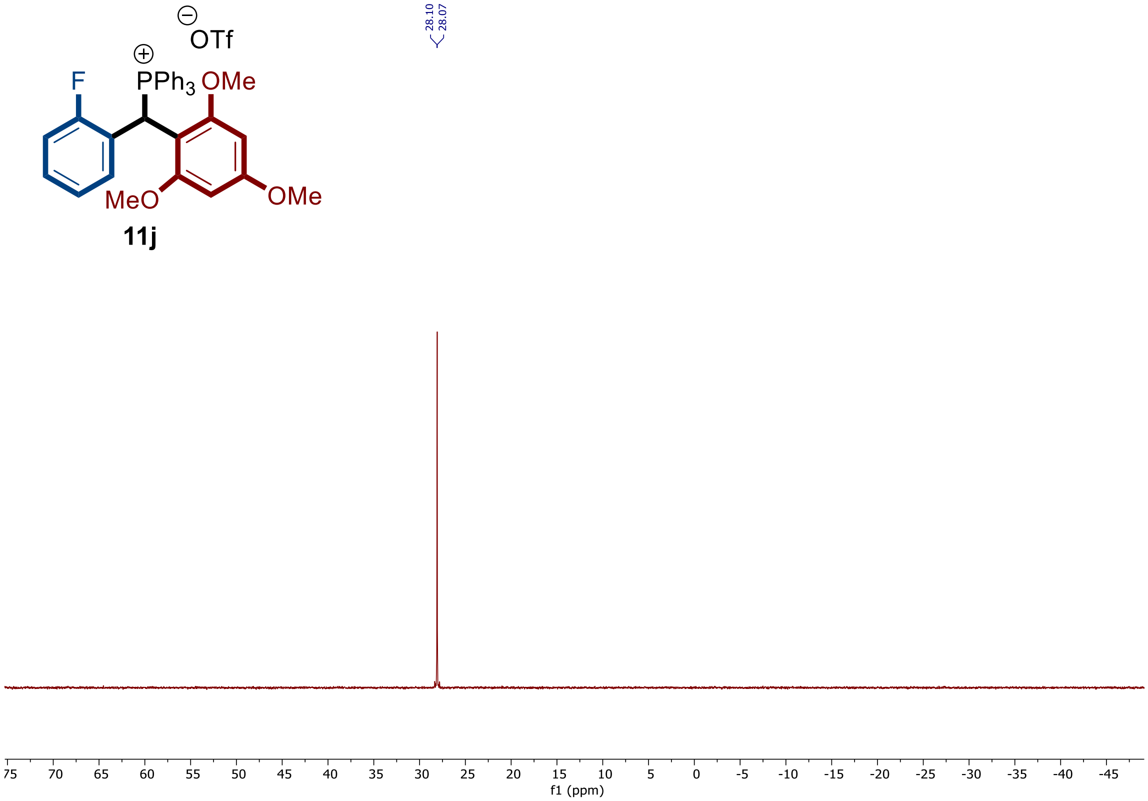

^{31}P NMR (162 MHz, $CDCl_3$) of compound (**11j**)

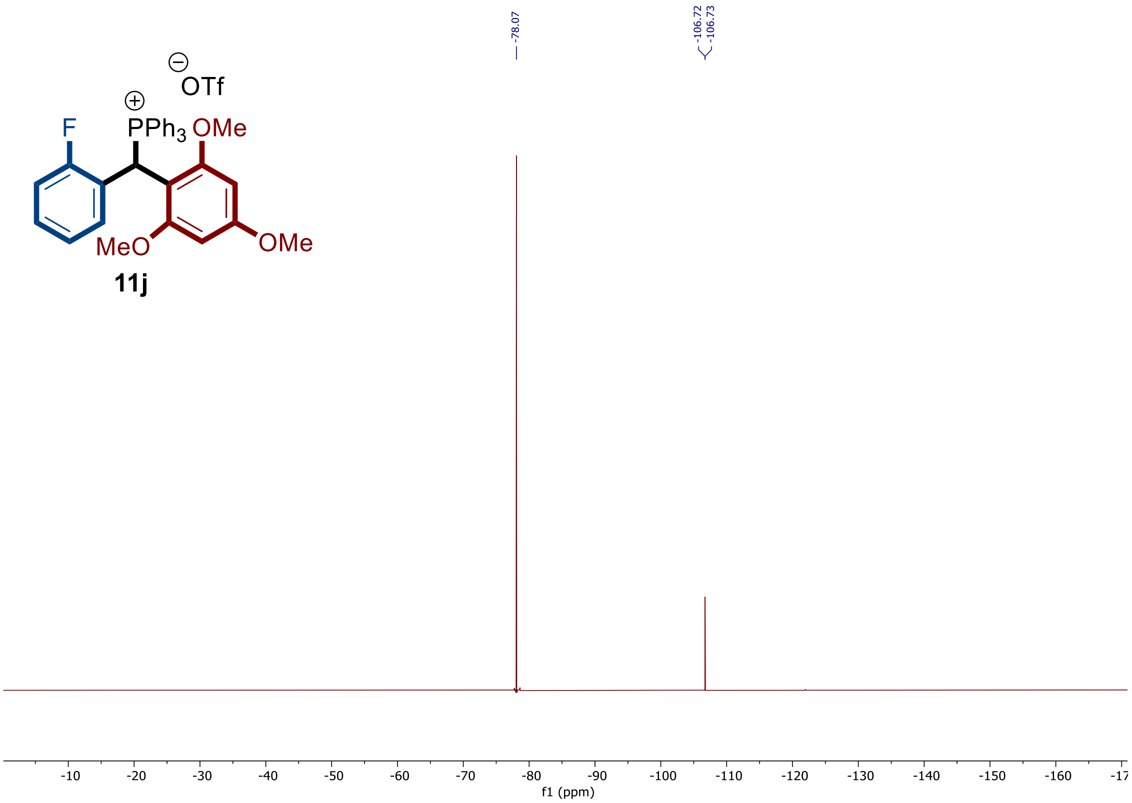

^{19}F NMR (376 MHz, CDCl$_3$) of compound (**11j**)

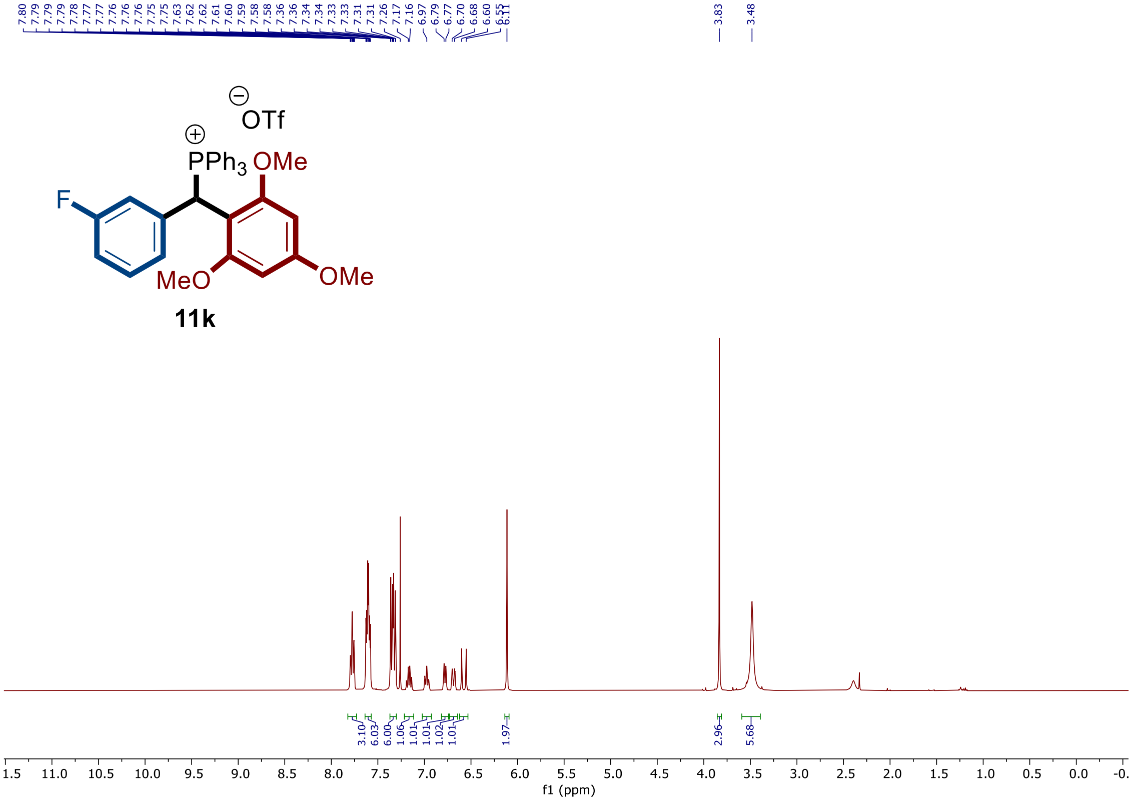

^{1}H NMR (400 MHz, CDCl$_3$) of compound (**11k**)

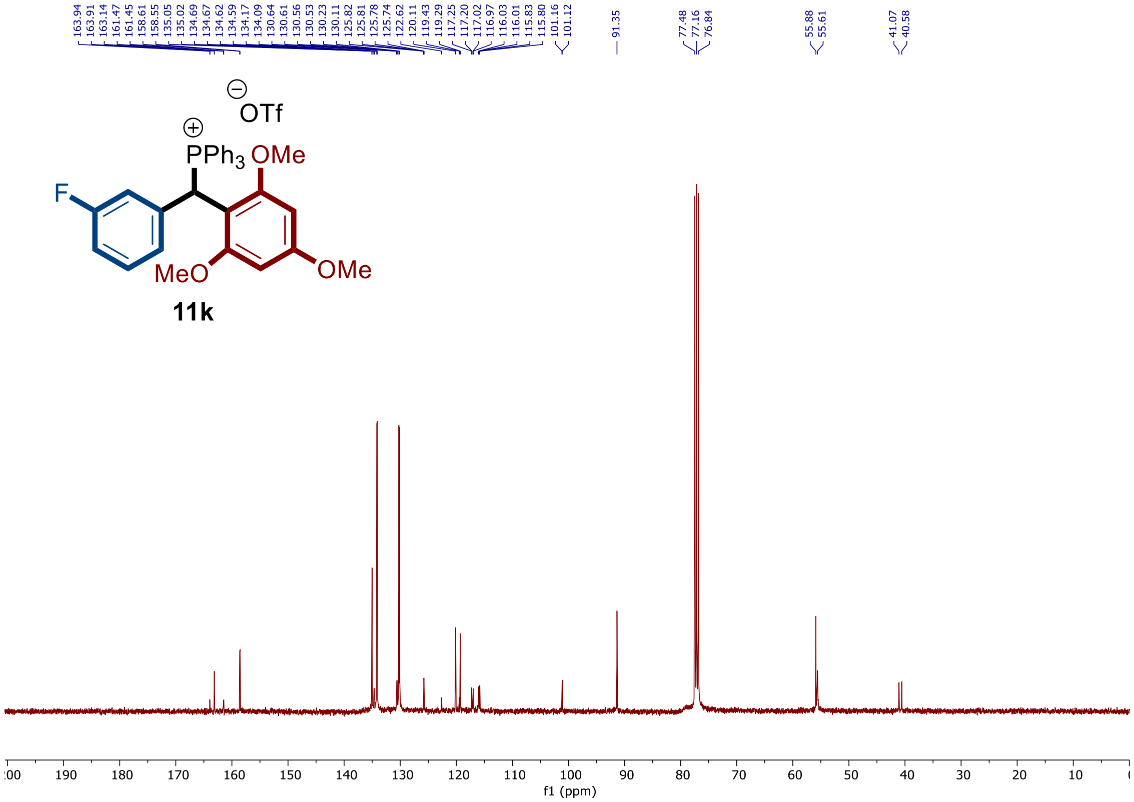

^{13}C NMR (101 MHz, $CDCl_3$) of compound (**11k**)

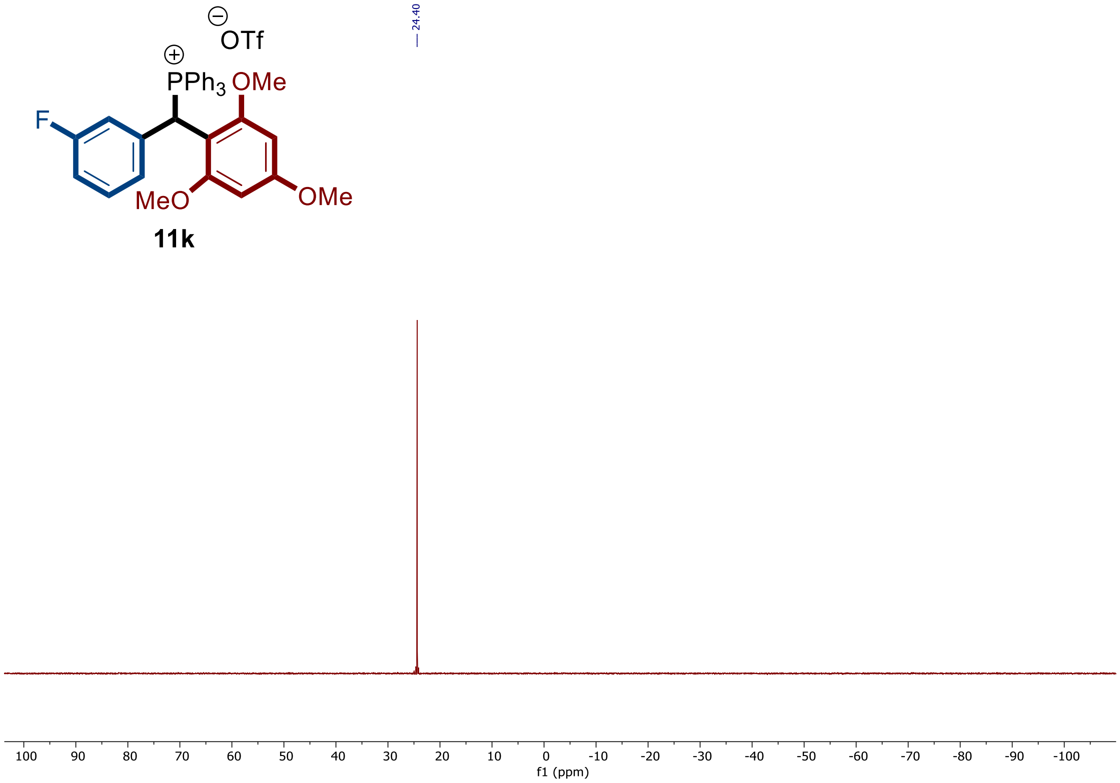

^{31}P NMR (162 MHz, $CDCl_3$) of compound (**11k**)

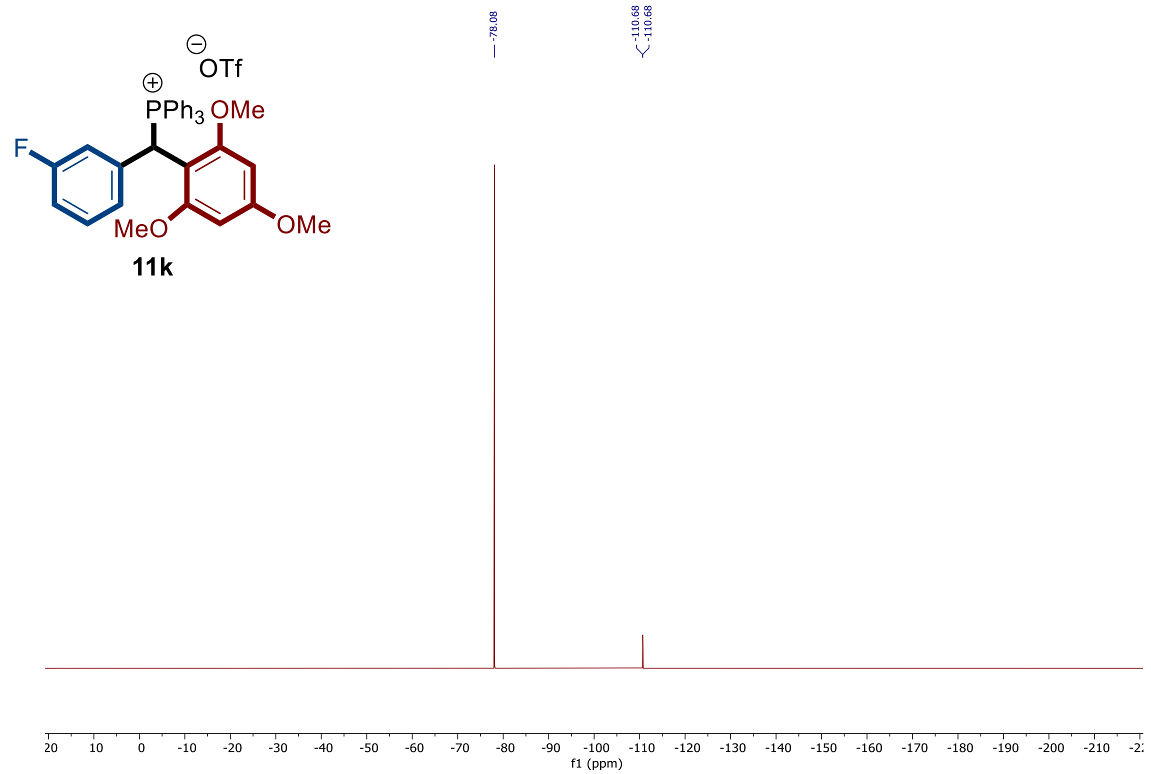

^{19}F NMR (376 MHz, $CDCl_3$) of compound (**11k**)

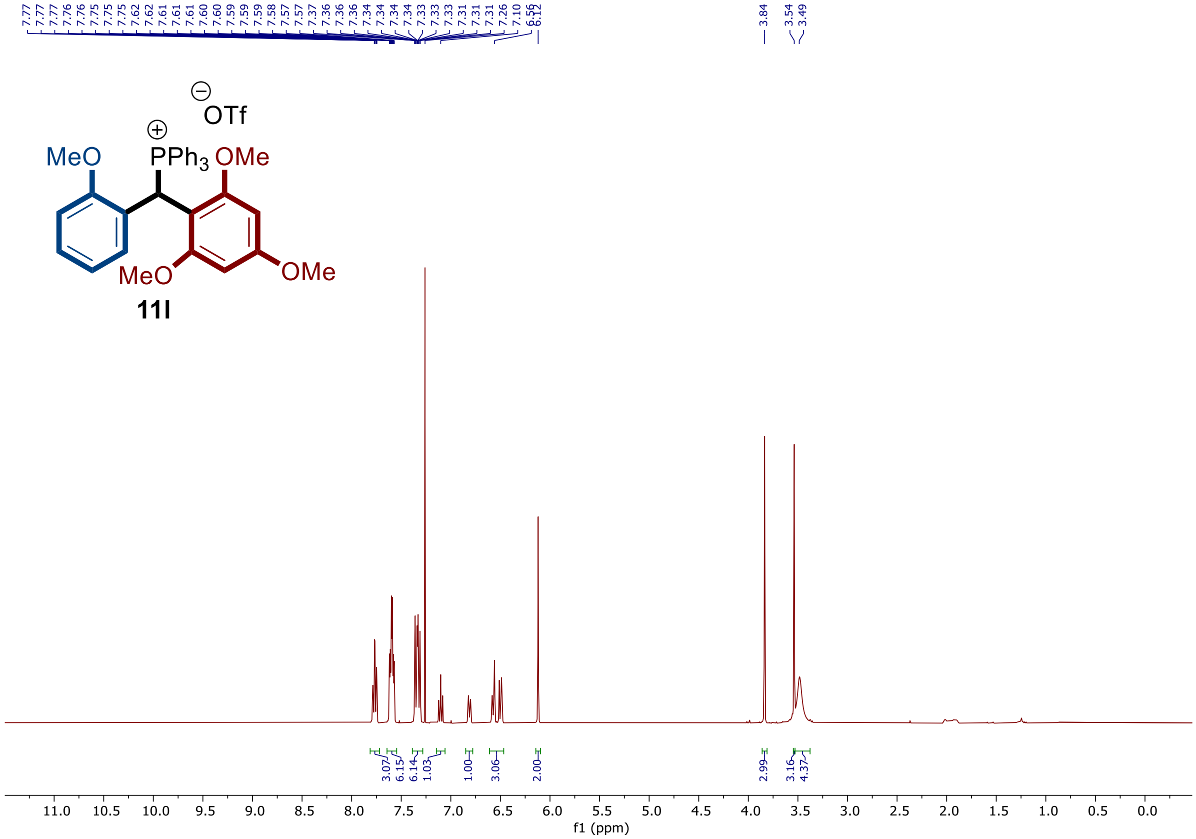

^{1}H NMR (400 MHz, $CDCl_3$) of compound (**11l**)

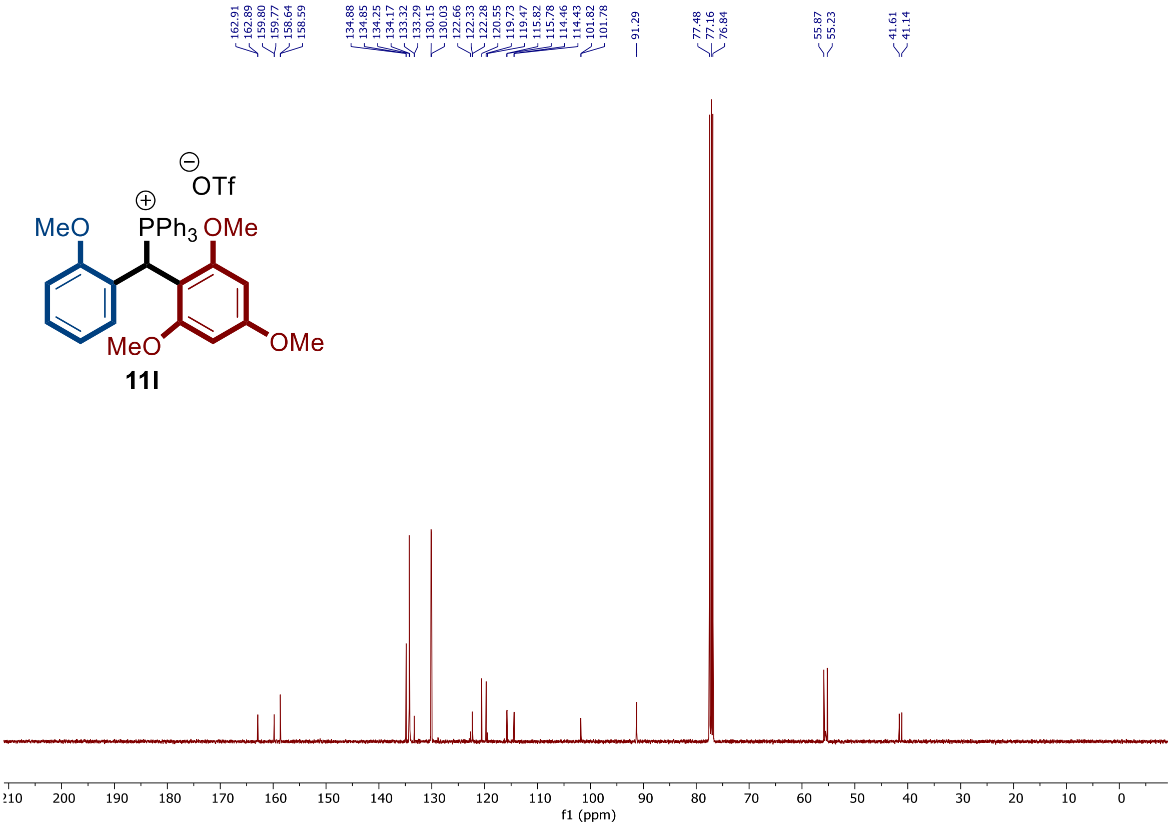

^{13}C NMR (101 MHz, $CDCl_3$) of compound (**11l**)

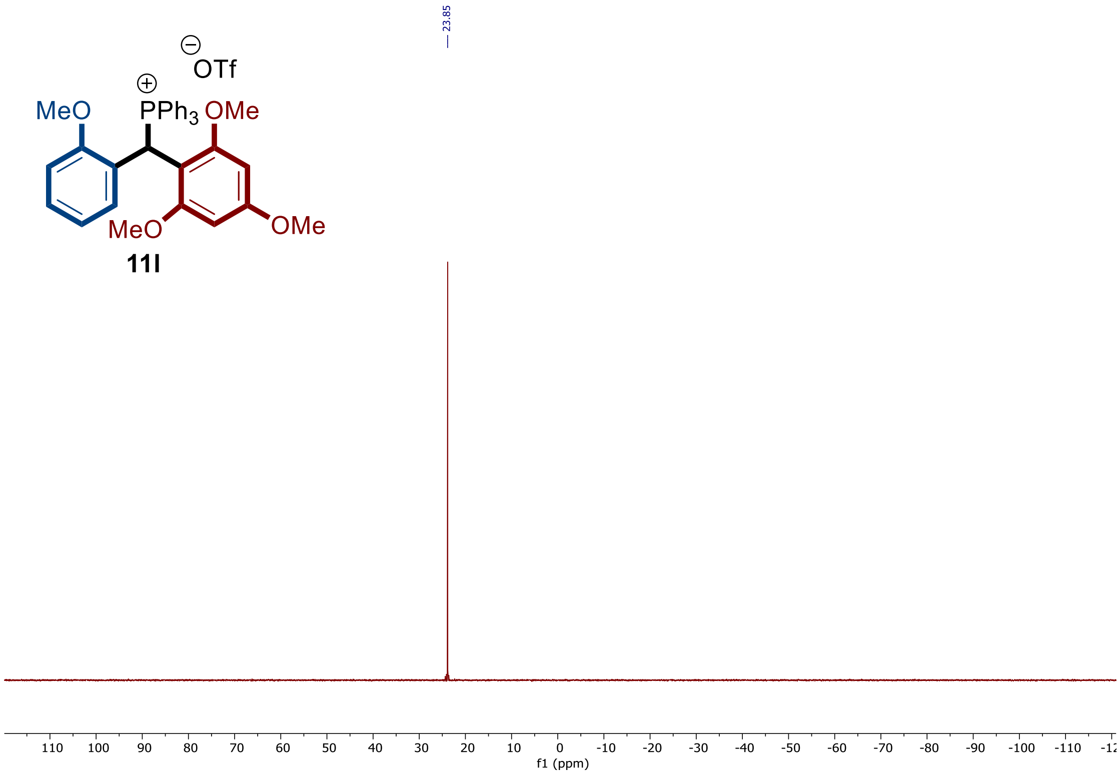

^{31}P NMR (162 MHz, $CDCl_3$) of compound (**11l**)

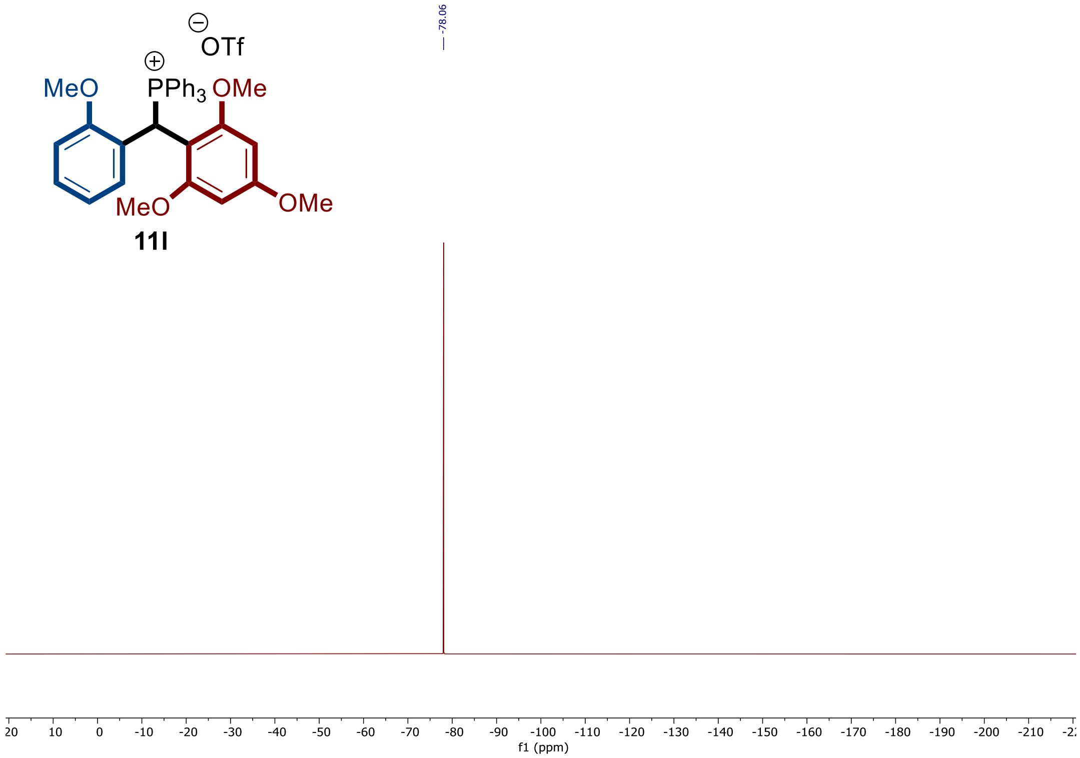

^{19}F NMR (376 MHz, $CDCl_3$) of compound (**11l**)

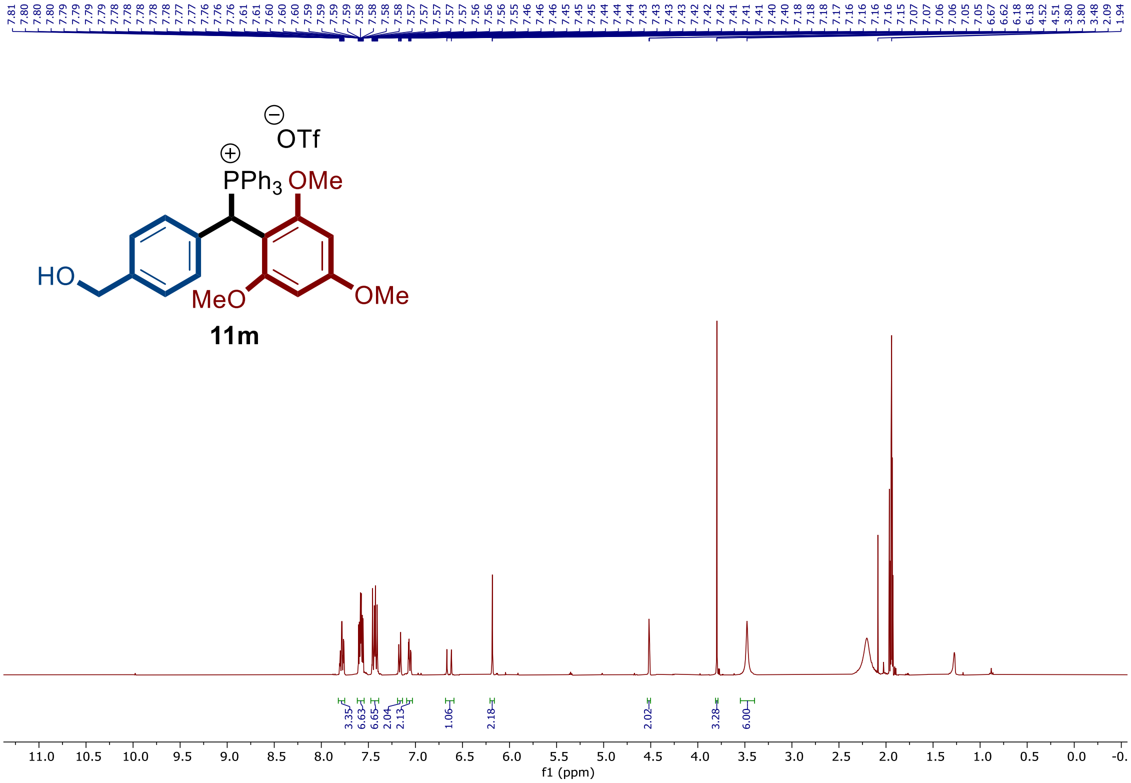

^{1}H NMR (400 MHz, CD_3CN) of compound (**11m**)

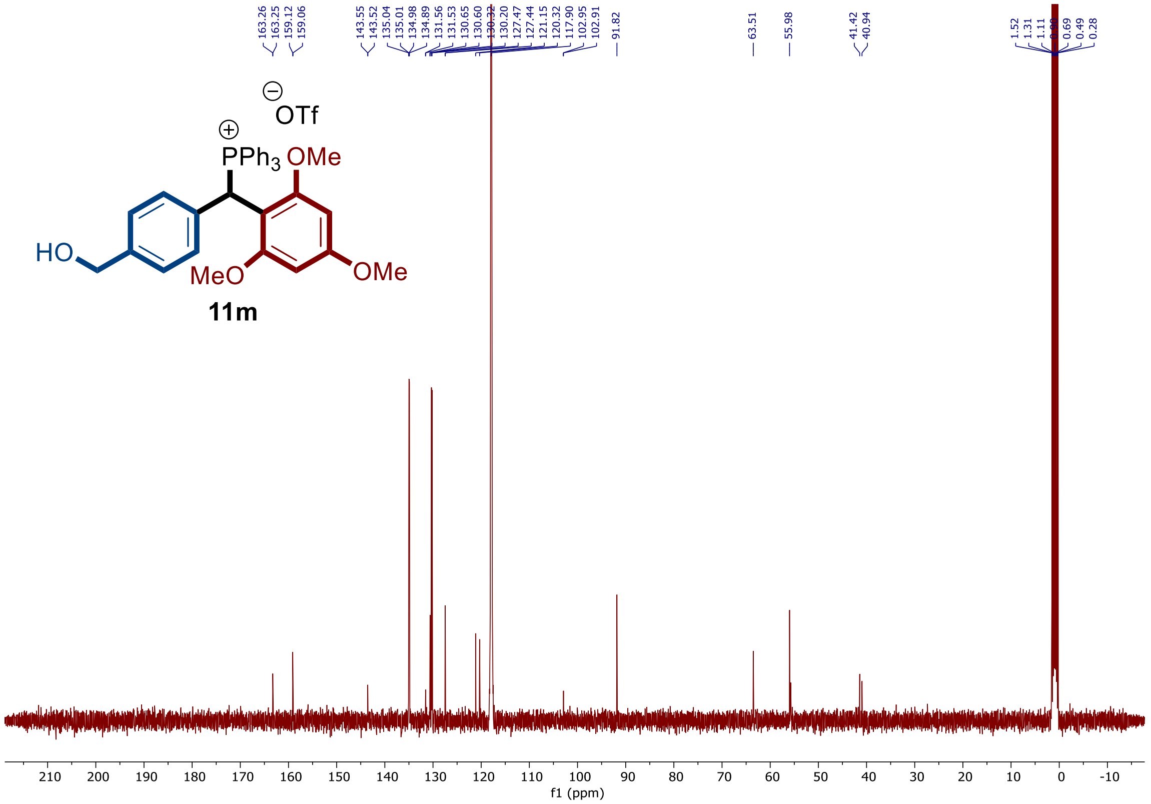

^{13}C NMR (101 MHz, CD_3CN) of compound (**11m**)

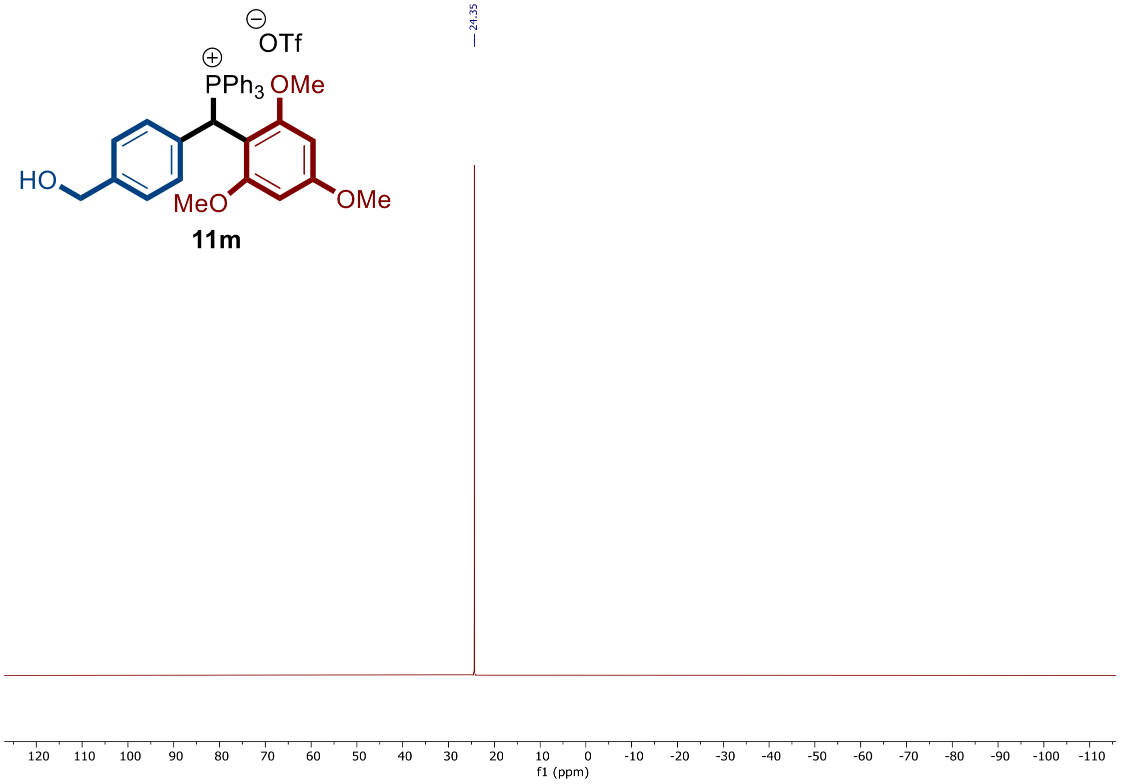

^{31}P NMR (162 MHz, CD_3CN) of compound (**11m**)

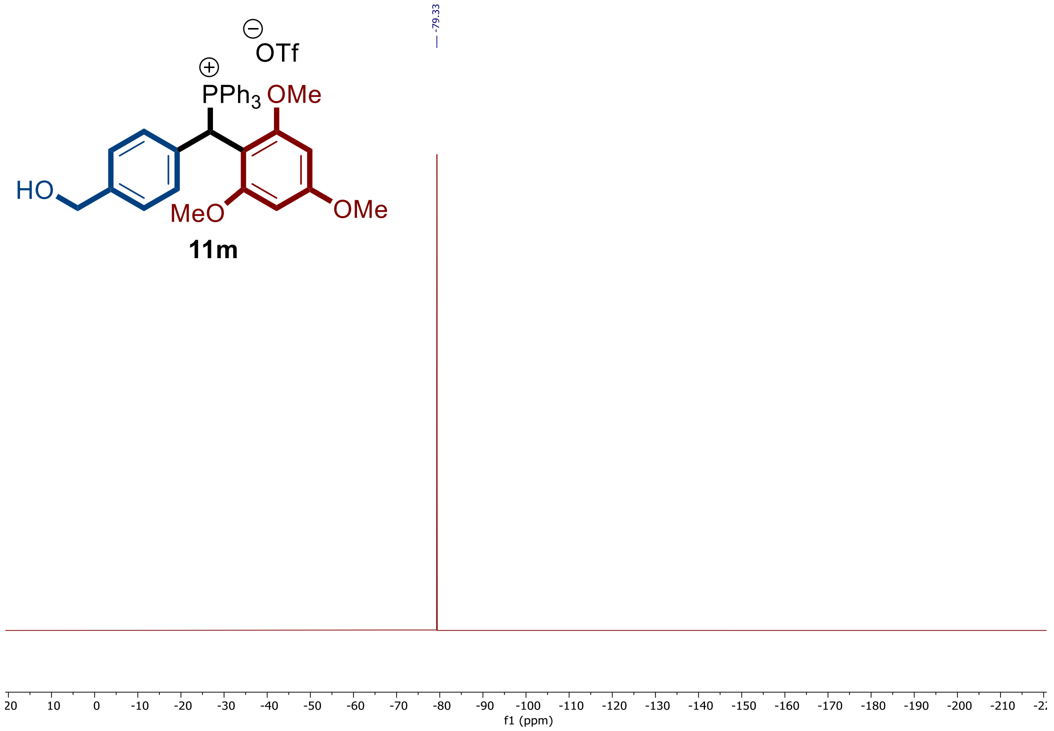

^{19}F NMR (376 MHz, CD_3CN) of compound (**11m**)

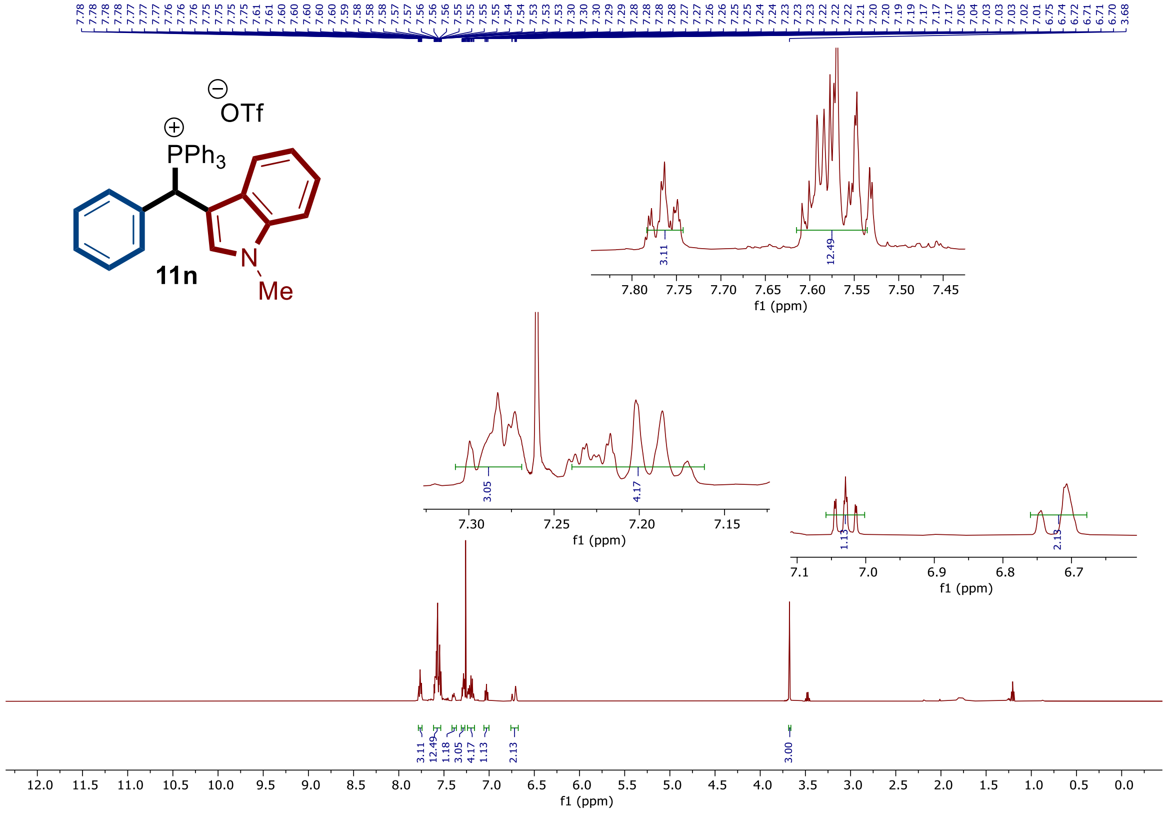

^{1}H NMR (400 MHz, $CDCl_3$) of compound (**11n**)

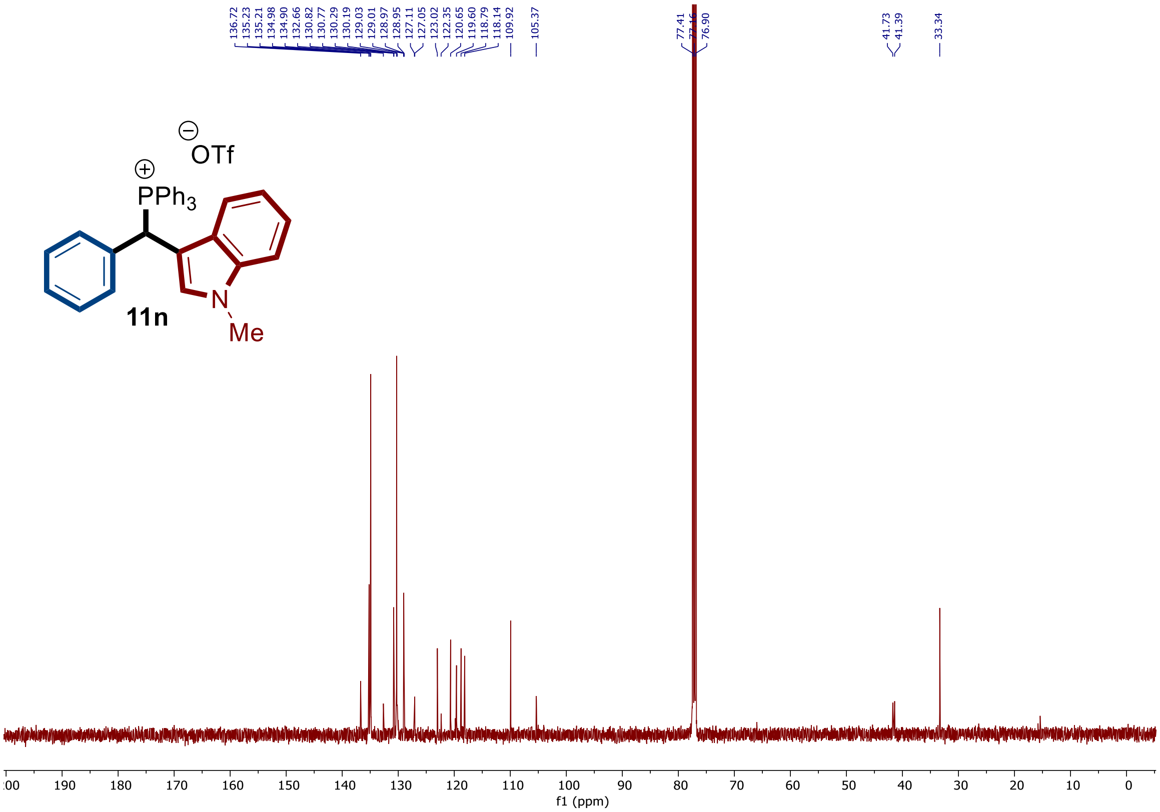

^{13}C NMR (101 MHz, $CDCl_3$) of compound (**11n**)

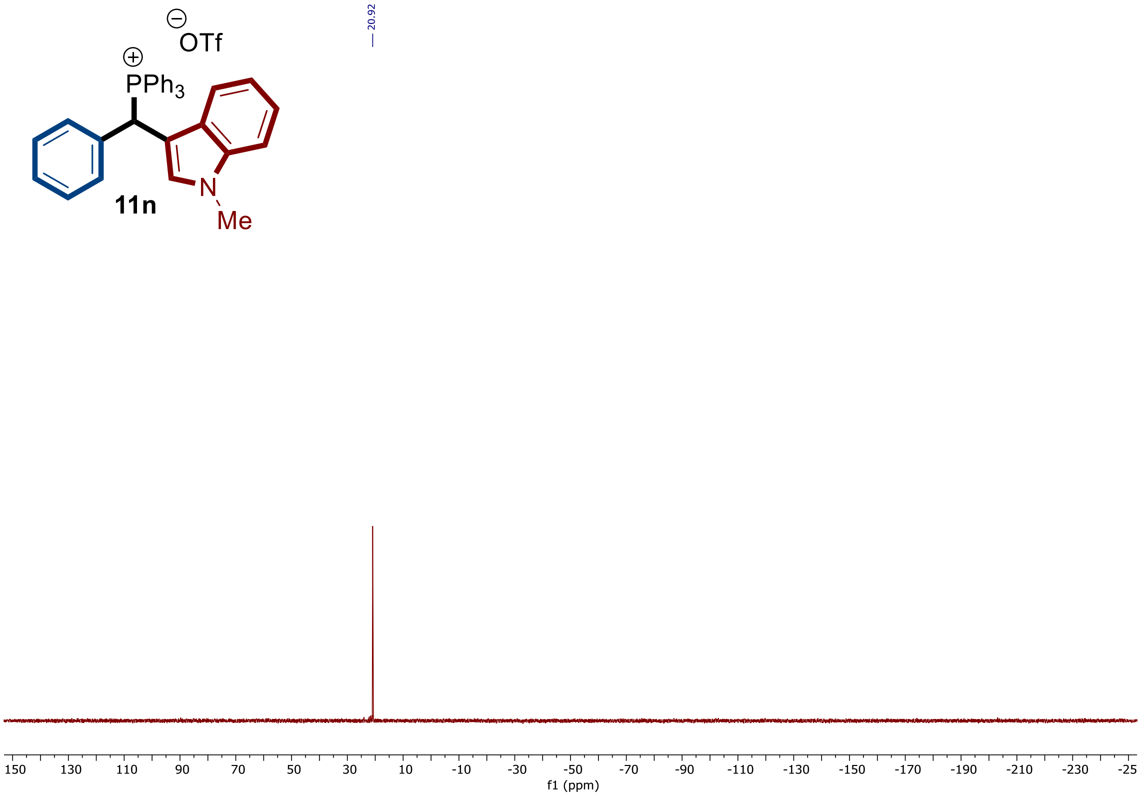

^{31}P NMR (162 MHz, $CDCl_3$) of compound (**11n**)

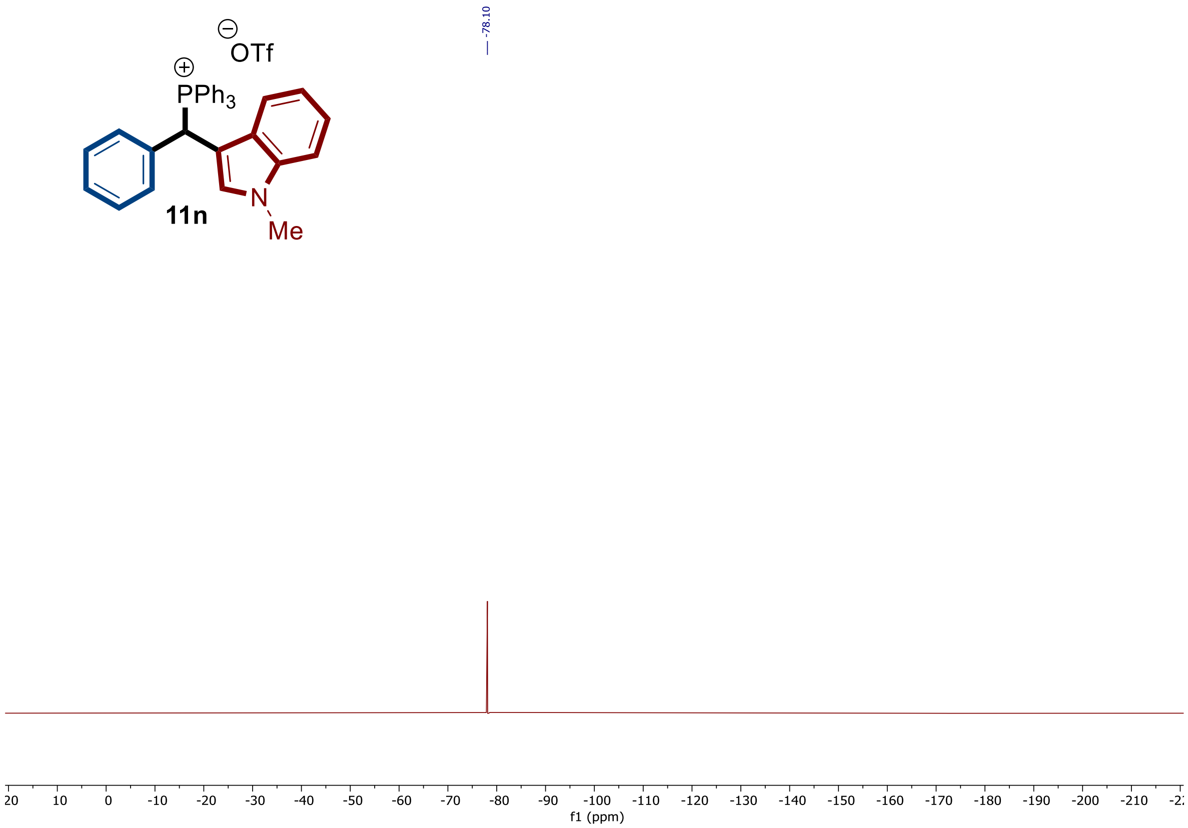

^{19}F NMR (376 MHz, $CDCl_3$) of compound (**11n**)

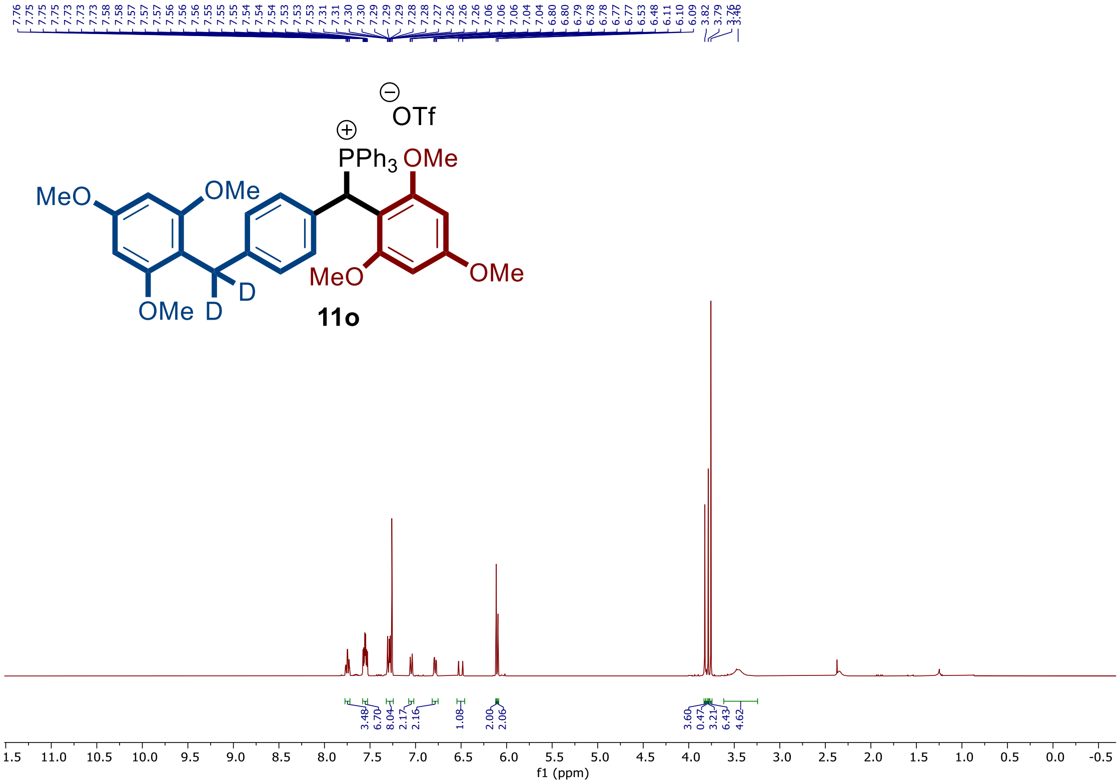

^{1}H NMR (400 MHz, $CDCl_3$) of compound (**11o**)

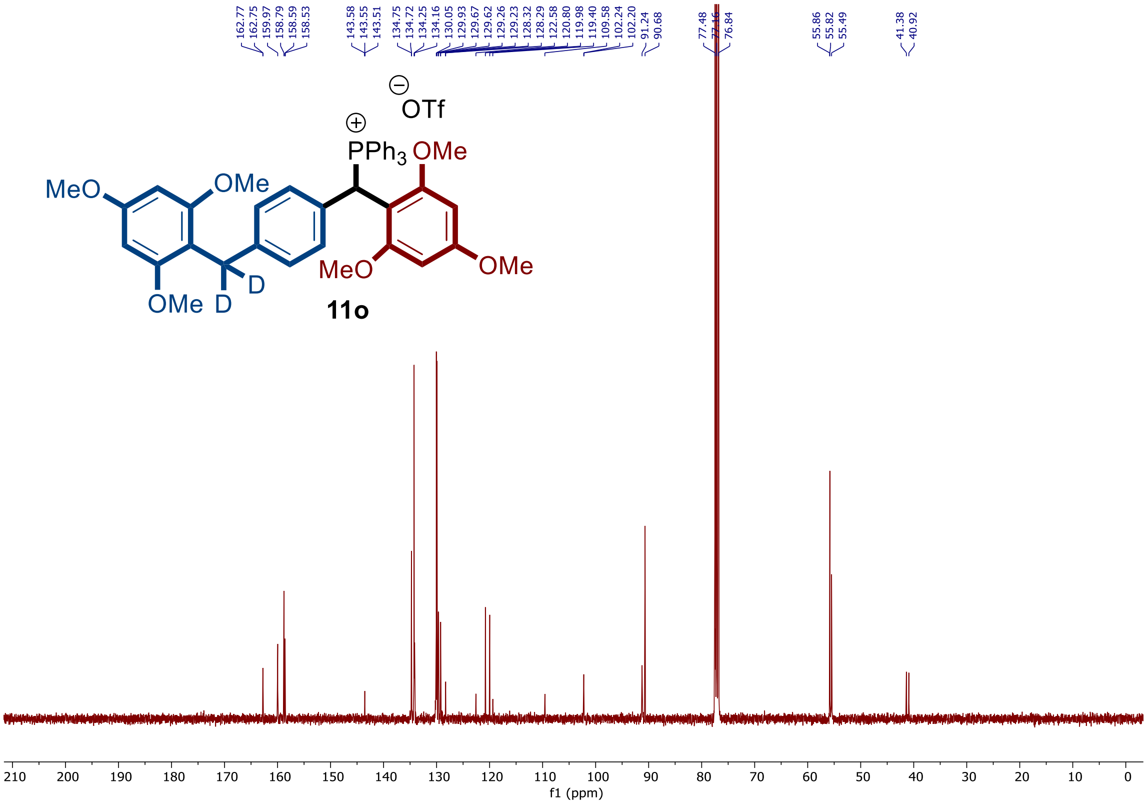

^{13}C NMR (101 MHz, $CDCl_3$) of compound (**11o**)

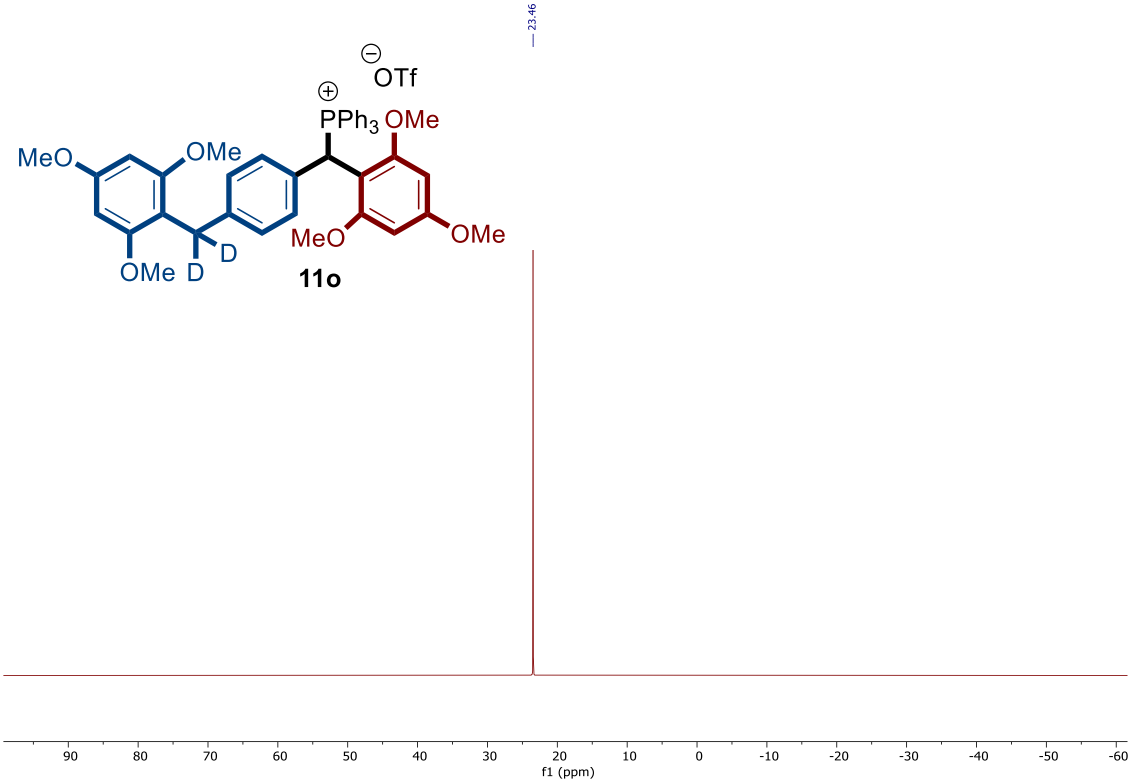

^{31}P NMR (162 MHz, $CDCl_3$) of compound (**11o**)

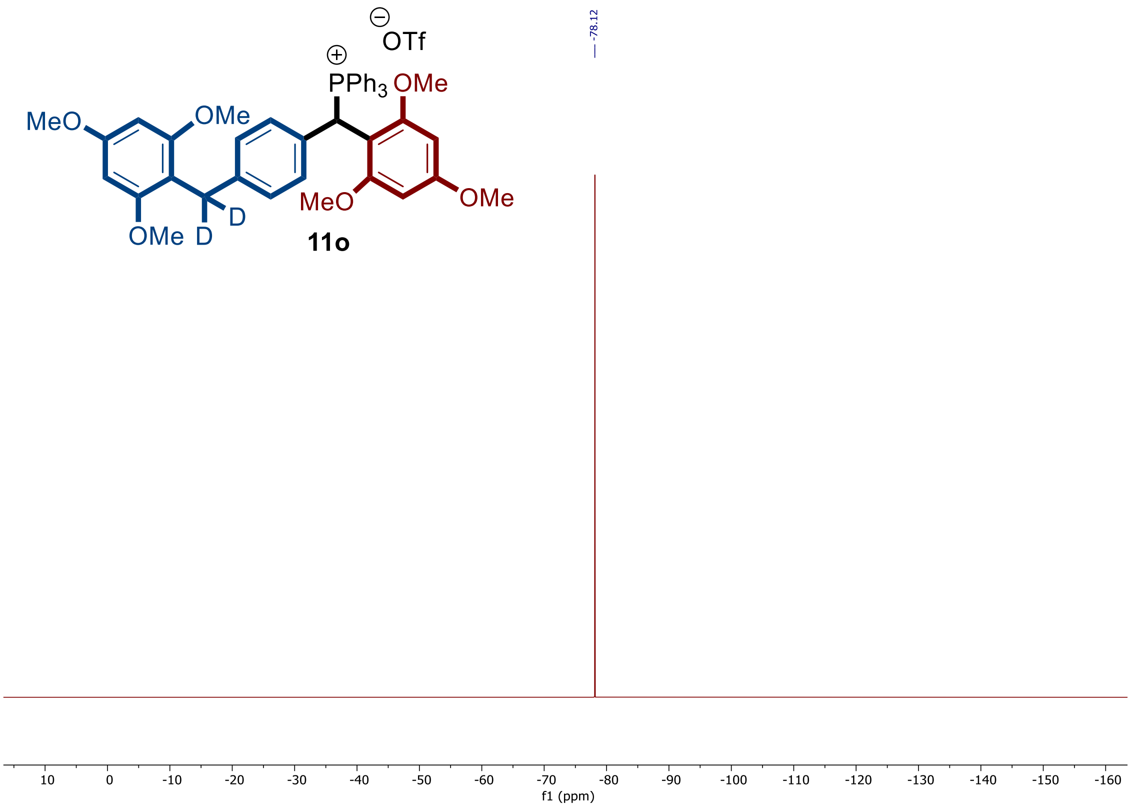

^{19}F NMR (376 MHz, $CDCl_3$) of compound (**11o**)

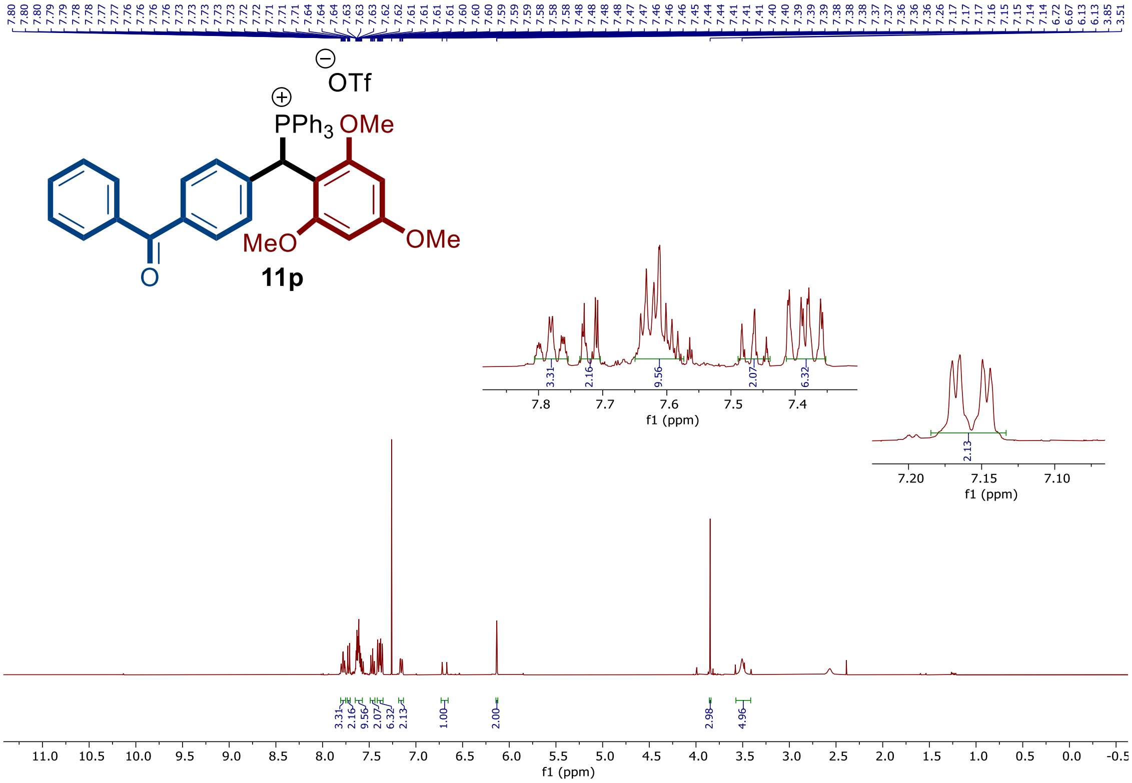

^{1}H NMR (400 MHz, $CDCl_3$) of compound (**11p**)

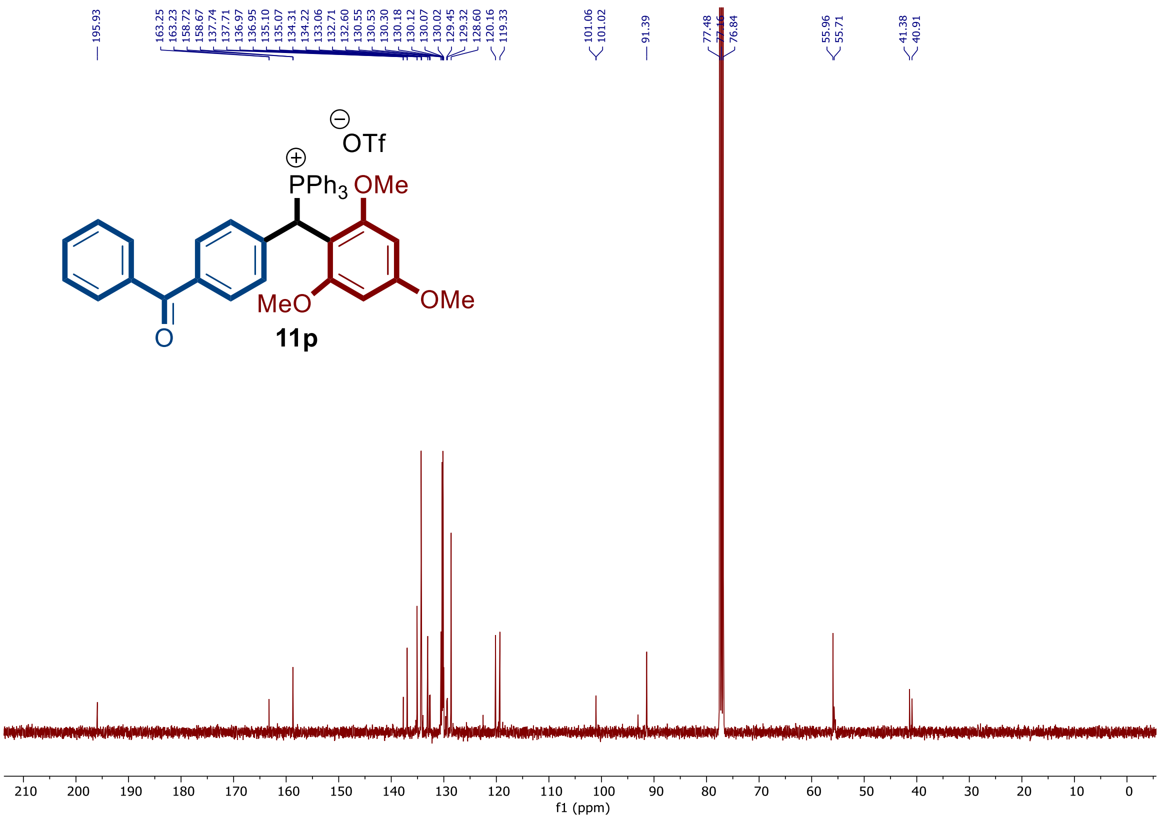

^{13}C NMR (101 MHz, $CDCl_3$) of compound (**11p**)

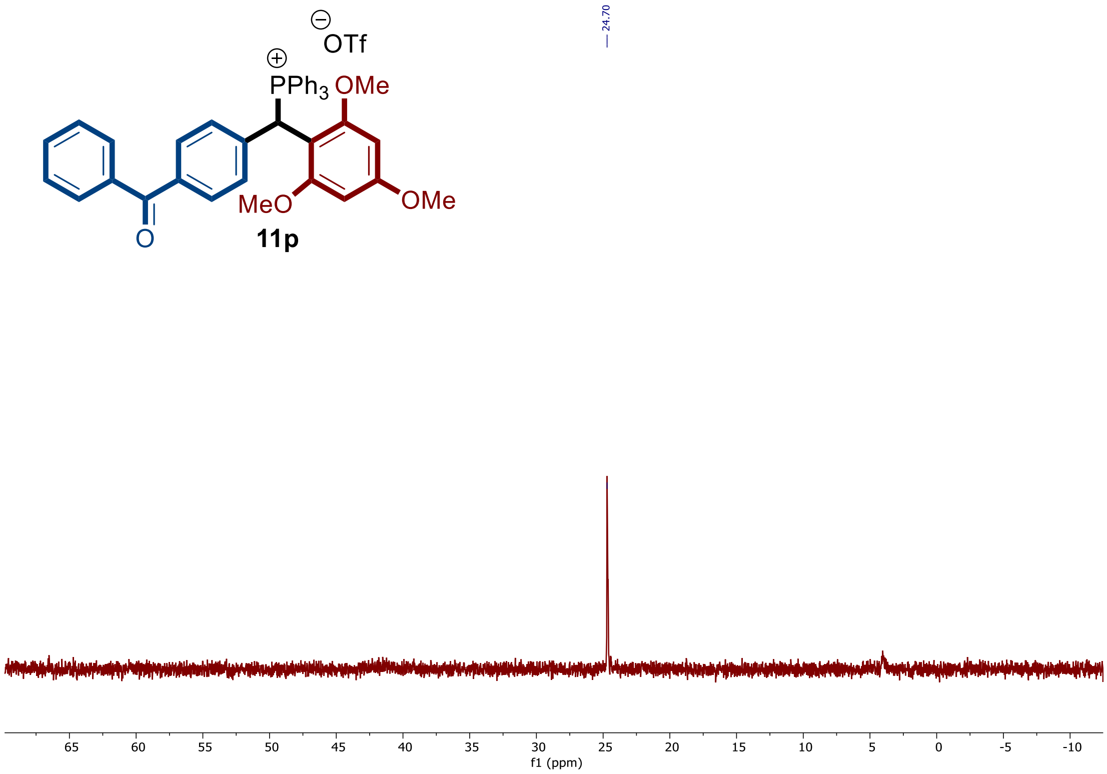

^{31}P NMR (162 MHz, $CDCl_3$) of compound (**11p**)

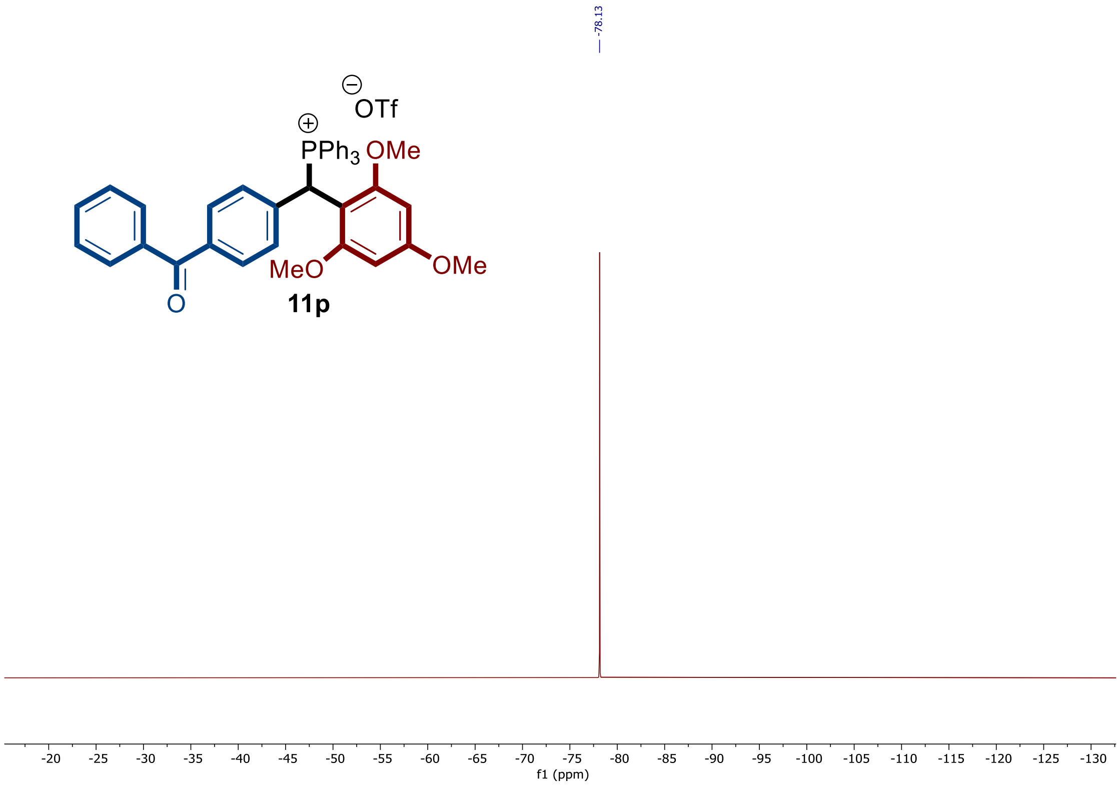

^{19}F NMR (376 MHz, $CDCl_3$) of compound (**11p**)

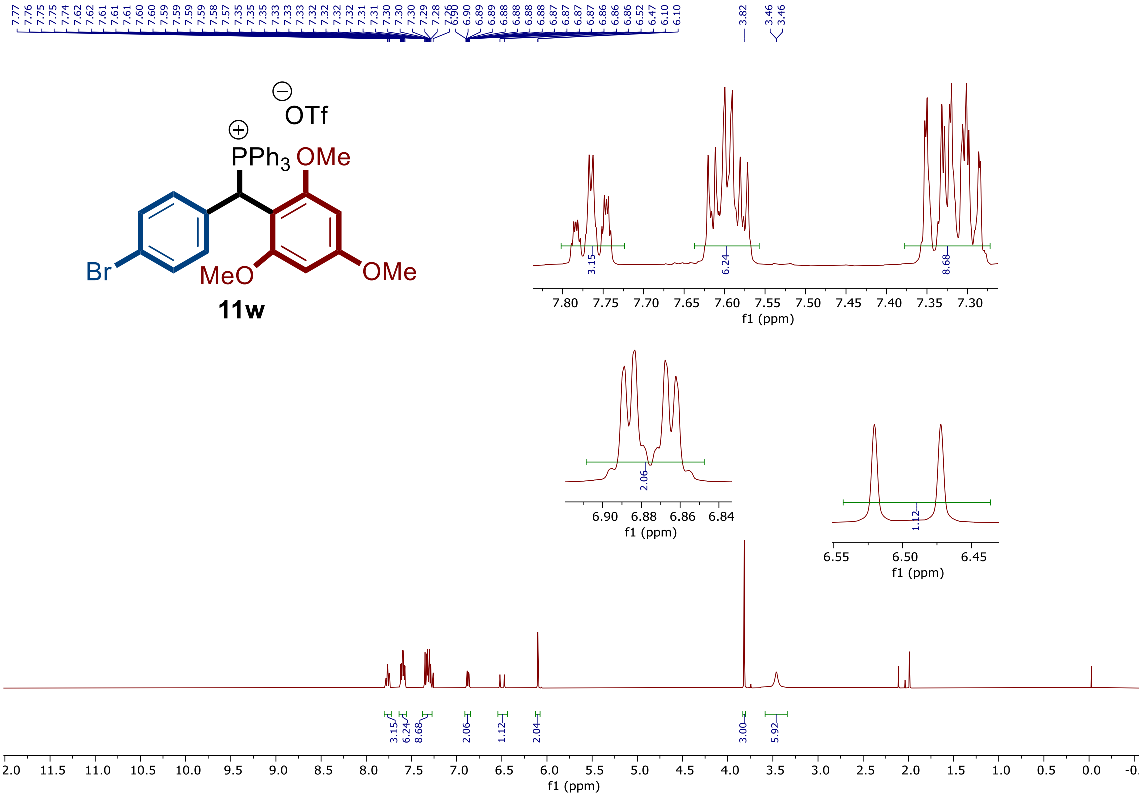

^{1}H NMR (400 MHz, $CDCl_3$) of compound (**11w**)

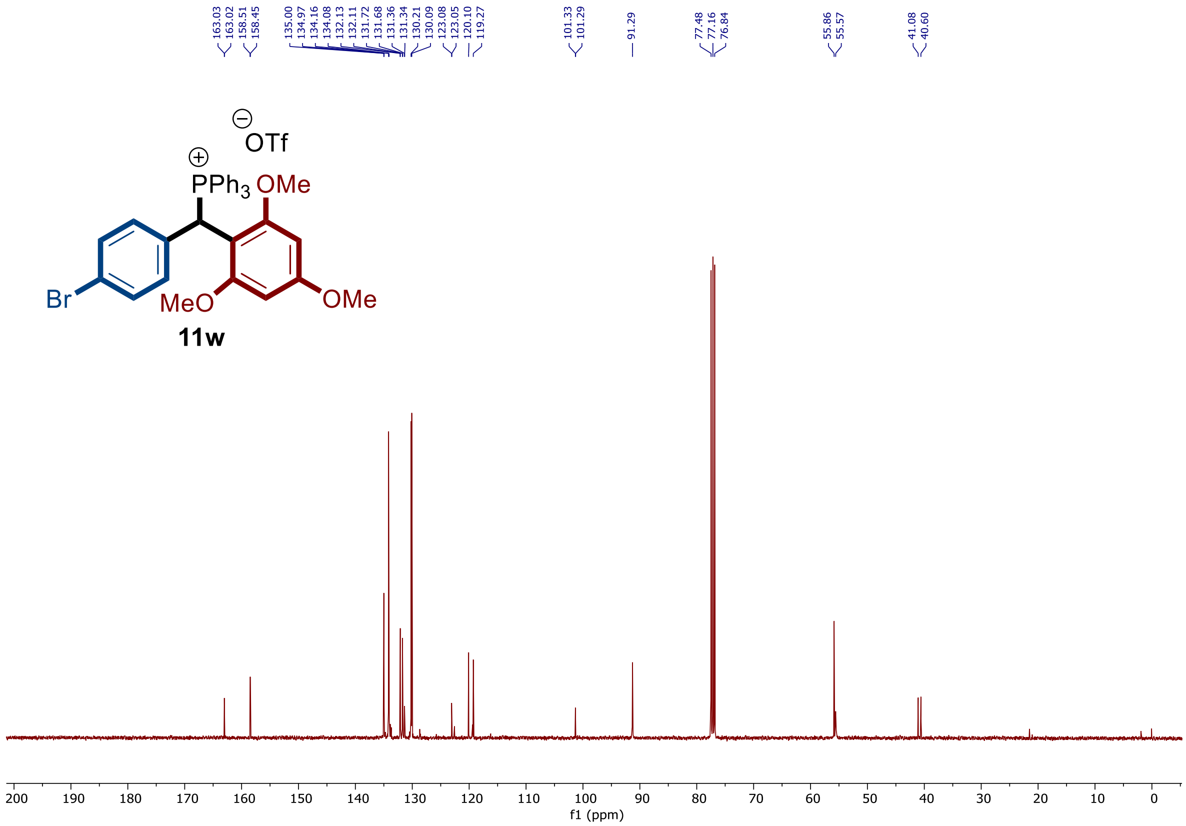

^{13}C NMR (101 MHz, $CDCl_3$) of compound (**11w**)

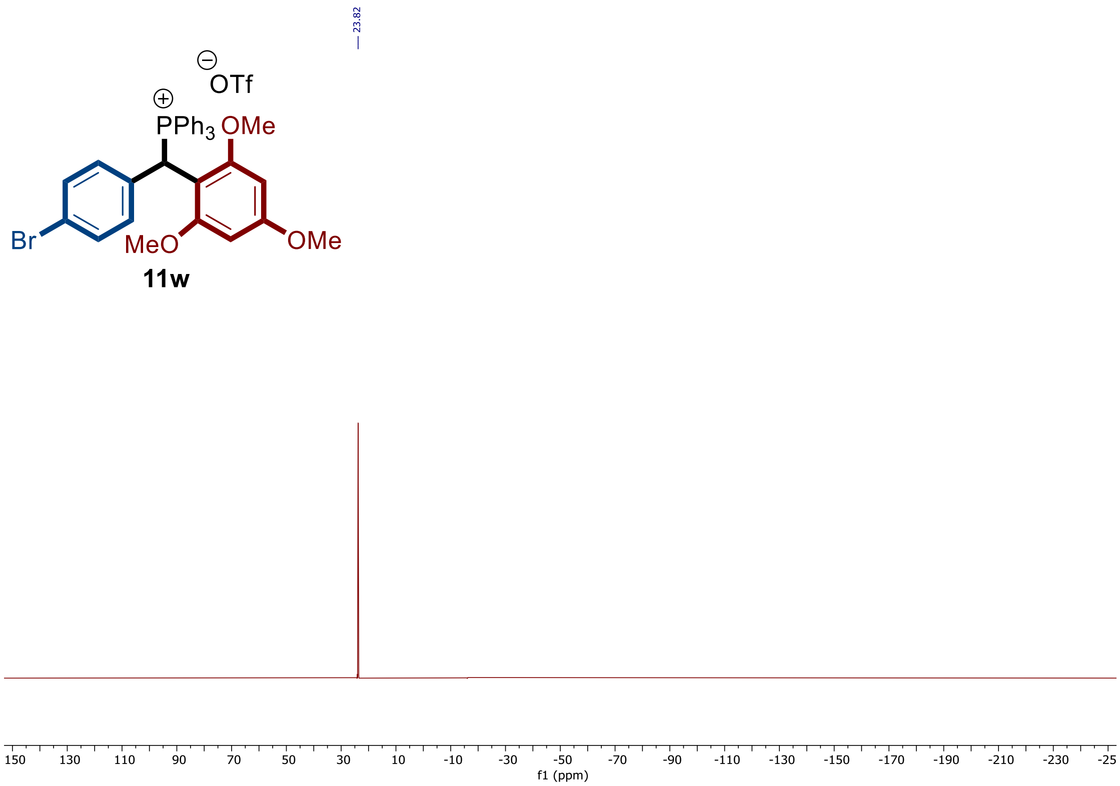

^{31}P NMR (162 MHz, $CDCl_3$) of compound (**11w**)

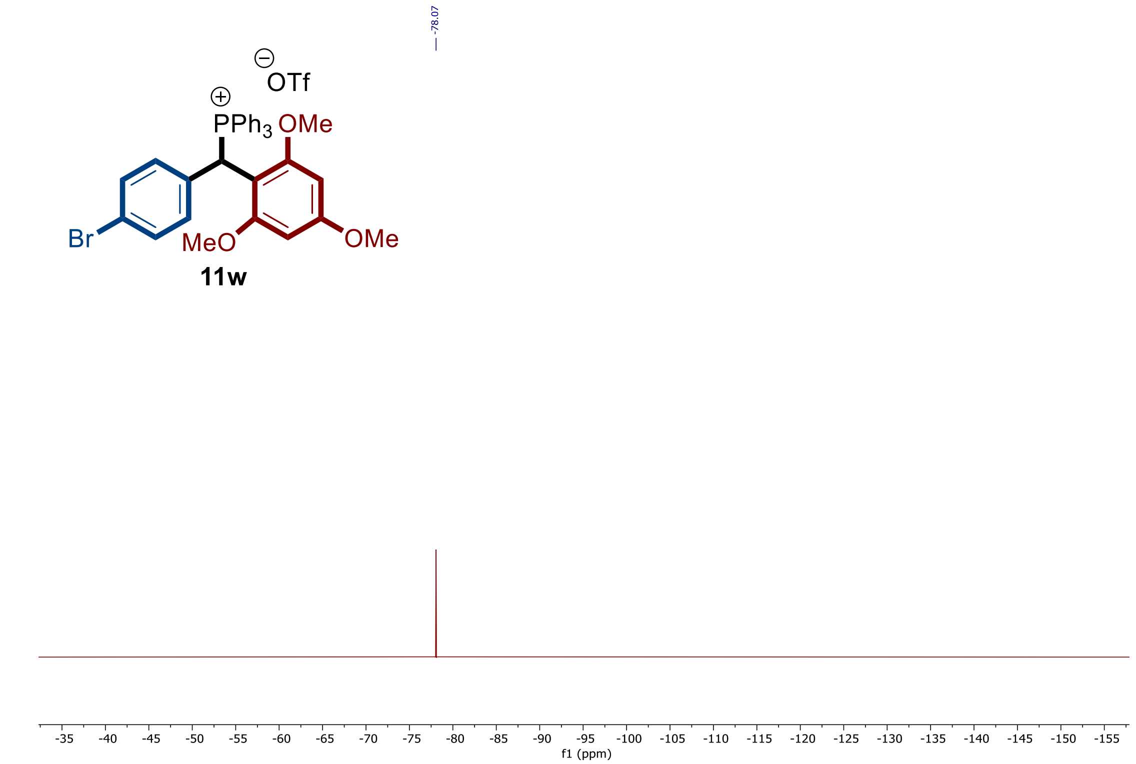

^{19}F NMR (376 MHz, $CDCl_3$) of compound (**11w**)

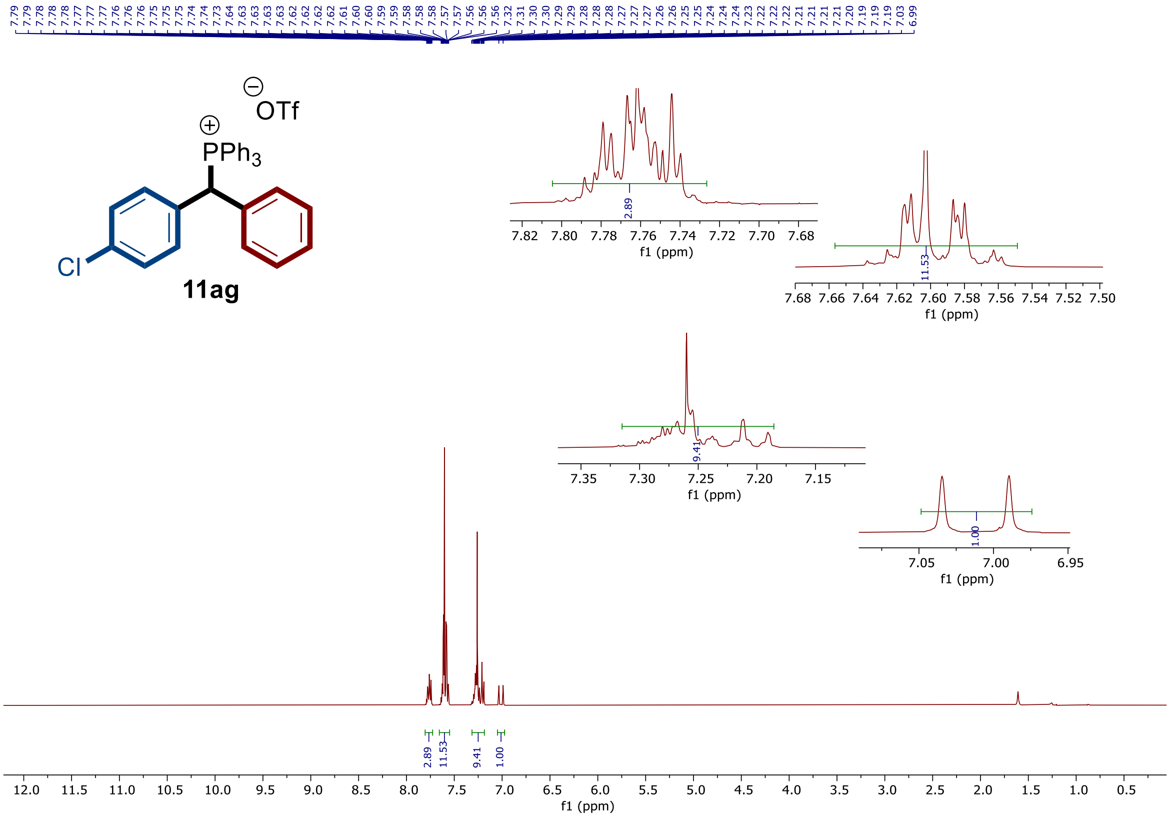

^{1}H NMR (400 MHz, $CDCl_3$) of compound (**11ag**)

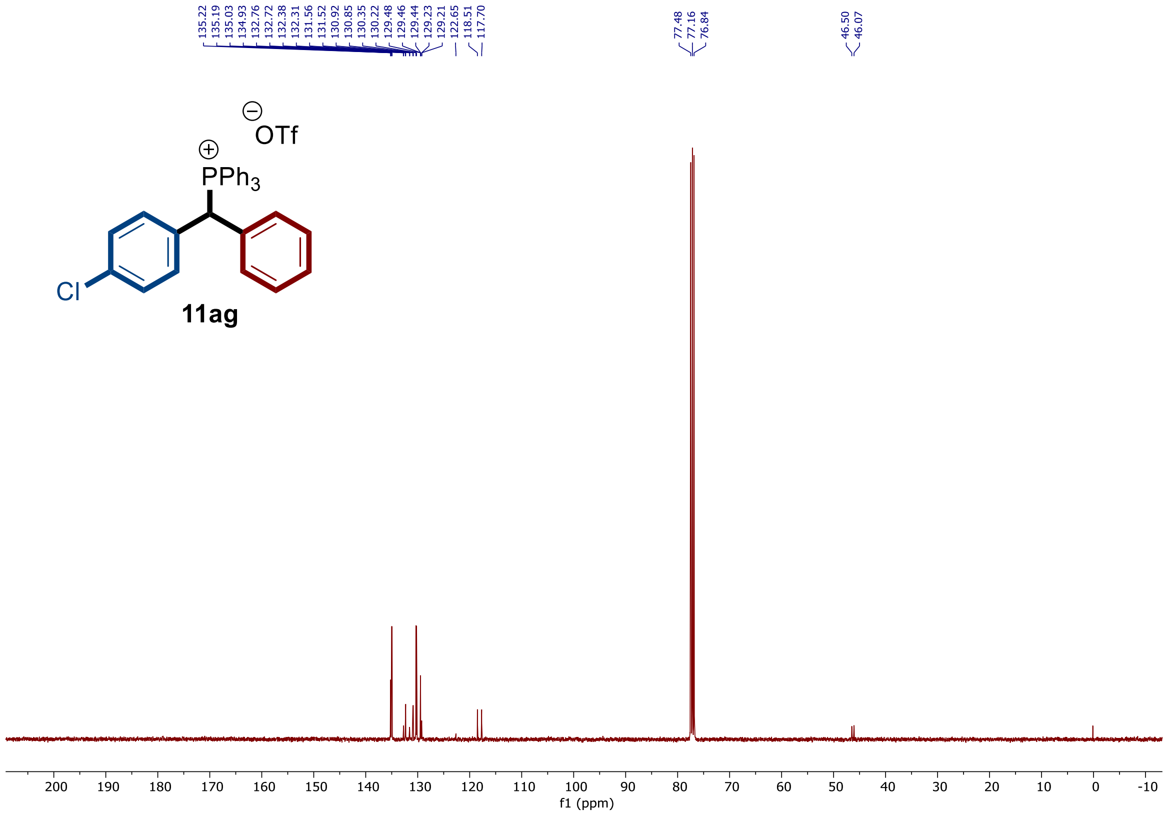

^{13}C NMR (101 MHz, $CDCl_3$) of compound (**11ag**)

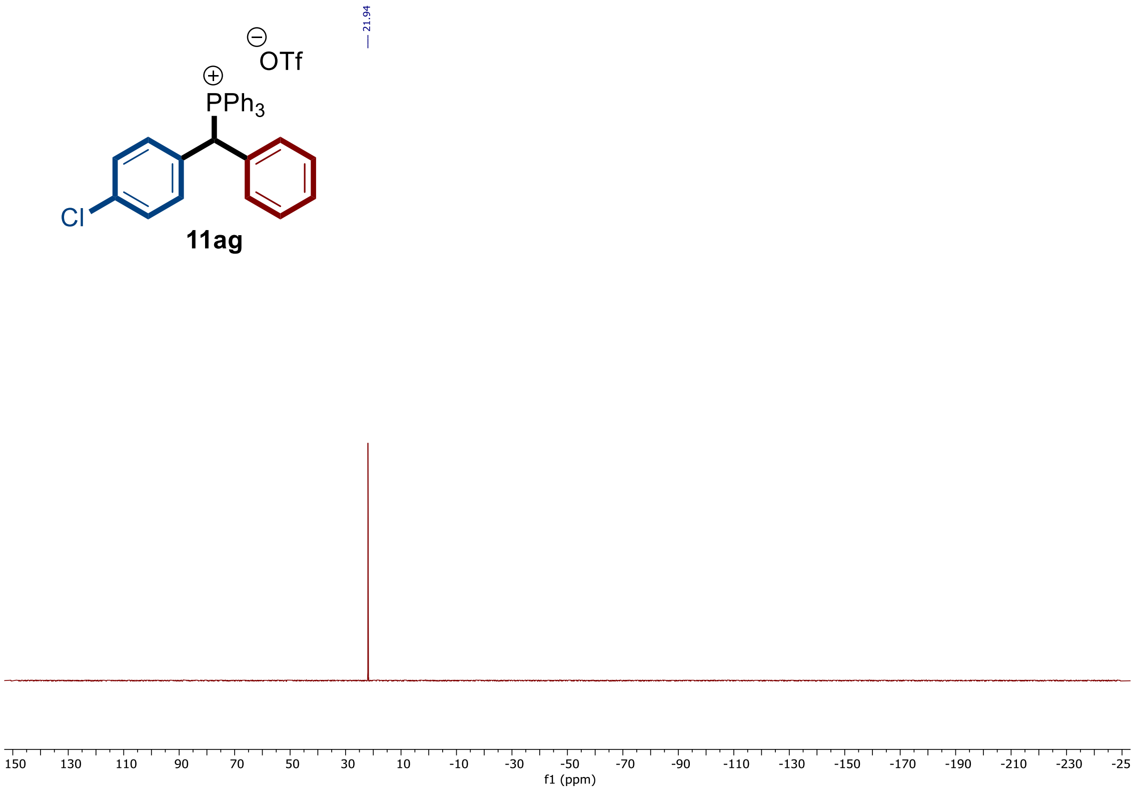

^{31}P NMR (162 MHz, $CDCl_3$) of compound (**11ag**)

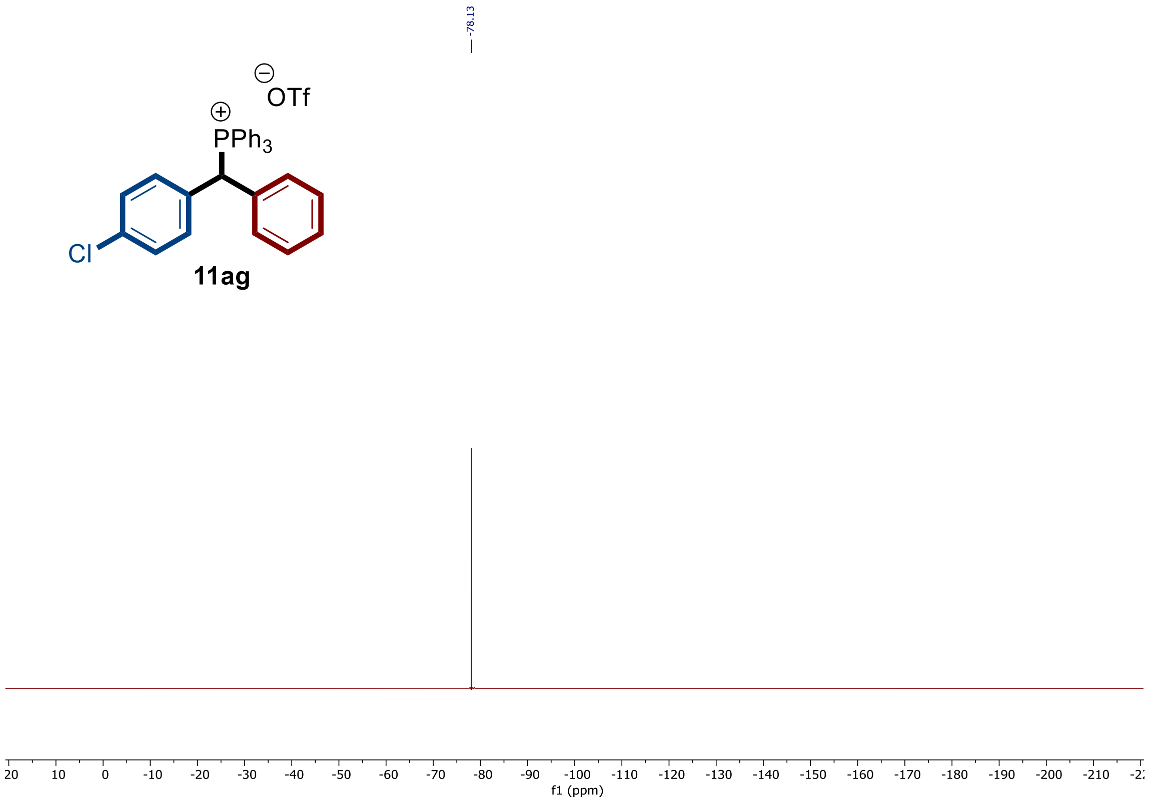

^{19}F NMR (376 MHz, $CDCl_3$) of compound (**11ag**)

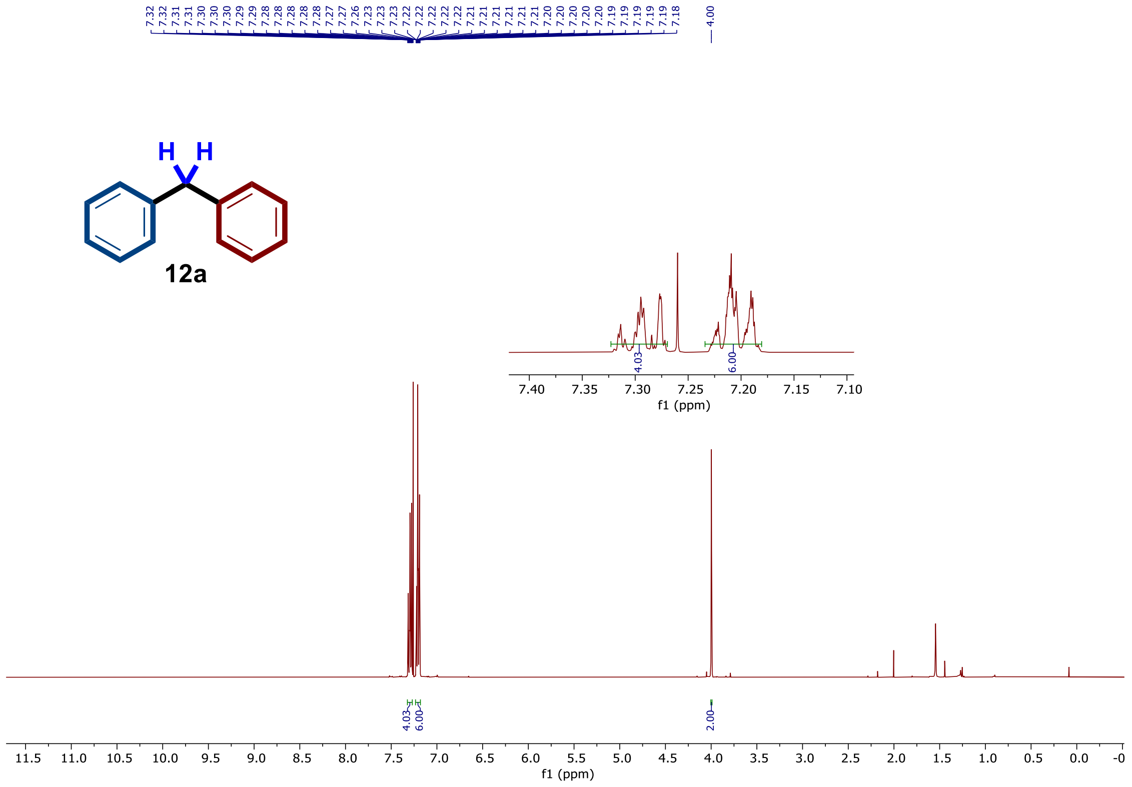

^{1}H NMR (400 MHz, $CDCl_3$) of compound (**12a**)

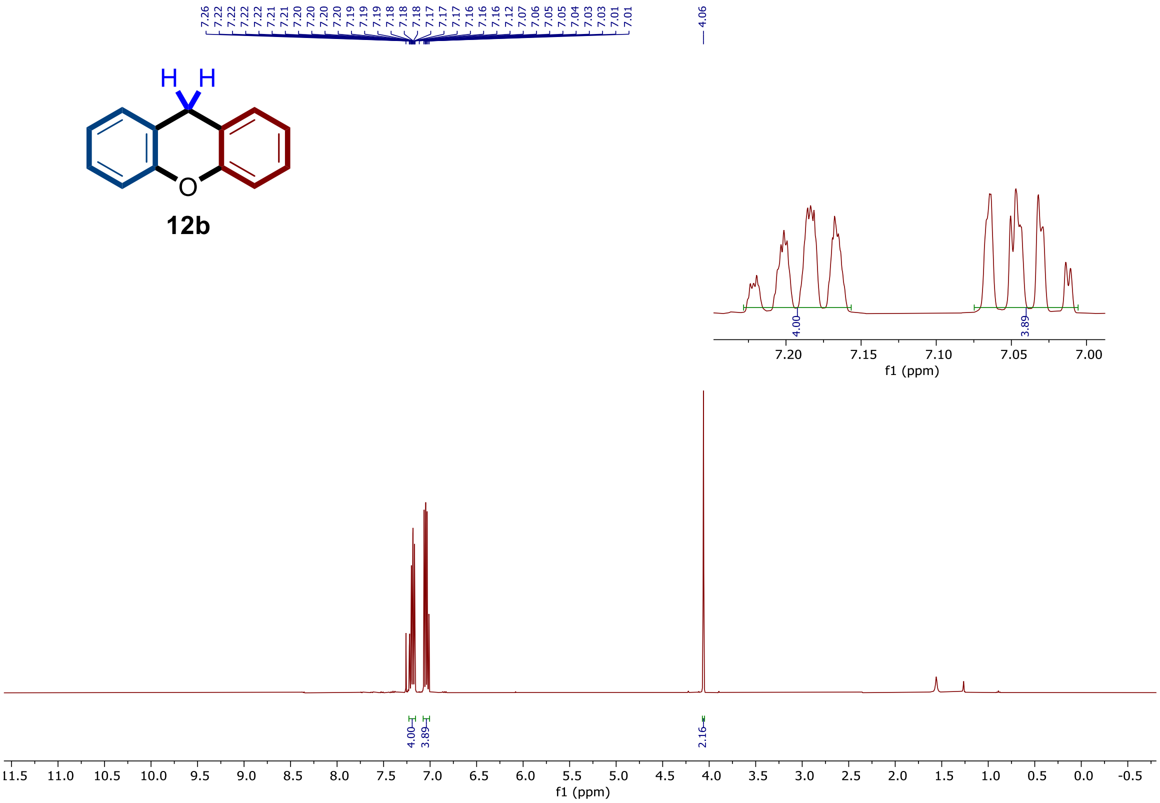

¹H NMR (400 MHz, $CDCl_3$) of compound (**12b**)

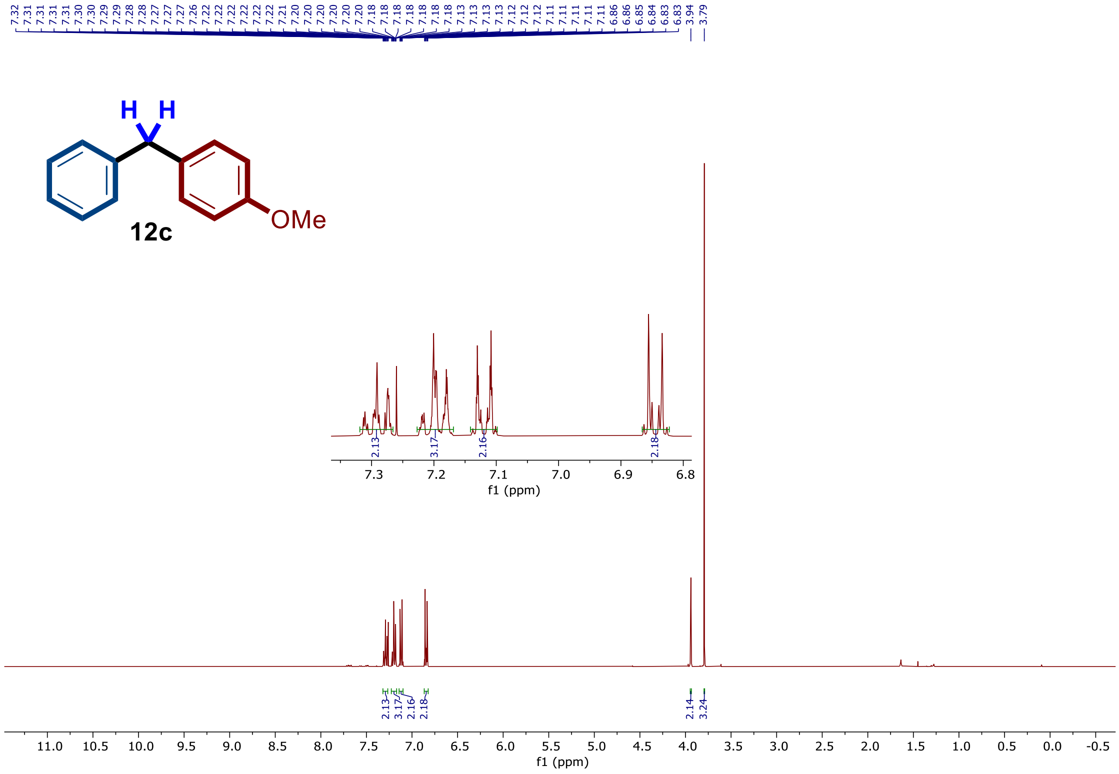

¹H NMR (400 MHz, $CDCl_3$) of compound (**12c**)

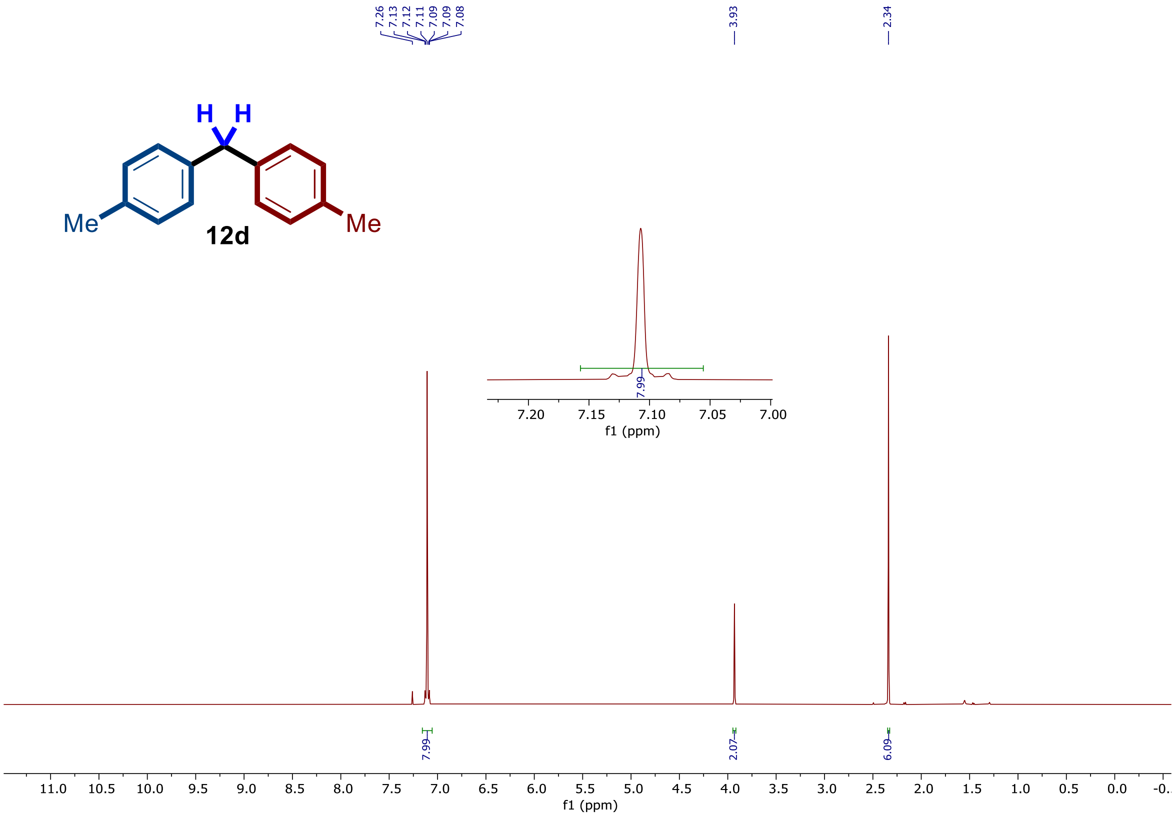

^{1}H NMR (400 MHz, $CDCl_3$) of compound (**12d**)

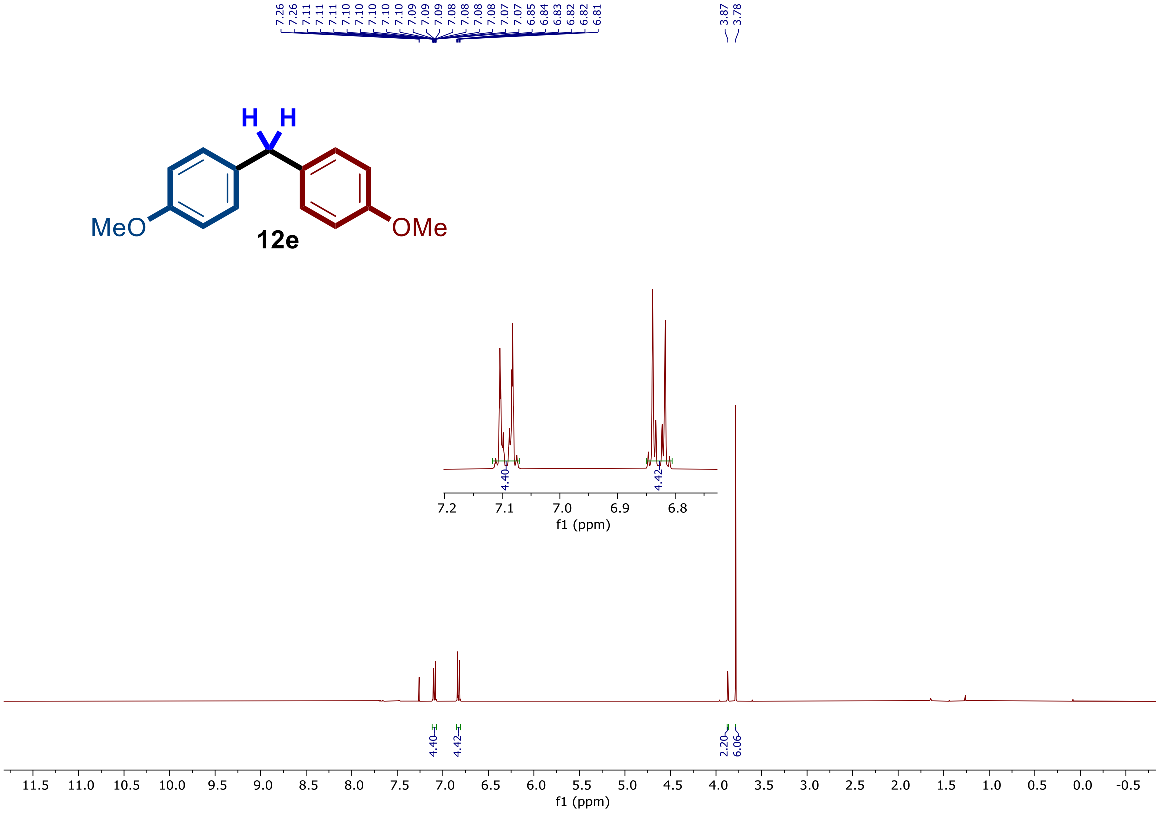

^{1}H NMR (400 MHz, $CDCl_3$) of compound (**12e**)

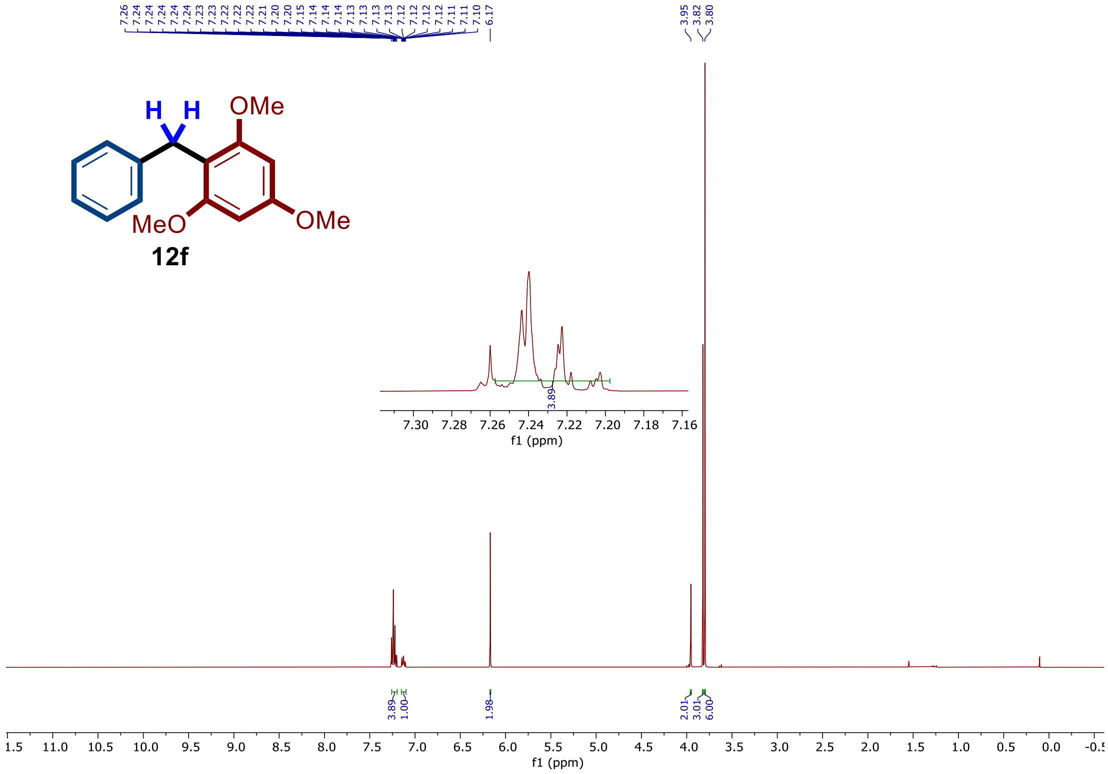

^1H NMR (400 MHz, $CDCl_3$) of compound (**12f**)

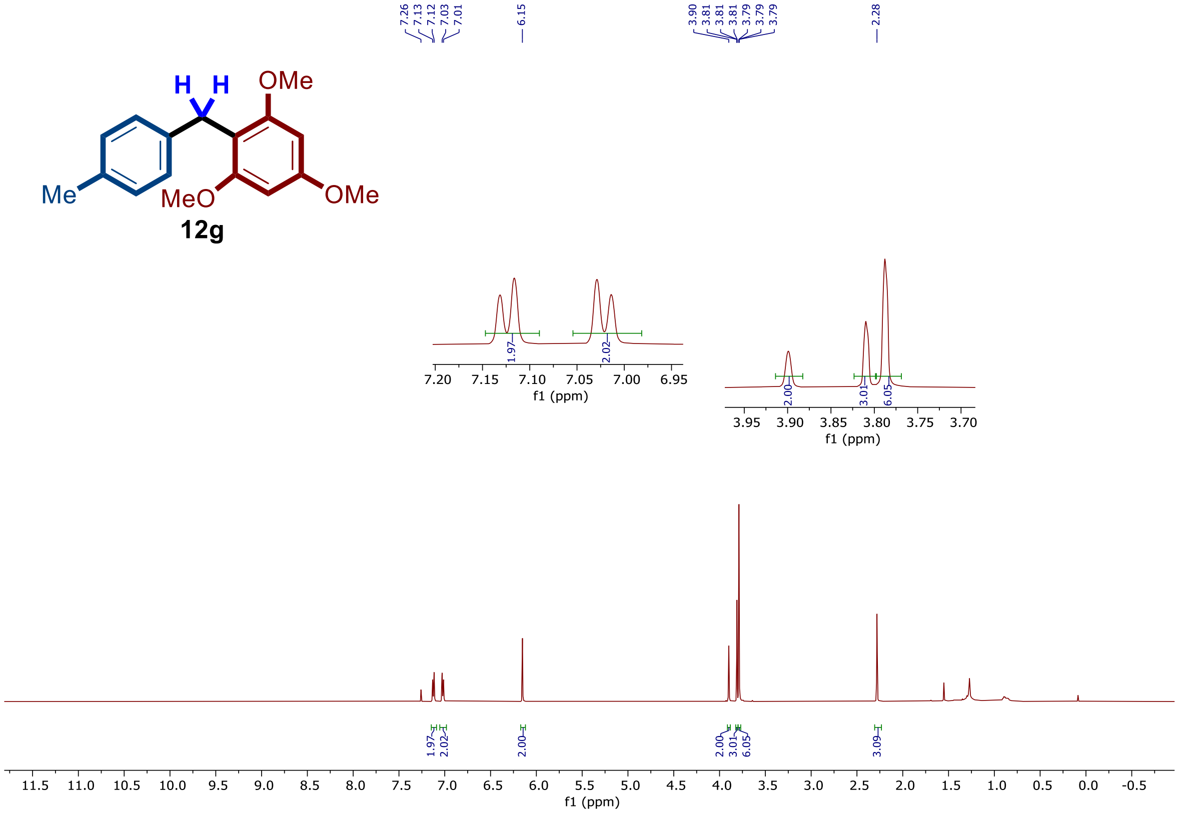

^1H NMR (400 MHz, $CDCl_3$) of compound (**12g**)

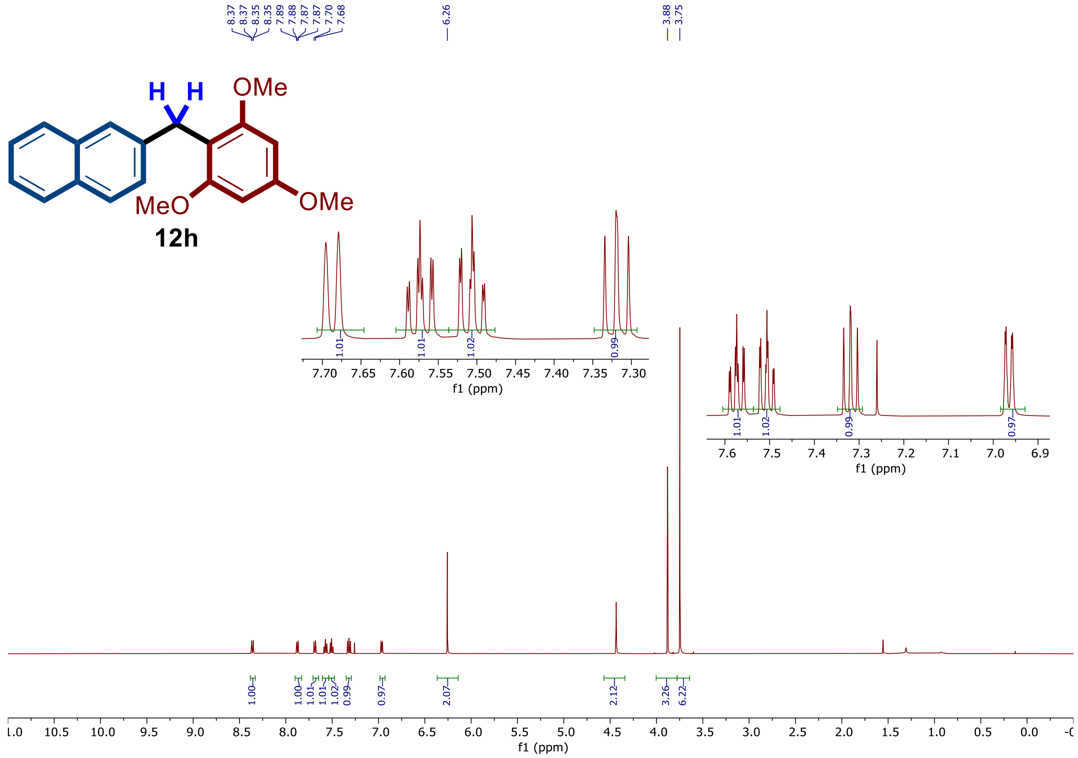

^{1}H NMR (400 MHz, $CDCl_3$) of compound (**12h**)

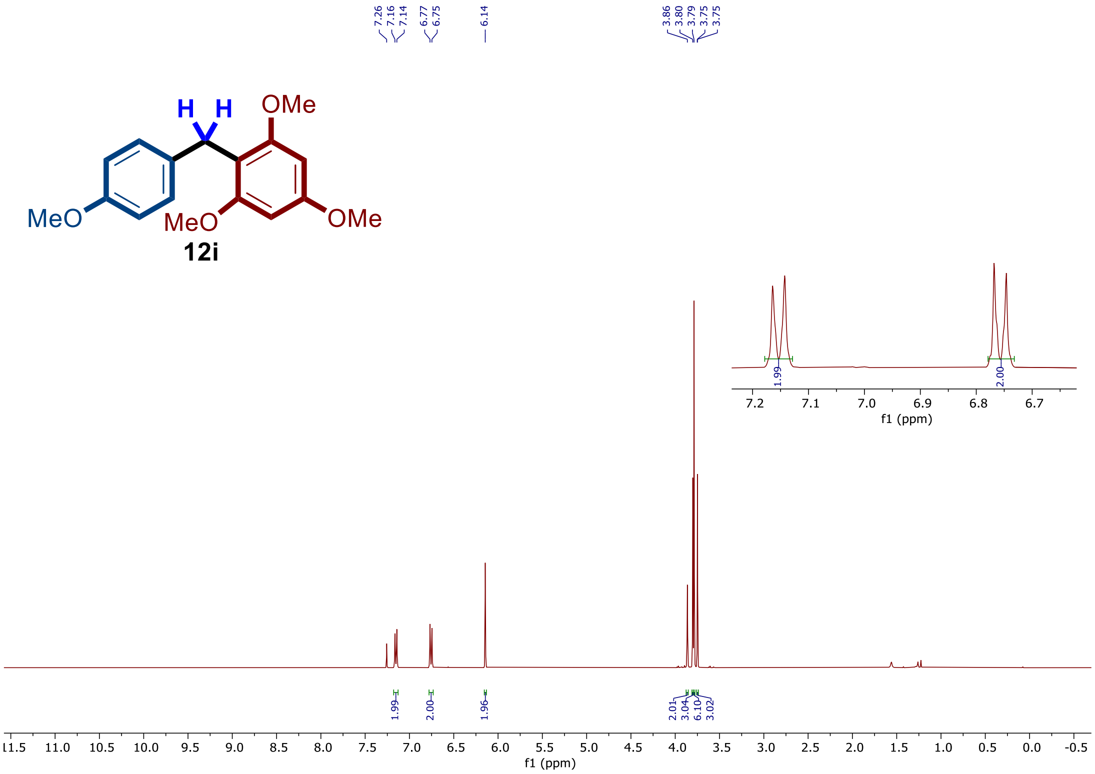

^{1}H NMR (400 MHz, $CDCl_3$) of compound (**12i**)

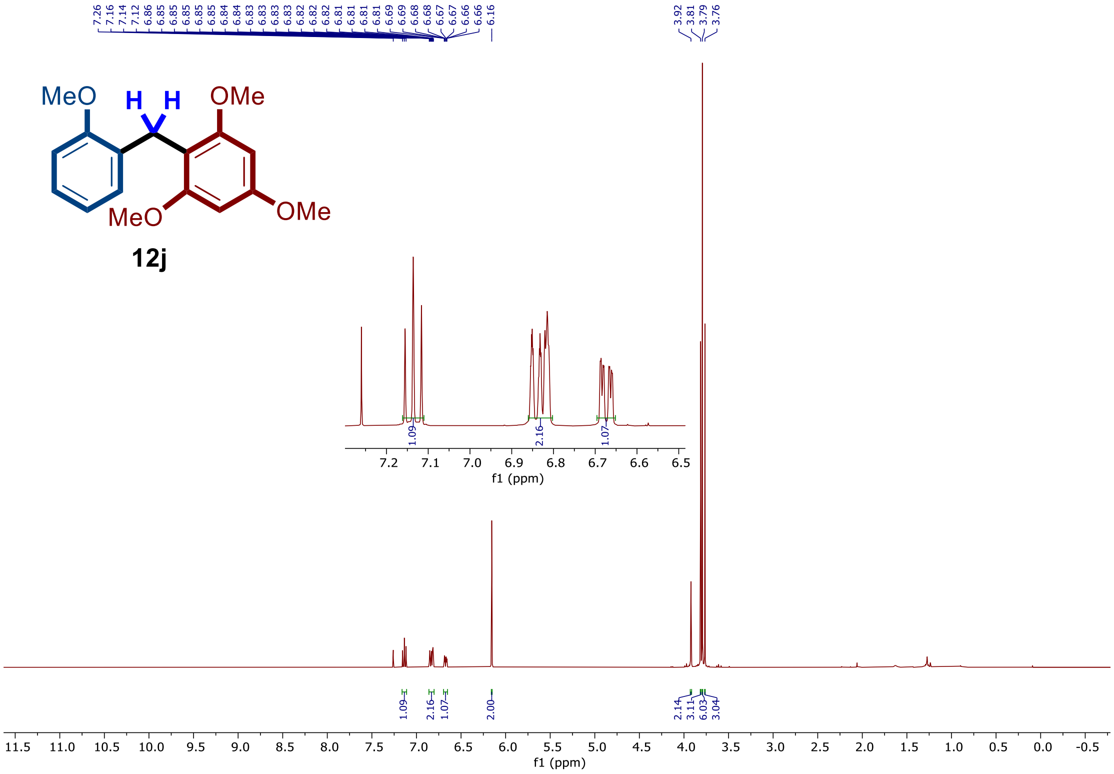

^{1}H NMR (400 MHz, $CDCl_3$) of compound (**12j**)

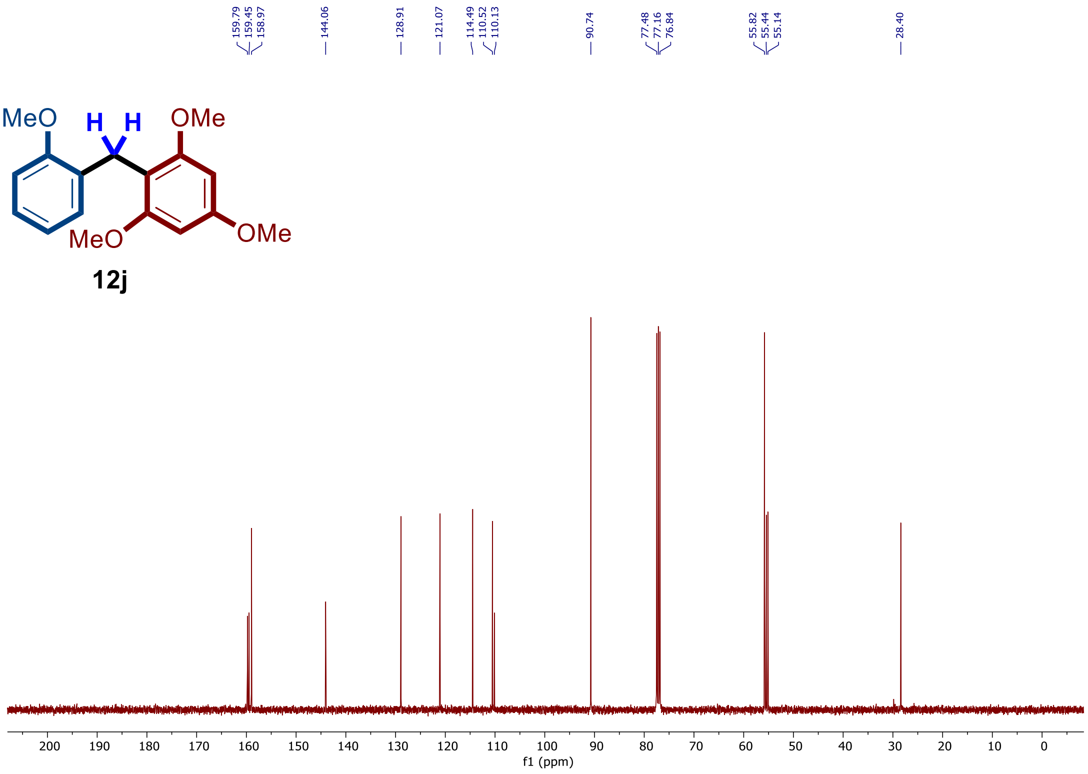

^{13}C NMR (101 MHz, $CDCl_3$) of compound (**12j**)

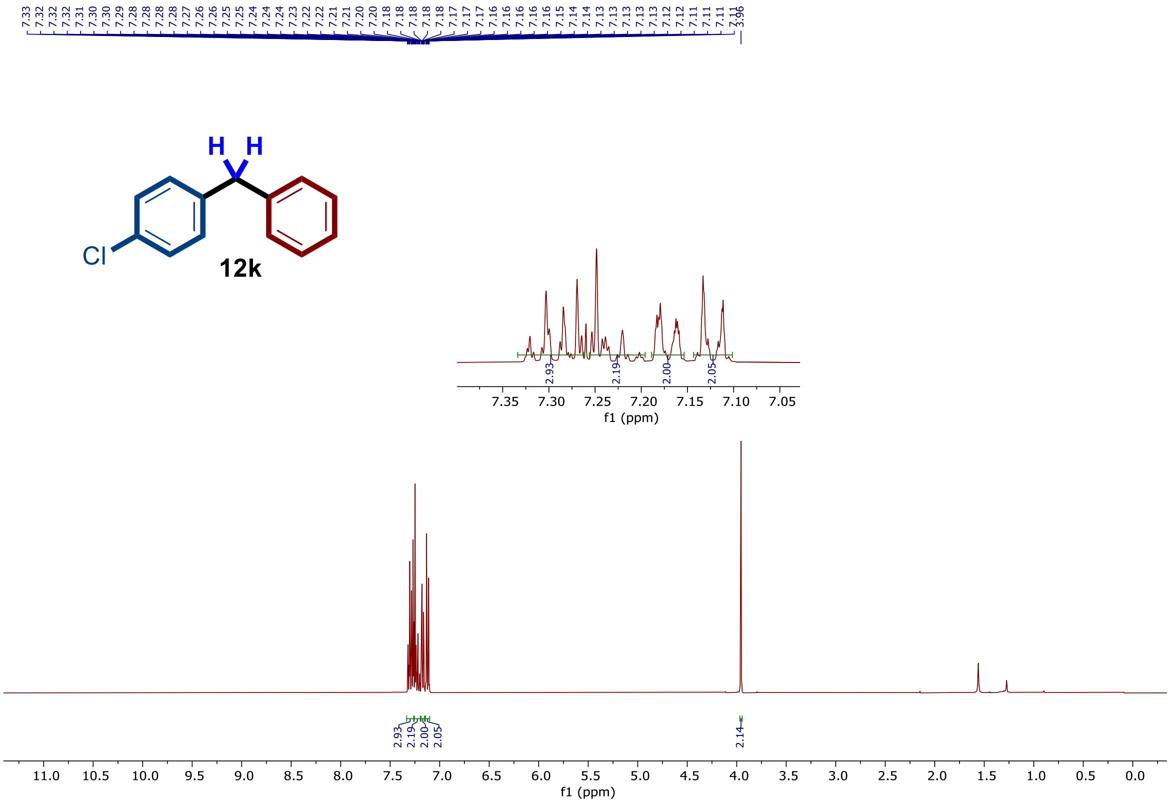

^{1}H NMR (400 MHz, $CDCl_3$) of compound (**12k**)

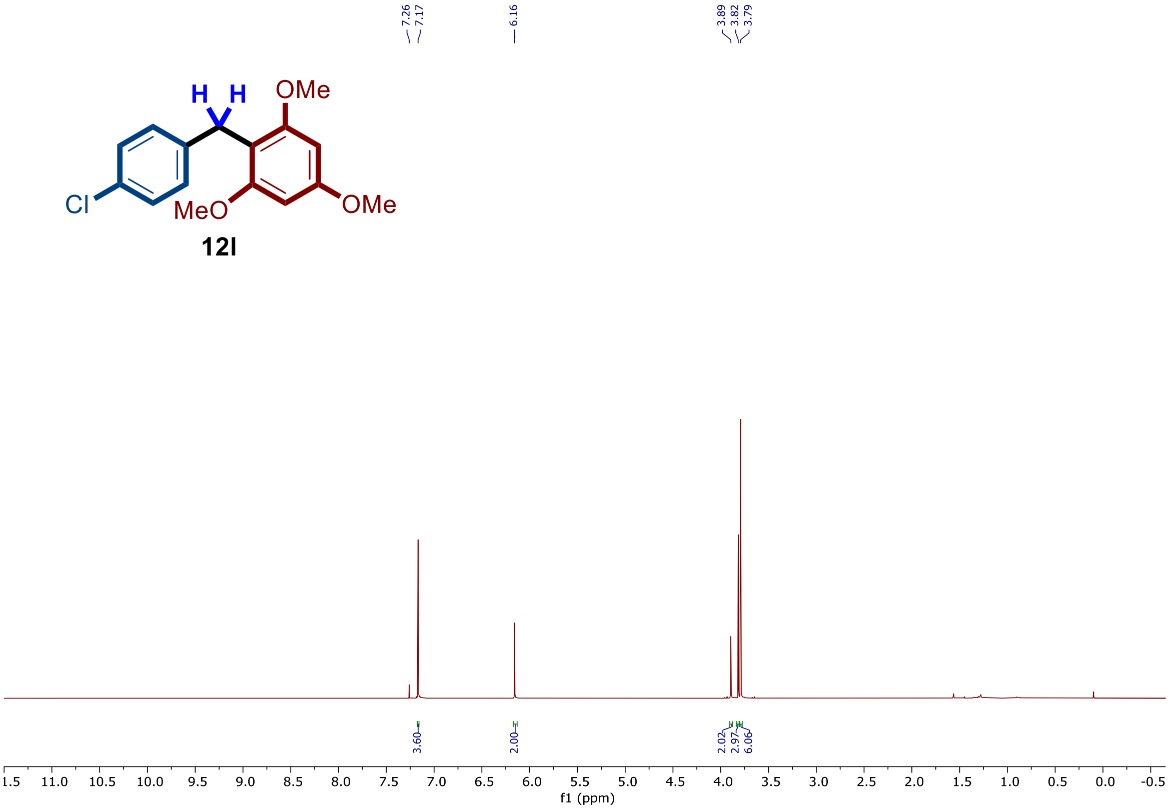

^{1}H NMR (400 MHz, $CDCl_3$) of compound (**12l**)

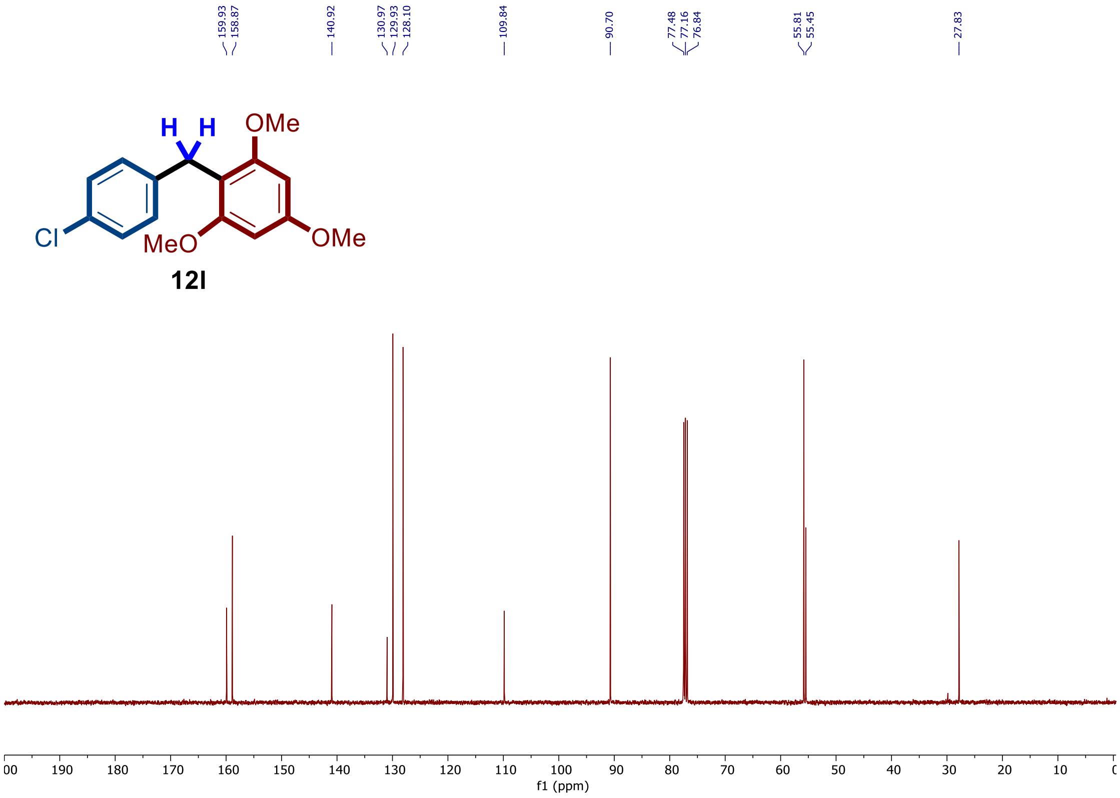

^{13}C NMR (101 MHz, $CDCl_3$) of compound (**12l**)

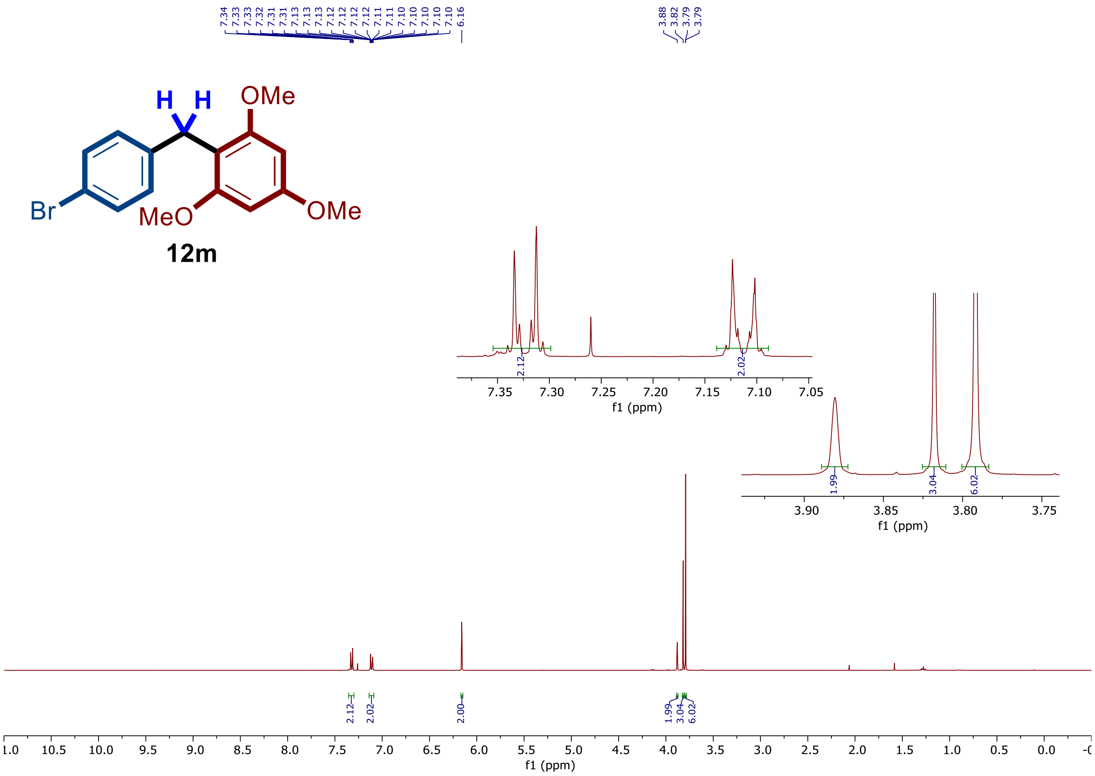

^{1}H NMR (400 MHz, $CDCl_3$) of compound (**12m**)

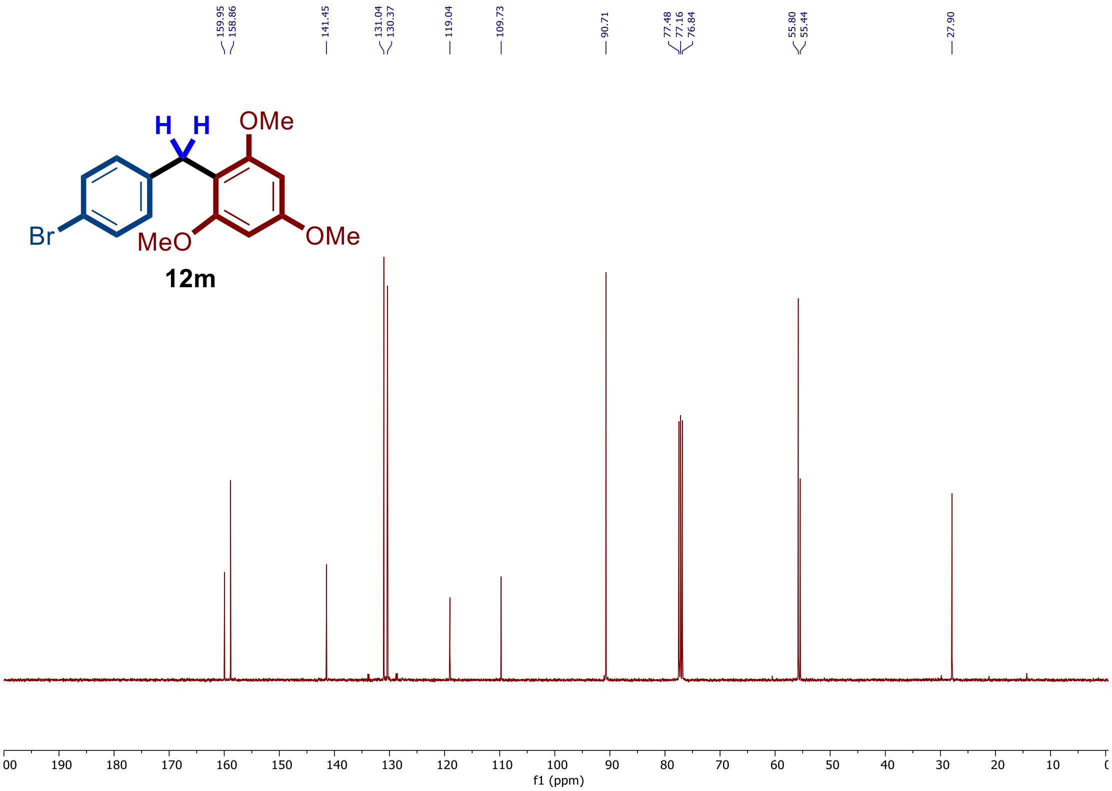

^{13}C NMR (101 MHz, $CDCl_3$) of compound (**12m**)

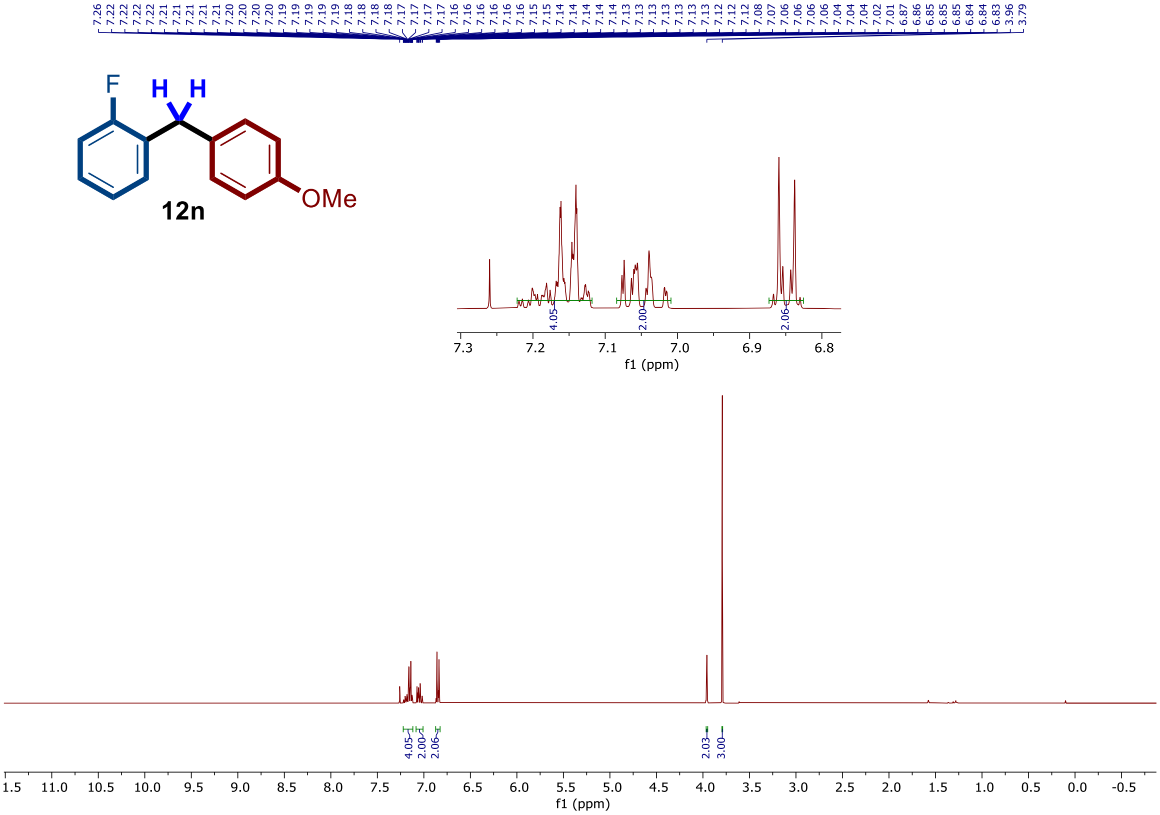

^{1}H NMR (400 MHz, $CDCl_3$) of compound (**12n**)

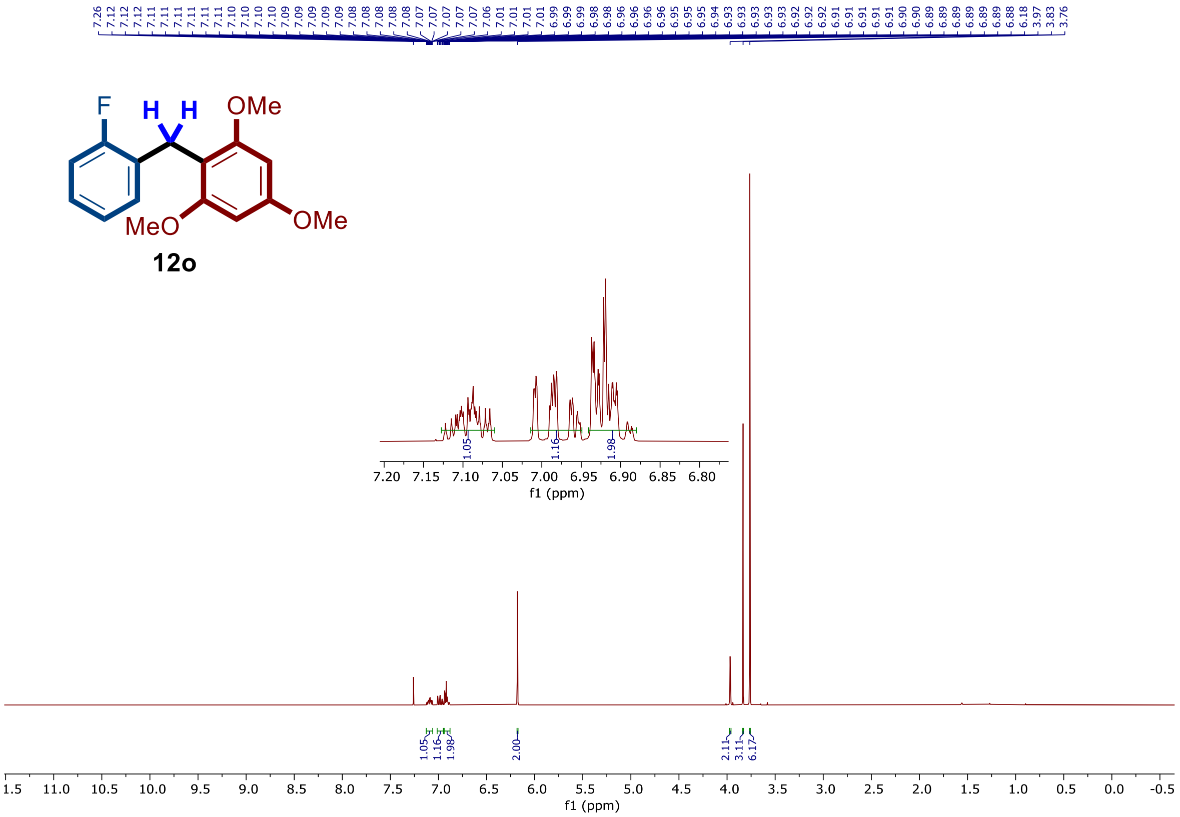

^{1}H NMR (400 MHz, $CDCl_3$) of compound (**12o**)

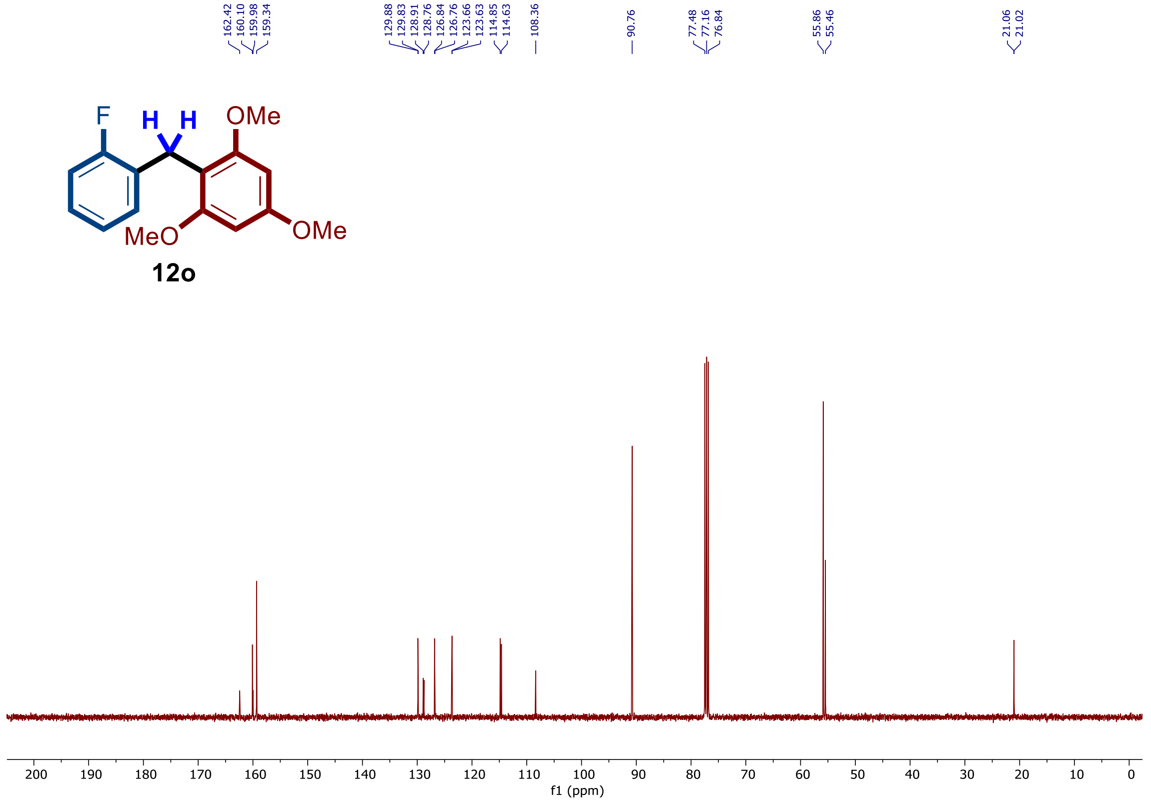

^{13}C NMR (101 MHz, $CDCl_3$) of compound (**12o**)

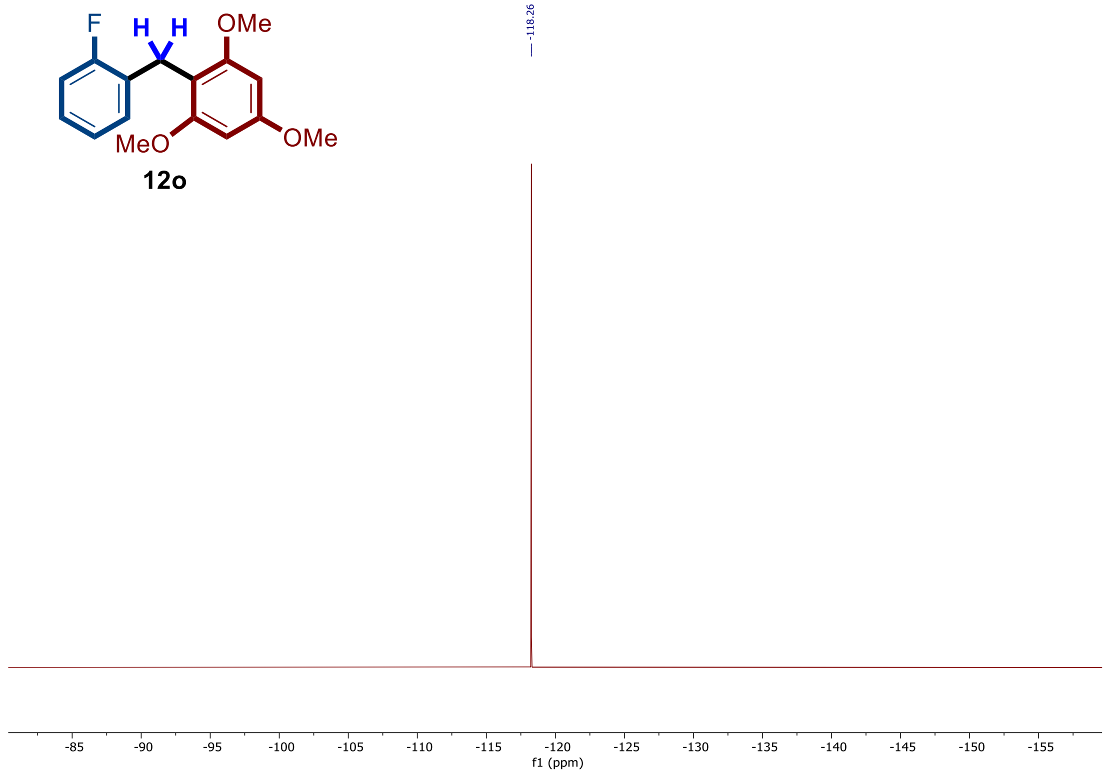

^{19}F NMR (376 MHz, $CDCl_3$) of compound (**12o**)

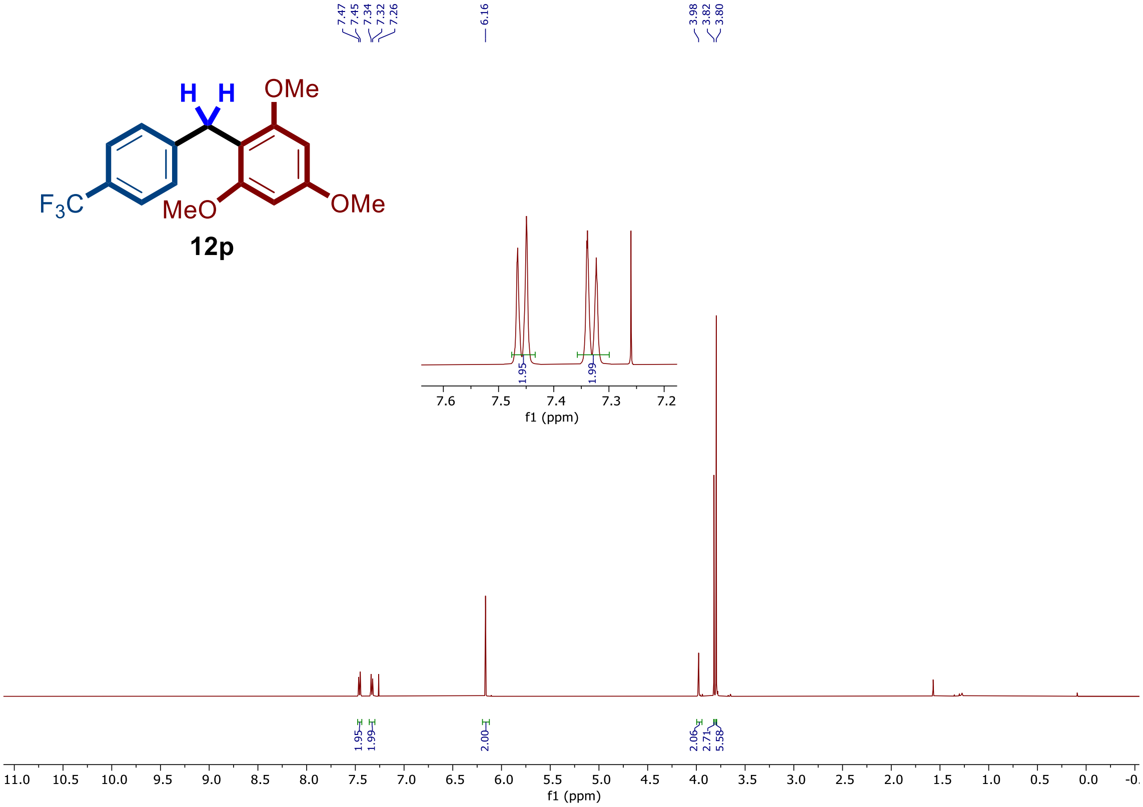

^{1}H NMR (400 MHz, $CDCl_3$) of compound (**12p**)

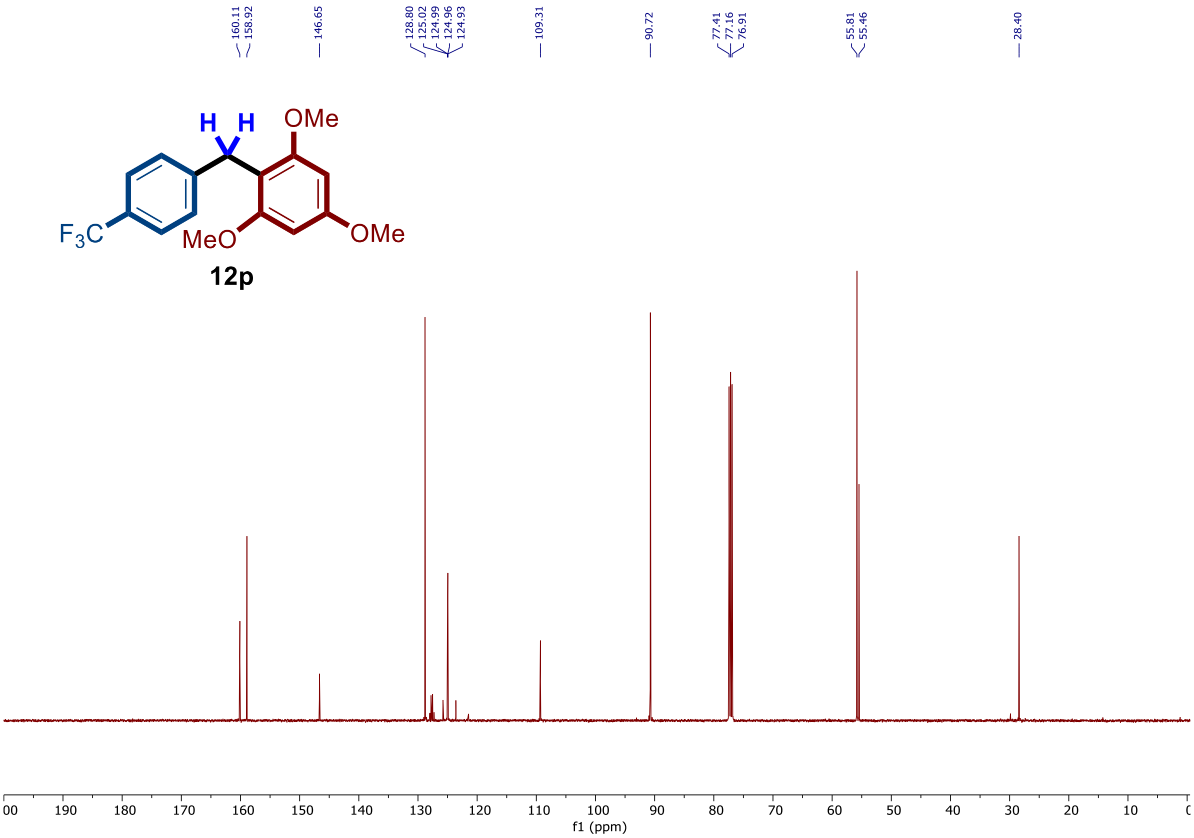

^{13}C NMR (101 MHz, $CDCl_3$) of compound (**12p**)

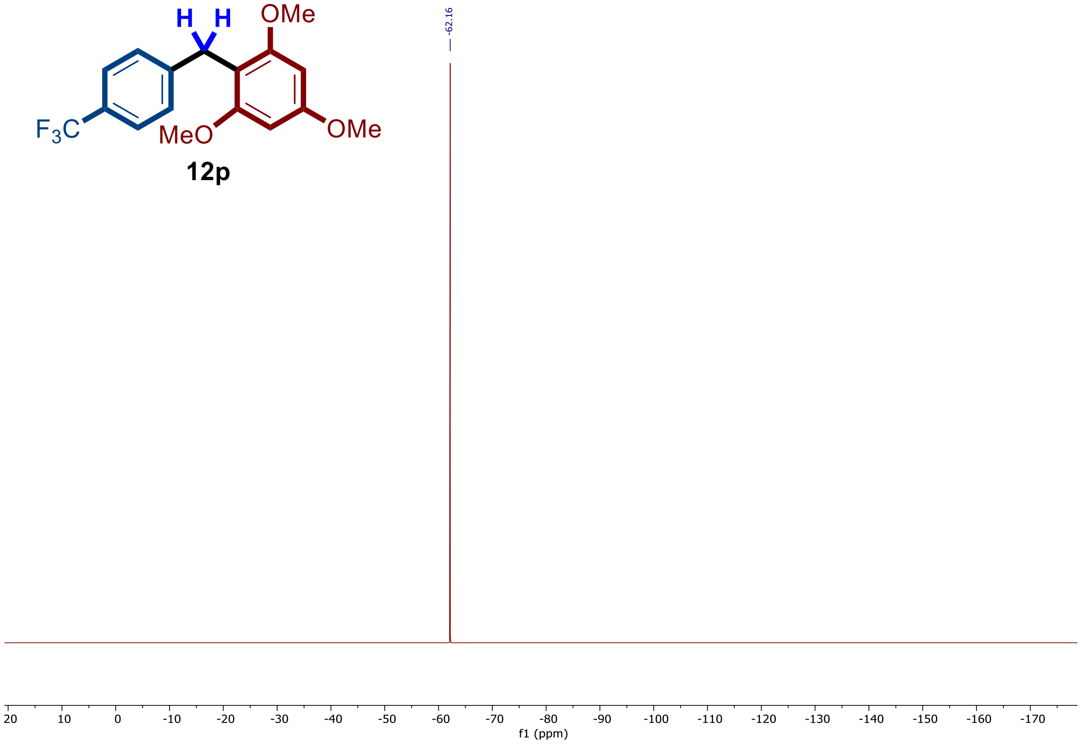

^{19}F NMR (376 MHz, $CDCl_3$) of compound (**12p**)

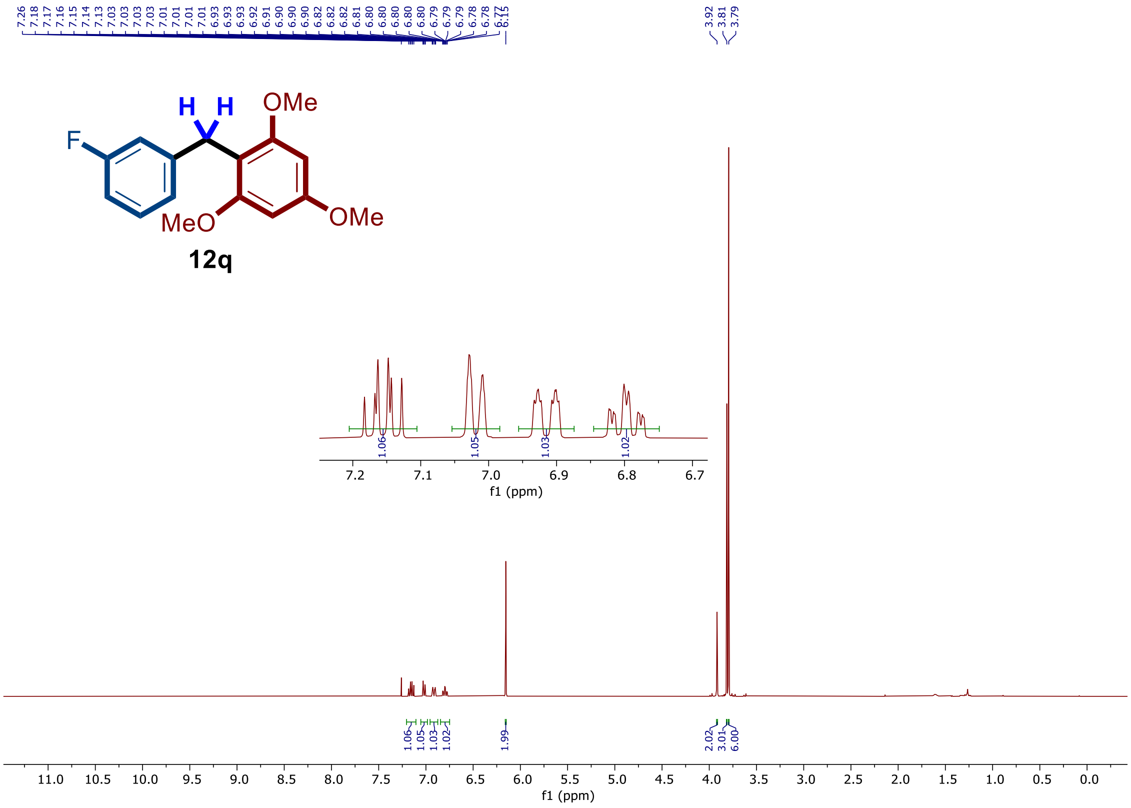

^{1}H NMR (400 MHz, $CDCl_3$) of compound (**12q**)

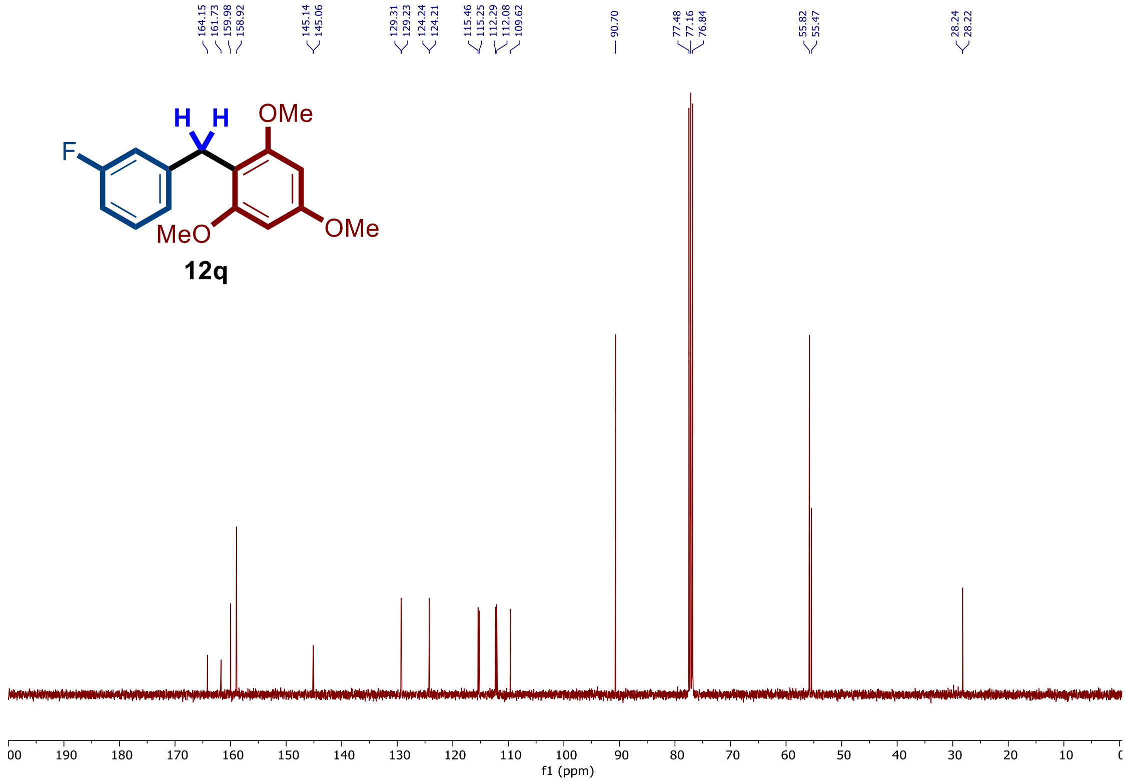

^{13}C NMR (101 MHz, $CDCl_3$) of compound (**12q**)

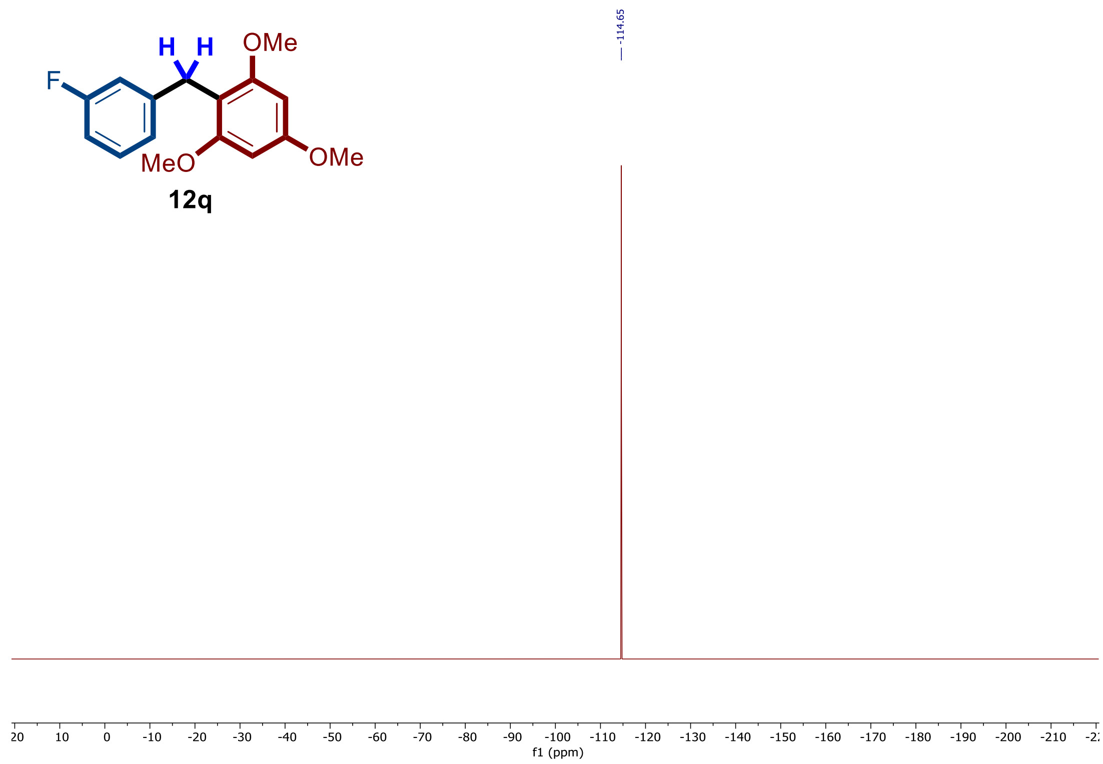

^{19}F NMR (376 MHz, $CDCl_3$) of compound (**12q**)

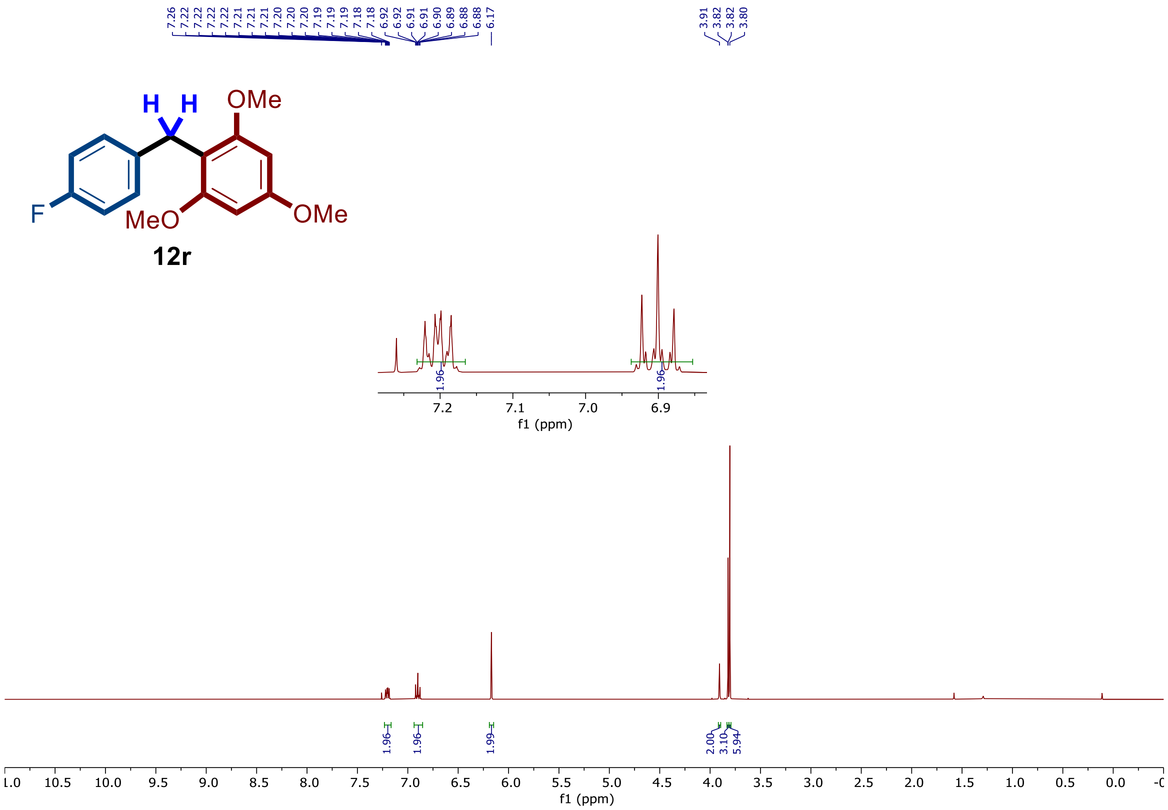

^{1}H NMR (400 MHz, $CDCl_3$) of compound (**12r**)

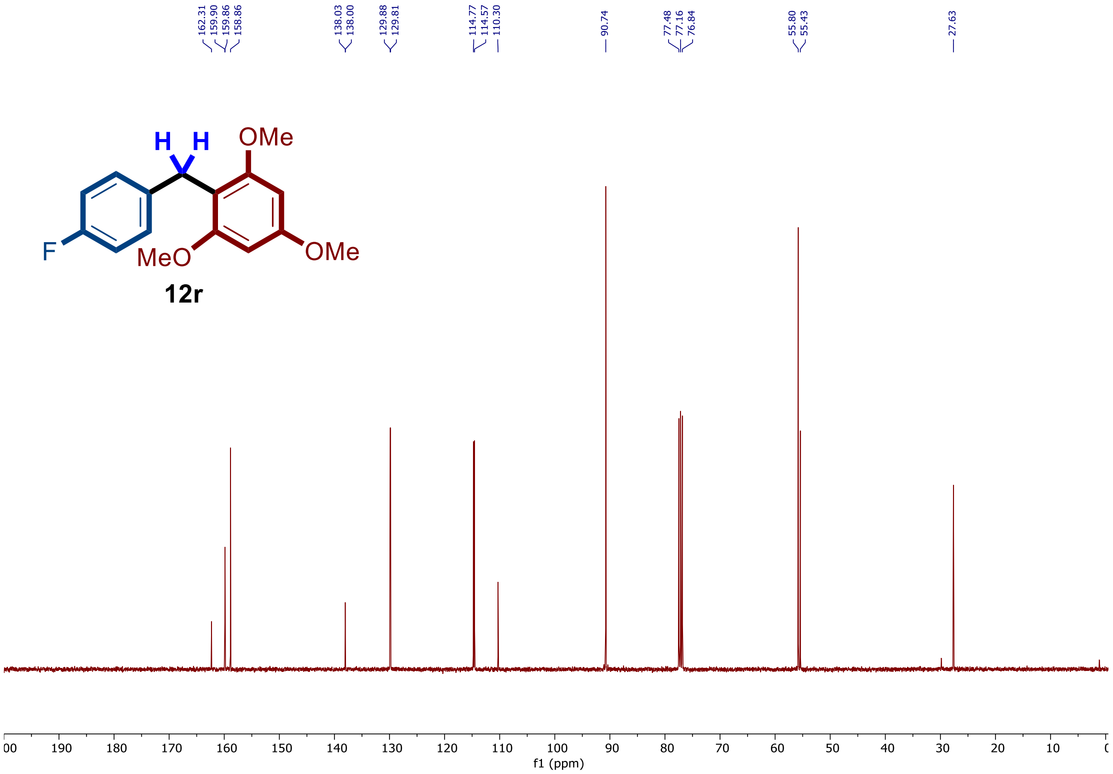

^{13}C NMR (101 MHz, $CDCl_3$) of compound (**12r**)

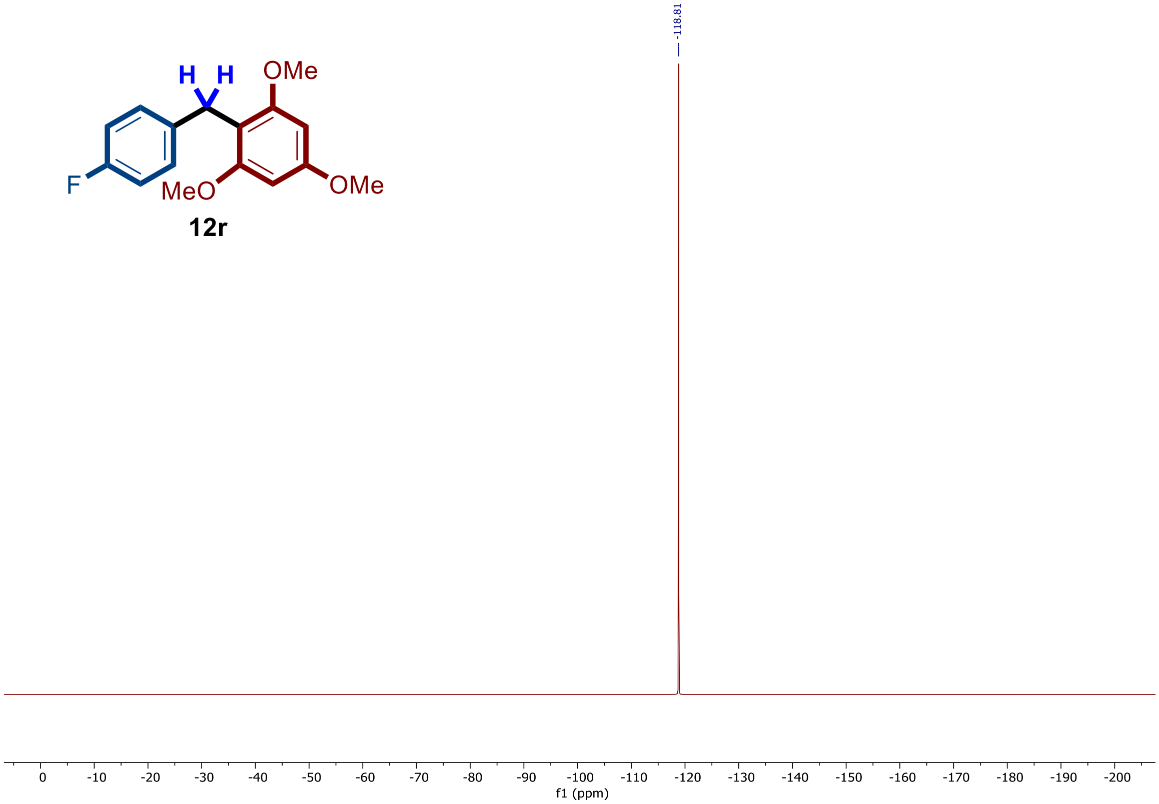

^{19}F NMR (376 MHz, $CDCl_3$) of compound (**12r**)

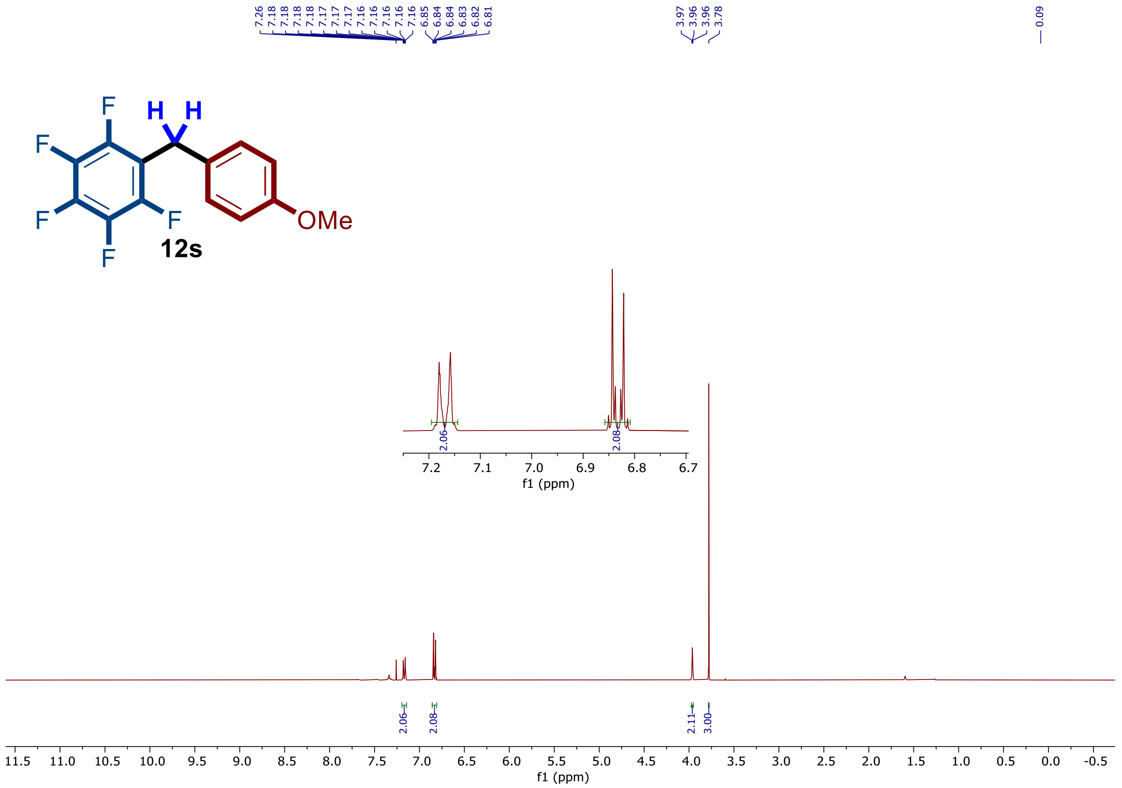

^{1}H NMR (400 MHz, $CDCl_3$) of compound (**12s**)

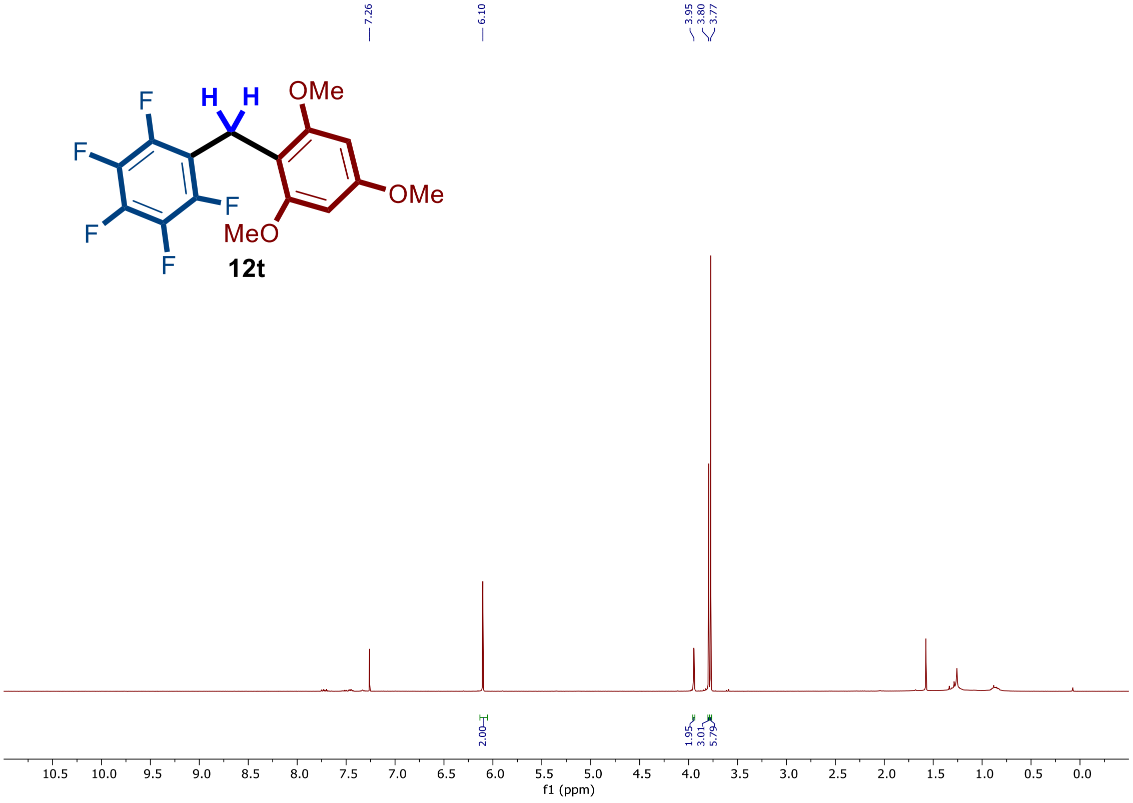

^{1}H NMR (400 MHz, $CDCl_3$) of compound (**12t**)

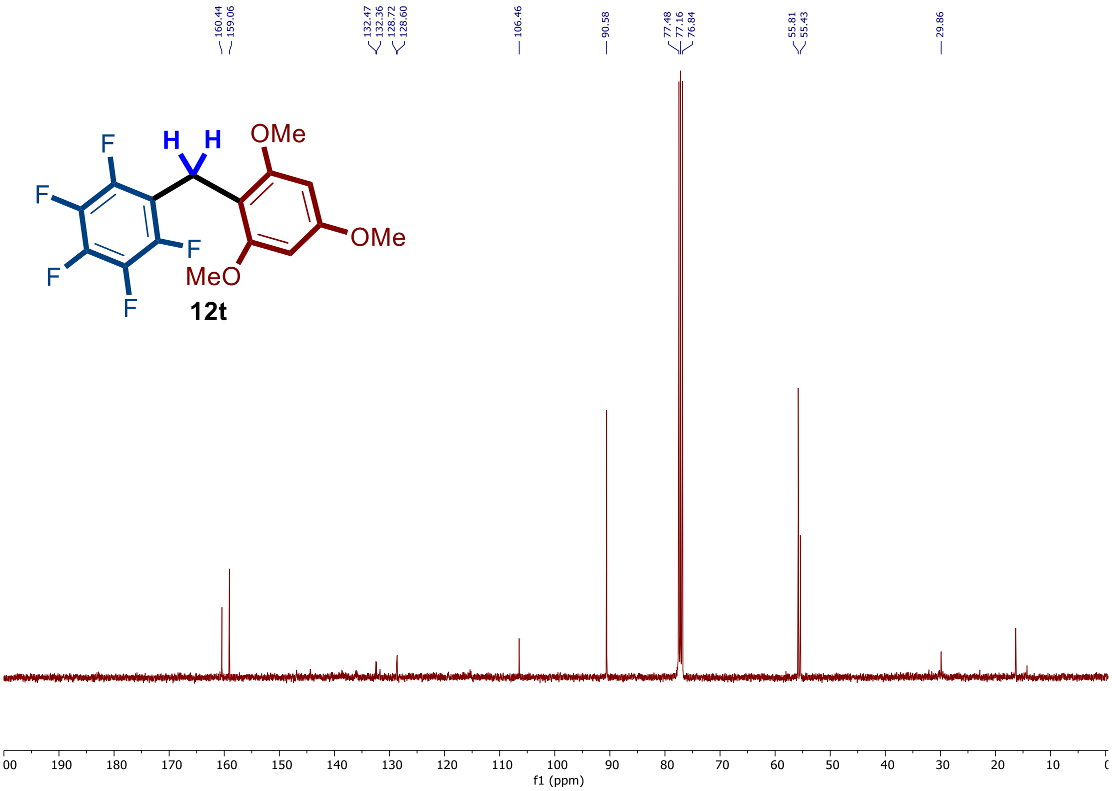

^{13}C NMR (101 MHz, $CDCl_3$) of compound (**12t**)

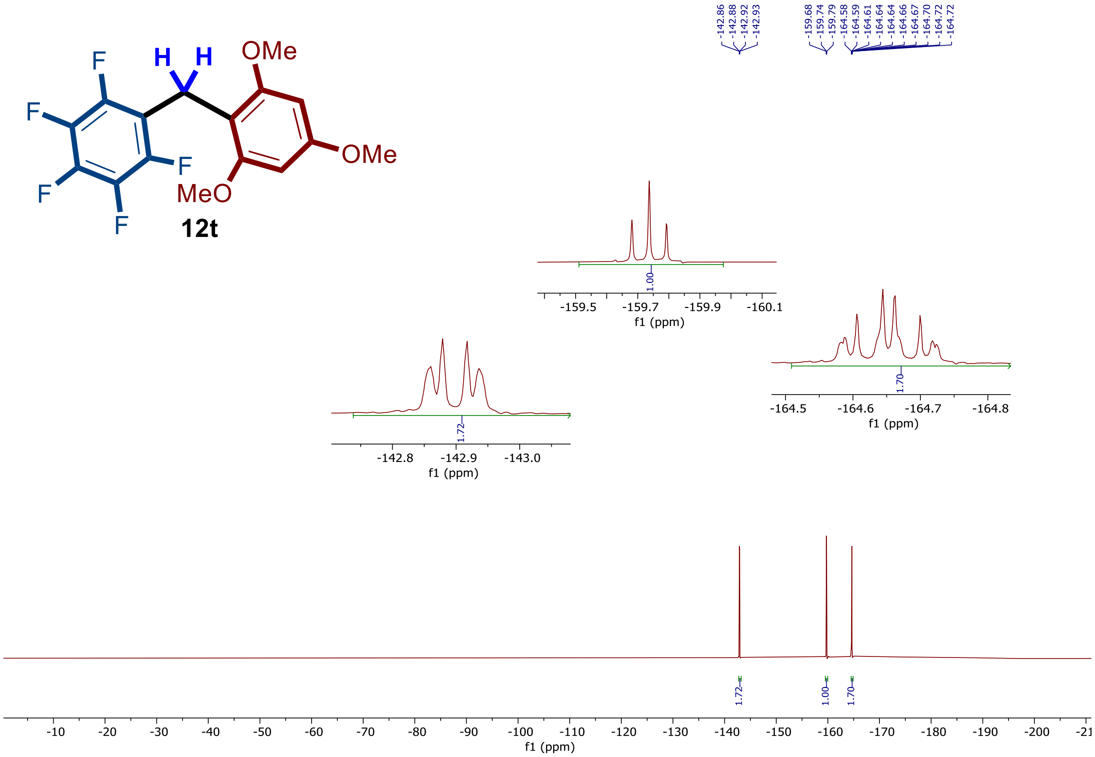

^{19}F NMR (376 MHz, $CDCl_3$) of compound (**12t**)

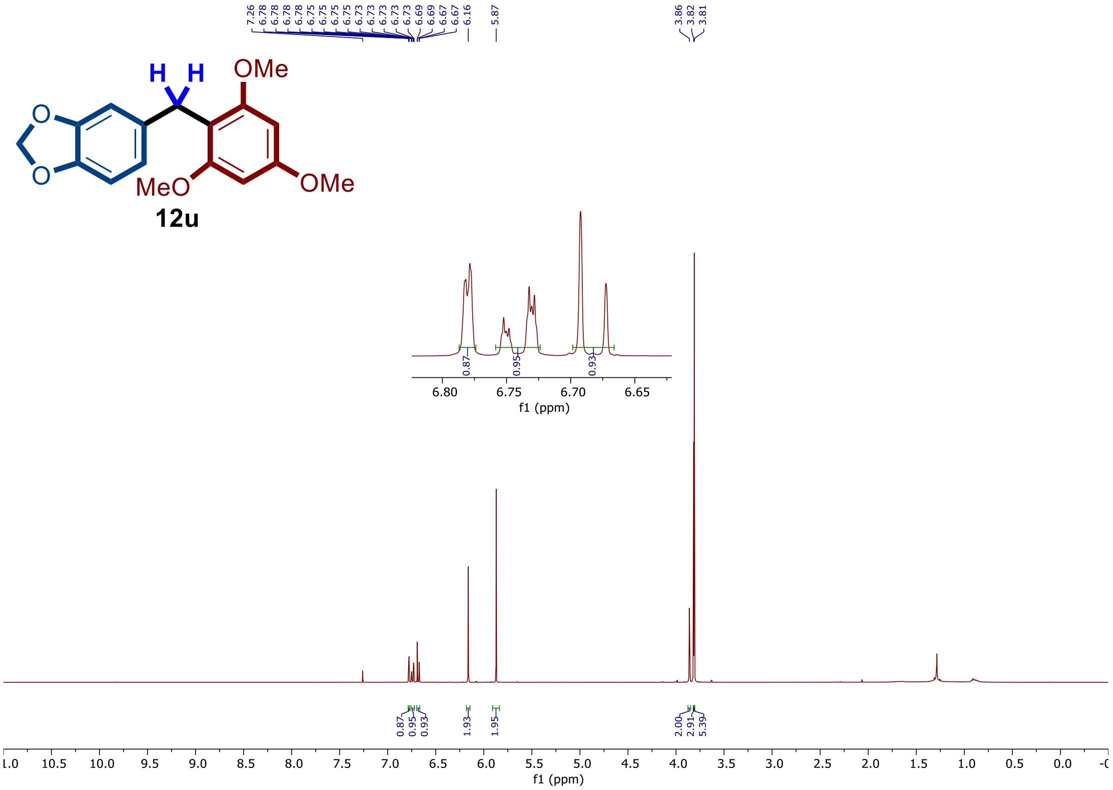

^{1}H NMR (400 MHz, $CDCl_3$) of compound (**12u**)

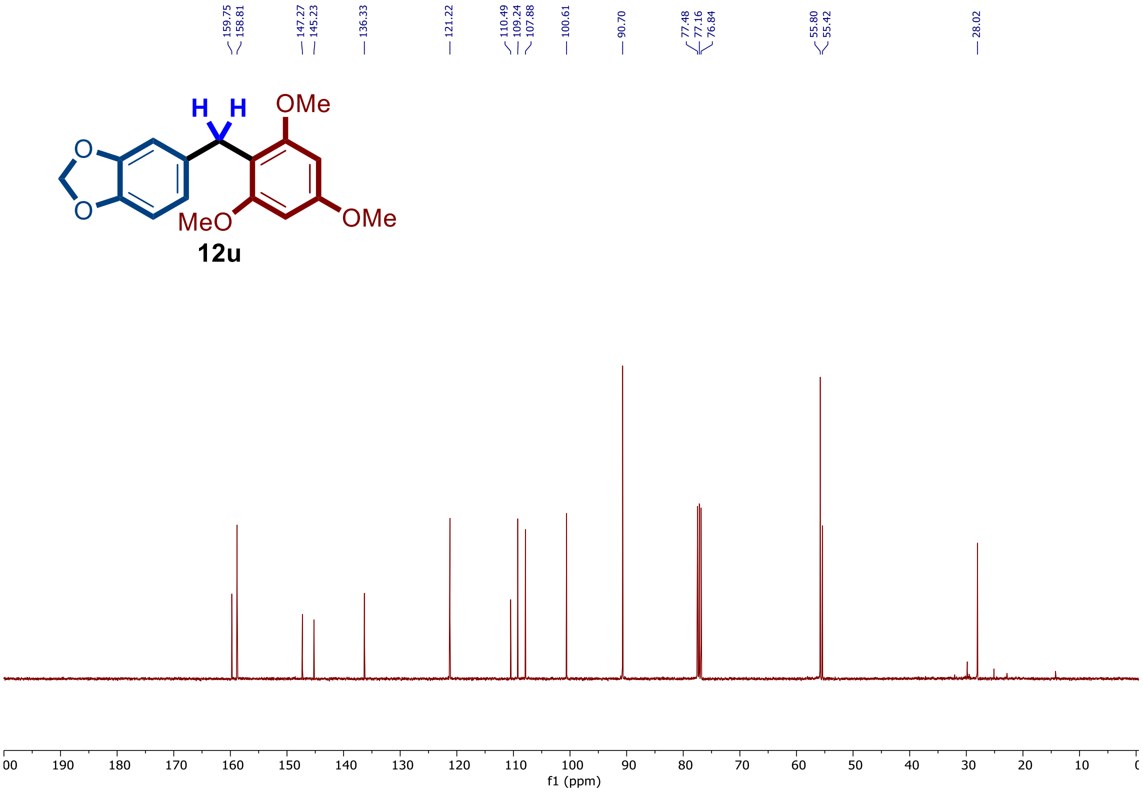

^{13}C NMR (101 MHz, $CDCl_3$) of compound (**12u**)

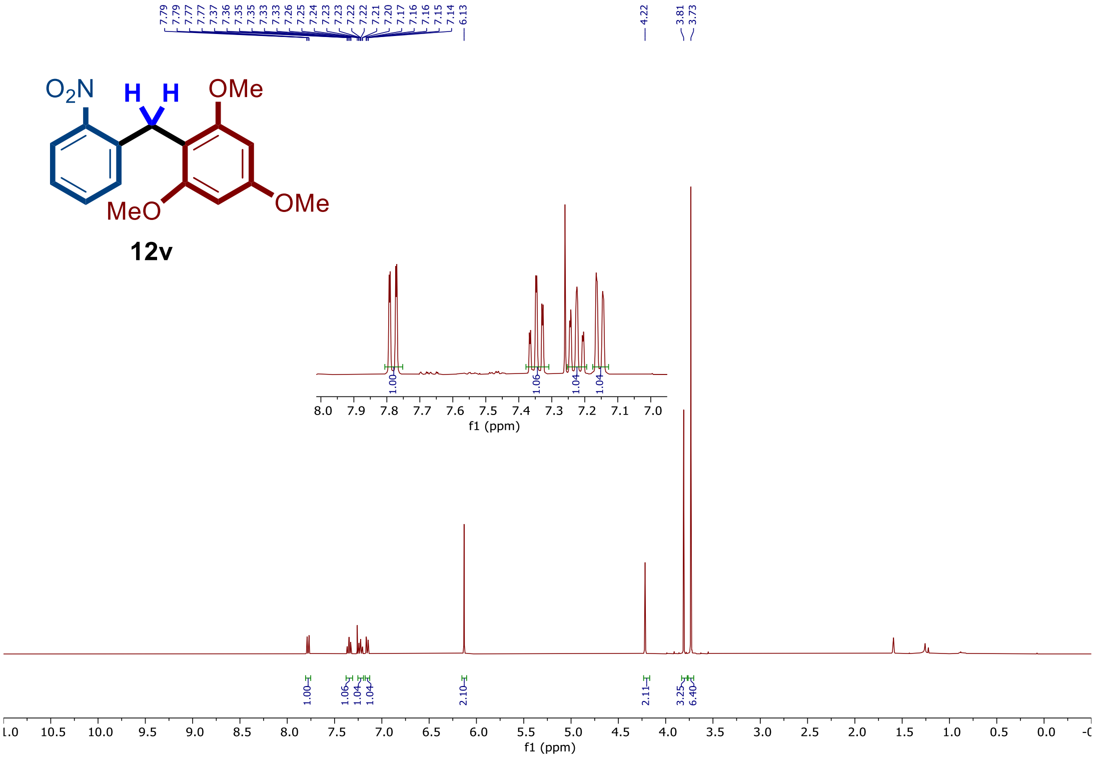

^{1}H NMR (400 MHz, $CDCl_3$) of compound (**12v**)

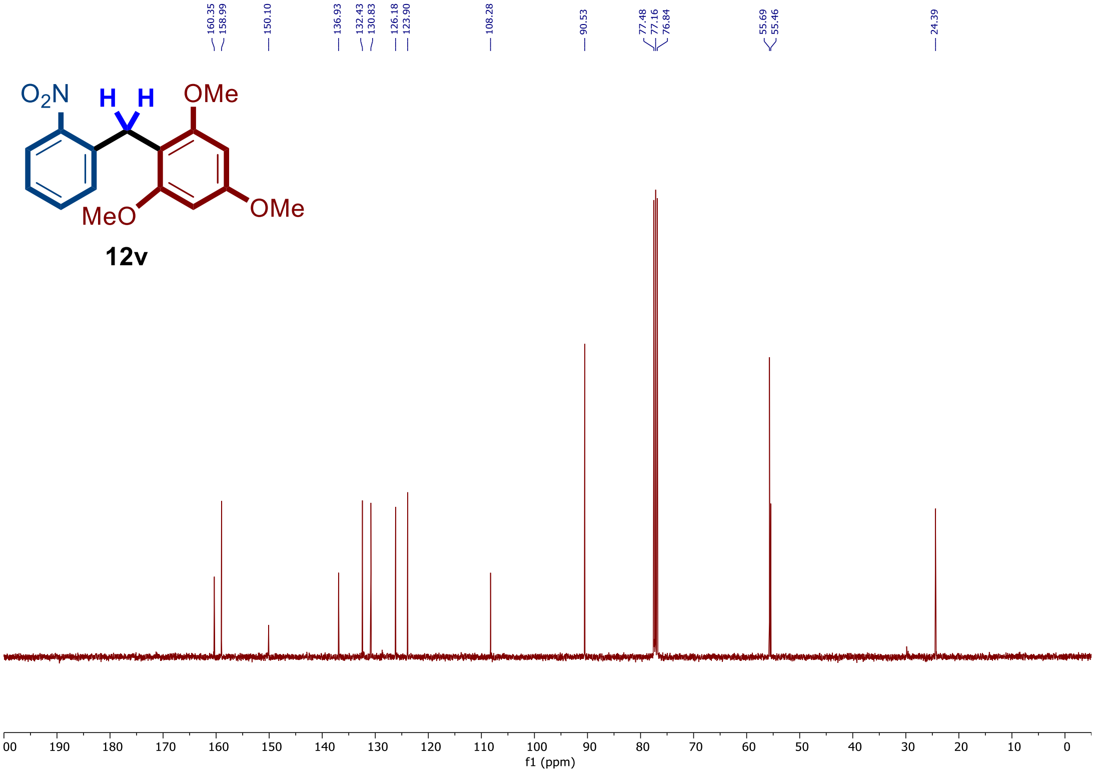

^{13}C NMR (101 MHz, $CDCl_3$) of compound (**12v**)

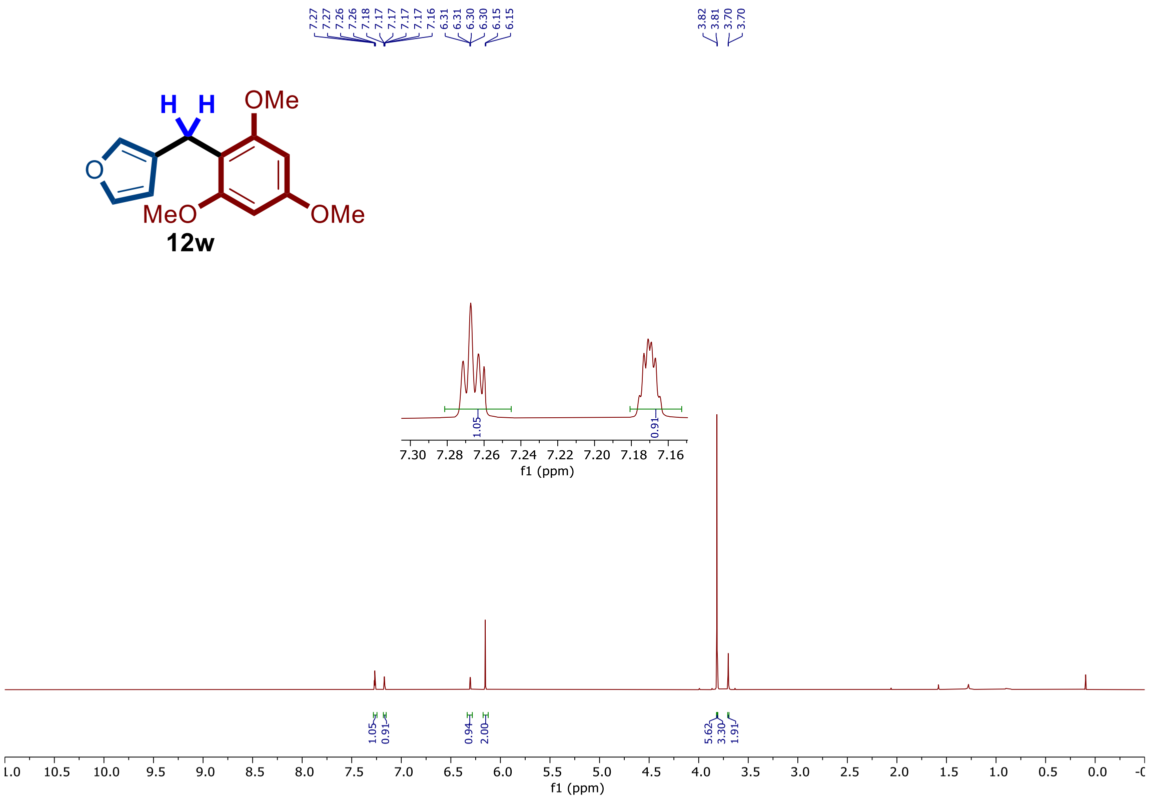

^{1}H NMR (400 MHz, $CDCl_3$) of compound (**12w**)

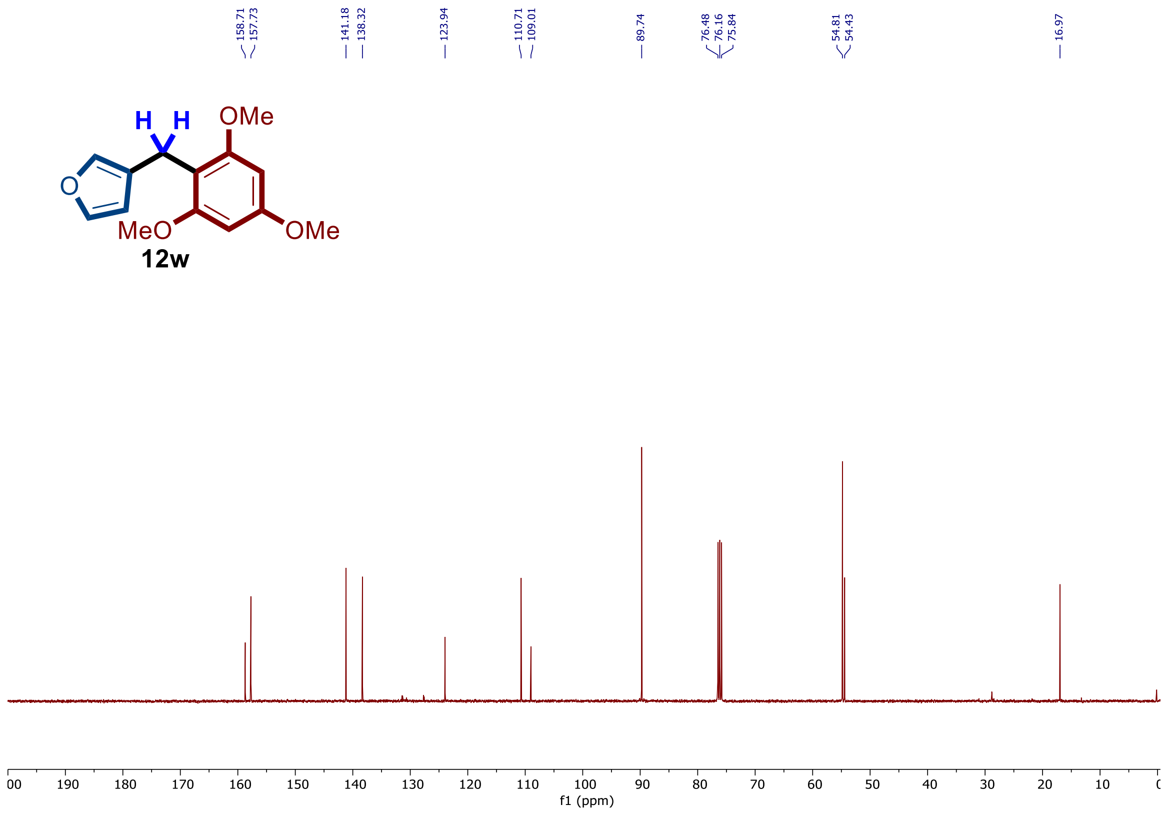

^{13}C NMR (101 MHz, $CDCl_3$) of compound (**12w**)

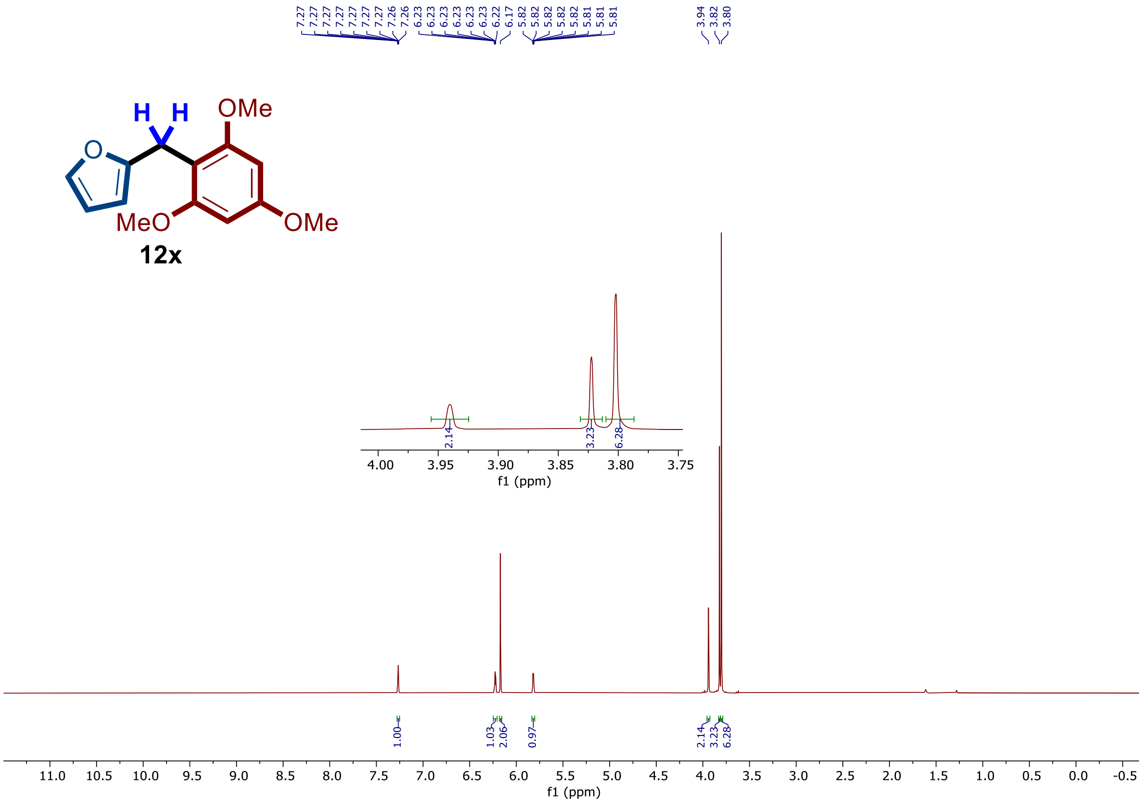

^{1}H NMR (400 MHz, $CDCl_3$) of compound (**12x**)

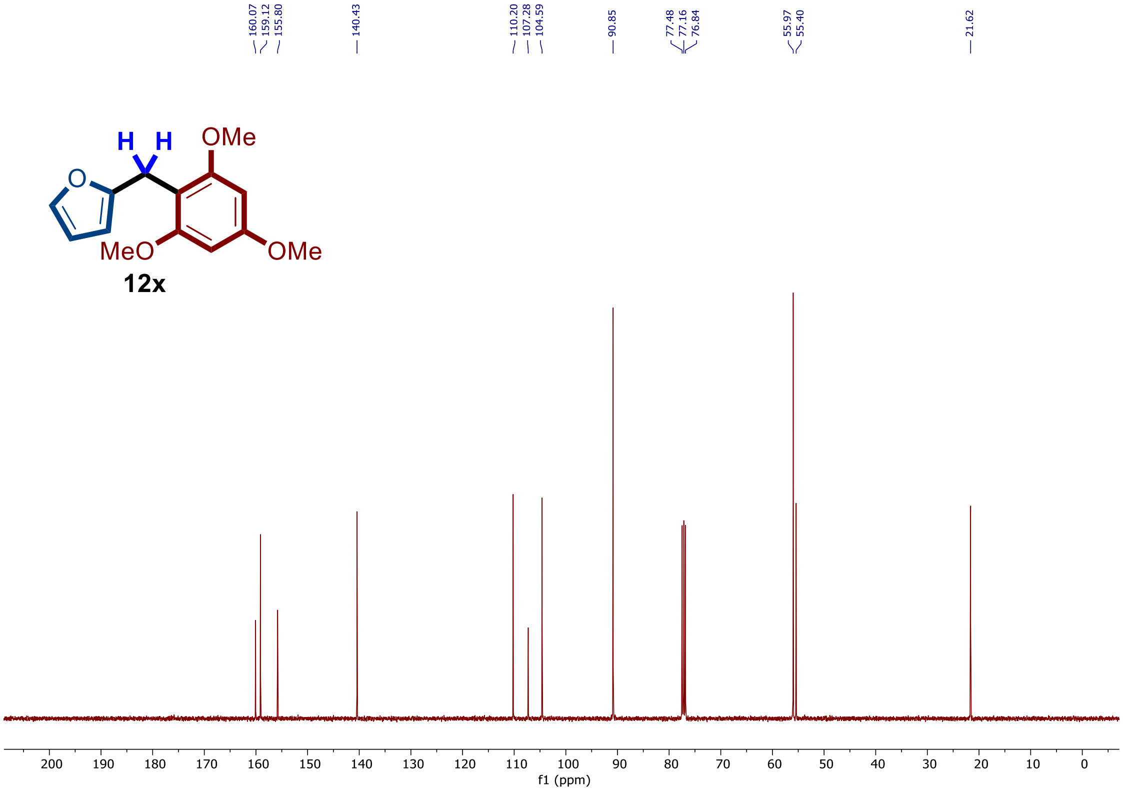

^{13}C NMR (101 MHz, $CDCl_3$) of compound (**12x**)

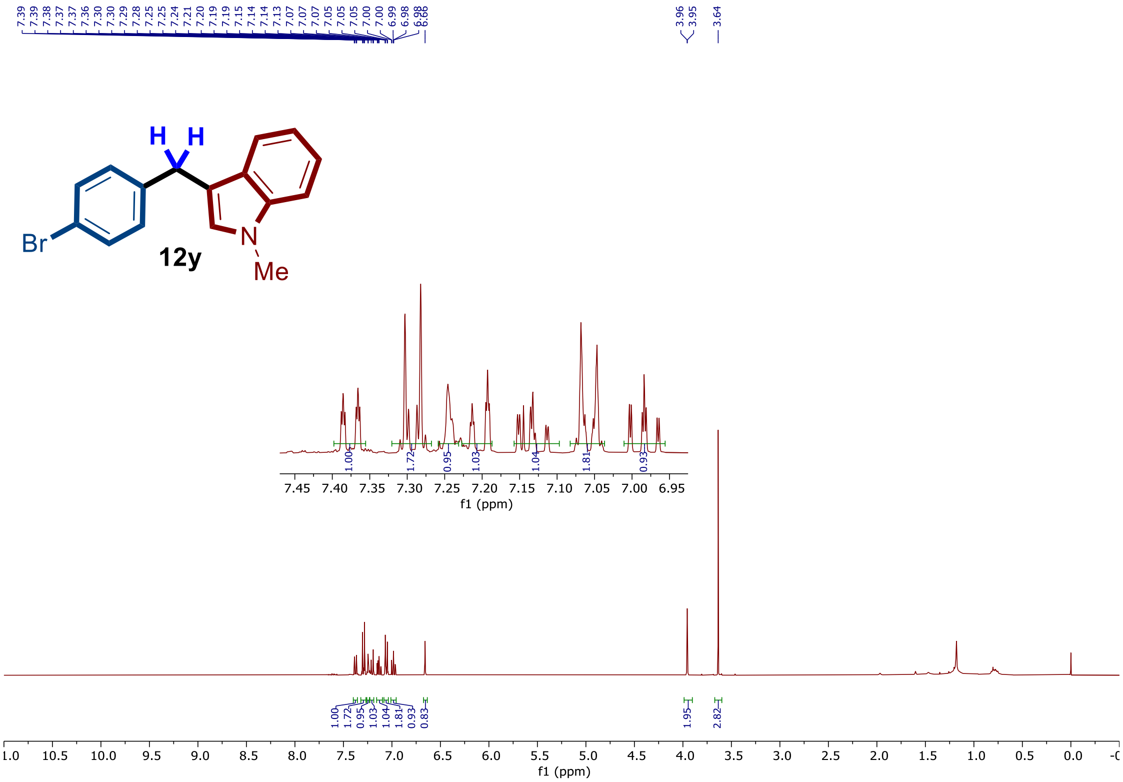

^{1}H NMR (400 MHz, $CDCl_3$) of compound (**12y**)

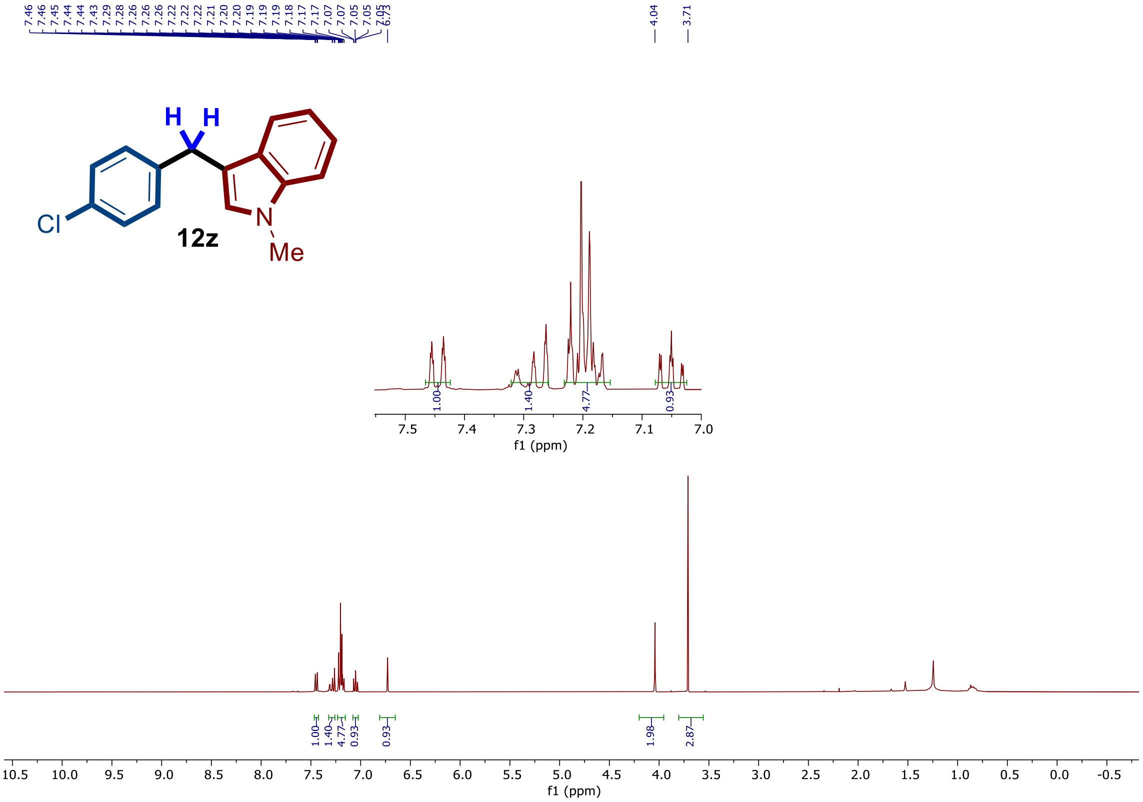

^{1}H NMR (400 MHz, $CDCl_3$) of compound (**12z**)

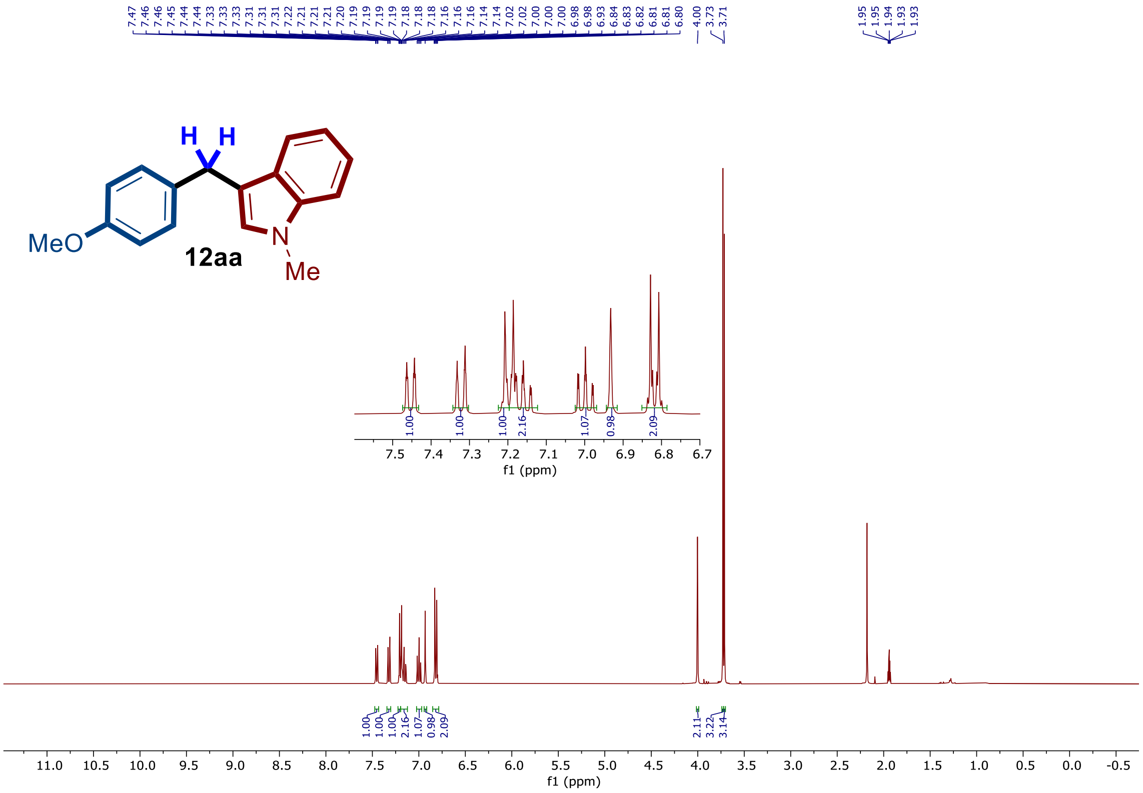

^{1}H NMR (400 MHz, CD_3CN) of compound (**12aa**)

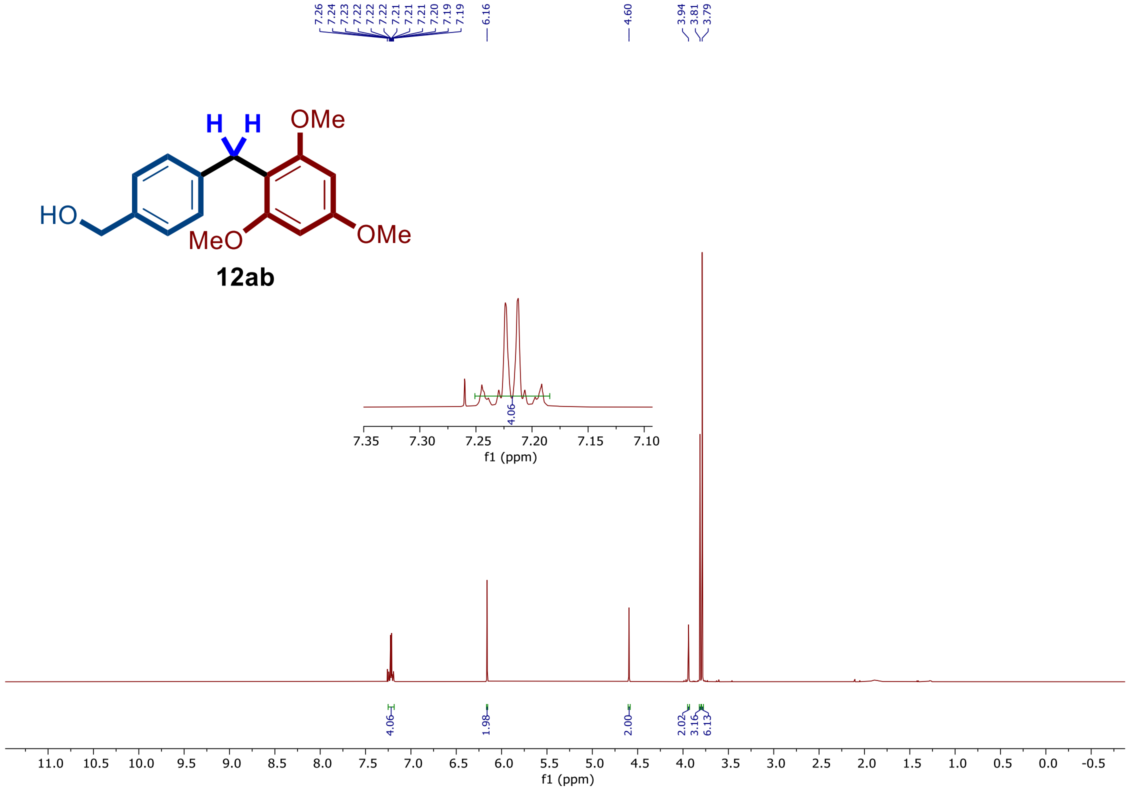

^{1}H NMR (400 MHz, $CDCl_3$) of compound (**12ab**)

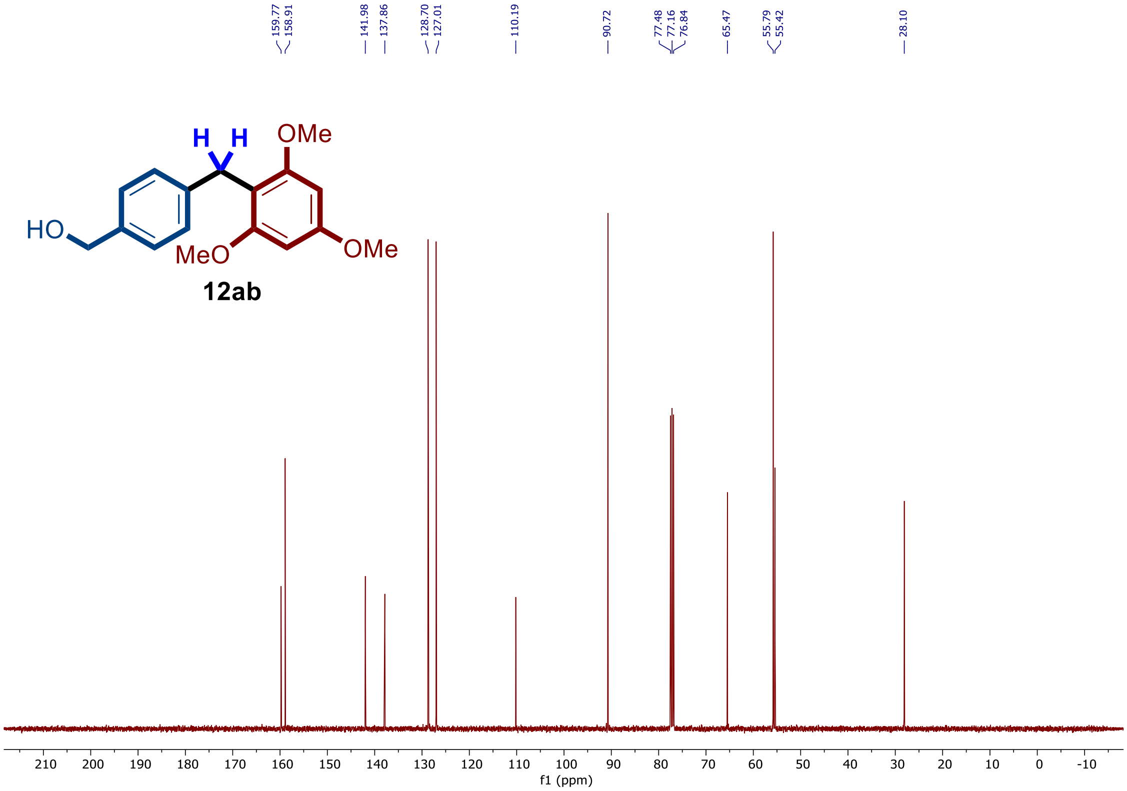

^{13}C NMR (101 MHz, $CDCl_3$) of compound (**12ab**)

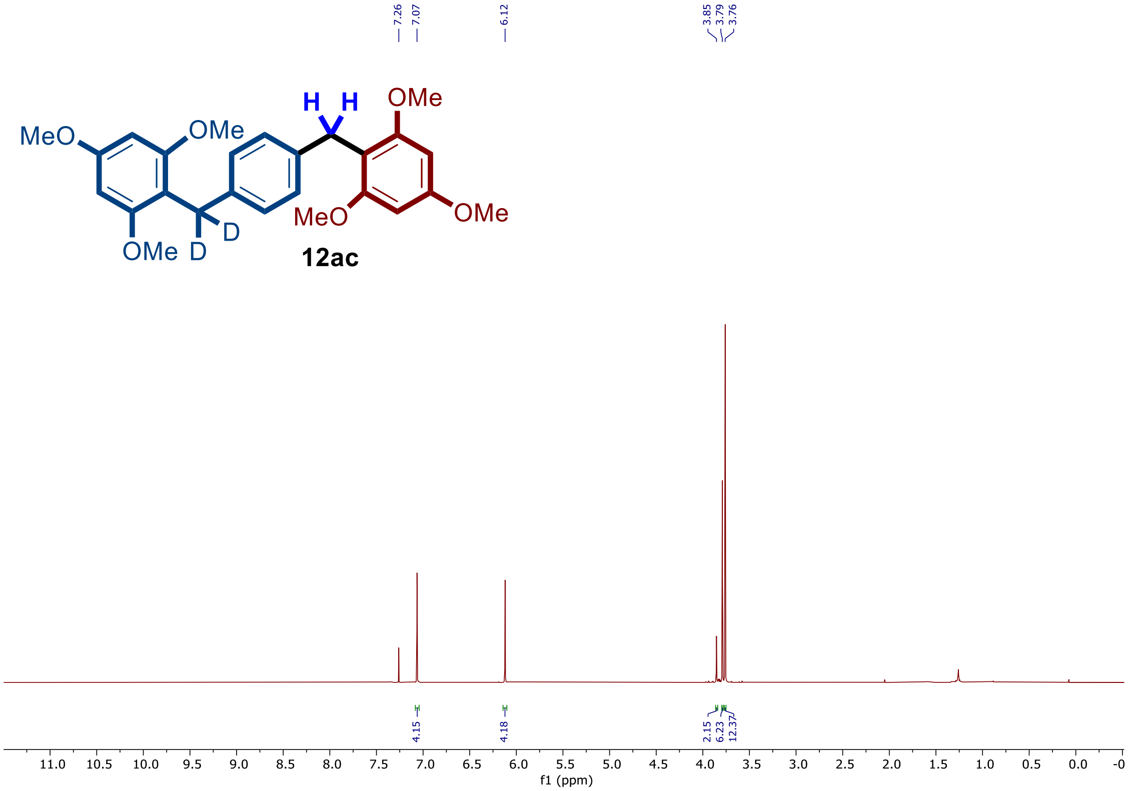

^{1}H NMR (400 MHz, $CDCl_3$) of compound (**12ac**)

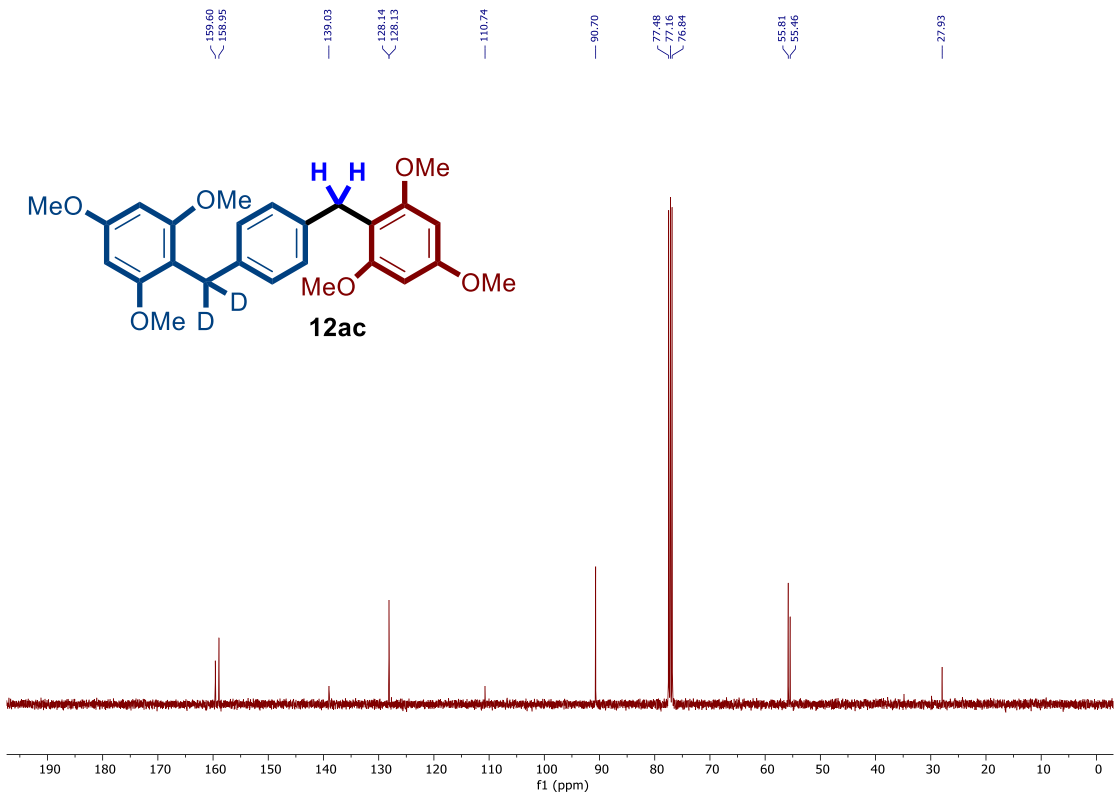

^{13}C NMR (101 MHz, $CDCl_3$) of compound (**12ac**)

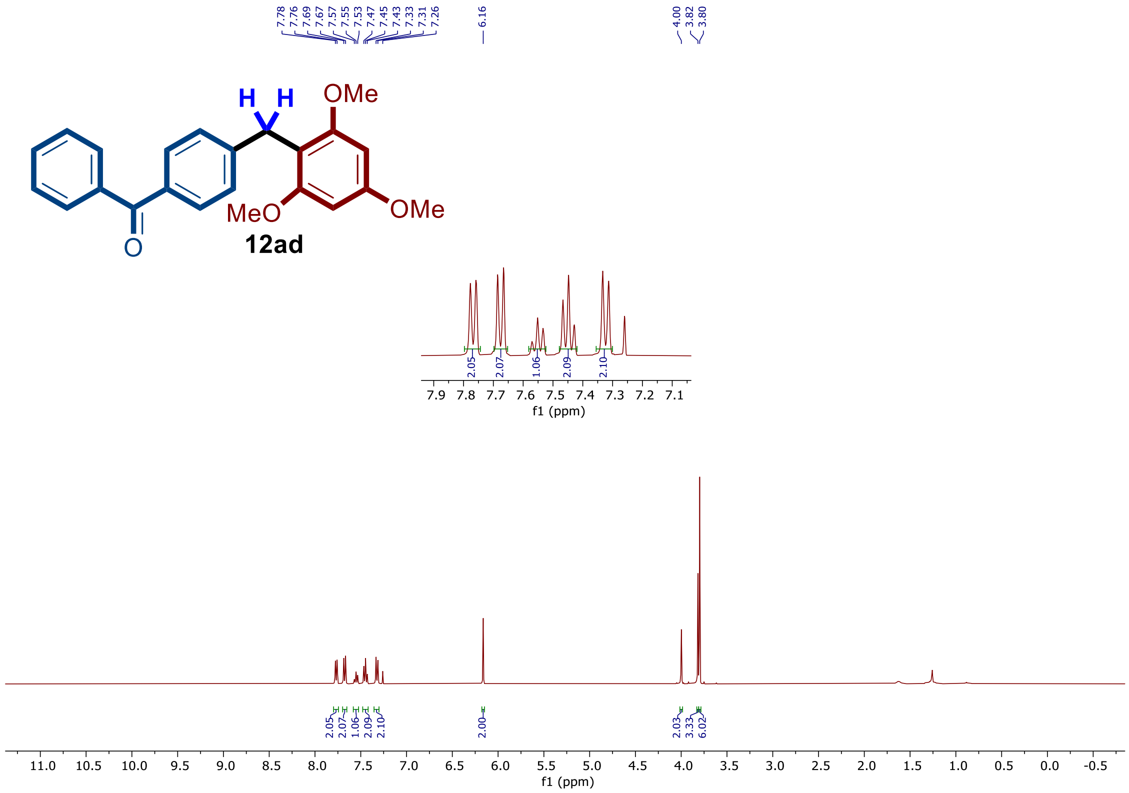

^{1}H NMR (400 MHz, $CDCl_3$) of compound (**12ad**)

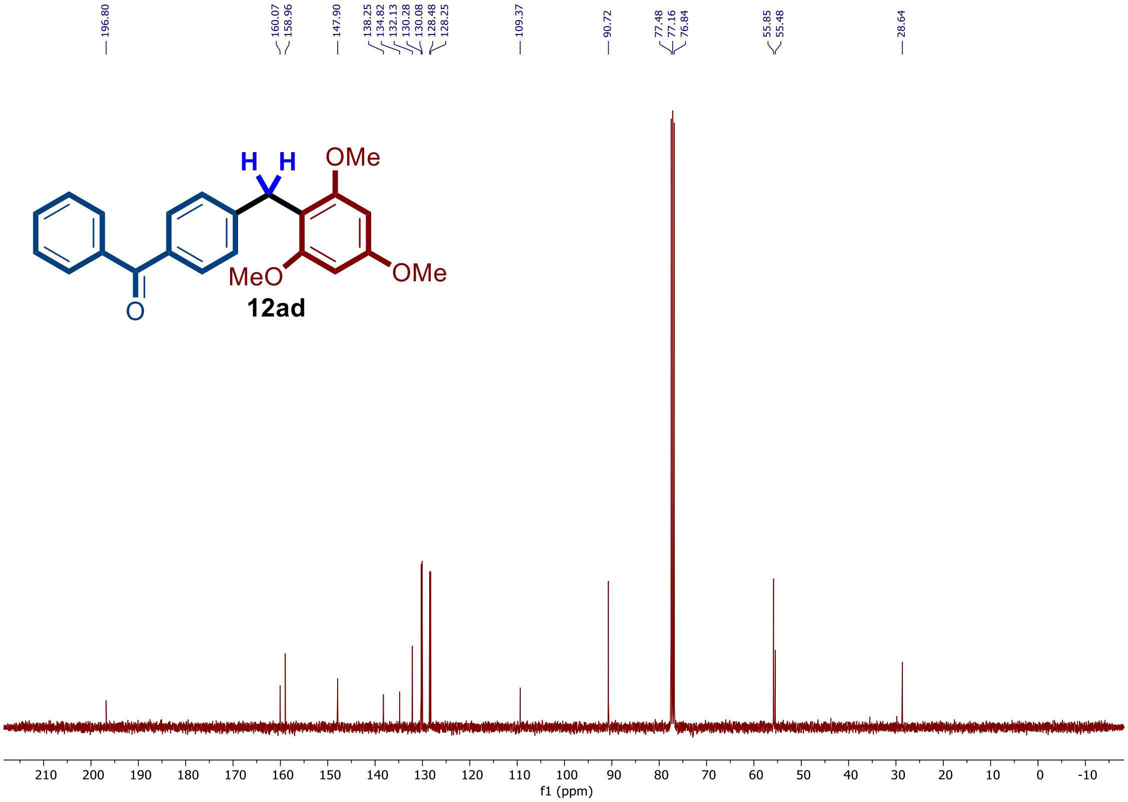

^{13}C NMR (101 MHz, $CDCl_3$) of compound (**12ad**)

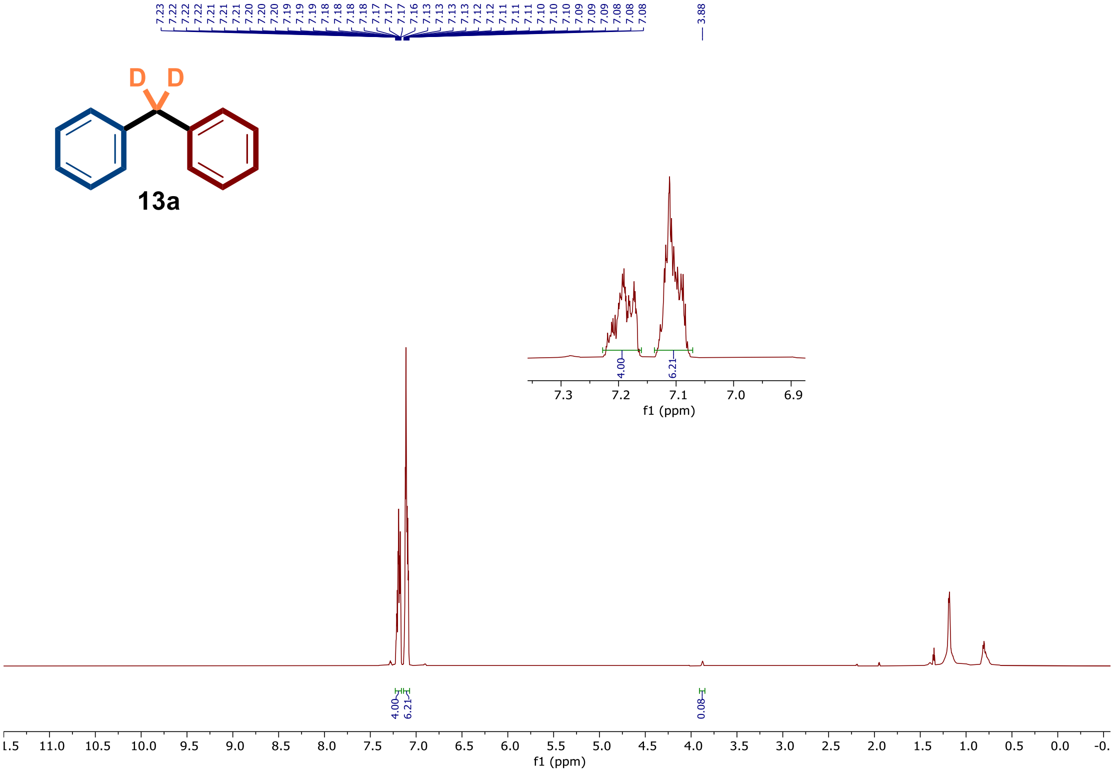

^{1}H NMR (400 MHz, $CDCl_3$) of compound (**13a**)

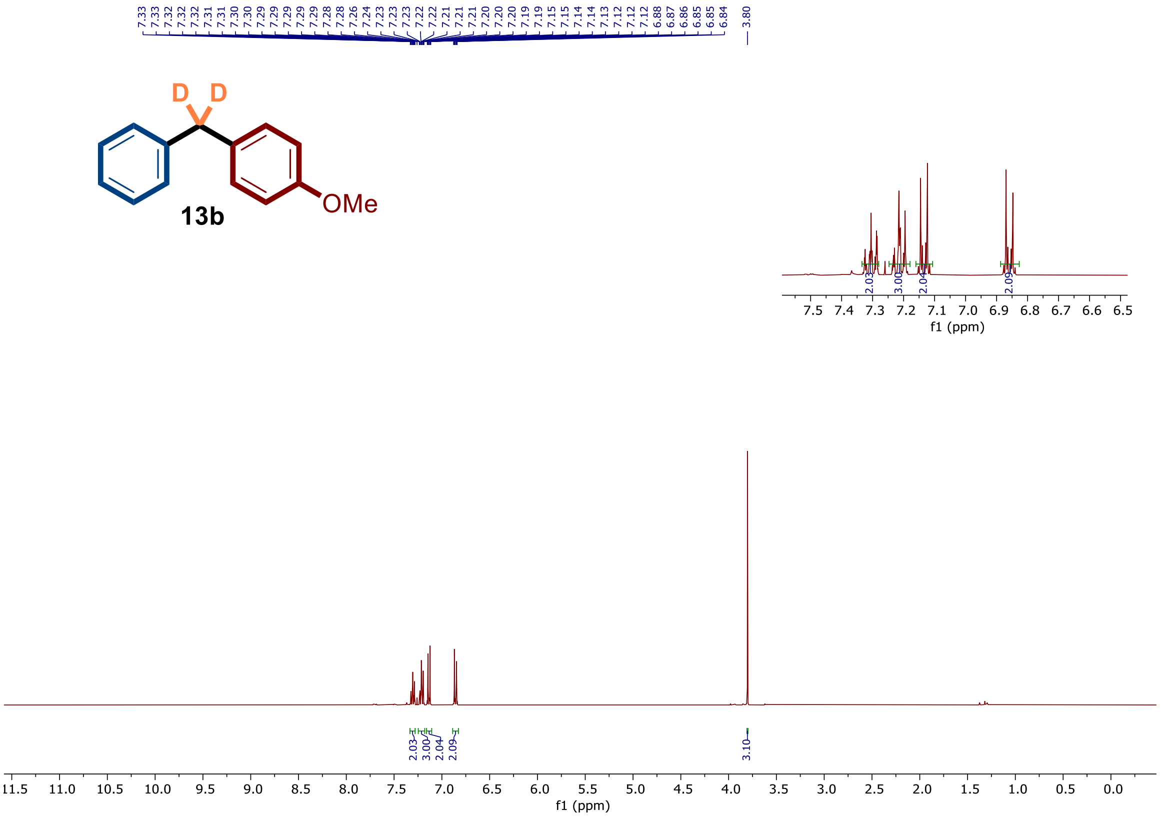

^{1}H NMR (400 MHz, $CDCl_3$) of compound (**13b**)

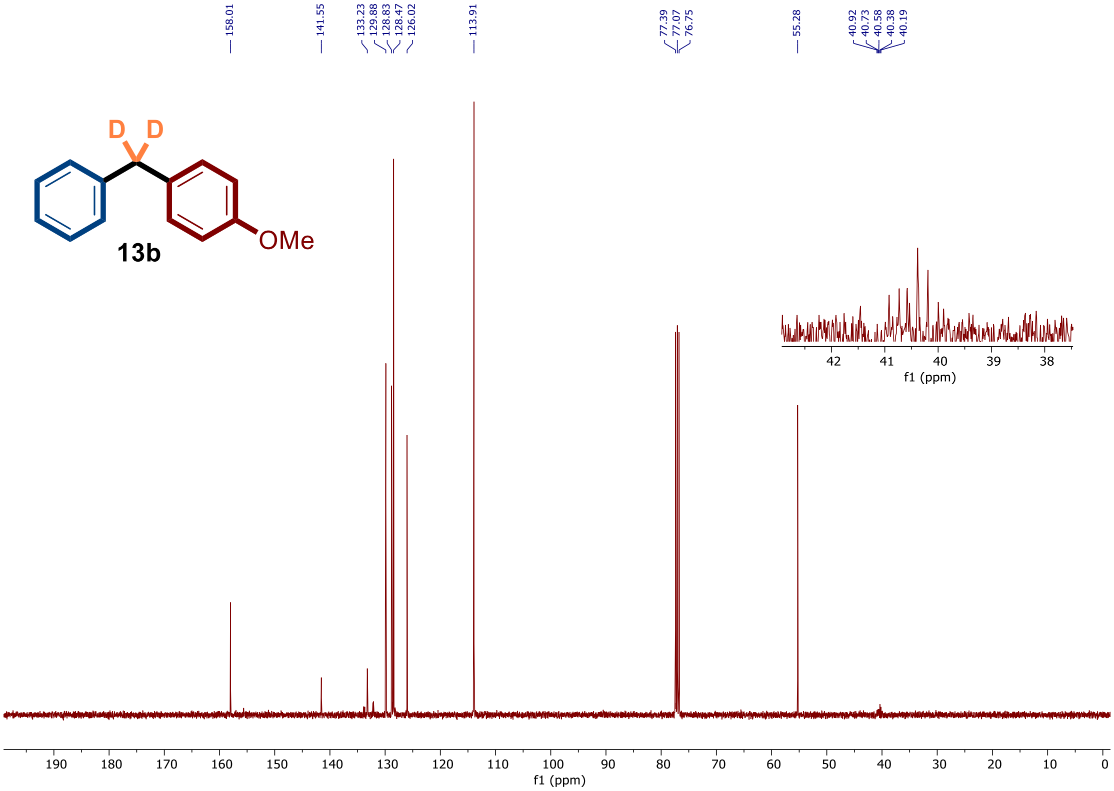

^{13}C NMR (100 MHz, $CDCl_3$) of compound (**13b**)

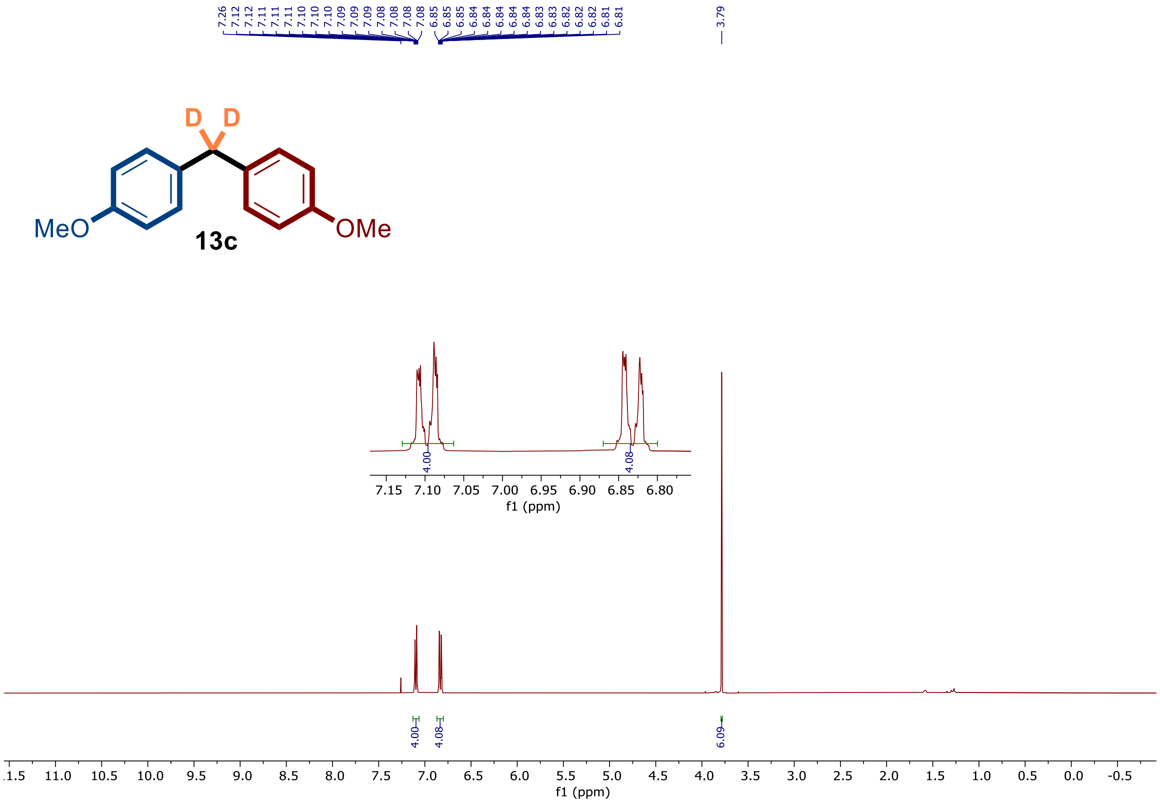

^{1}H NMR (400 MHz, $CDCl_3$) of compound (**13c**)

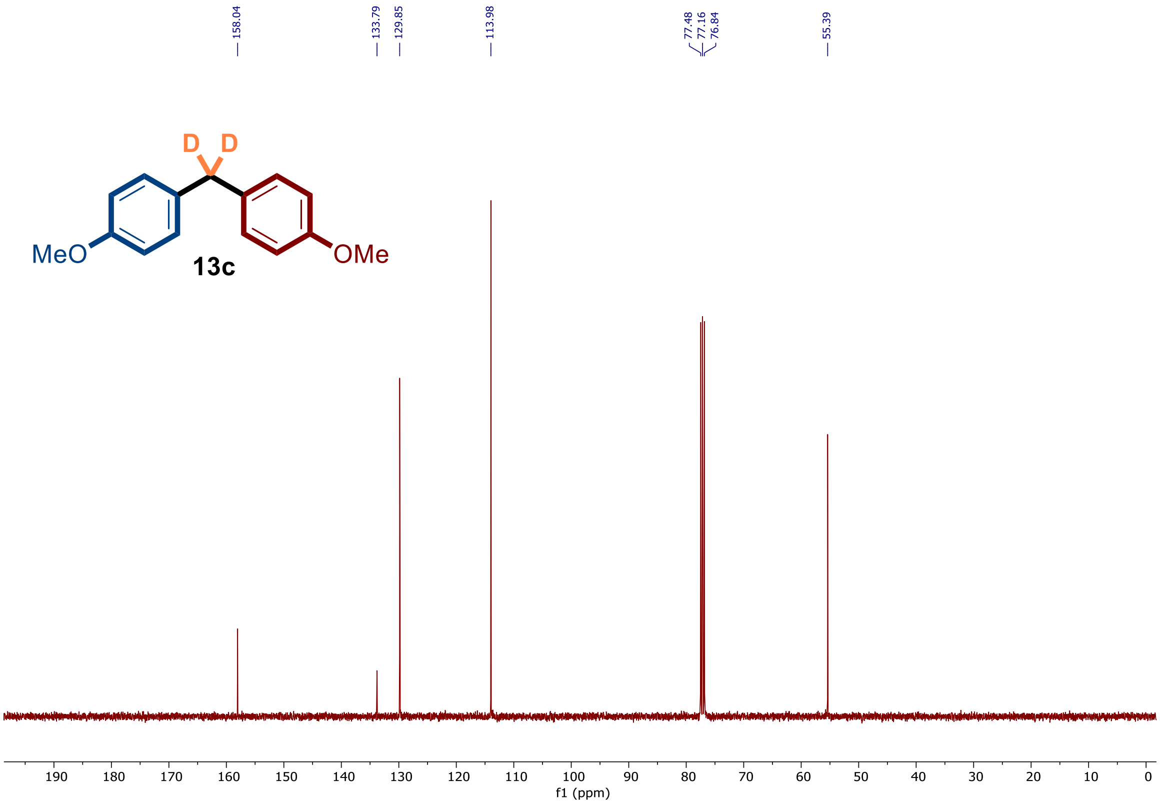

^{13}C NMR (101 MHz, $CDCl_3$) of compound (**13c**)

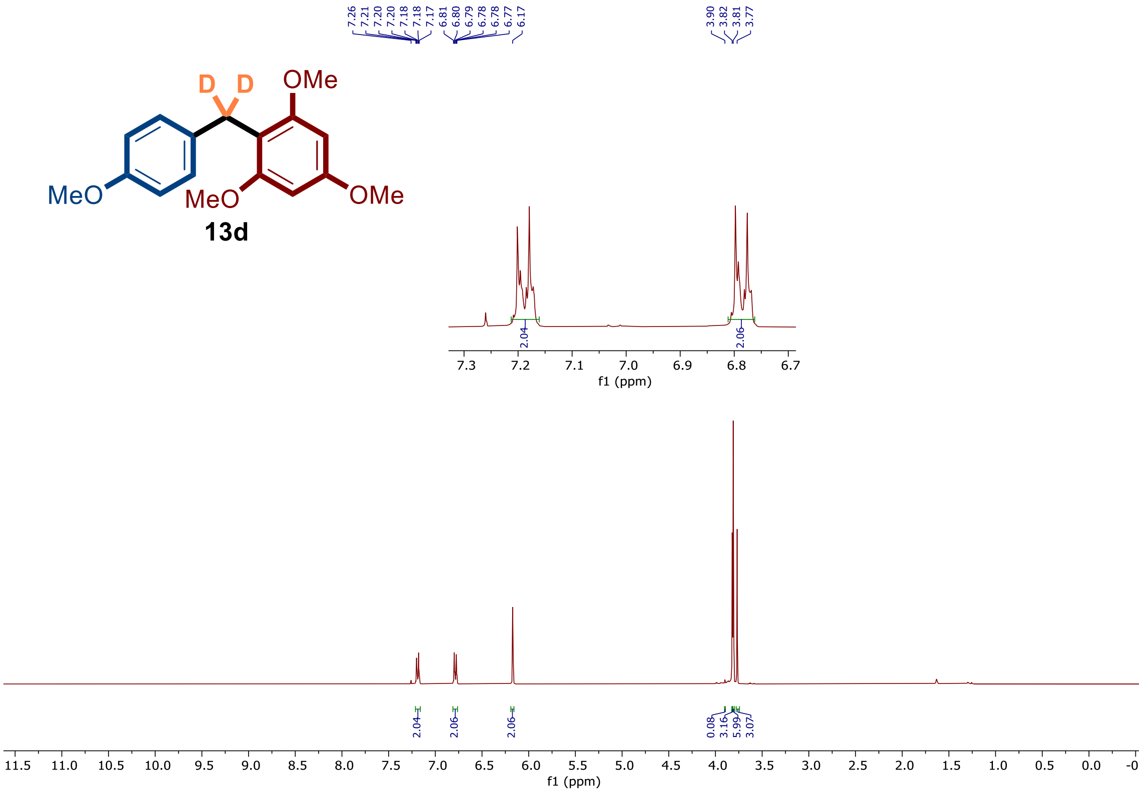

^{1}H NMR (400 MHz, $CDCl_3$) of compound (**13d**)

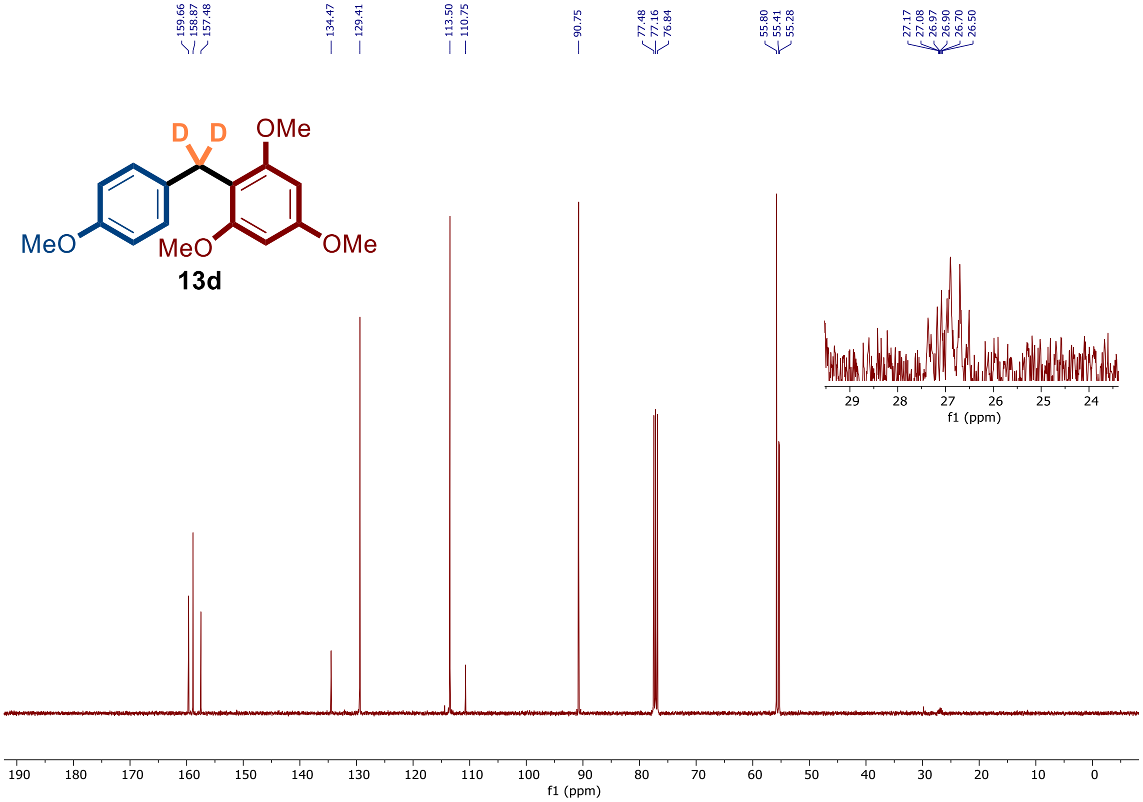

^{13}C NMR (101 MHz, $CDCl_3$) of compound (**13d**)

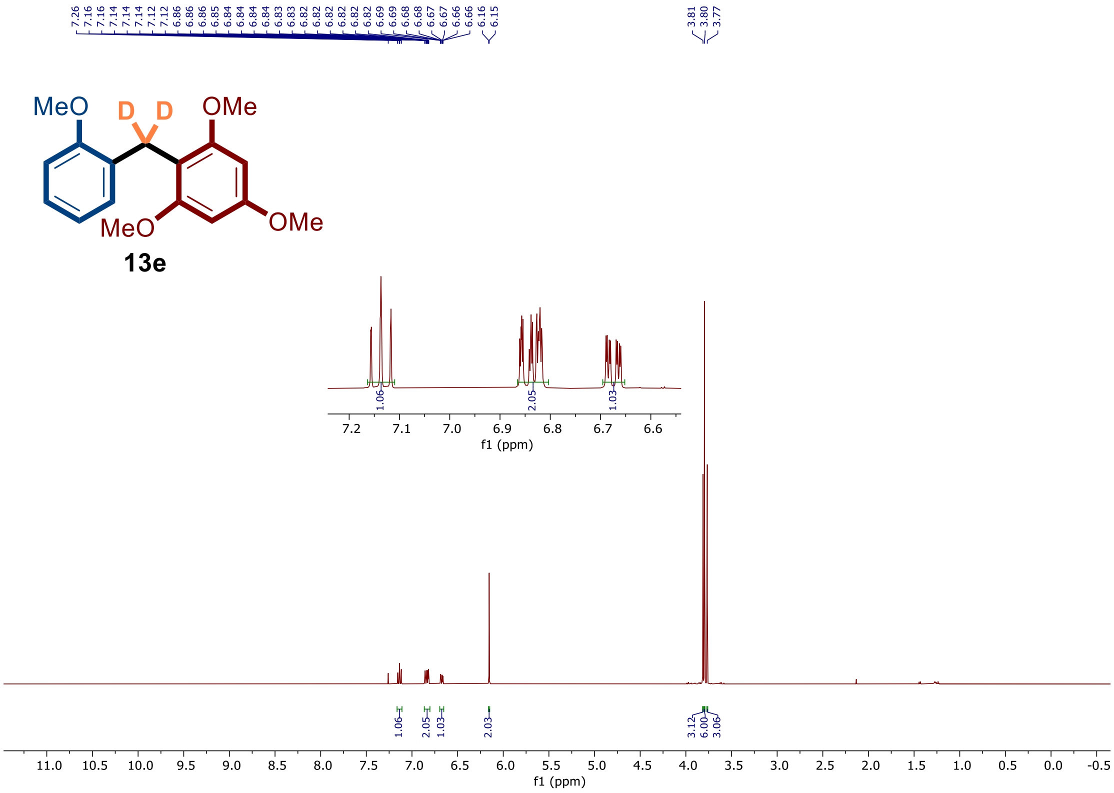

^{1}H NMR (400 MHz, $CDCl_3$) of compound (**13e**)

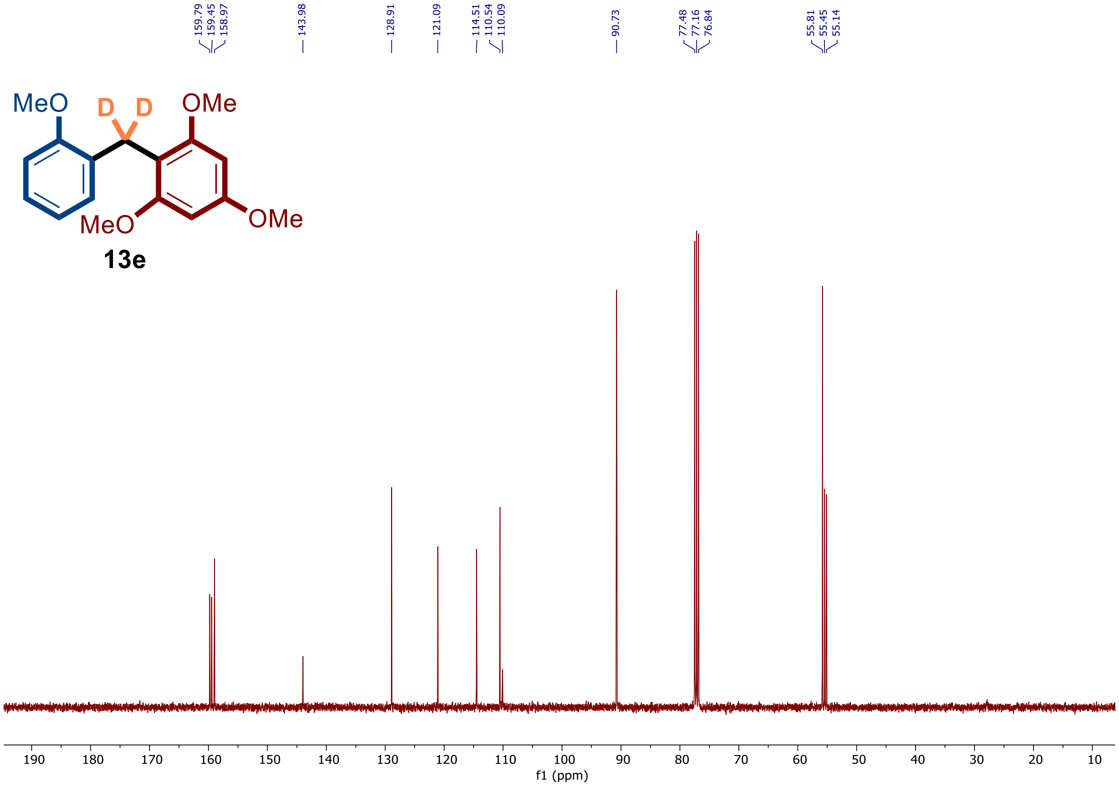

^{13}C NMR (101 MHz, $CDCl_3$) of compound (**13e**)

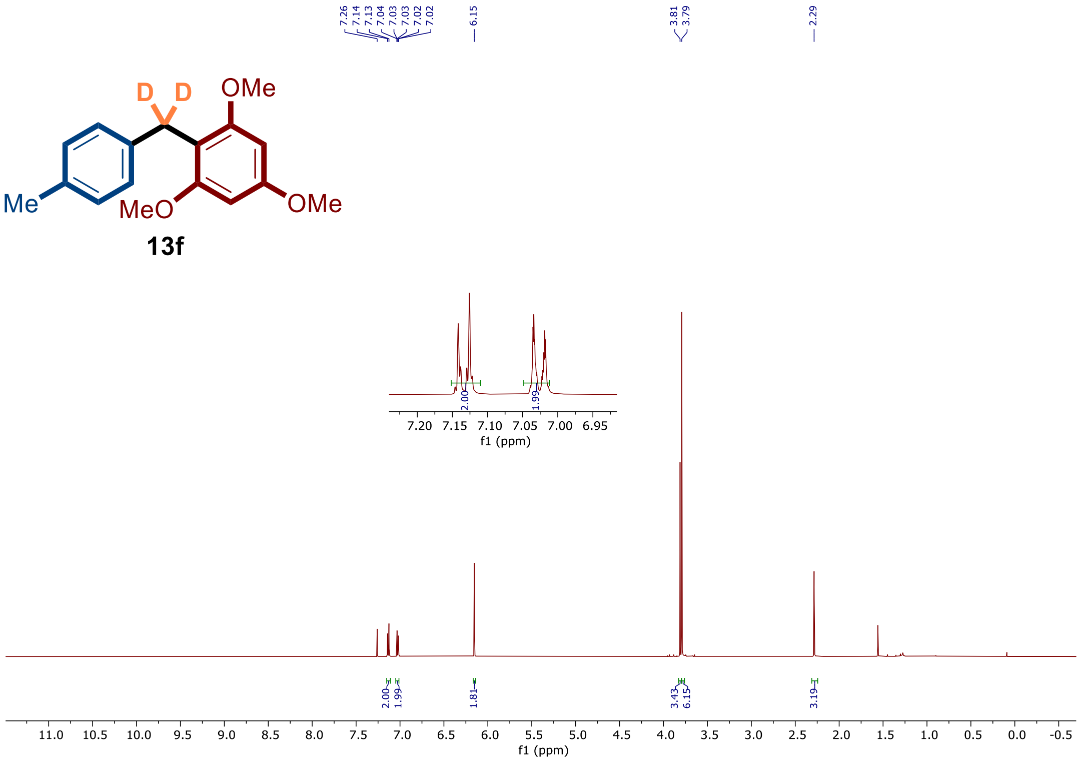

^{1}H NMR (400 MHz, CDCl$_3$) of compound (**13f**)

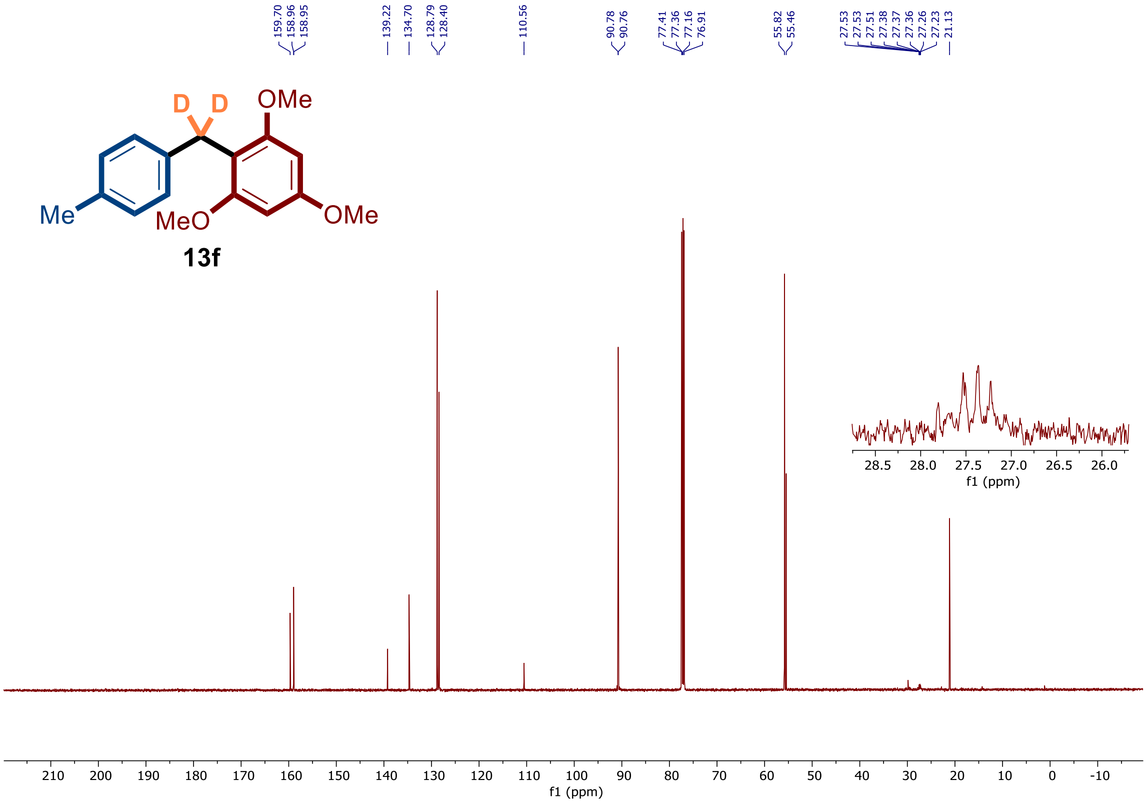

^{13}C NMR (101 MHz, CDCl$_3$) of compound (**13f**)

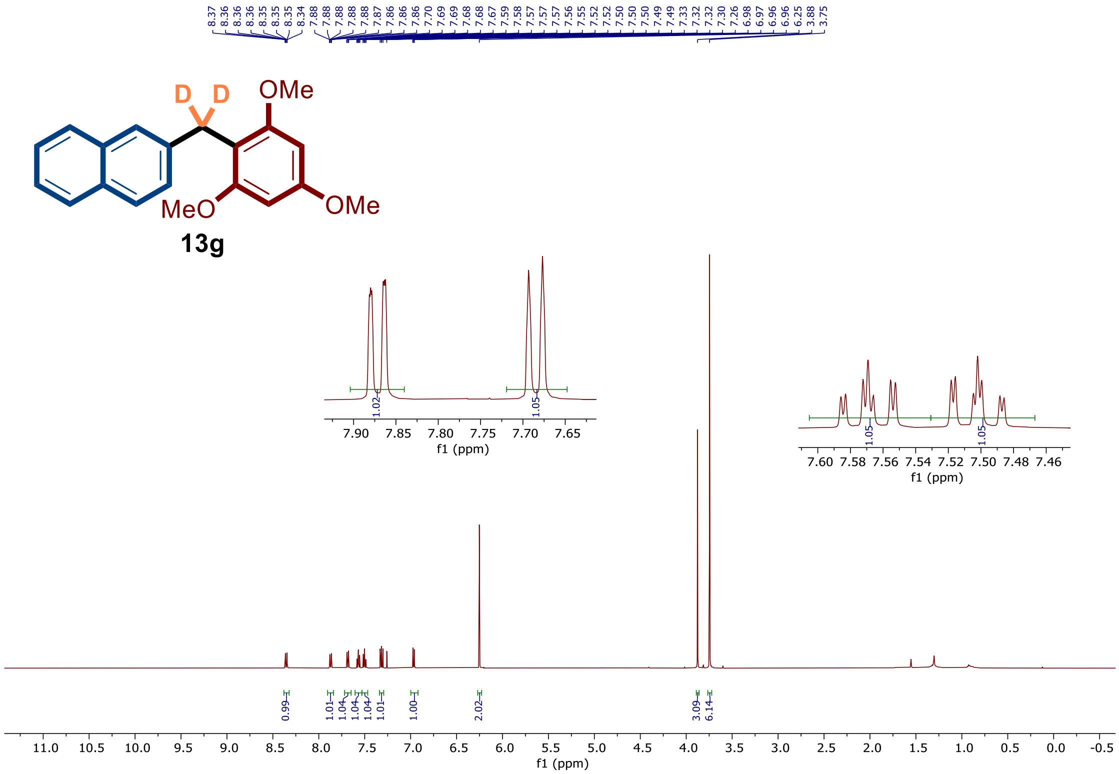

^{1}H NMR (400 MHz, $CDCl_3$) of compound (**13g**)

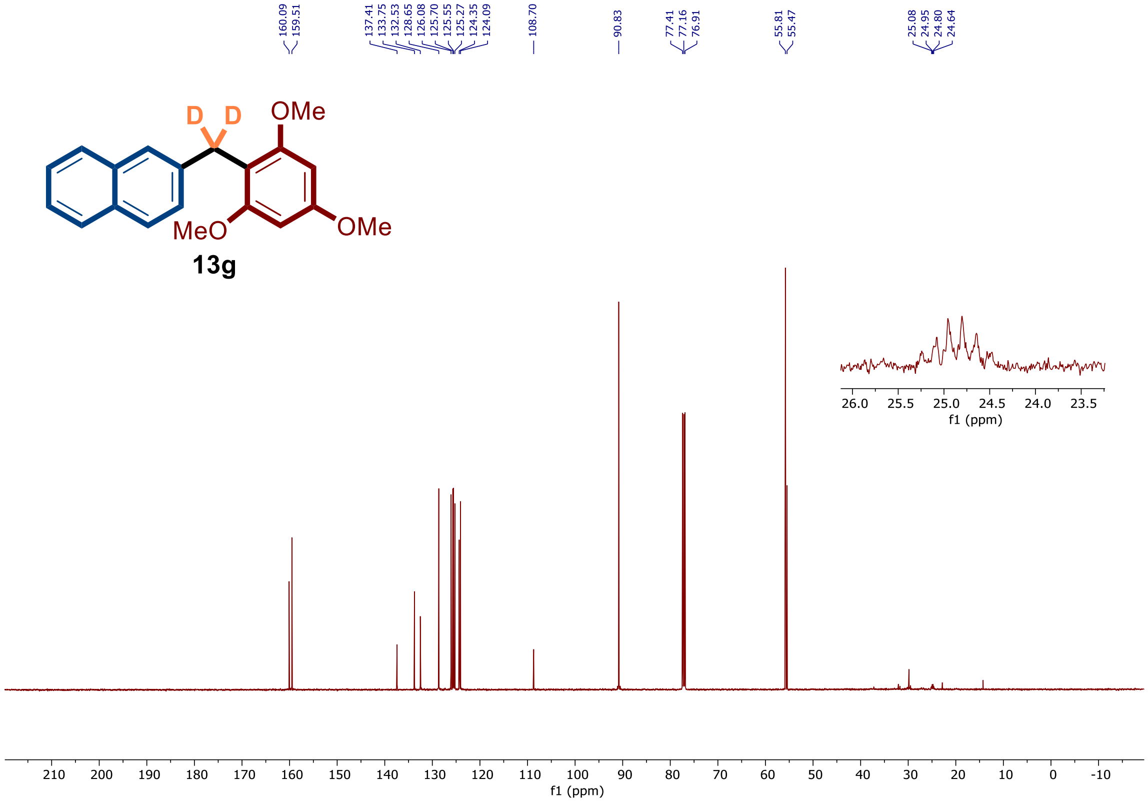

^{13}C NMR (101 MHz, $CDCl_3$) of compound (**13g**)

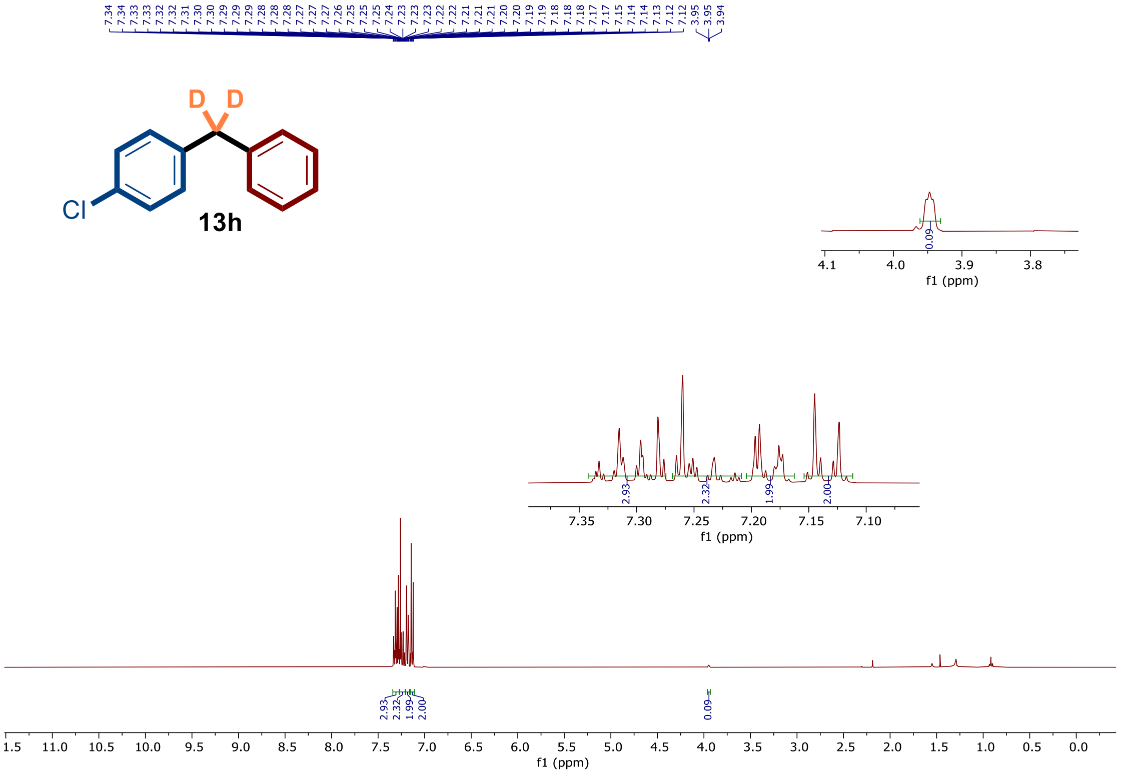

^{1}H NMR (400 MHz, $CDCl_3$) of compound (**13h**)

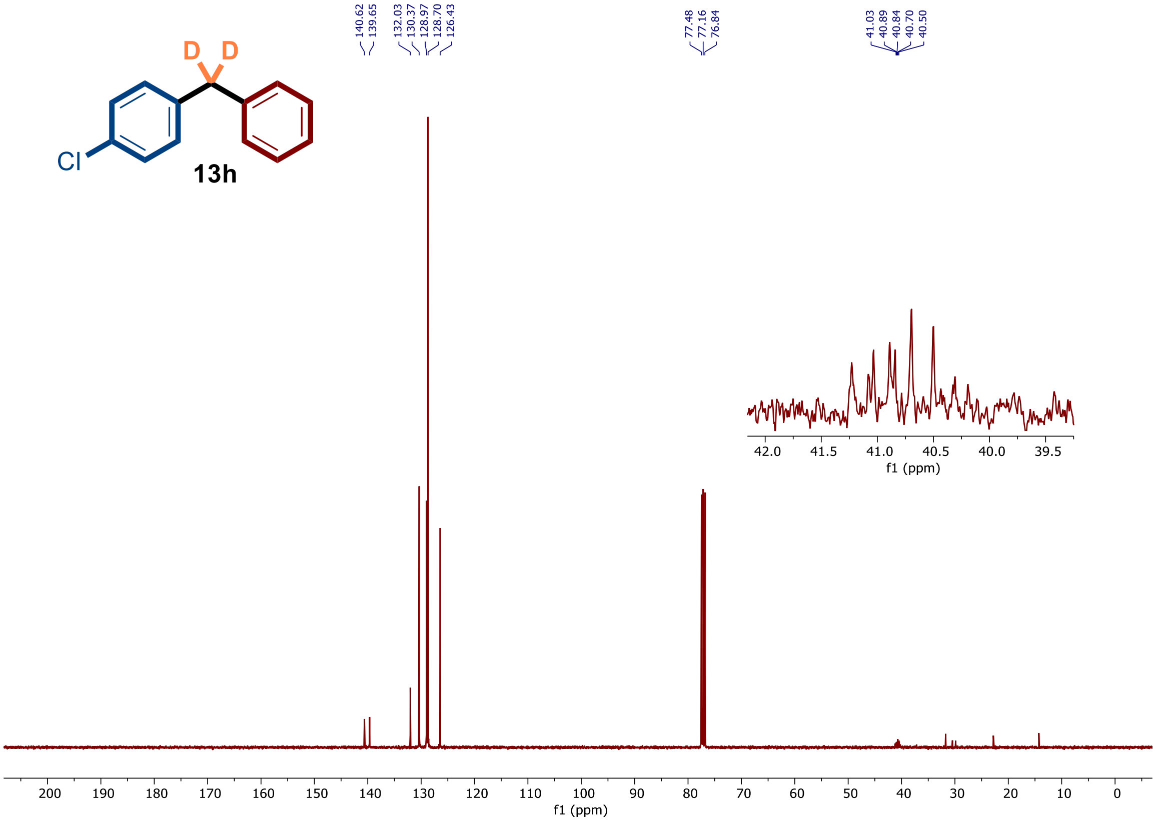

^{13}C NMR (101 MHz, $CDCl_3$) of compound (**13h**)

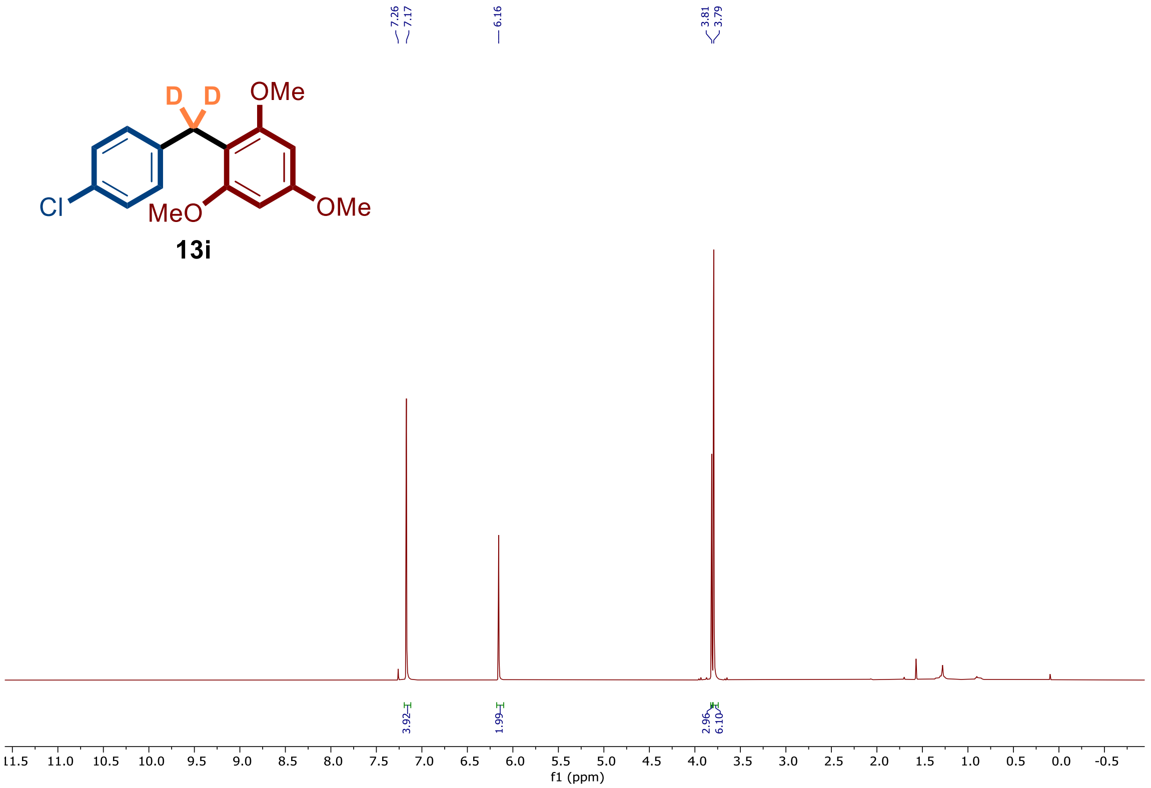

^{1}H NMR (400 MHz, $CDCl_3$) of compound (**13i**)

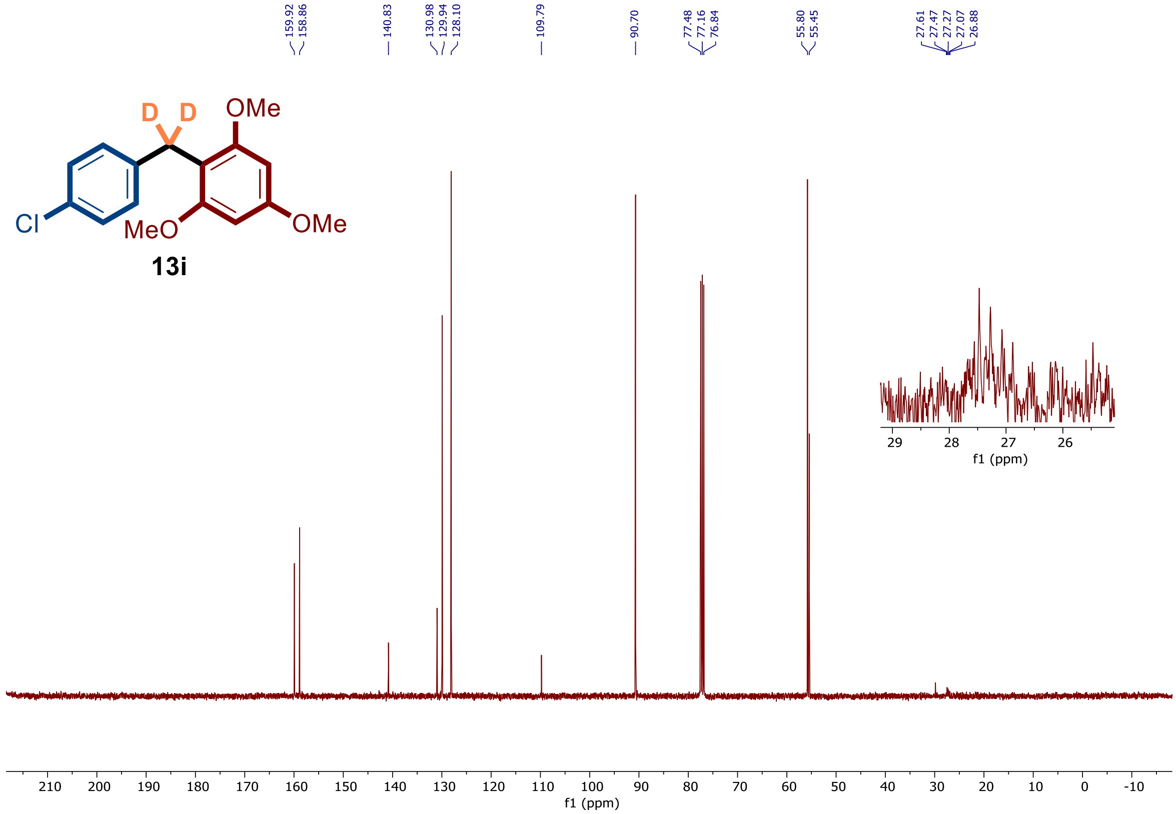

^{13}C NMR (101 MHz, $CDCl_3$) of compound (**13i**)

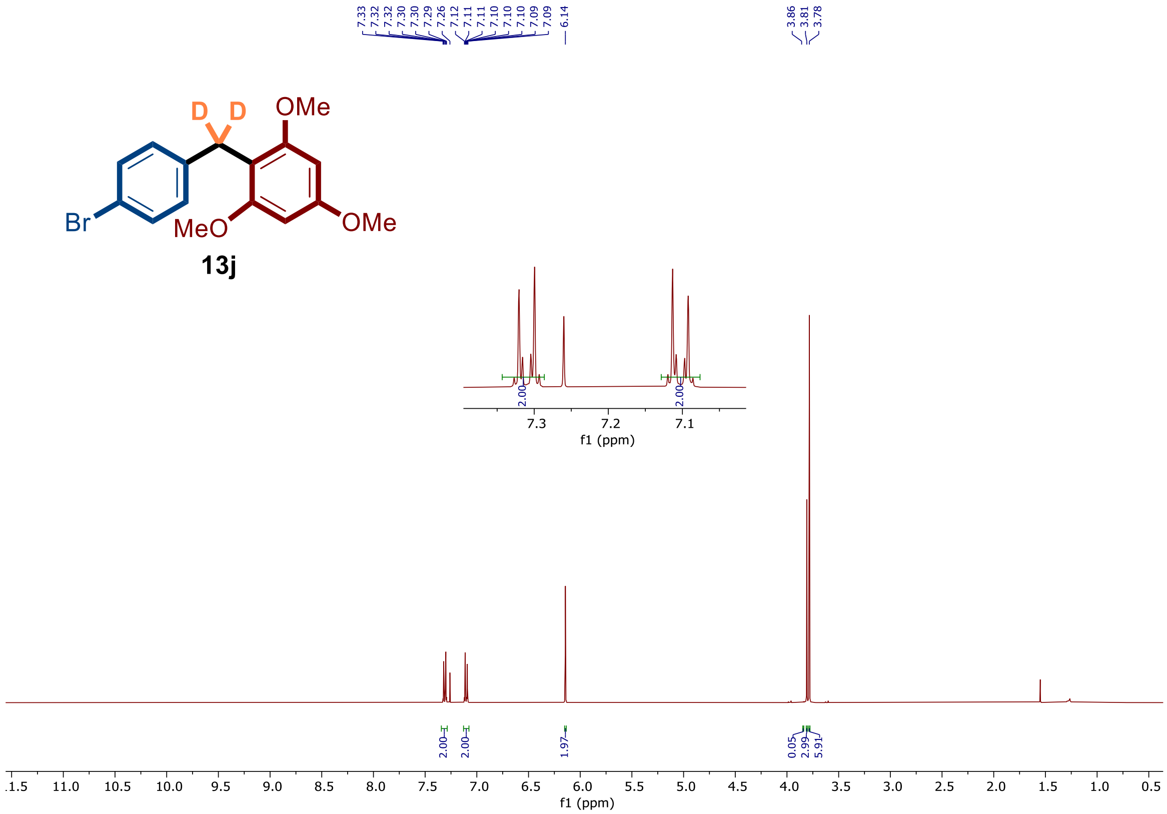

^{1}H NMR (400 MHz, $CDCl_3$) of compound (**13j**)

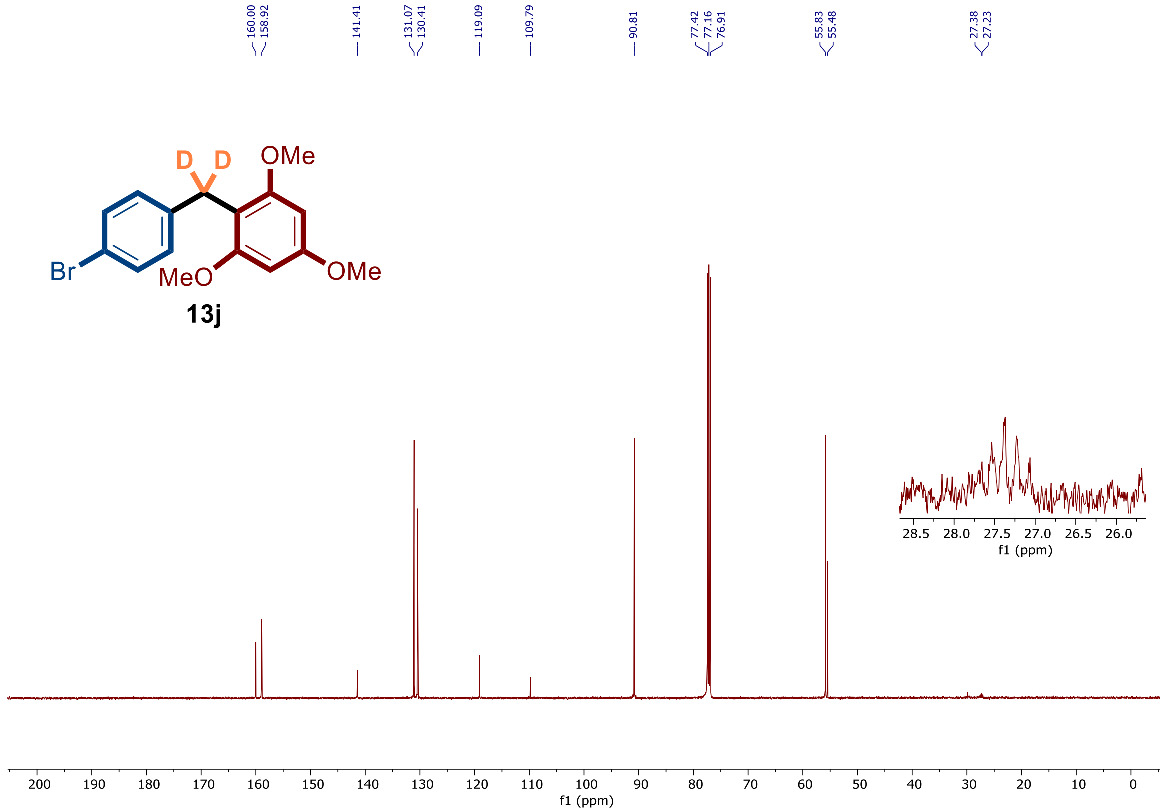

^{13}C NMR (101 MHz, $CDCl_3$) of compound (**13j**)

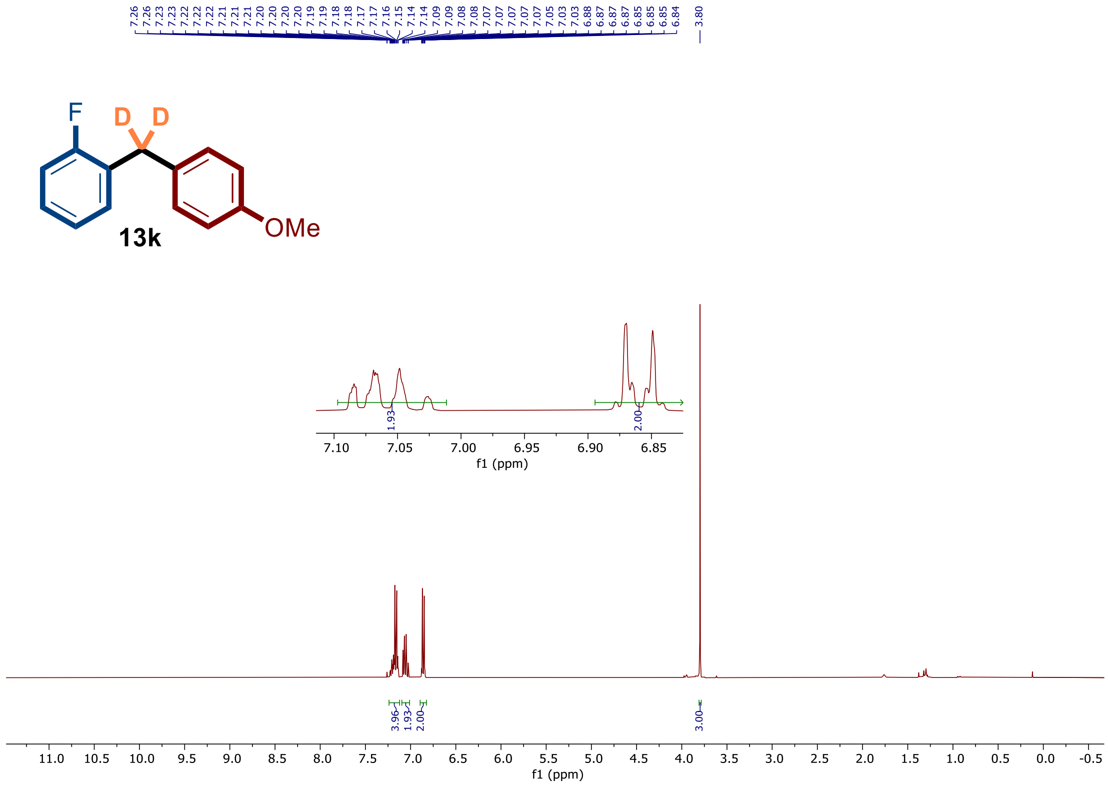

^{1}H NMR (400 MHz, $CDCl_3$) of compound (**13k**)

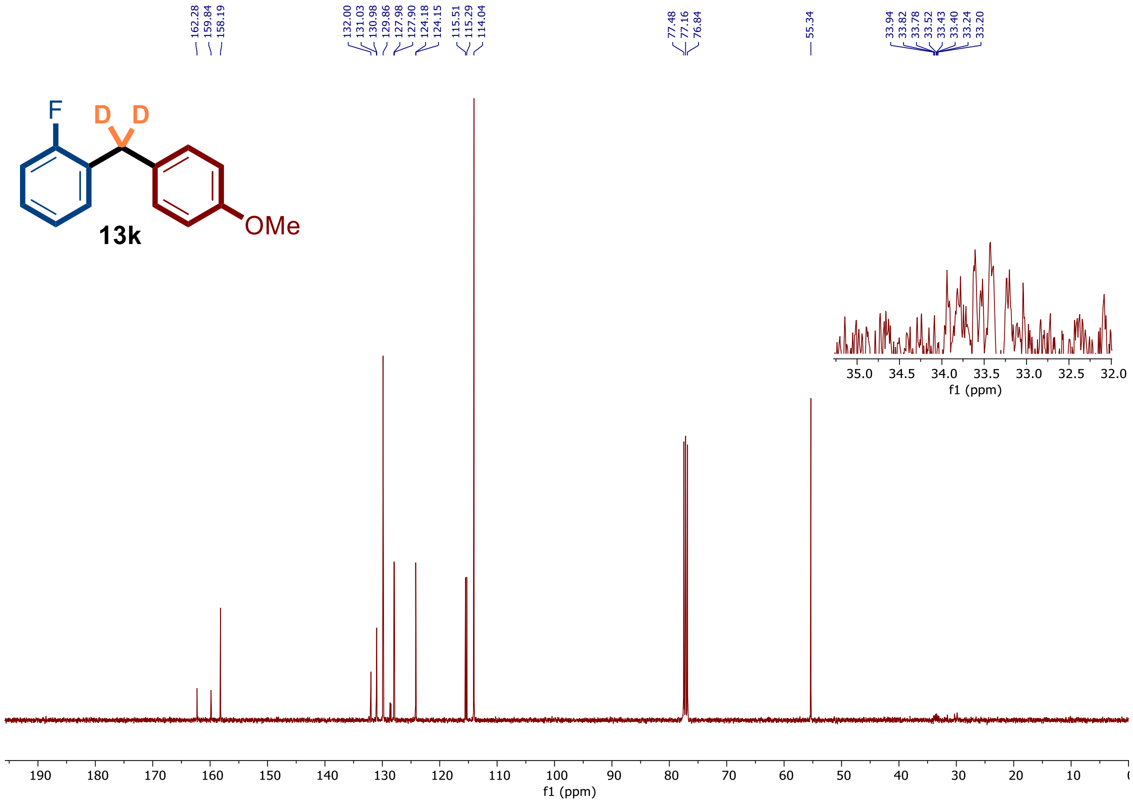

^{13}C NMR (101 MHz, $CDCl_3$) of compound (**13k**)

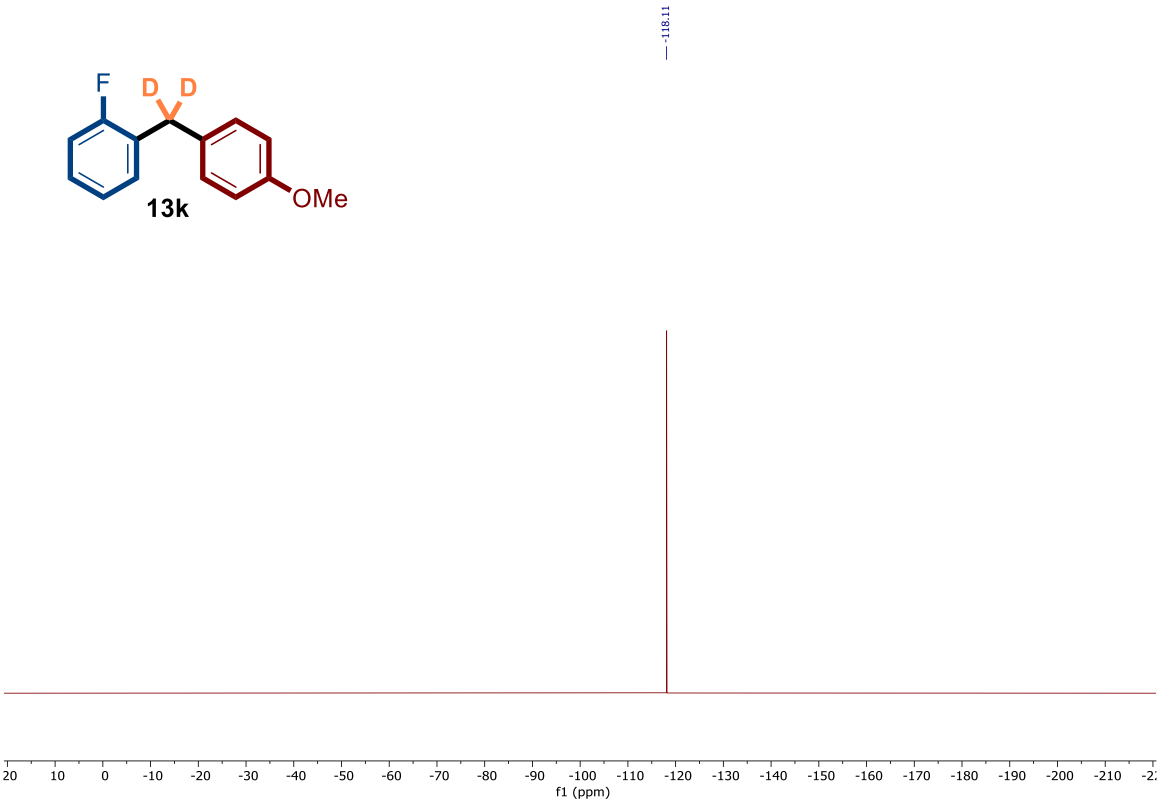

^{19}F NMR (376 MHz, $CDCl_3$) of compound (**13k**)

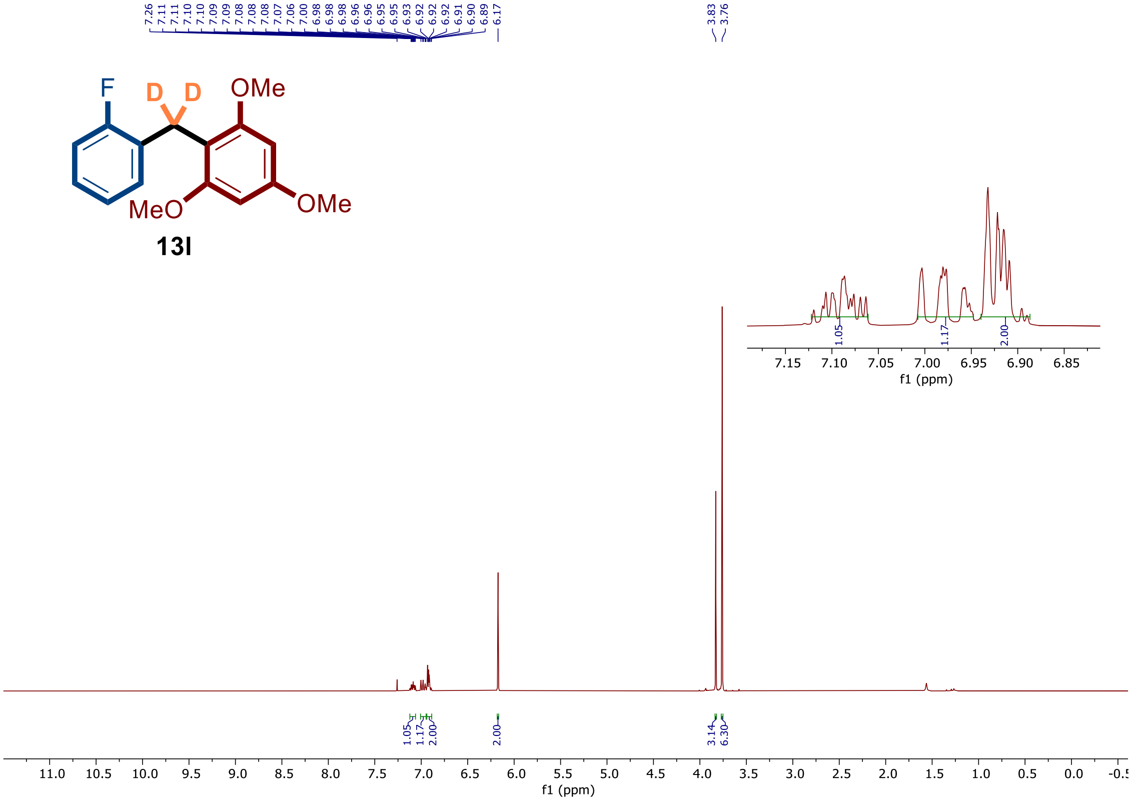

^{1}H NMR (400 MHz, $CDCl_3$) of compound (**13l**)

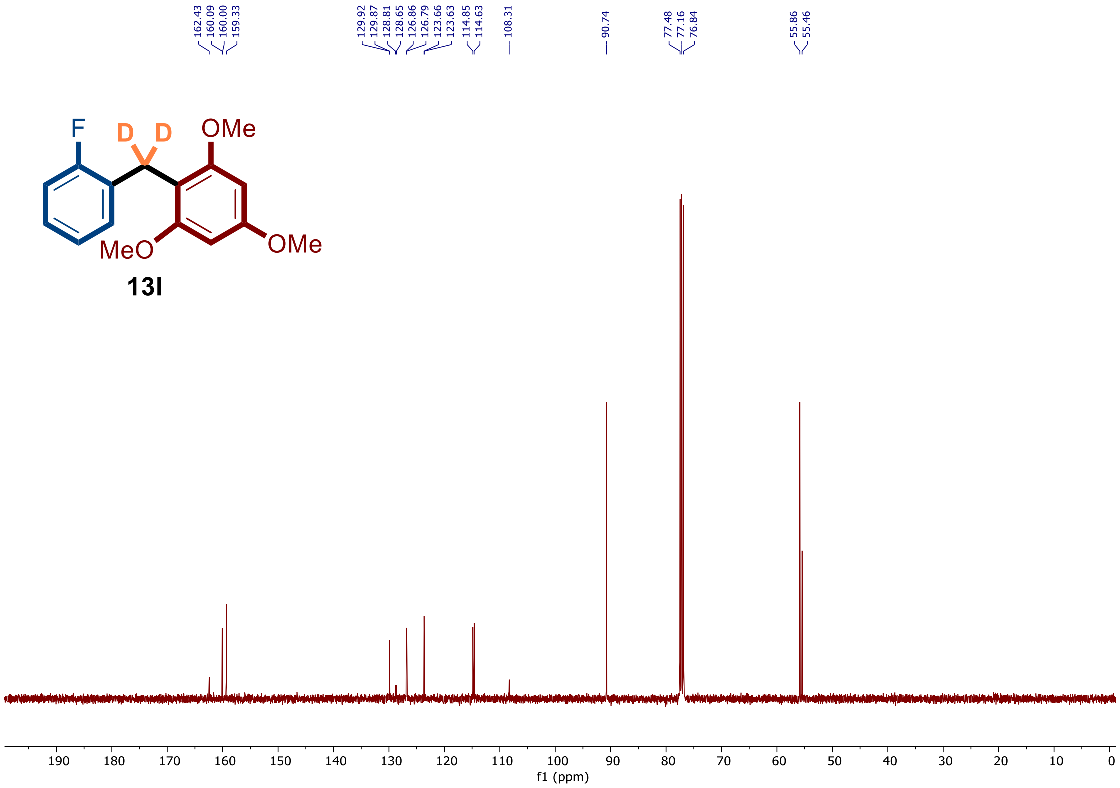

^{13}C NMR (101 MHz, $CDCl_3$) of compound (**13l**)

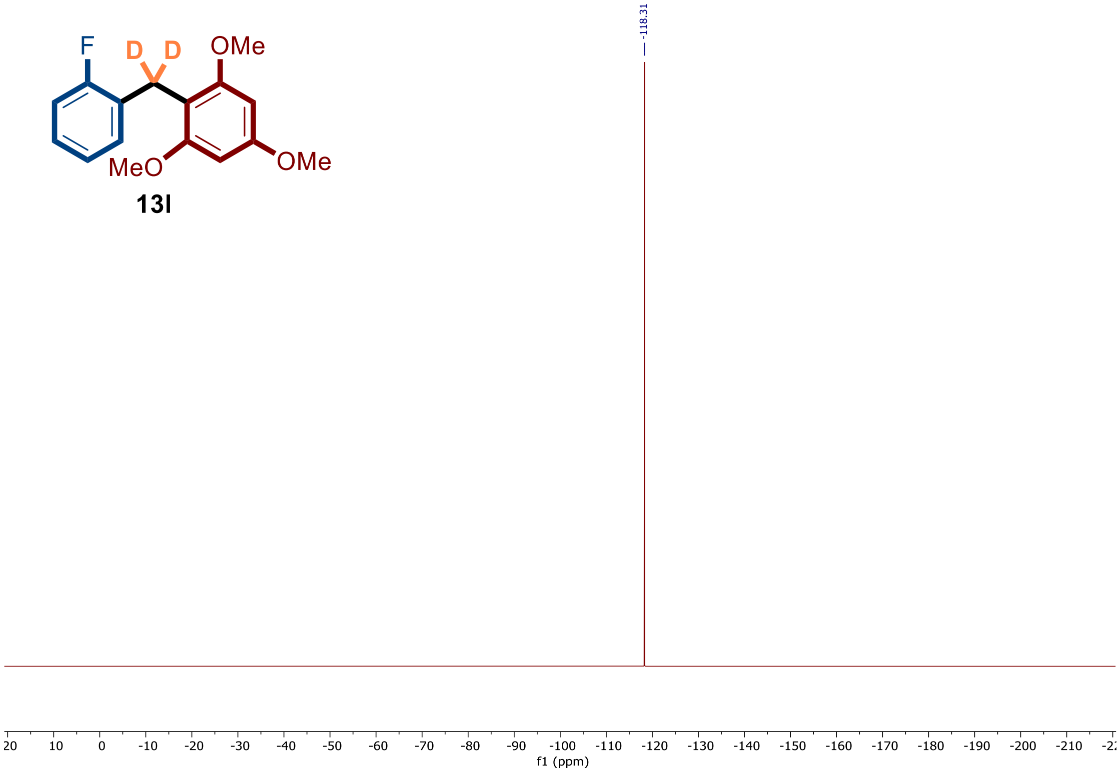

^{19}F NMR (376 MHz, $CDCl_3$) of compound (**13l**)

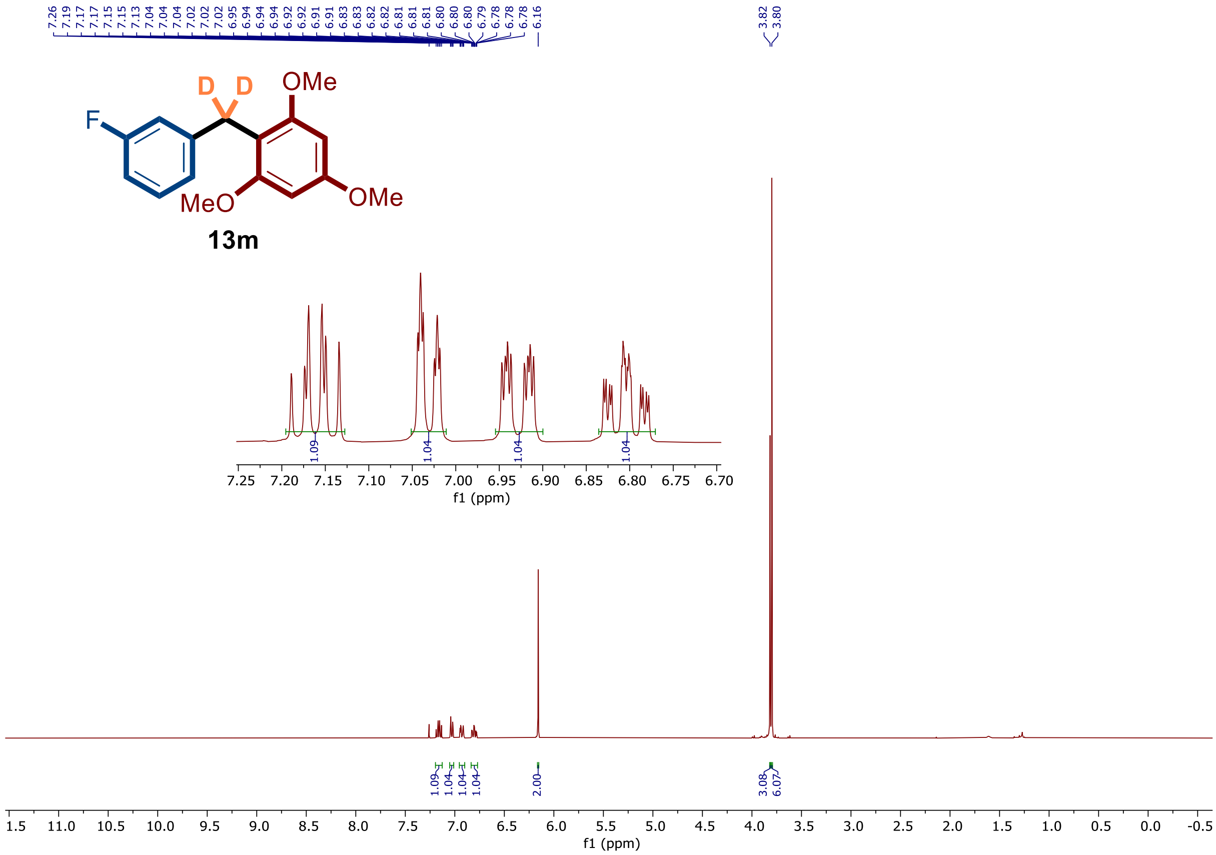

^{1}H NMR (400 MHz, $CDCl_3$) of compound (**13m**)

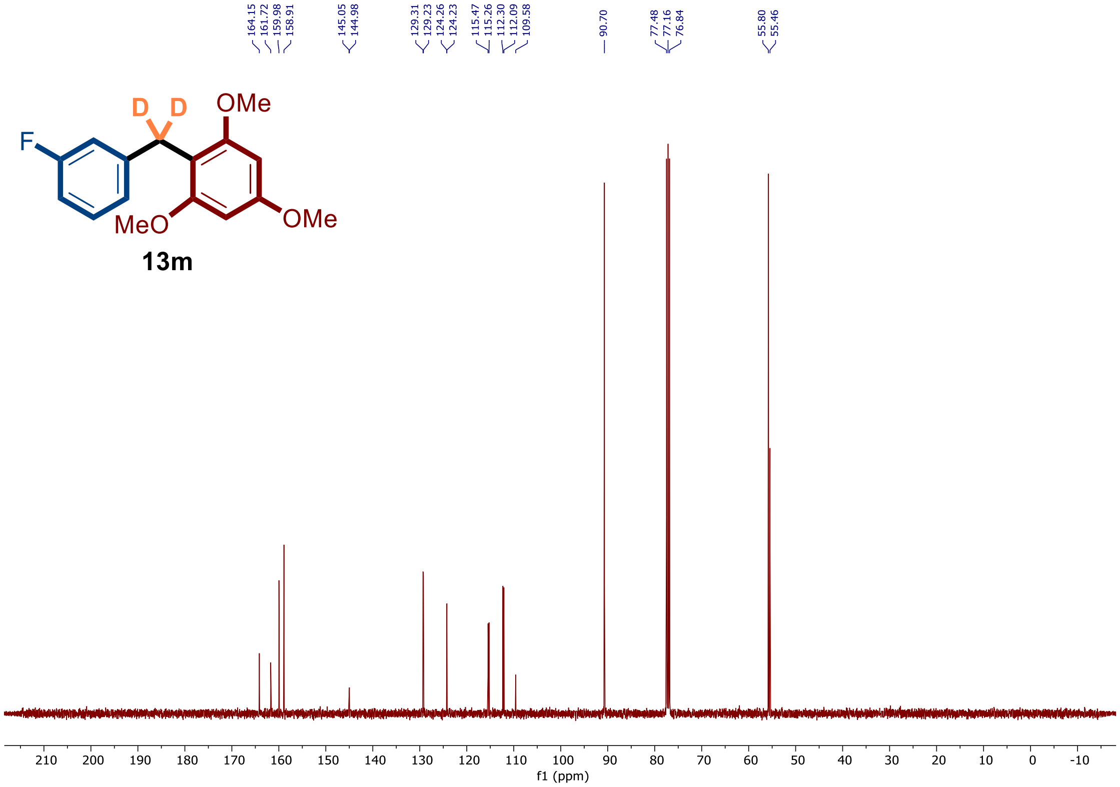

^{13}C NMR (101 MHz, $CDCl_3$) of compound (**13m**)

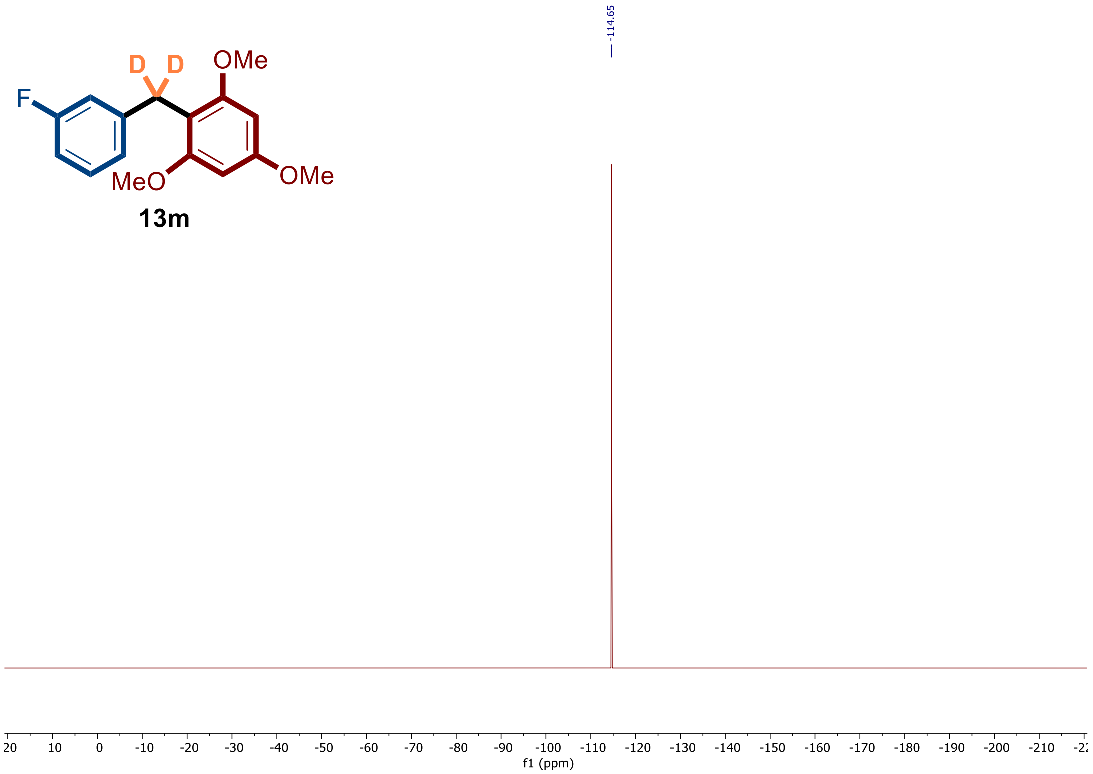

^{19}F NMR (376 MHz, $CDCl_3$) of compound (**13m**)

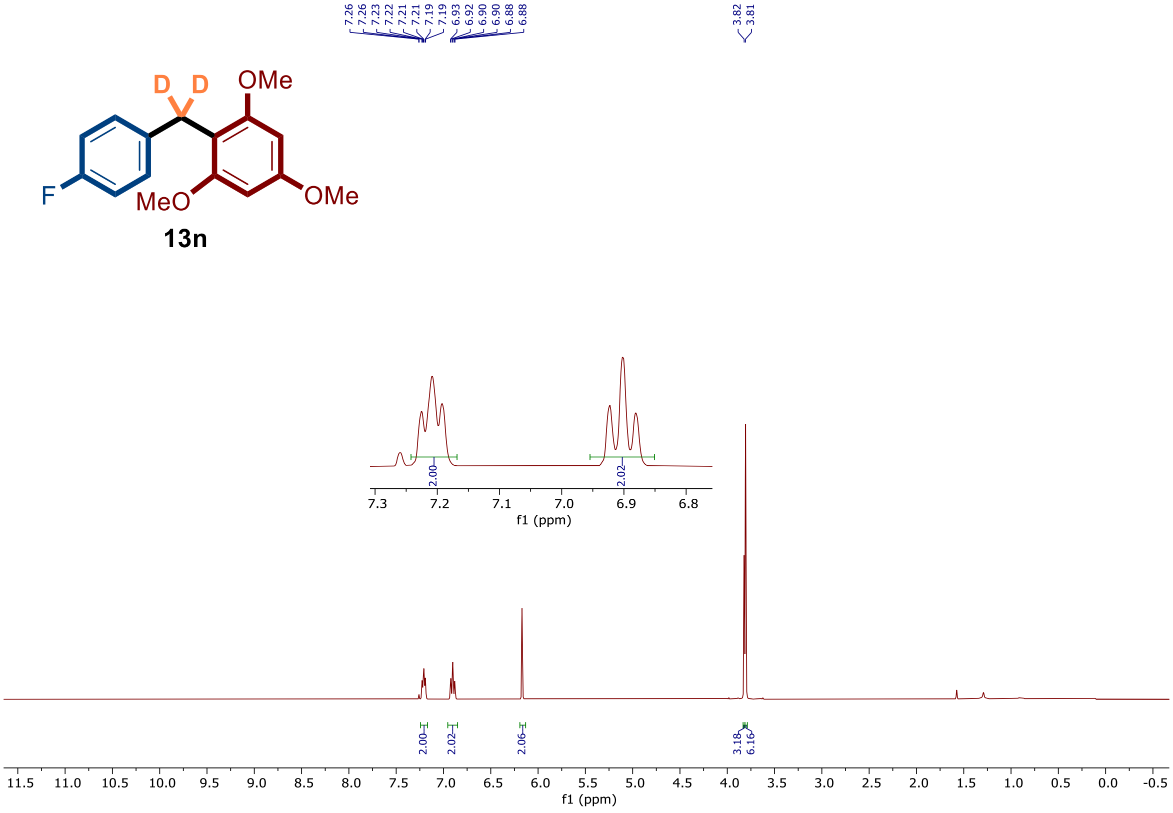

^{1}H NMR (400 MHz, $CDCl_3$) of compound (**13n**)

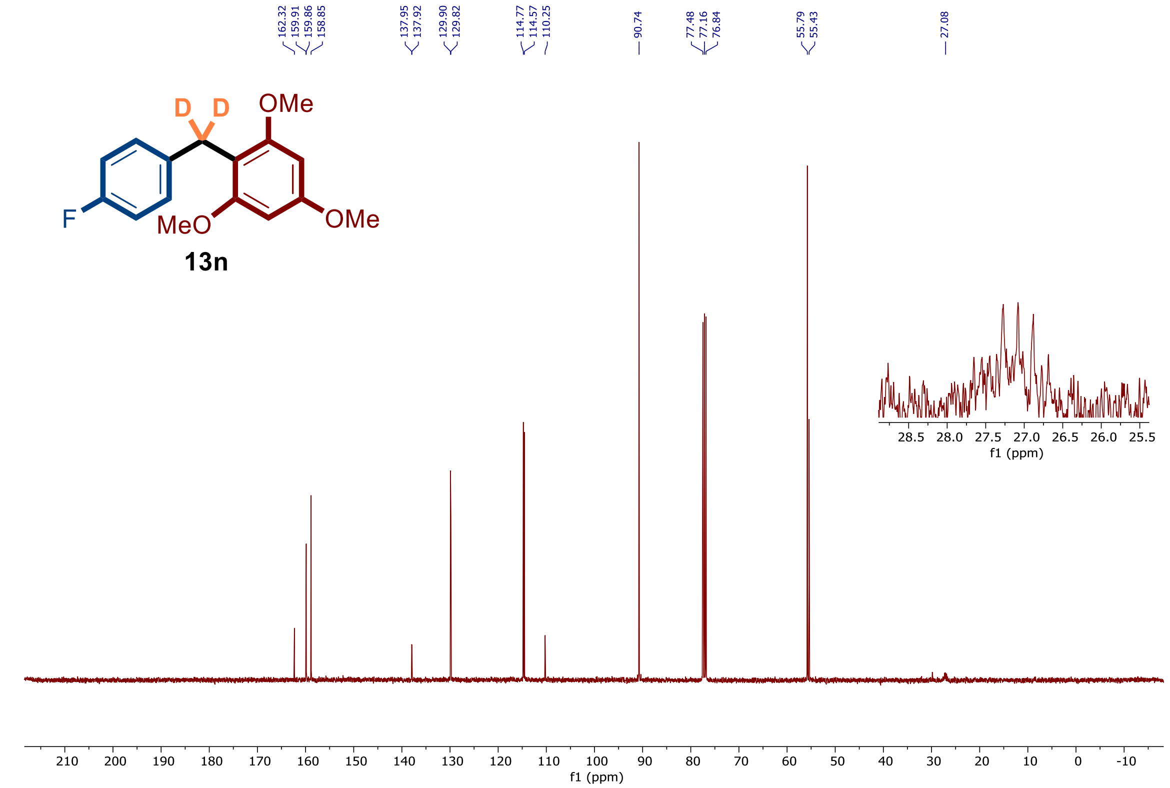

^{13}C NMR (101 MHz, $CDCl_3$) of compound (**13n**)

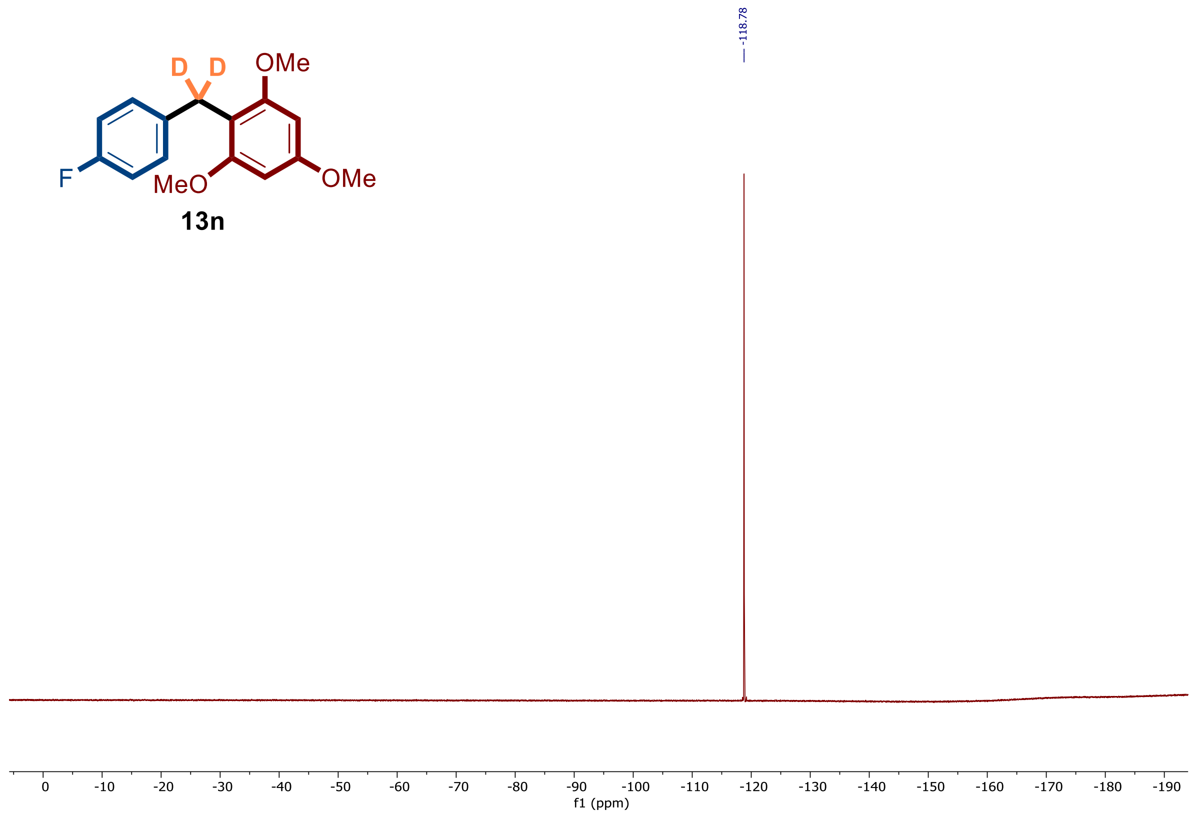

^{19}F NMR (376 MHz, $CDCl_3$) of compound (**13n**)

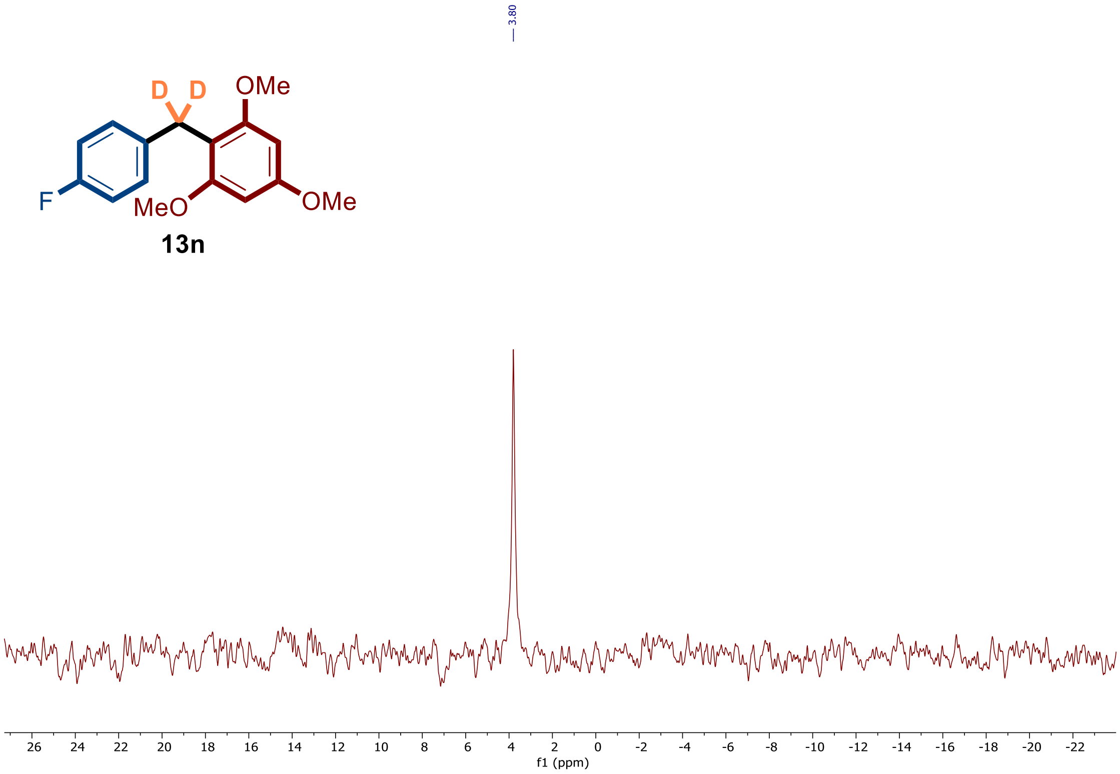

^{2}H NMR (77 MHz, $CDCl_3$) of compound (**13n**)

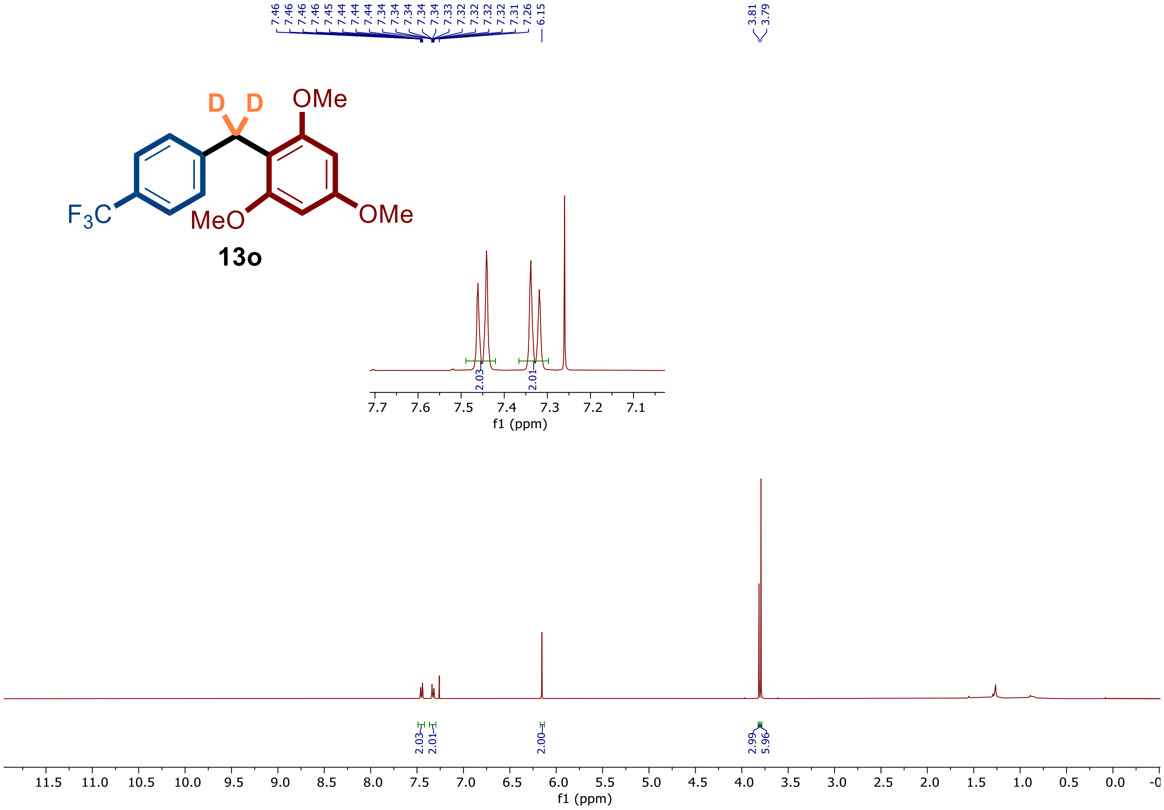

^{1}H NMR (400 MHz, $CDCl_3$) of compound (**13o**)

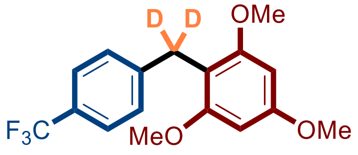

13o

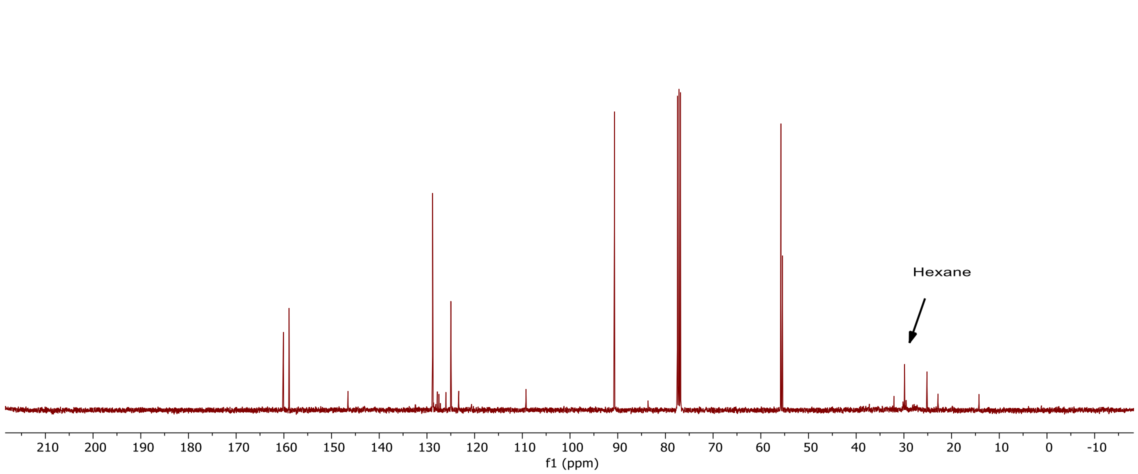

^{13}C NMR (101 MHz, $CDCl_3$) of compound (**13o**)

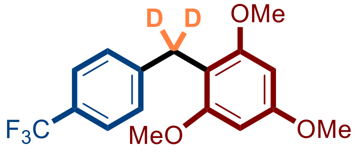

13o

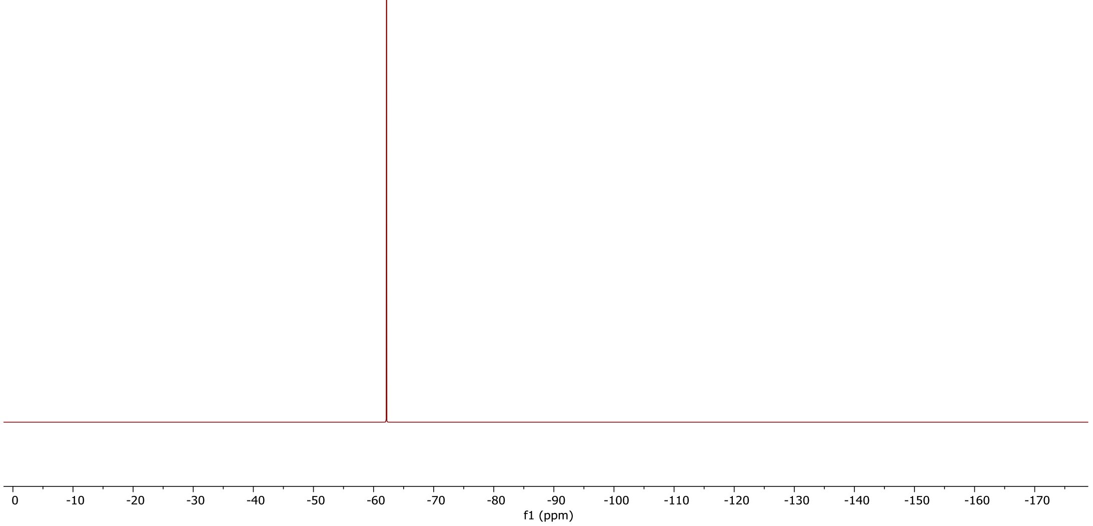

^{19}F NMR (376 MHz, $CDCl_3$) of compound (**13o**)

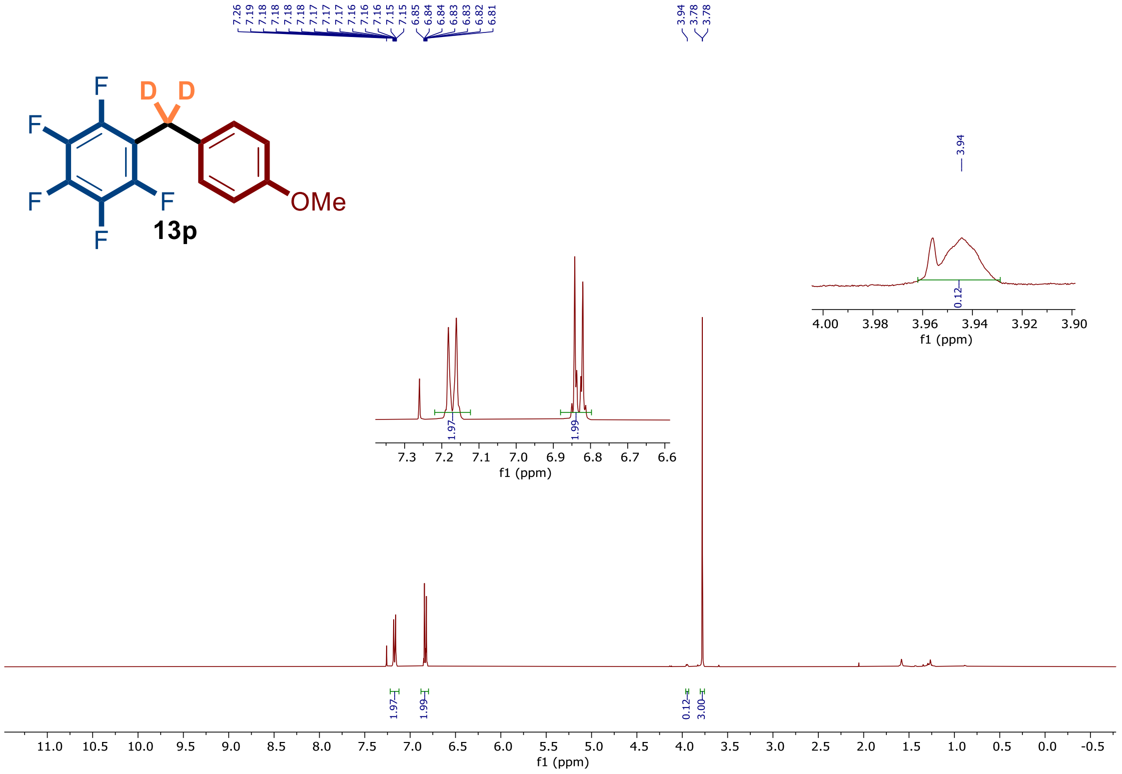

^{1}H NMR (400 MHz, $CDCl_3$) of compound (**13p**)

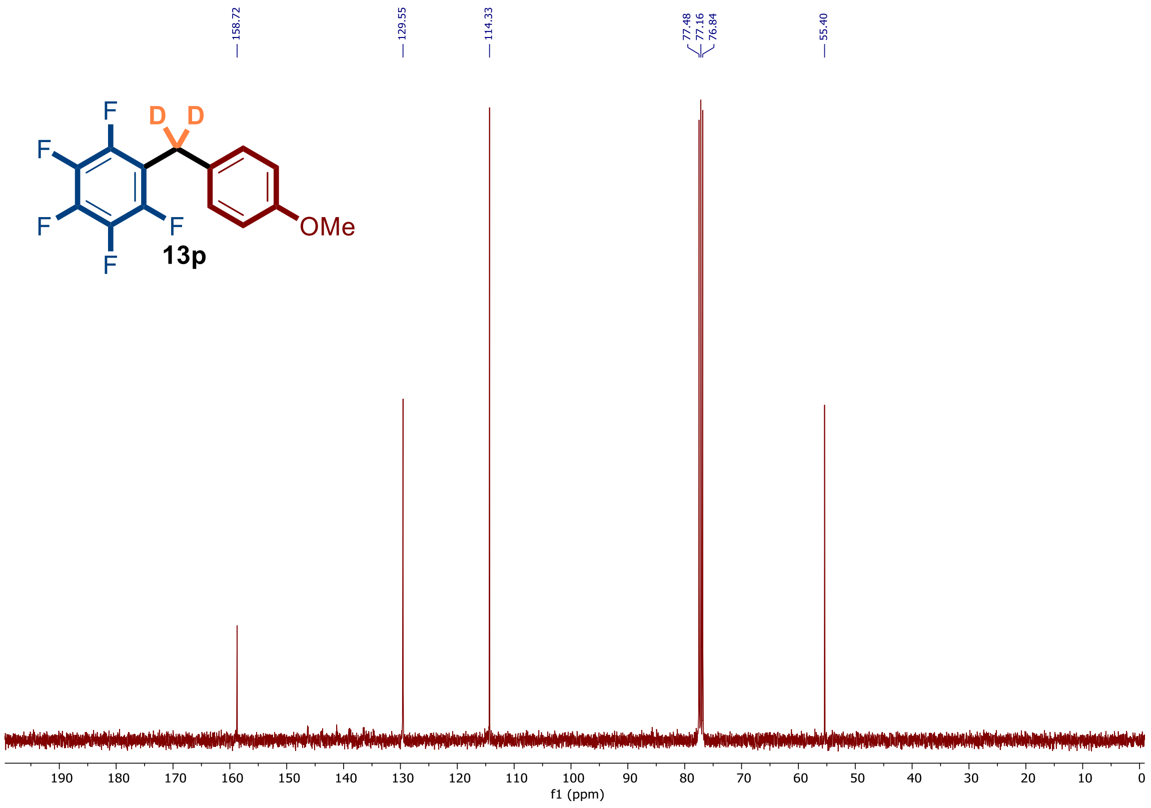

^{13}C NMR (101 MHz, $CDCl_3$) of compound (**13p**)

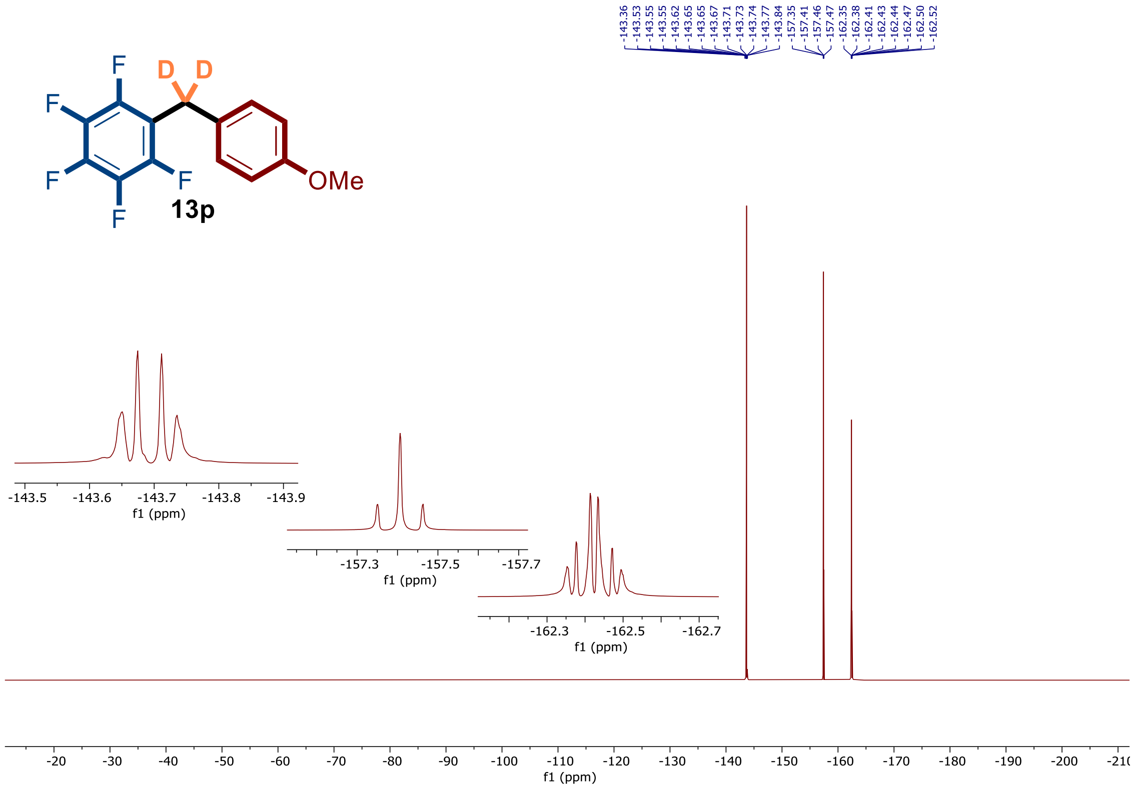

^{19}F NMR (376 MHz, CDCl$_3$) of compound (**13p**)

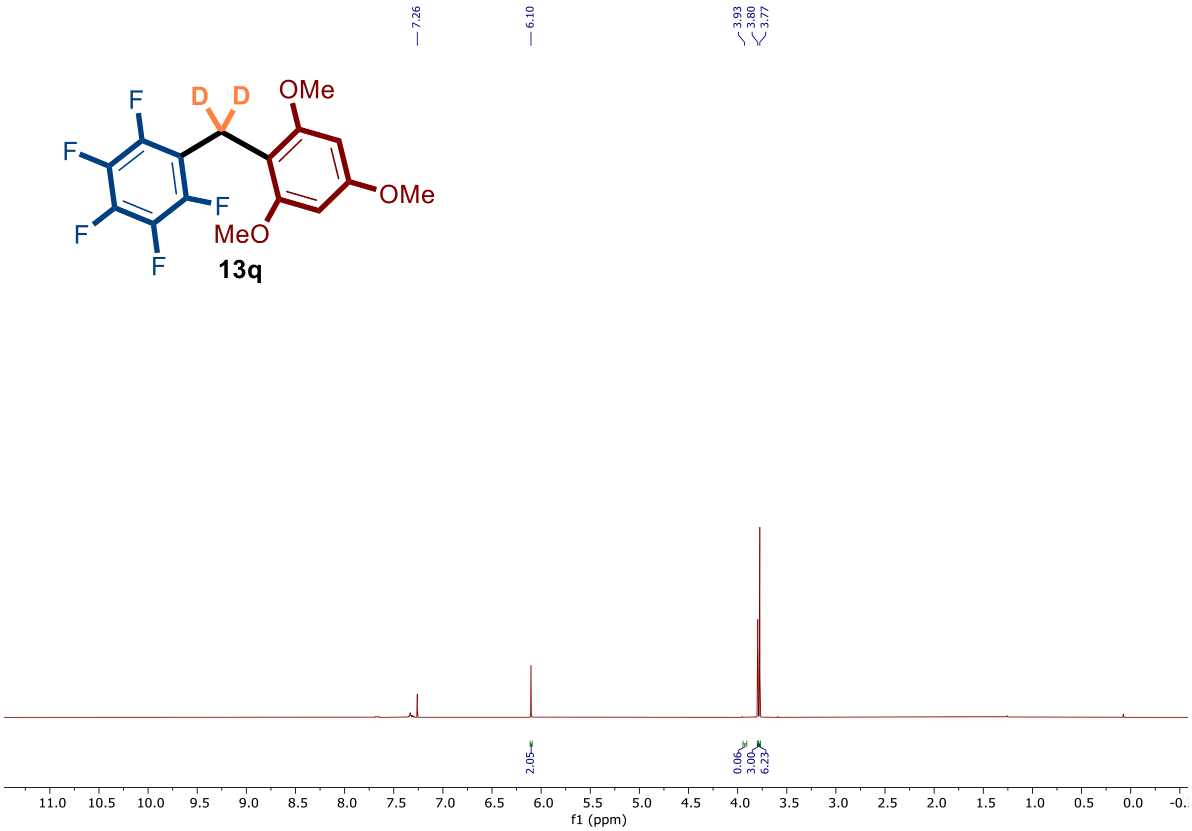

^{1}H NMR (400 MHz, CDCl$_3$) of compound (**13q**)

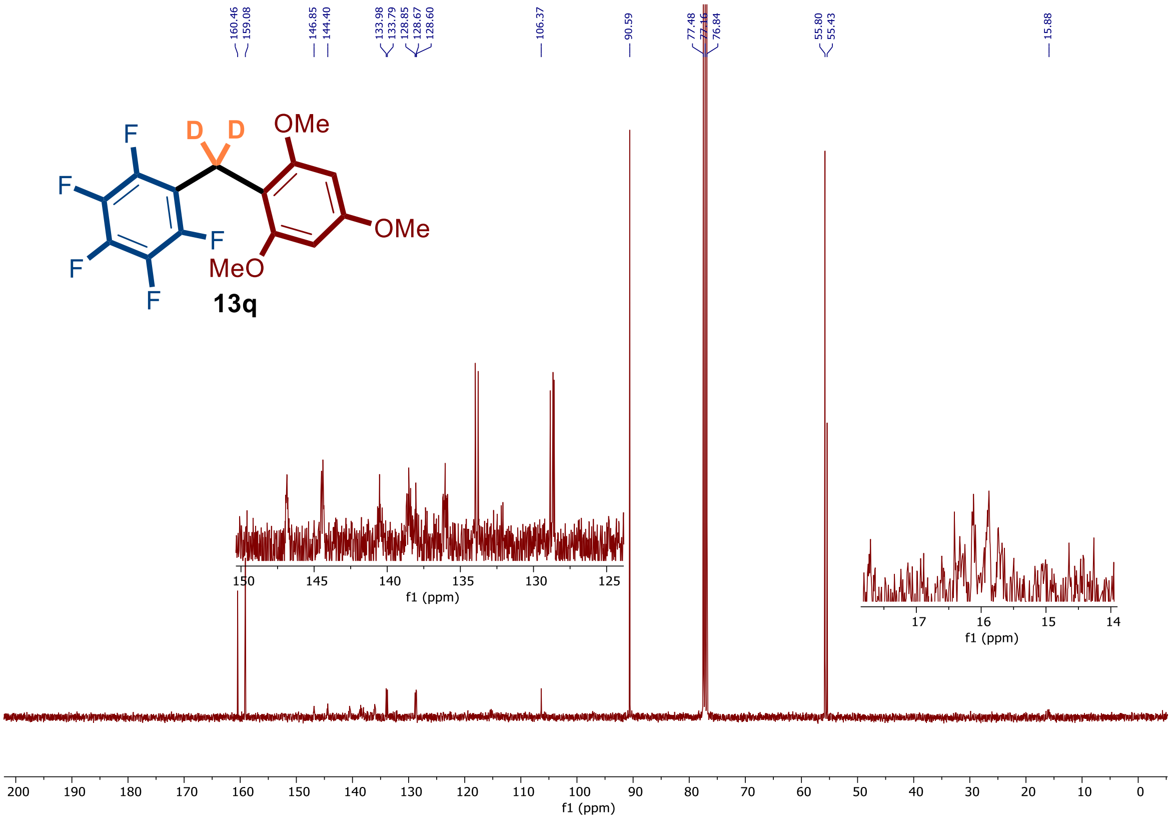

^{13}C NMR (101 MHz, $CDCl_3$) of compound (**13q**)

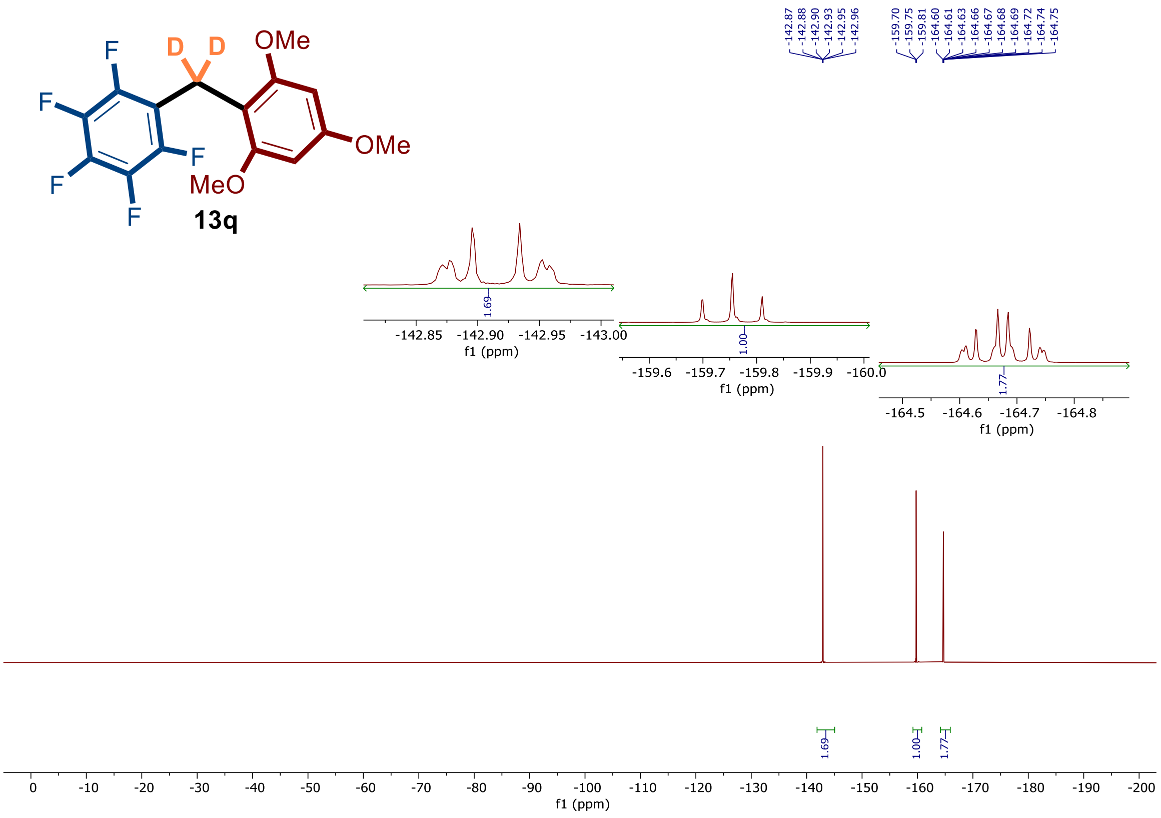

^{19}F NMR (376 MHz, $CDCl_3$) of compound (**13q**)

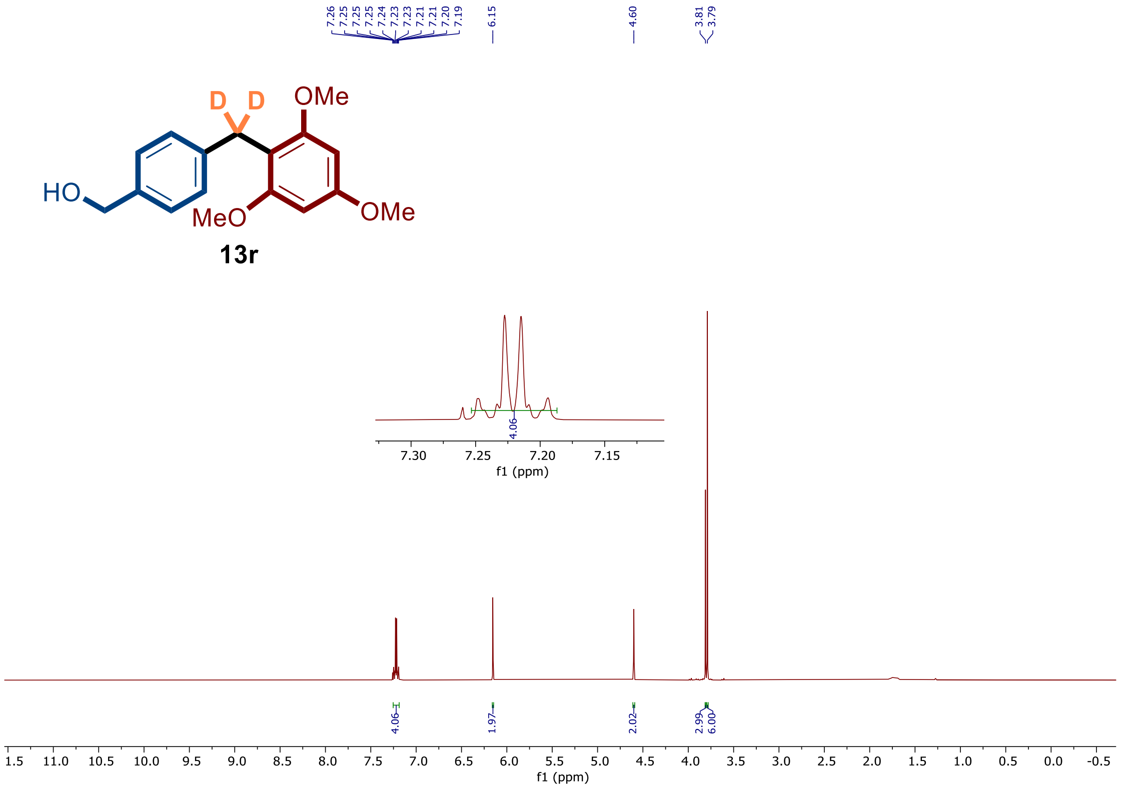

^{1}H NMR (400 MHz, $CDCl_3$) of compound (**13r**)

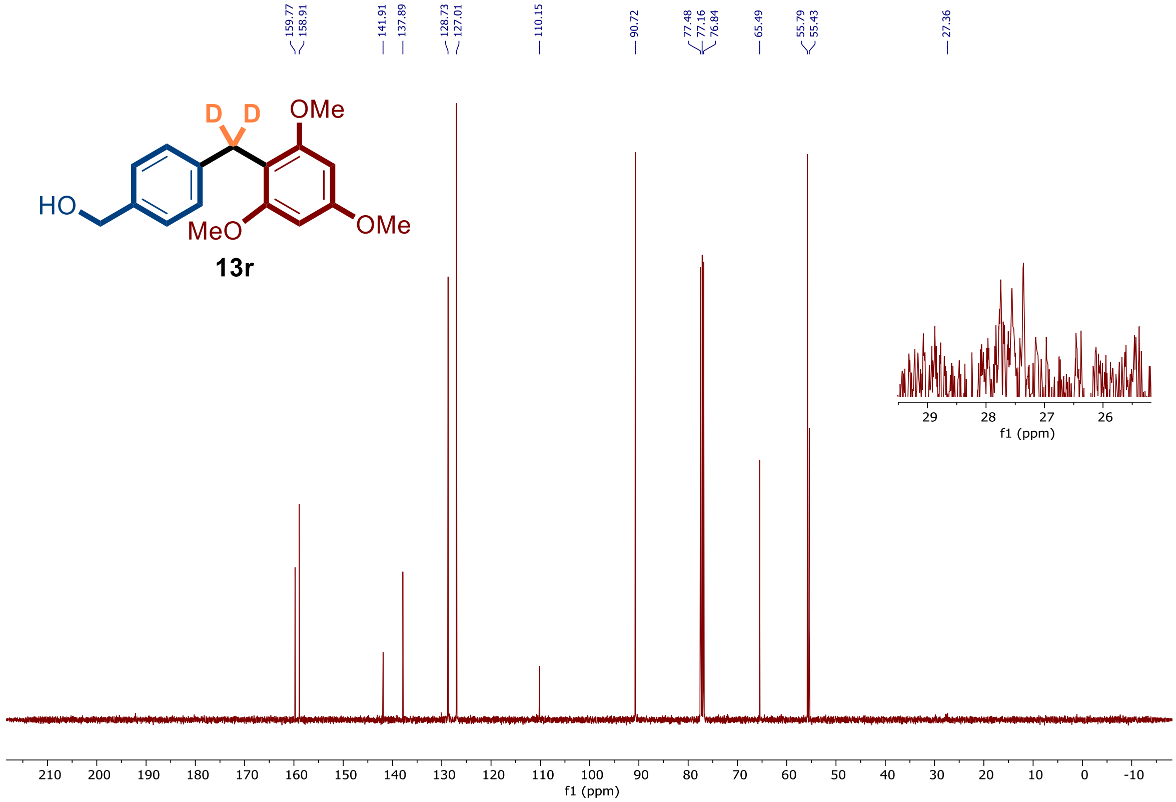

^{13}C NMR (101 MHz, $CDCl_3$) of compound (**13r**)

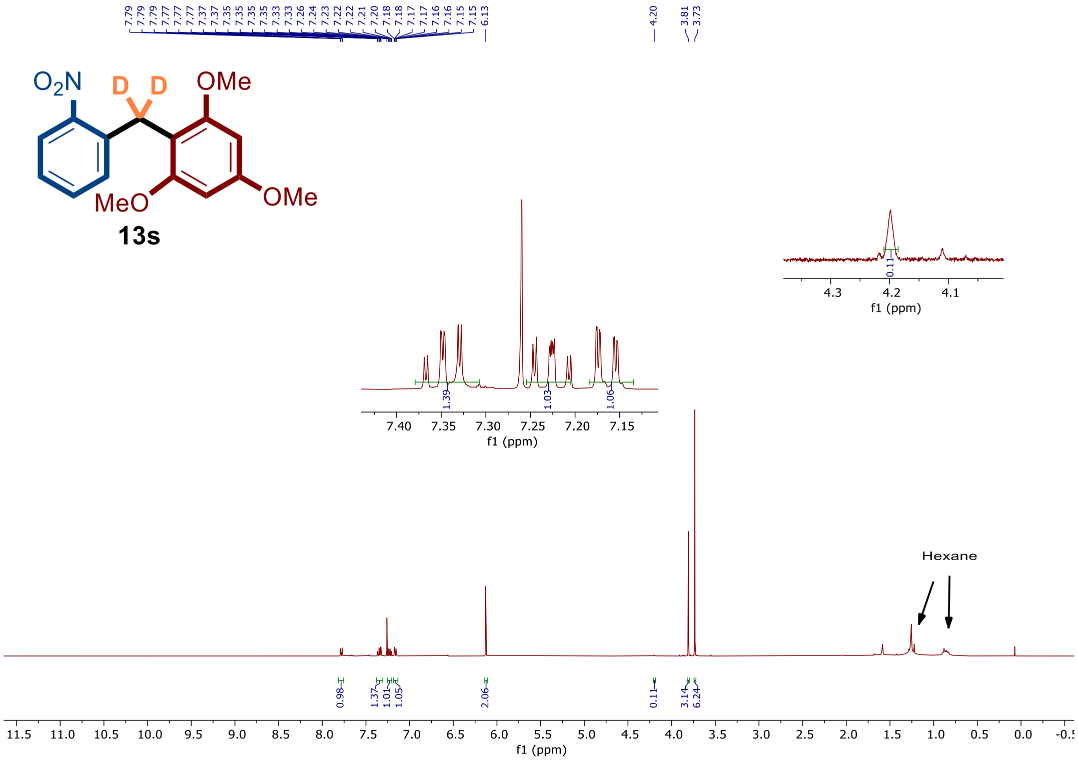

^{1}H NMR (400 MHz, $CDCl_3$) of compound (**13s**)

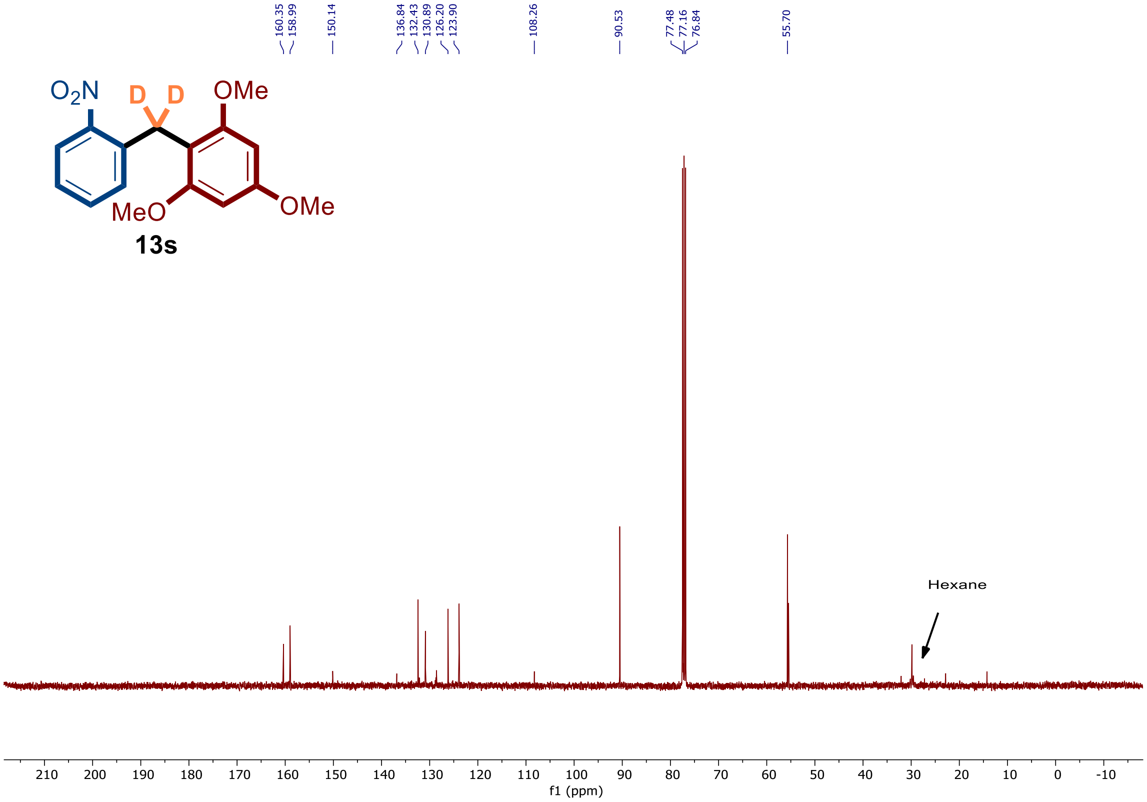

^{13}C NMR (101 MHz, $CDCl_3$) of compound (**13s**)

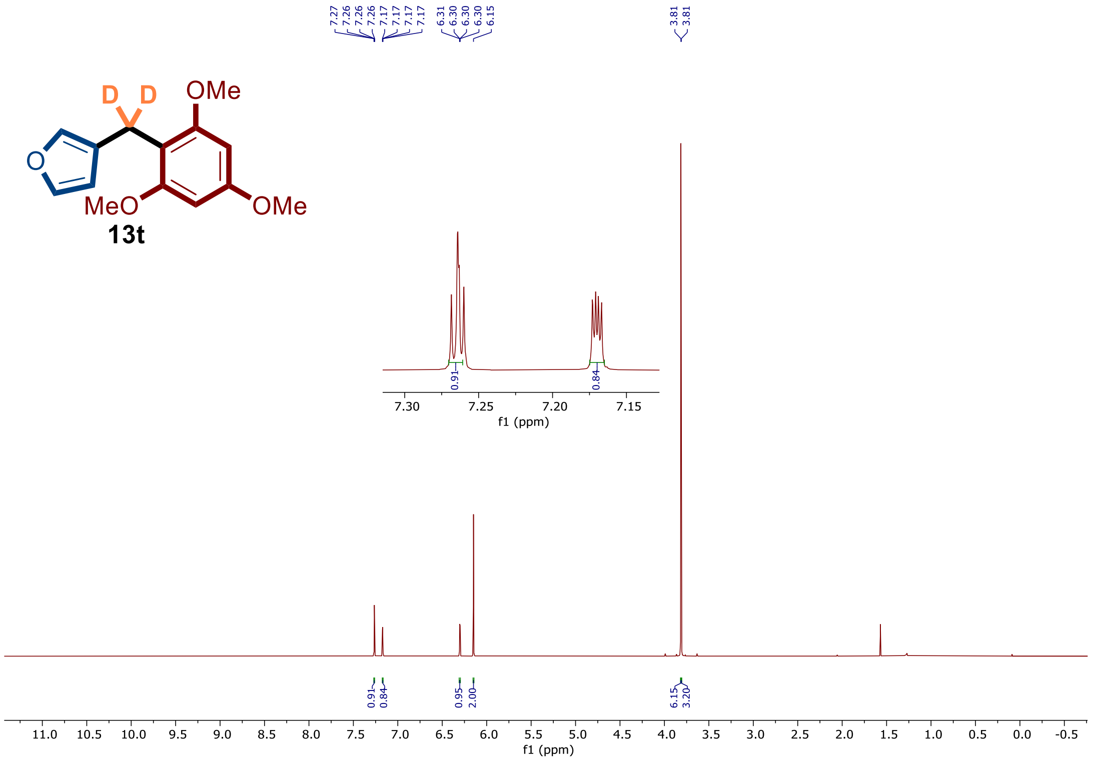

^{1}H NMR (400 MHz, CDCl$_3$) of compound (**13t**)

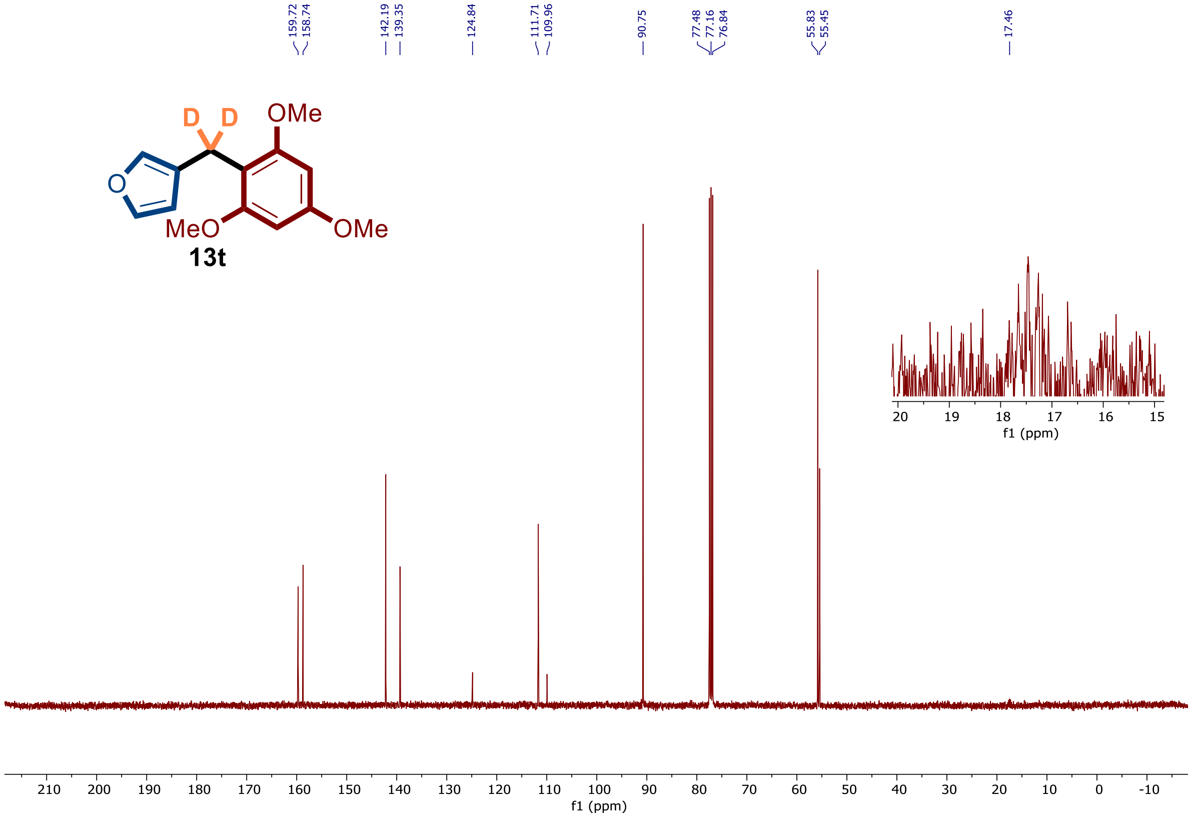

^{13}C NMR (101 MHz, CDCl$_3$) of compound (**13t**)

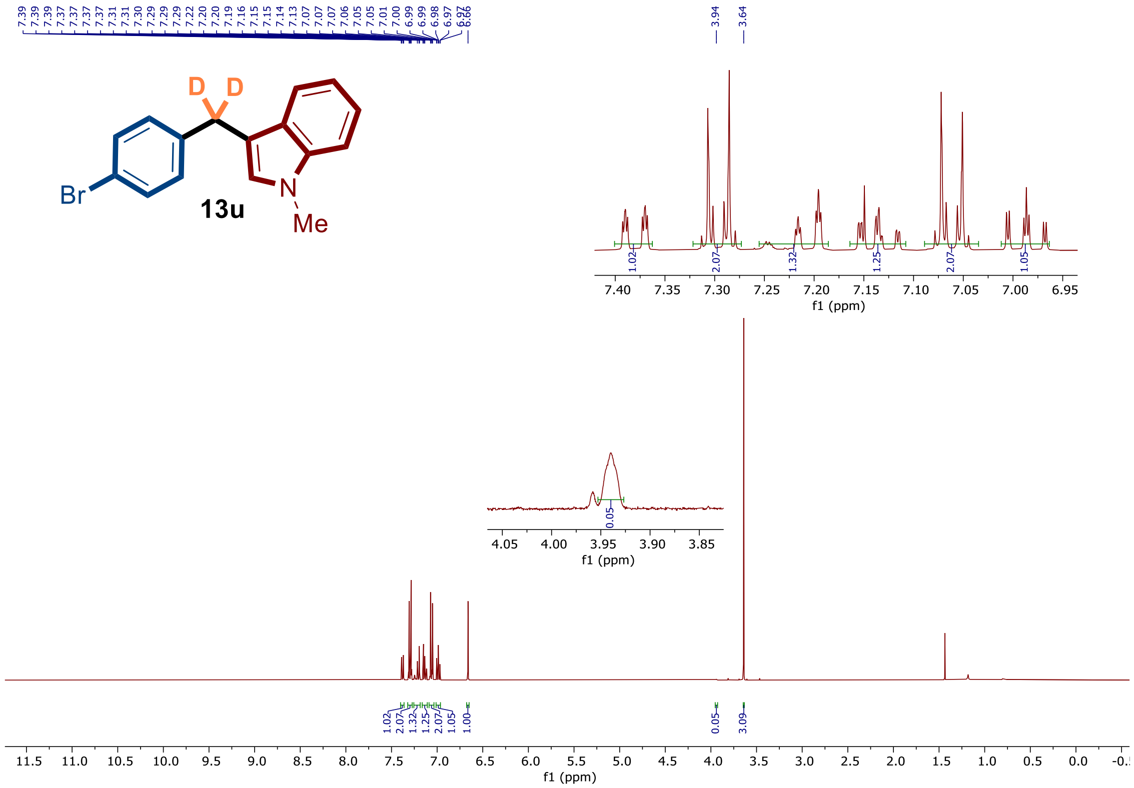

^{1}H NMR (400 MHz, $CDCl_3$) of compound (**13u**)

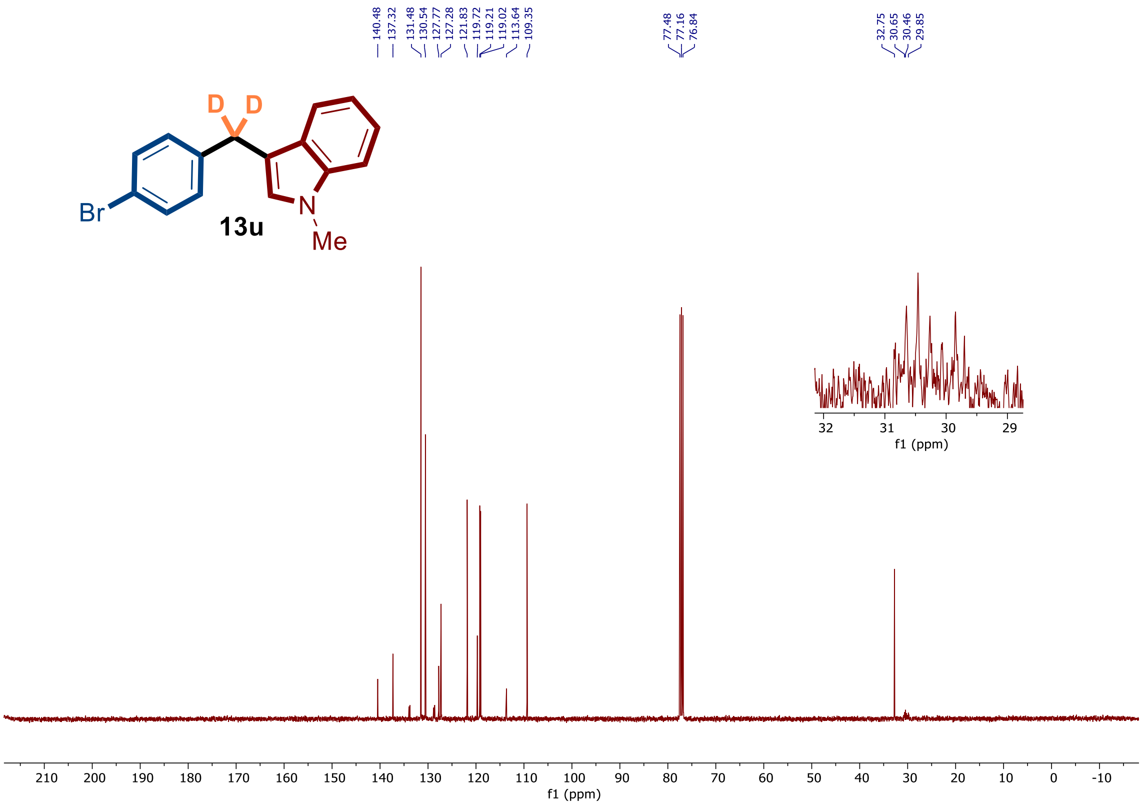

^{13}C NMR (101 MHz, $CDCl_3$) of compound (**13u**)

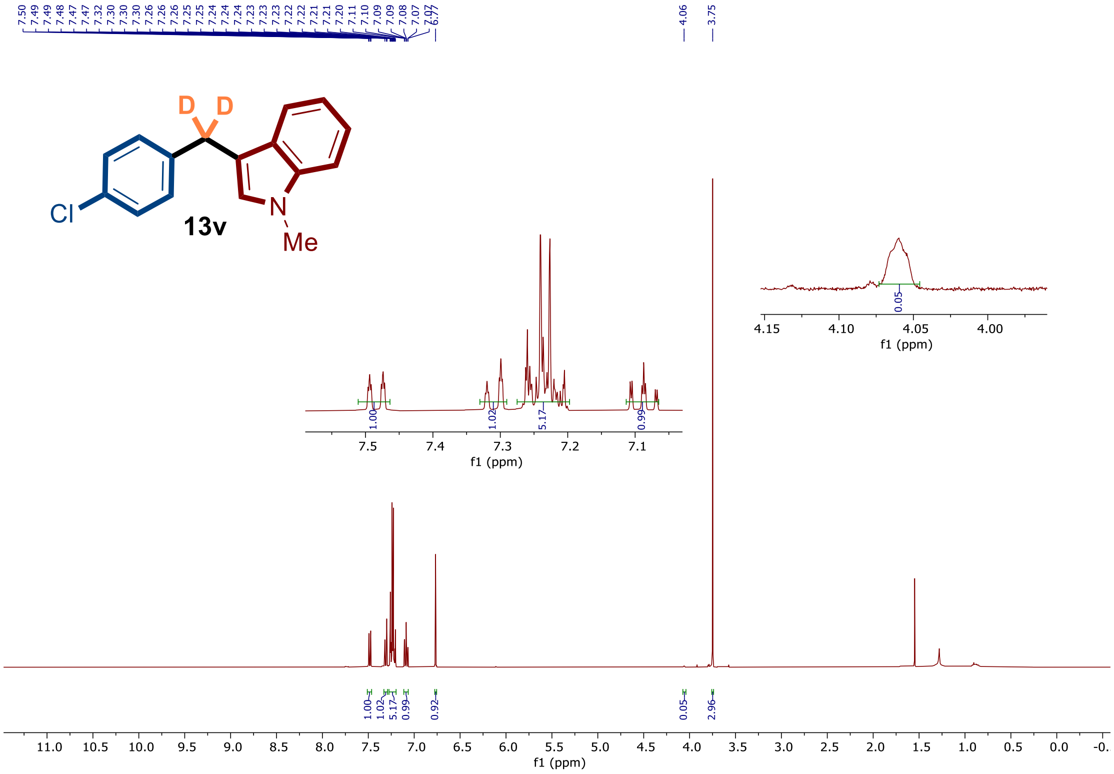

^{1}H NMR (400 MHz, $CDCl_3$) of compound (**13v**)

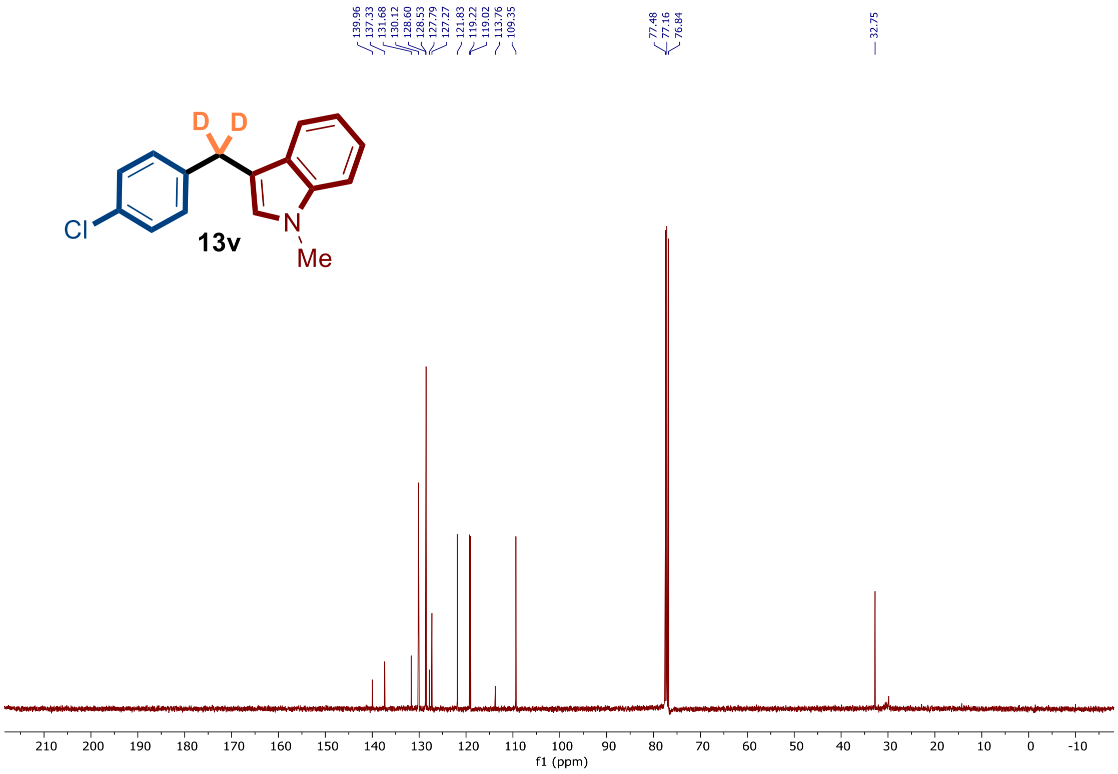

^{13}C NMR (101 MHz, $CDCl_3$) of compound (**13v**)

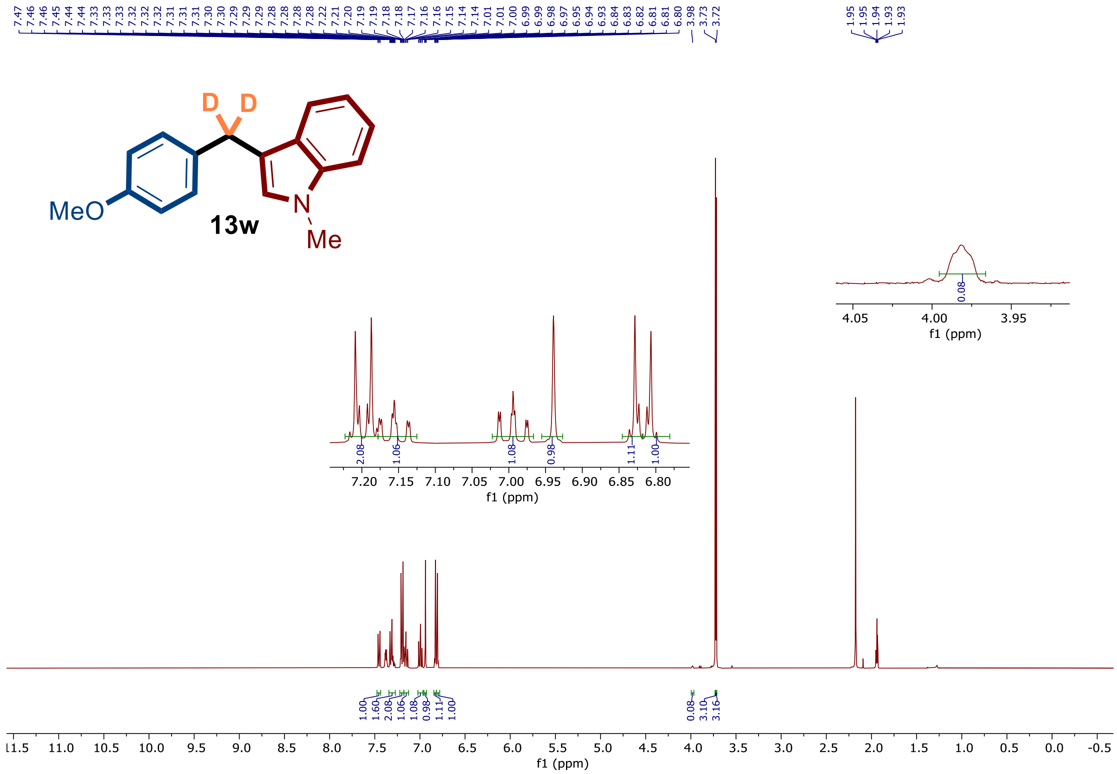

^{1}H NMR (400 MHz, CD_3CN) of compound (**13w**)

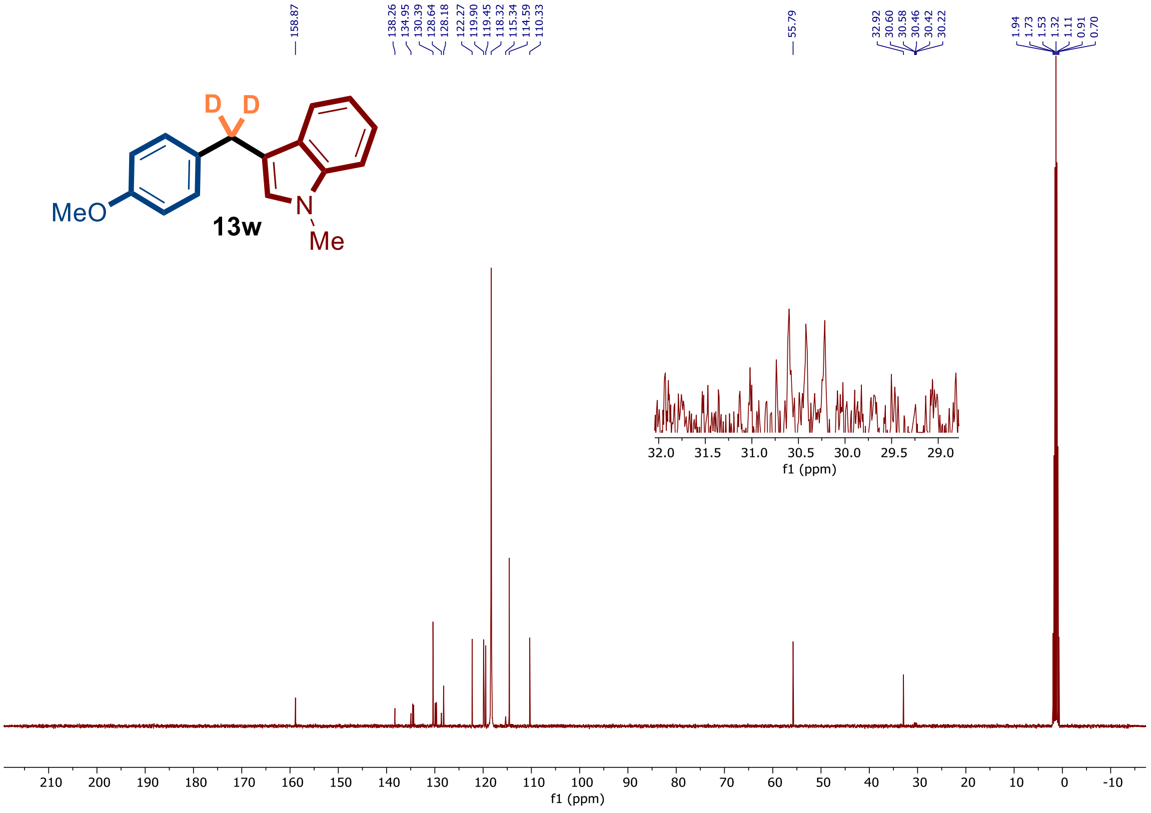

^{13}C NMR (101 MHz, CD_3CN) of compound (**13w**)

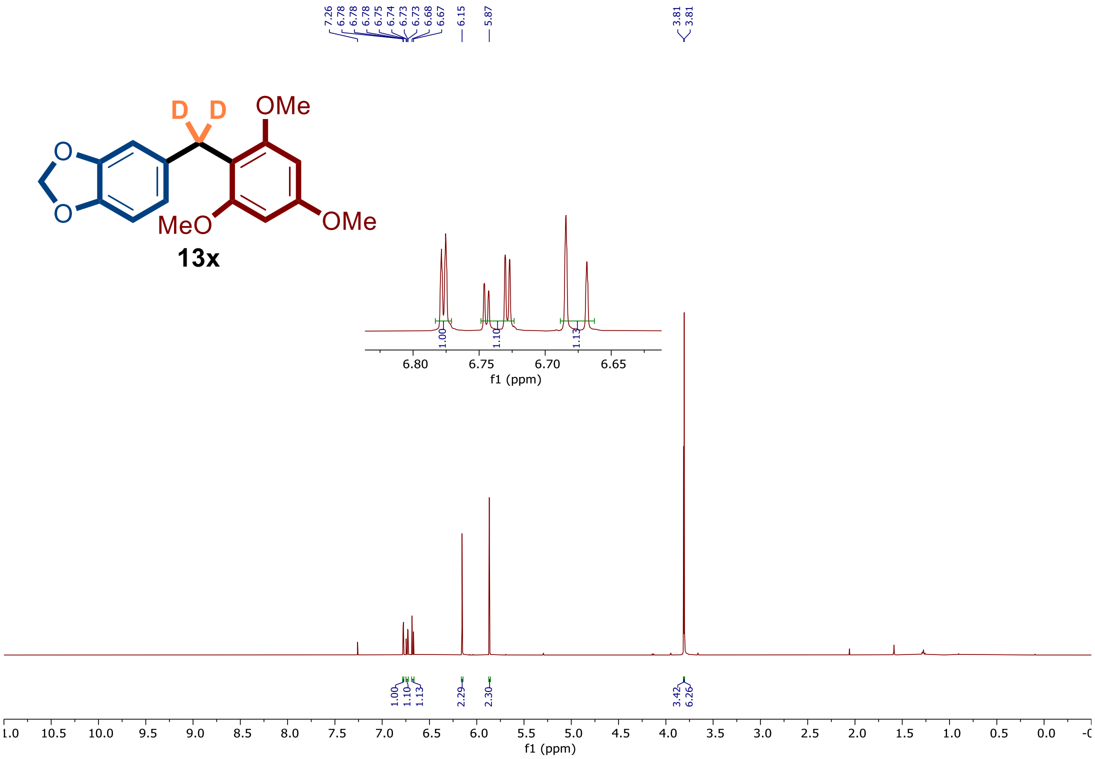

^{1}H NMR (400 MHz, $CDCl_3$) of compound (**13x**)

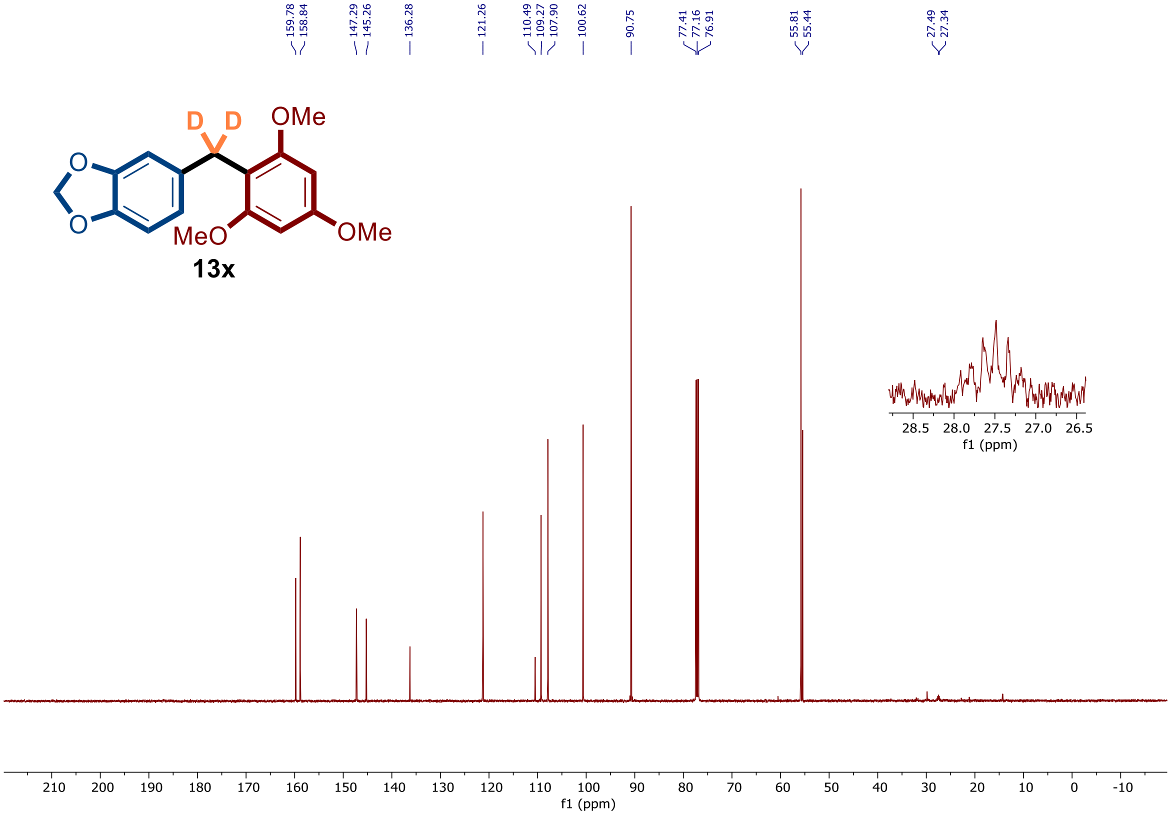

^{13}C NMR (101 MHz, $CDCl_3$) of compound (**13x**)

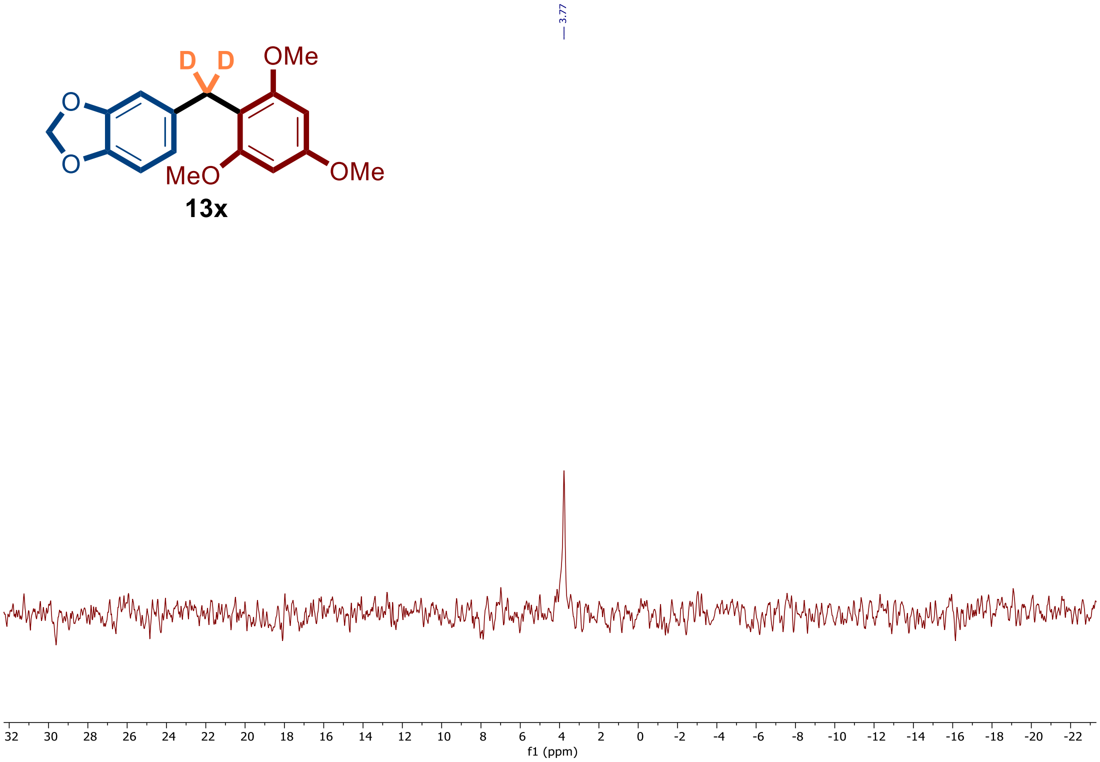

^{2}H NMR (77 MHz, $CDCl_3$) of compound (**13x**)

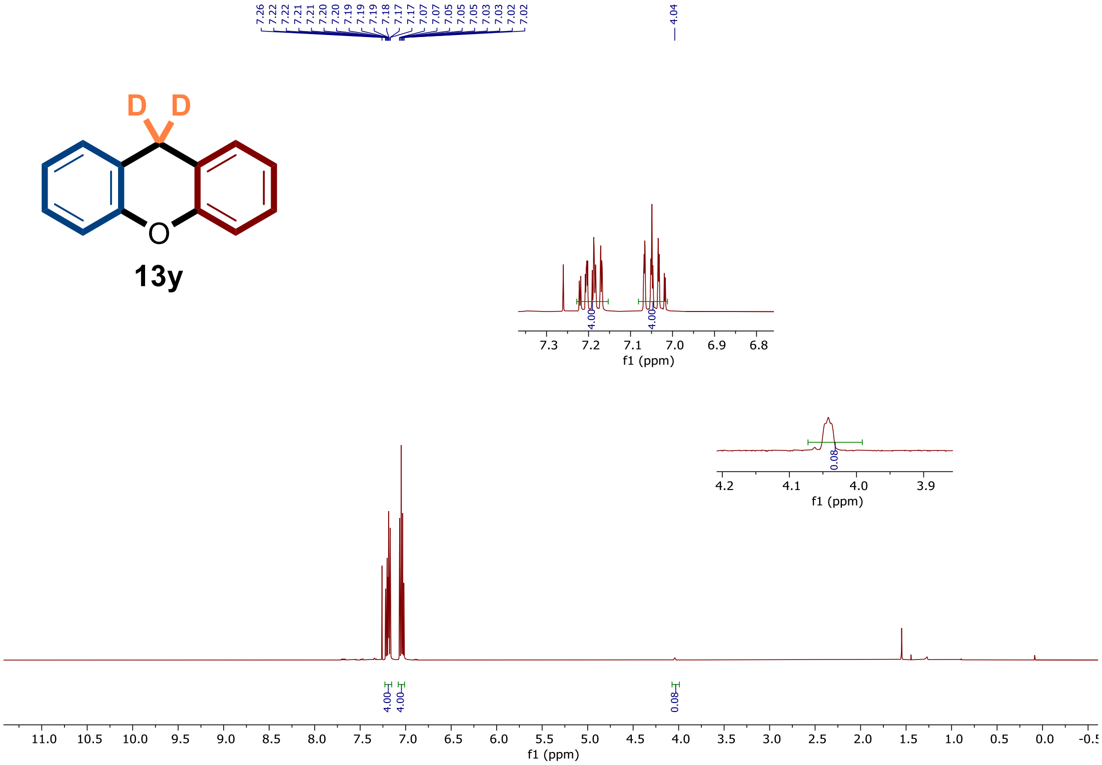

^{1}H NMR (400 MHz, $CDCl_3$) of compound (**13y**)

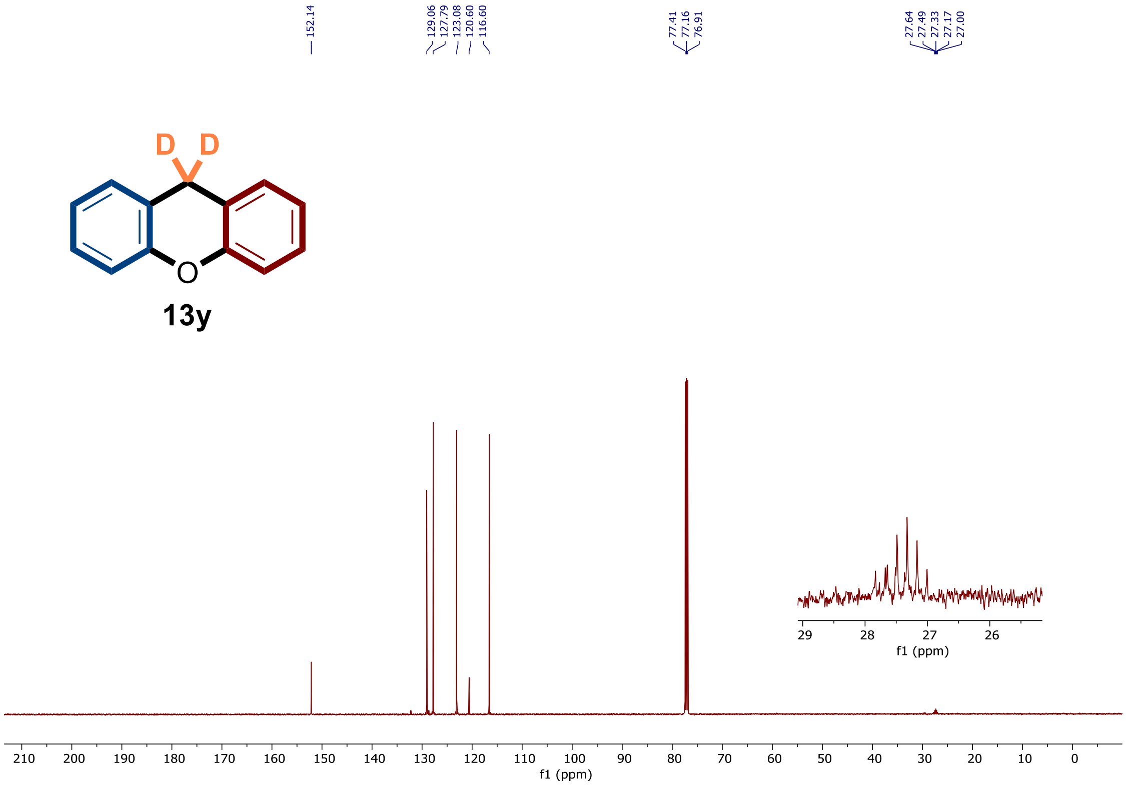

^{13}C NMR (101 MHz, $CDCl_3$) of compound (**13y**)

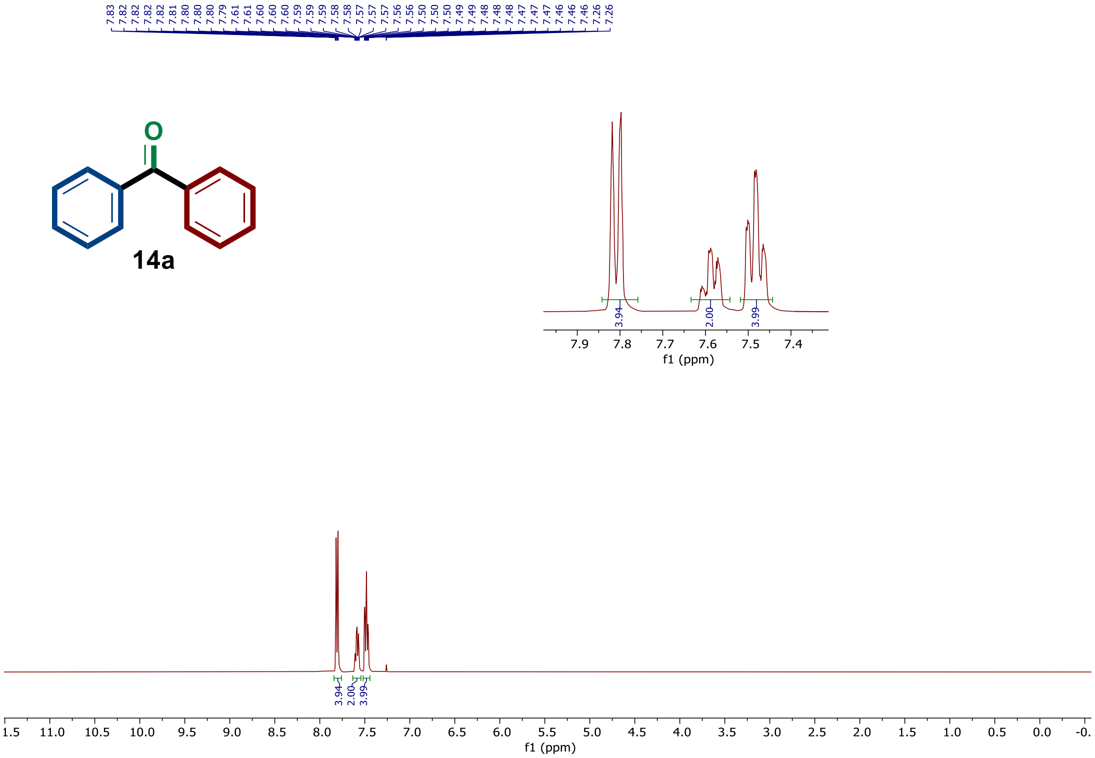

^{1}H NMR (400 MHz, $CDCl_3$) of compound (**14a**)

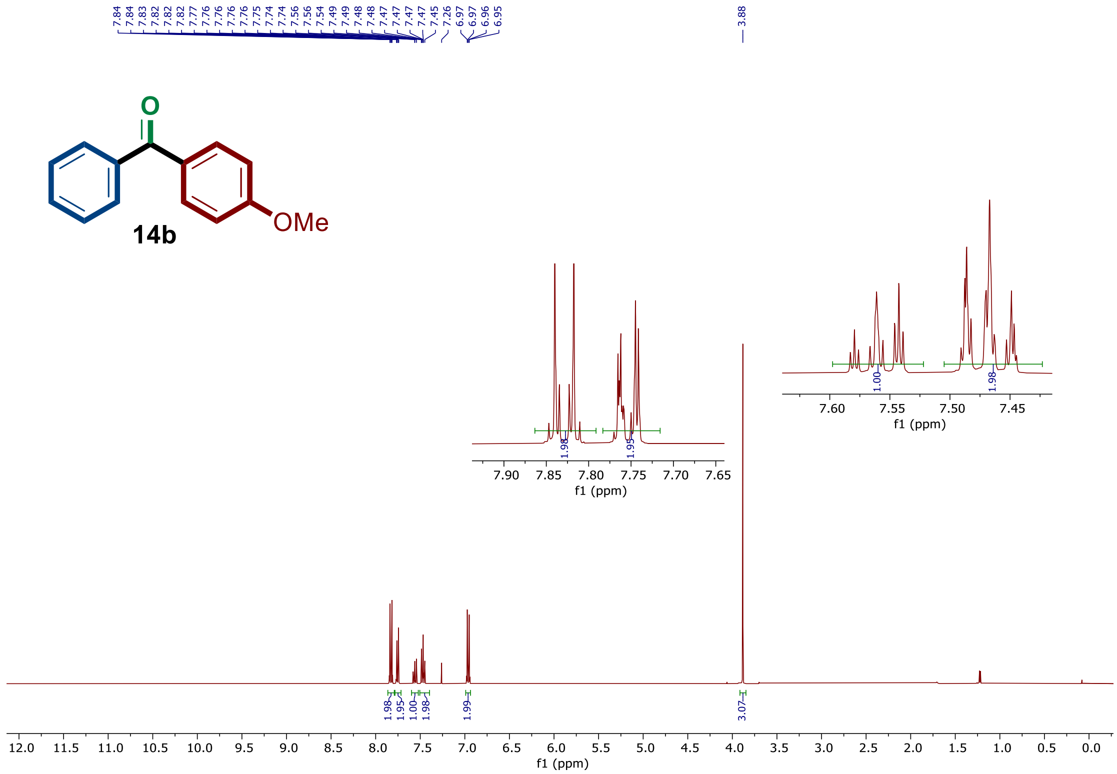

^{1}H NMR (400 MHz, $CDCl_3$) of compound (**14b**)

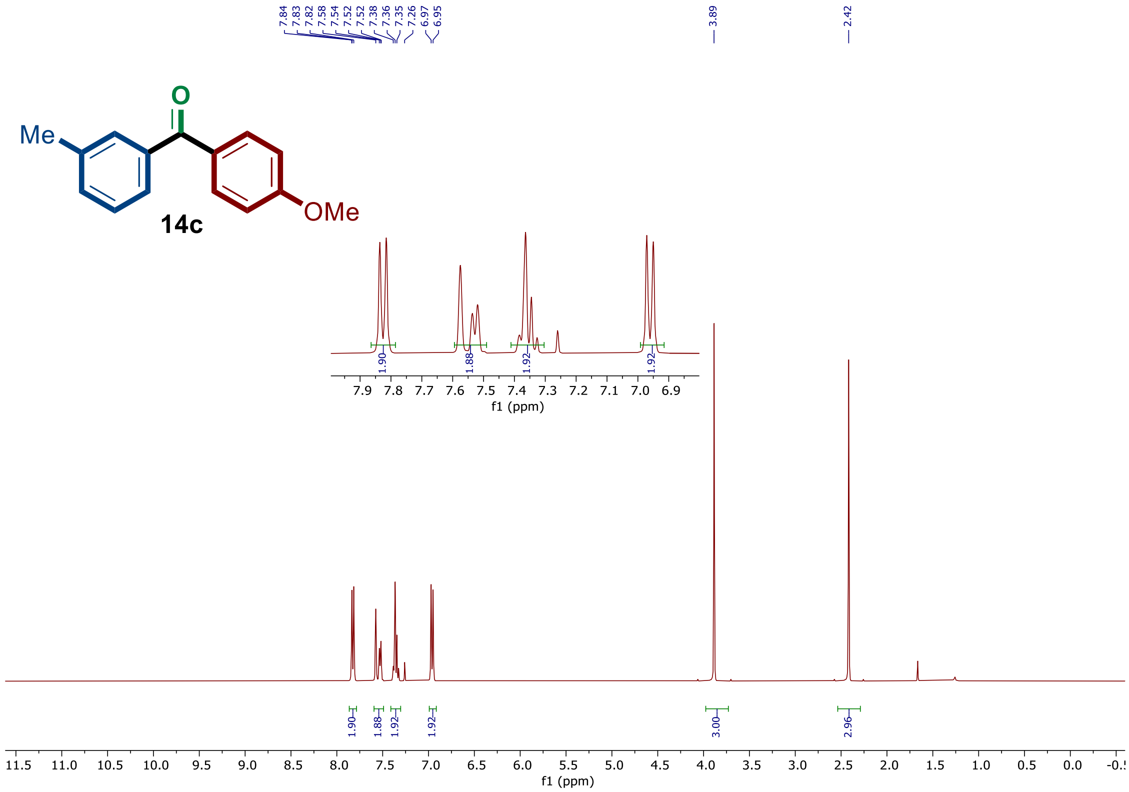

^{1}H NMR (400 MHz, $CDCl_3$) of compound (**14c**)

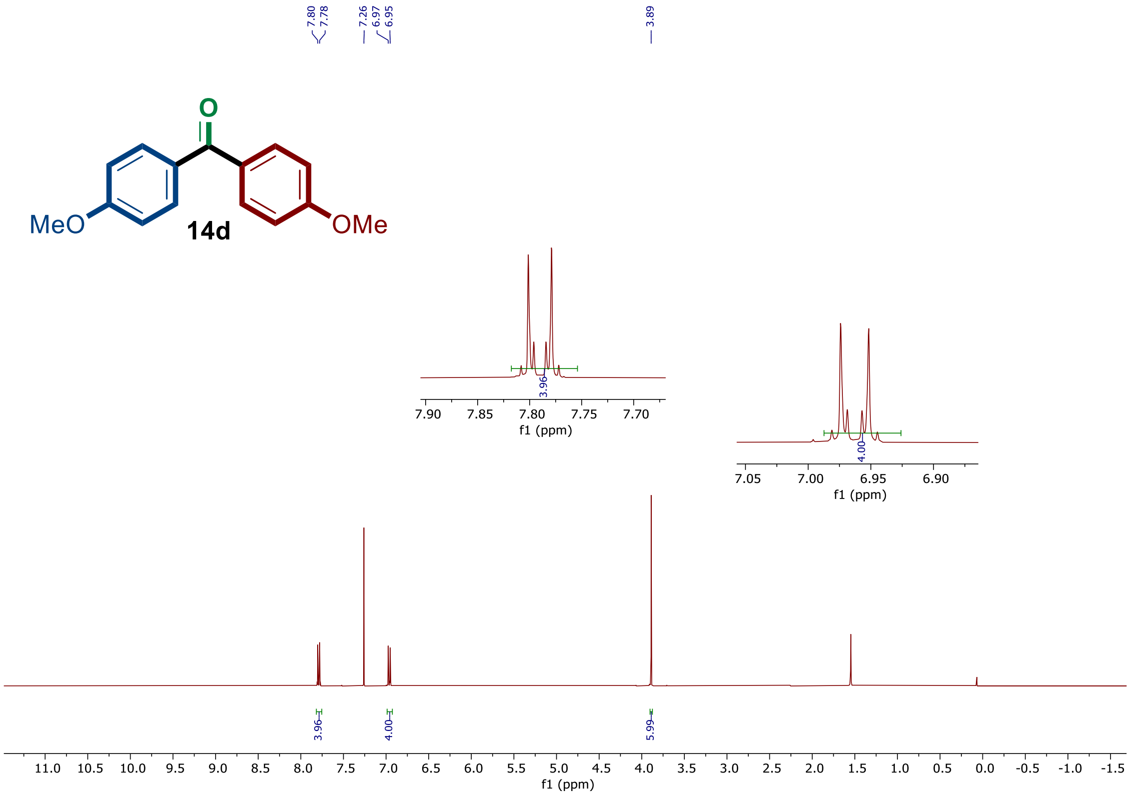

^{1}H NMR (400 MHz, $CDCl_3$) of compound (**14d**)

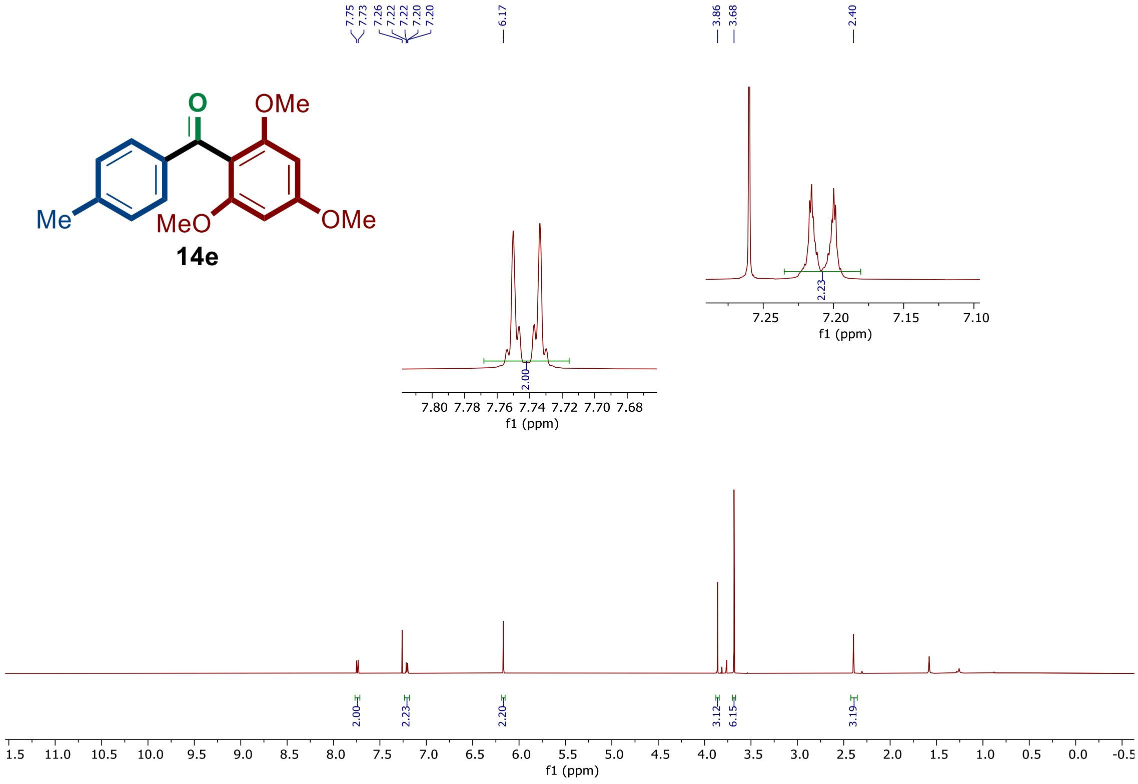

^{1}H NMR (400 MHz, $CDCl_3$) of compound (**14e**)

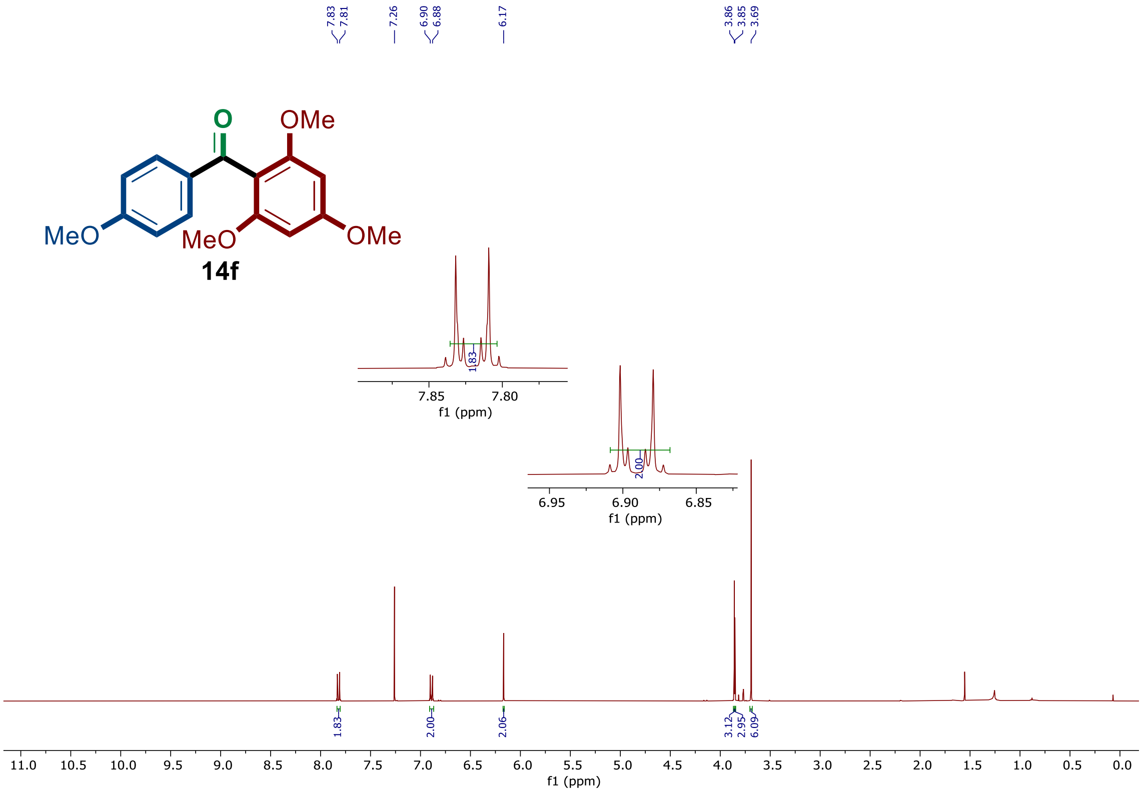

^1H NMR (400 MHz, $CDCl_3$) of compound (**14f**)

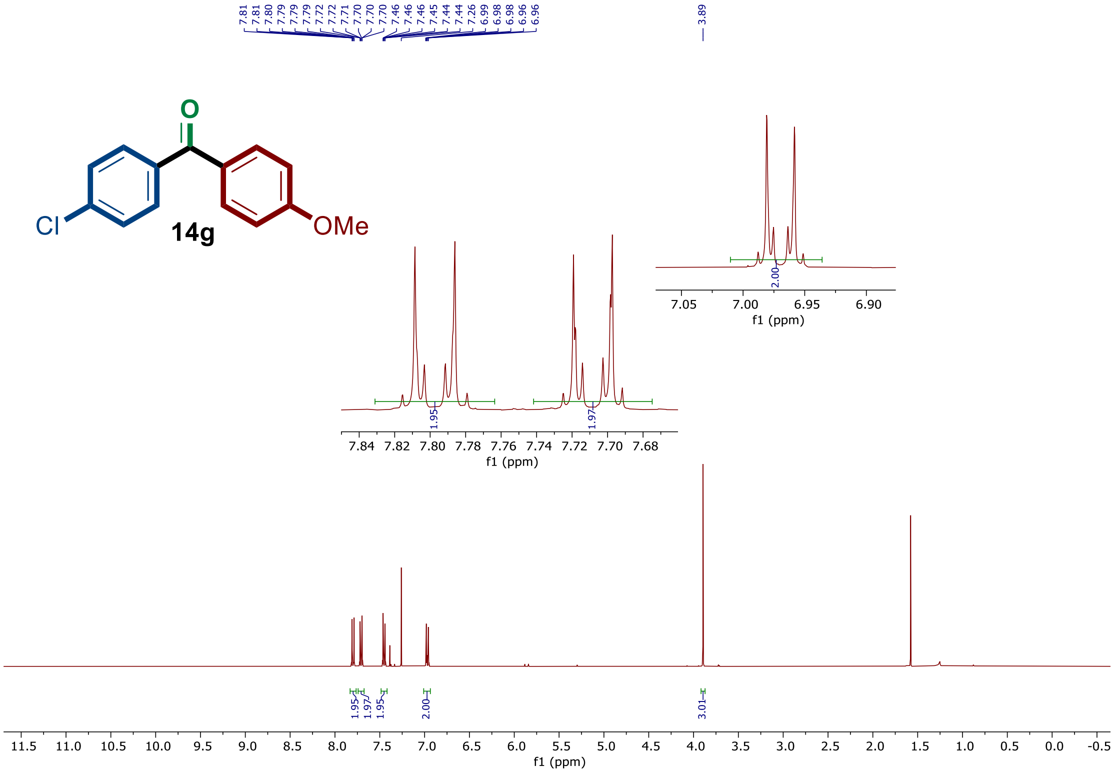

^1H NMR (400 MHz, $CDCl_3$) of compound (**14g**)

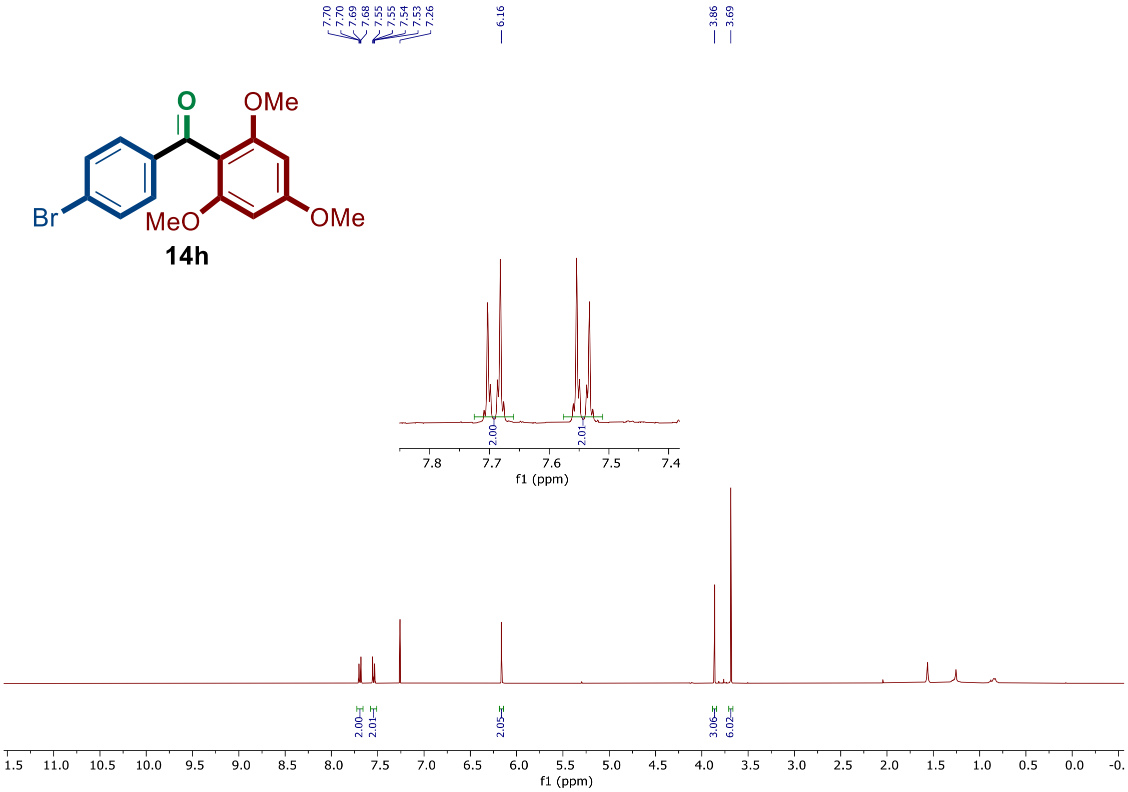

^{1}H NMR (400 MHz, $CDCl_3$) of compound (**14h**)

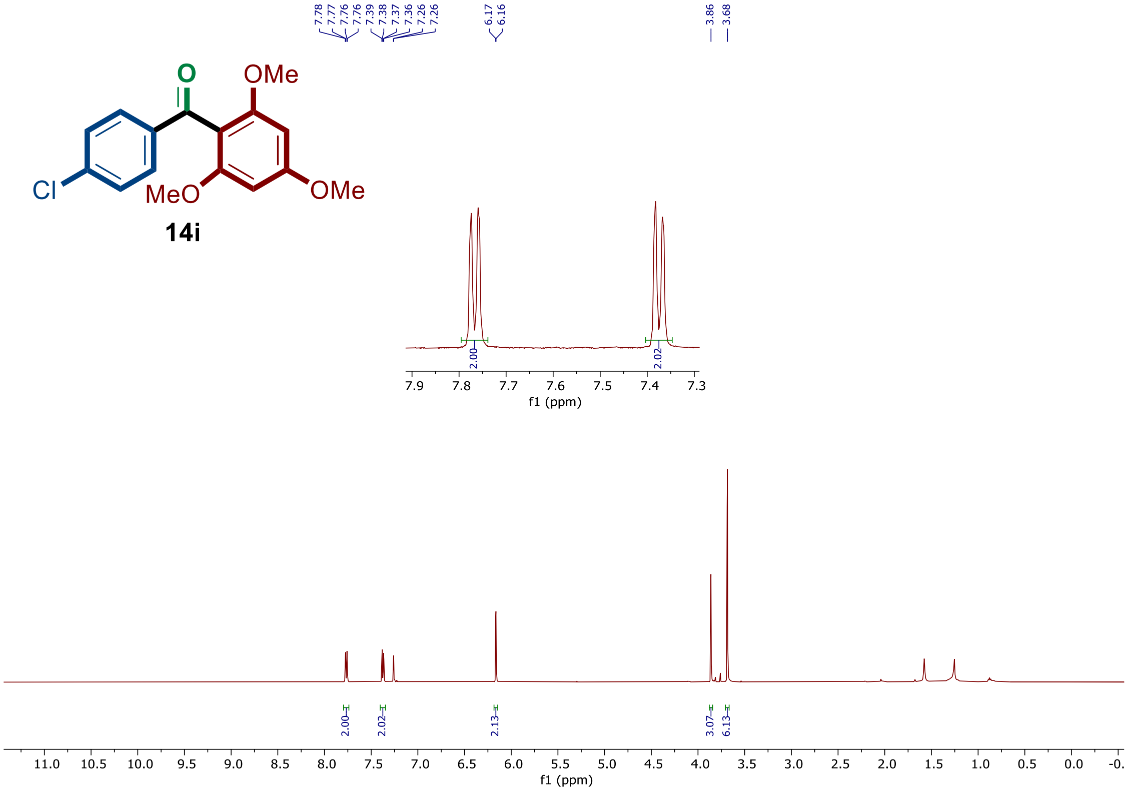

^{1}H NMR (400 MHz, $CDCl_3$) of compound (**14i**)

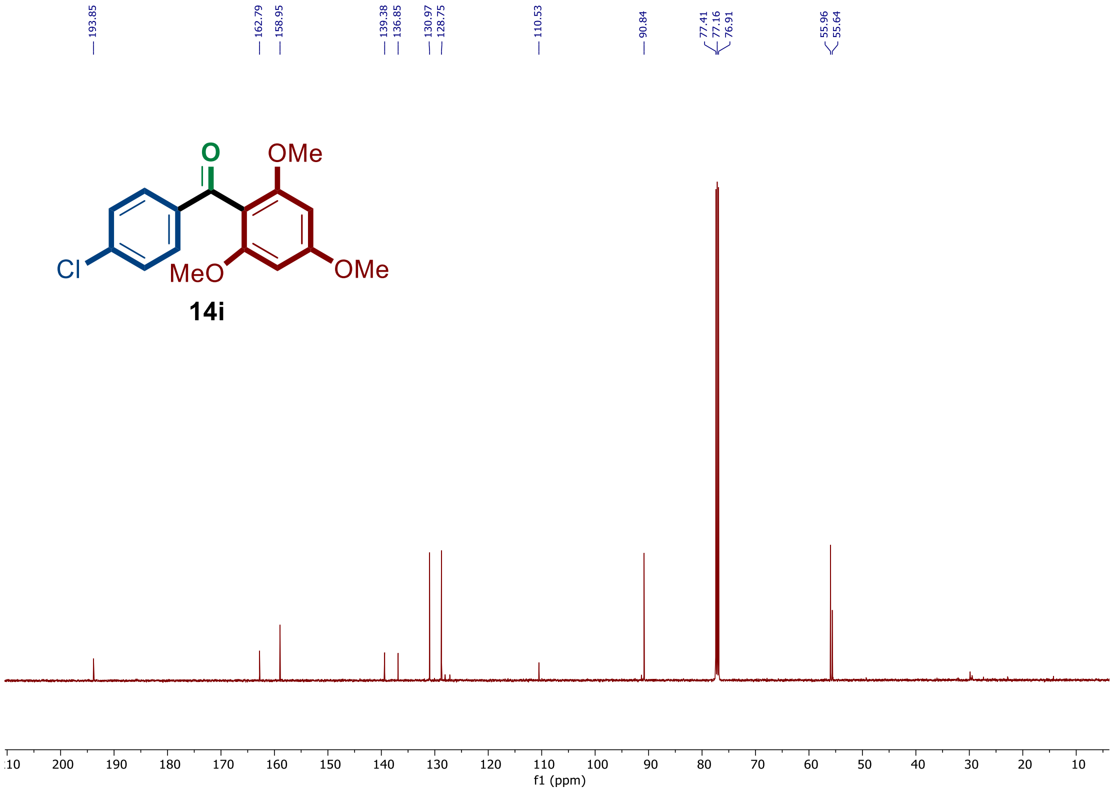

^{13}C NMR (101 MHz, $CDCl_3$) of compound (**14i**)

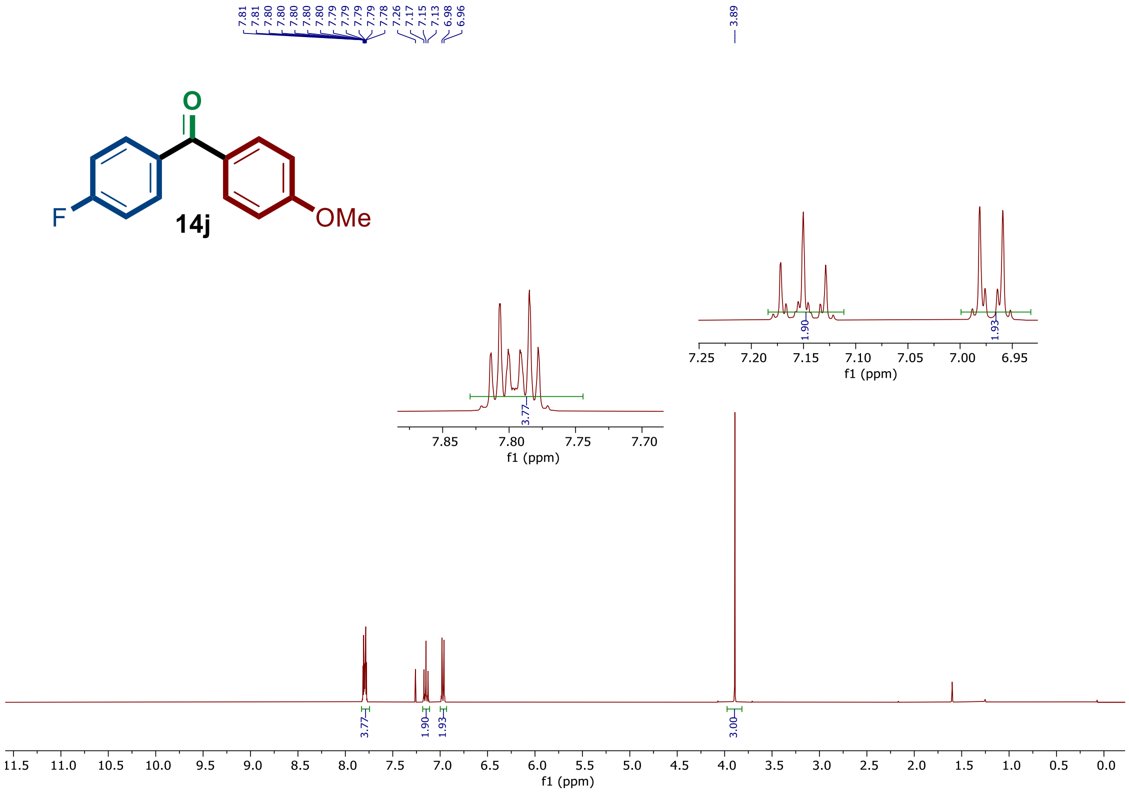

^{1}H NMR (400 MHz, $CDCl_3$) of compound (**14j**)

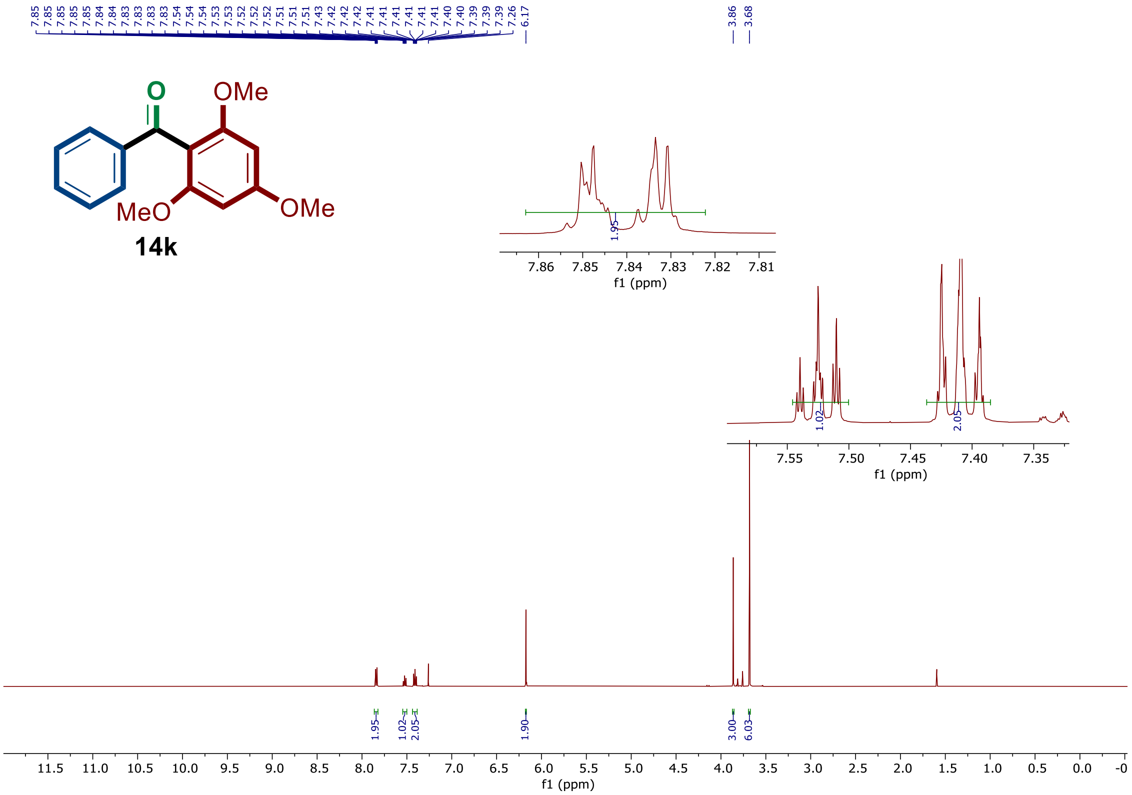

^{1}H NMR (400 MHz, CDCl$_3$) of compound (**14k**)

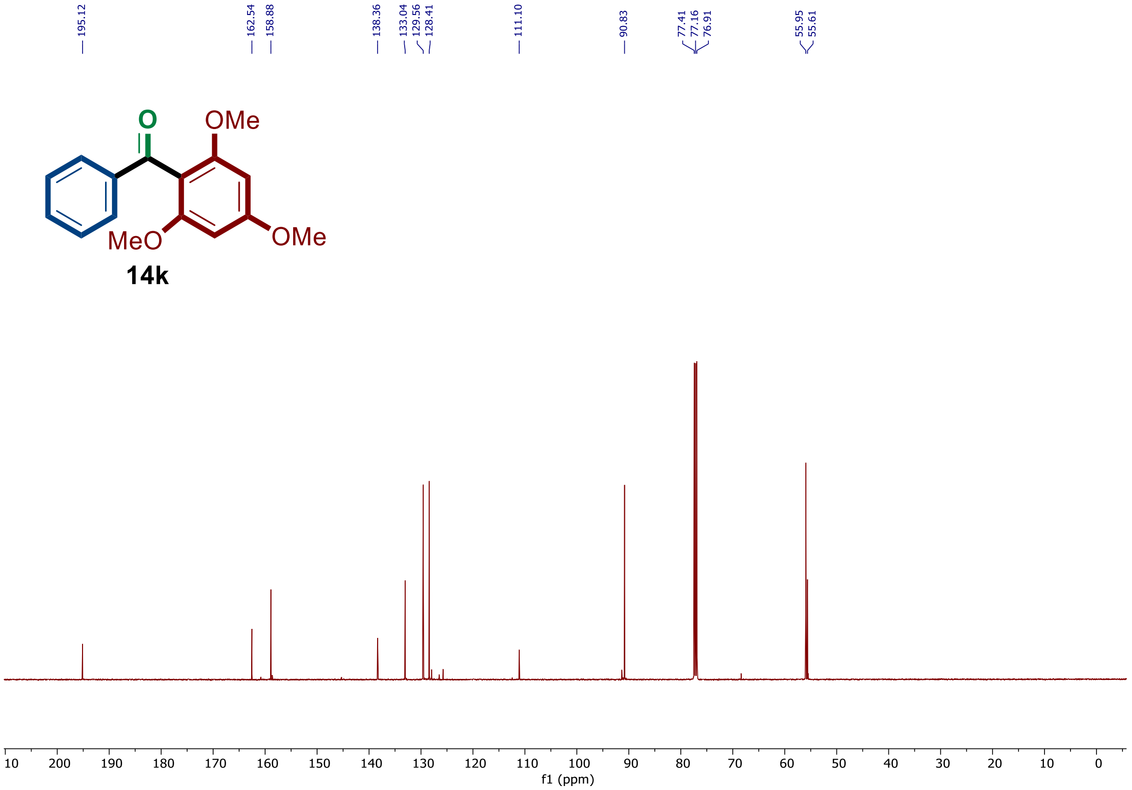

^{13}C NMR (101 MHz, CDCl$_3$) of compound (**14k**)

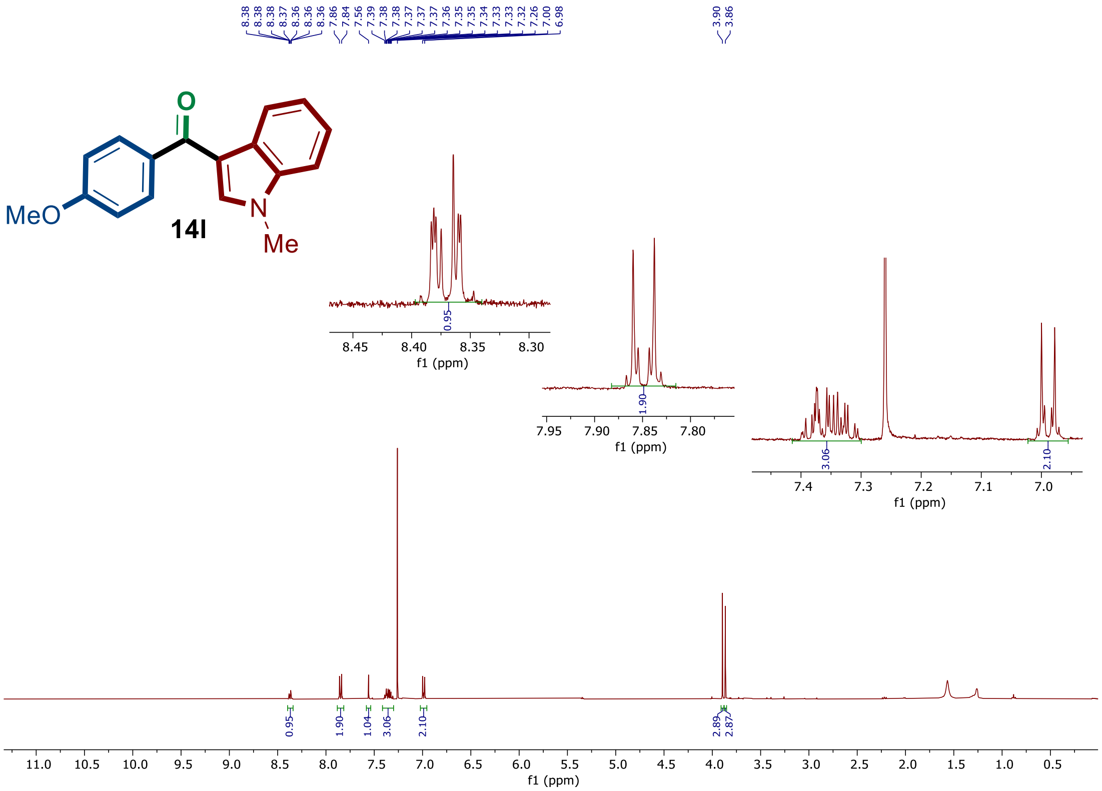

^{1}H NMR (400 MHz, $CDCl_3$) of compound (**14l**)

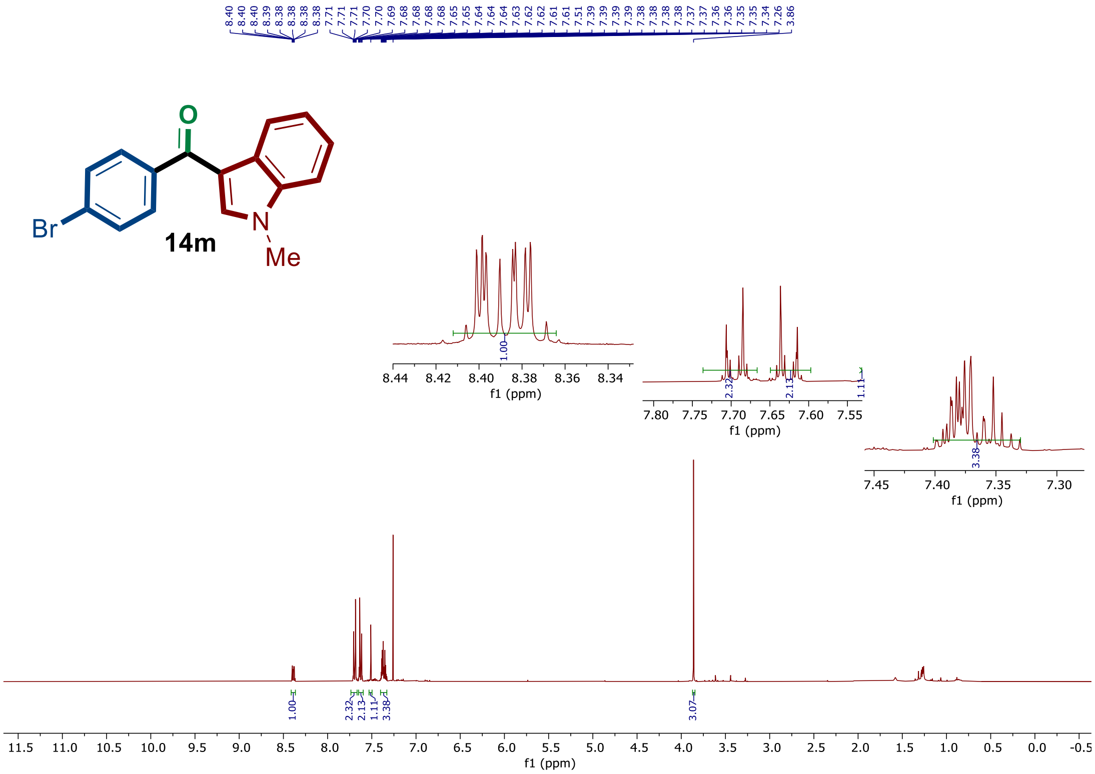

^{1}H NMR (400 MHz, $CDCl_3$) of compound (**14m**)

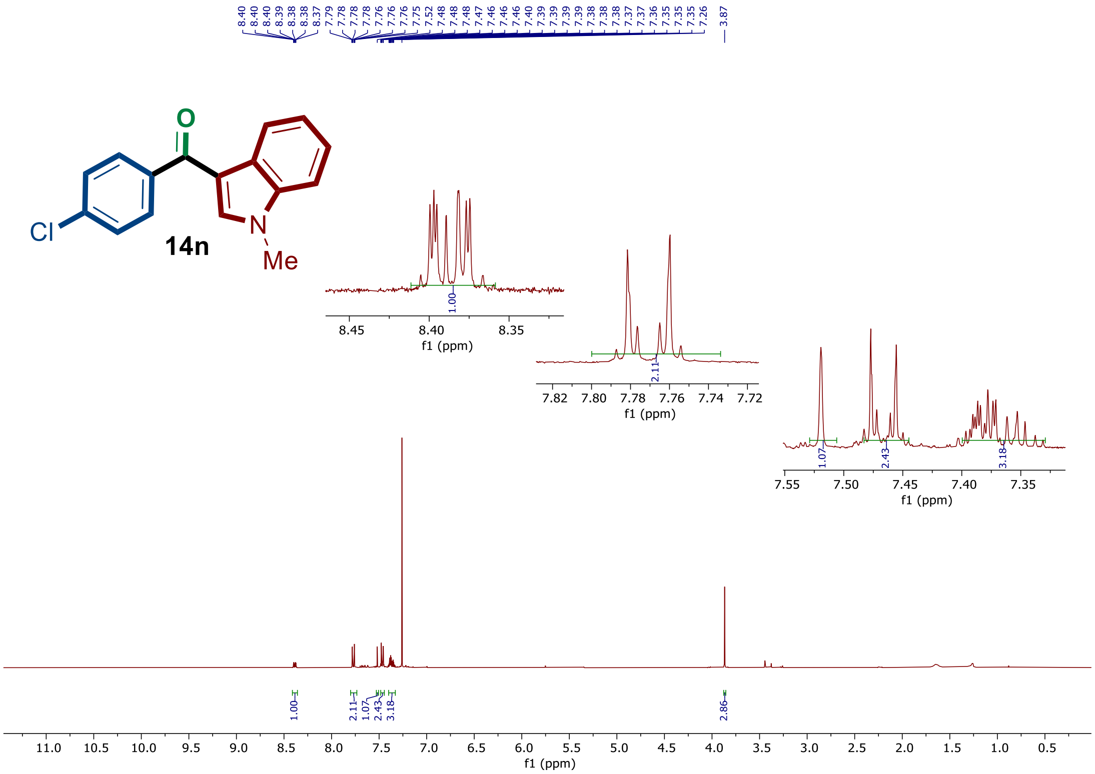

^{1}H NMR (400 MHz, $CDCl_3$) of compound (**14n**)

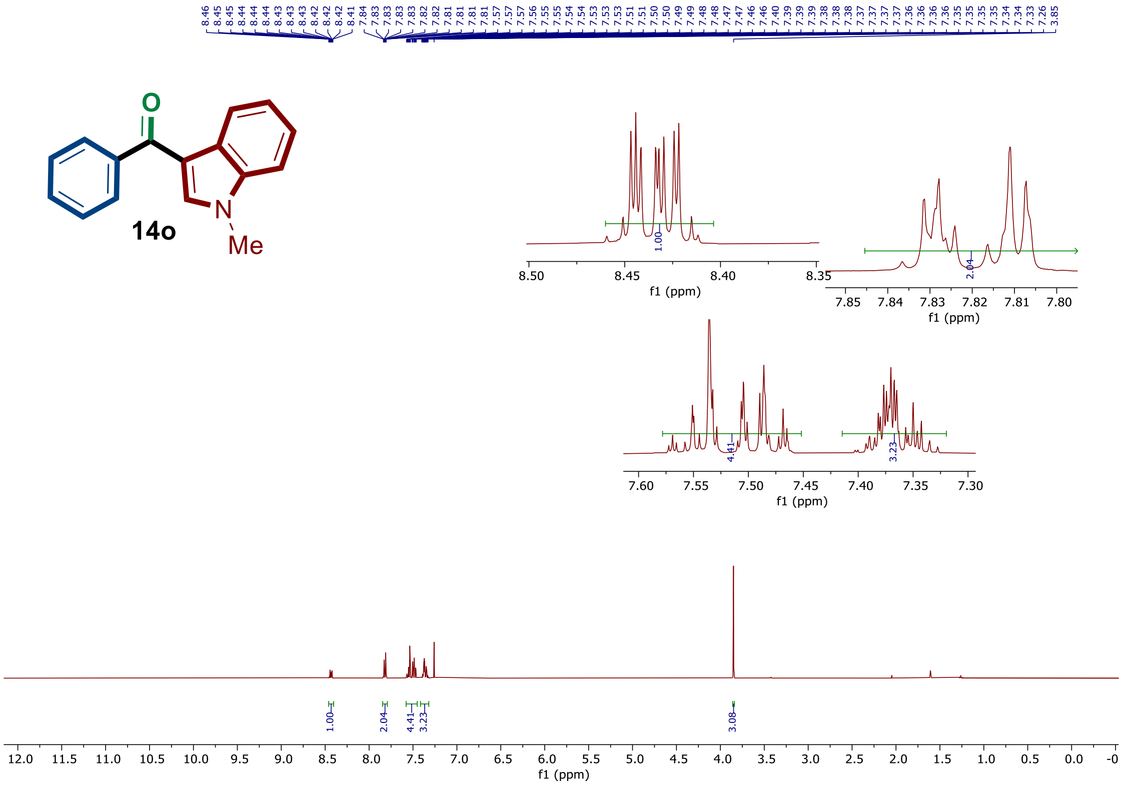

^{1}H NMR (400 MHz, $CDCl_3$) of compound (**14o**)

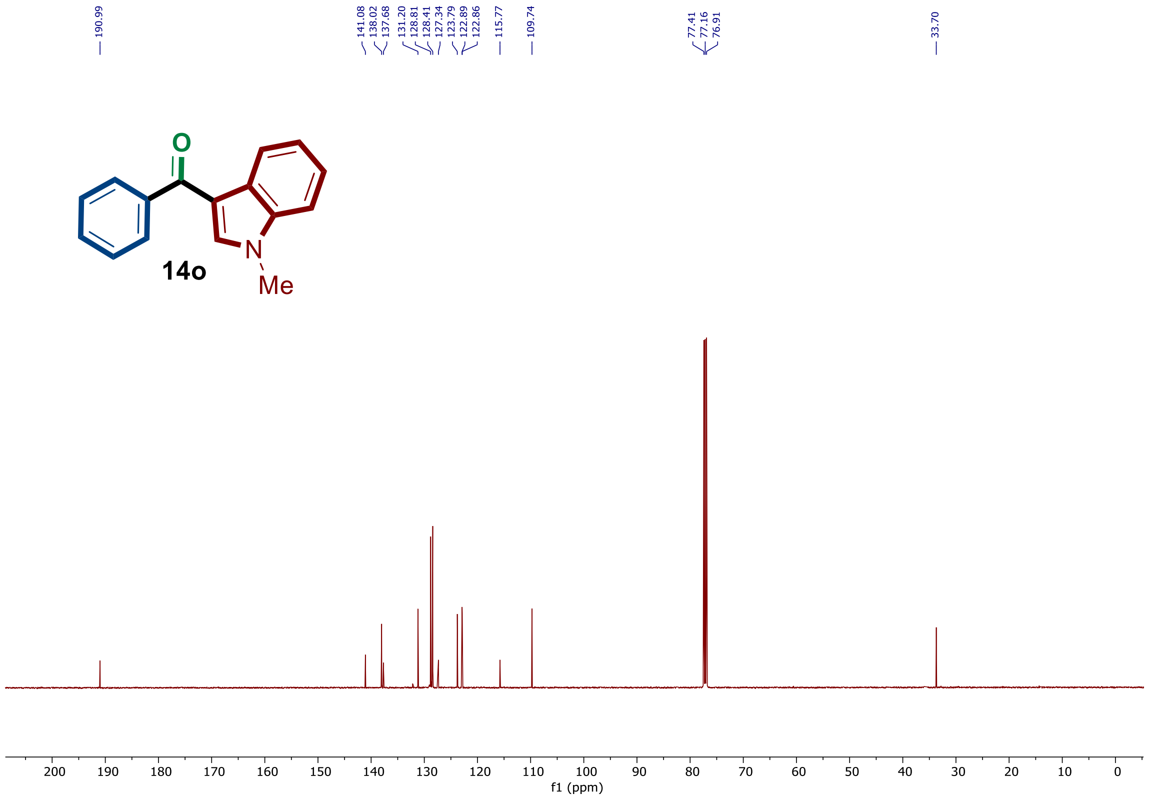

^{13}C NMR (101 MHz, $CDCl_3$) of compound (**14o**)

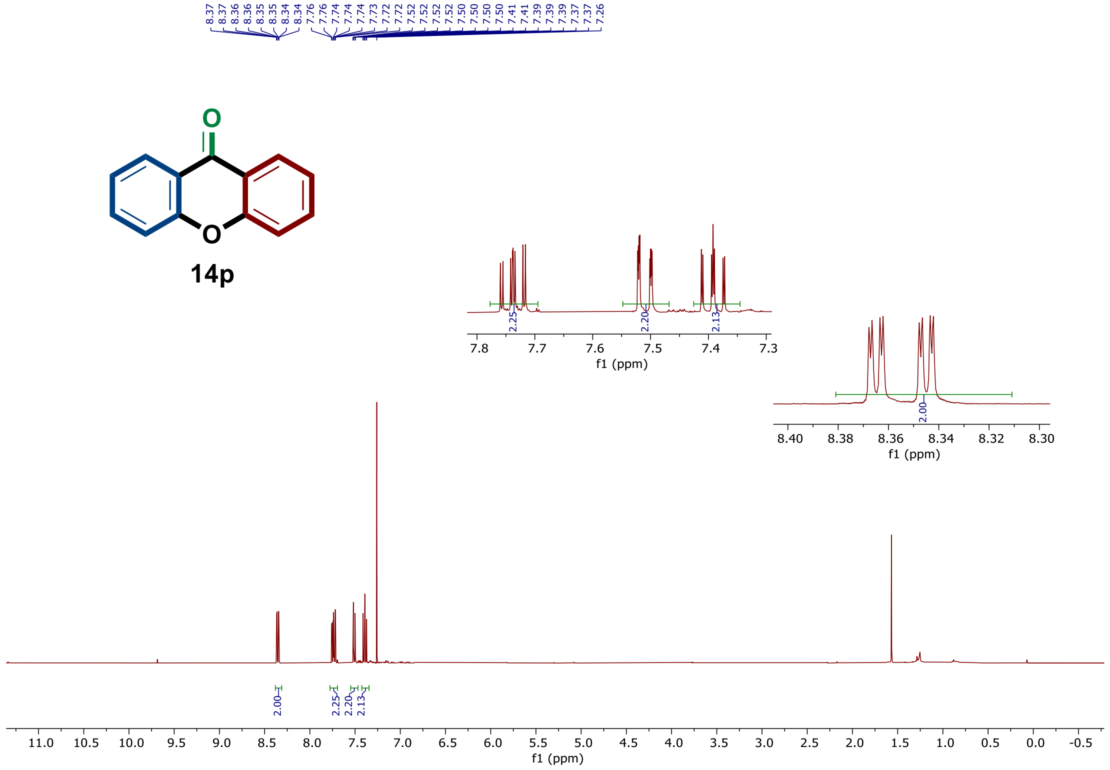

^{1}H NMR (400 MHz, $CDCl_3$) of compound (**14p**)

3.1. Reaction progress of site-selective oxidation of benzhydryl phosphonium salt (11z):

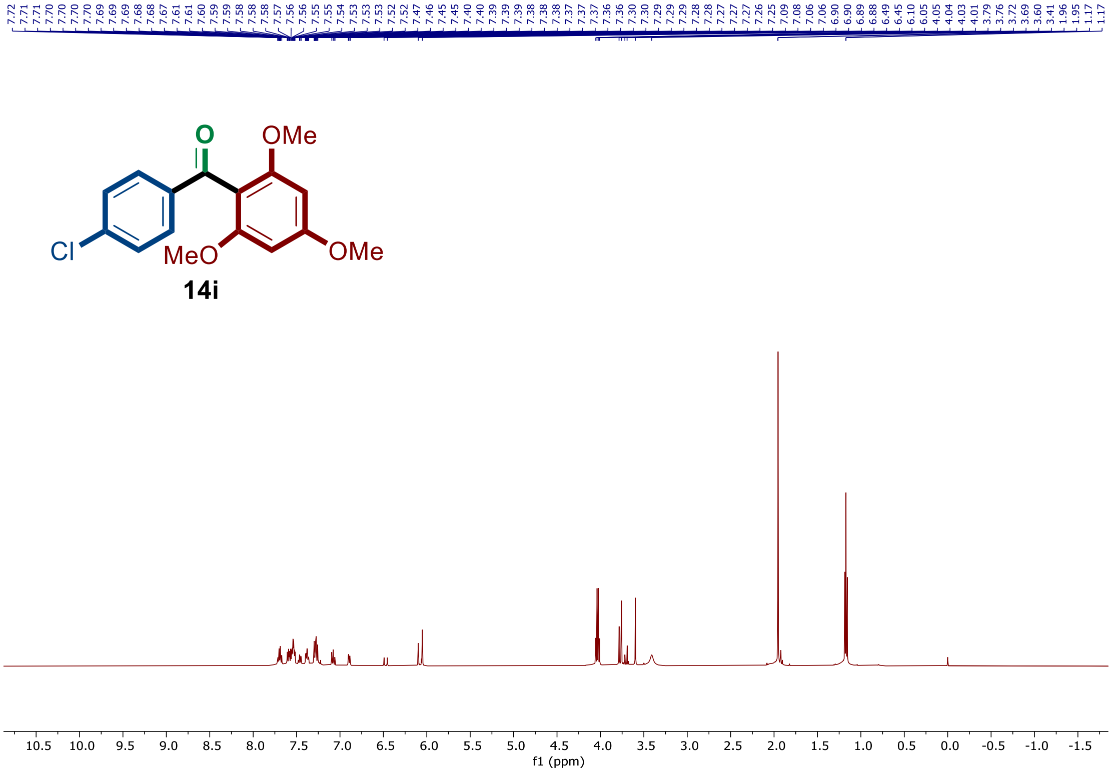

^{1}H NMR (400 MHz, $CDCl_3$) of the crude reaction mixture of (**14i**)

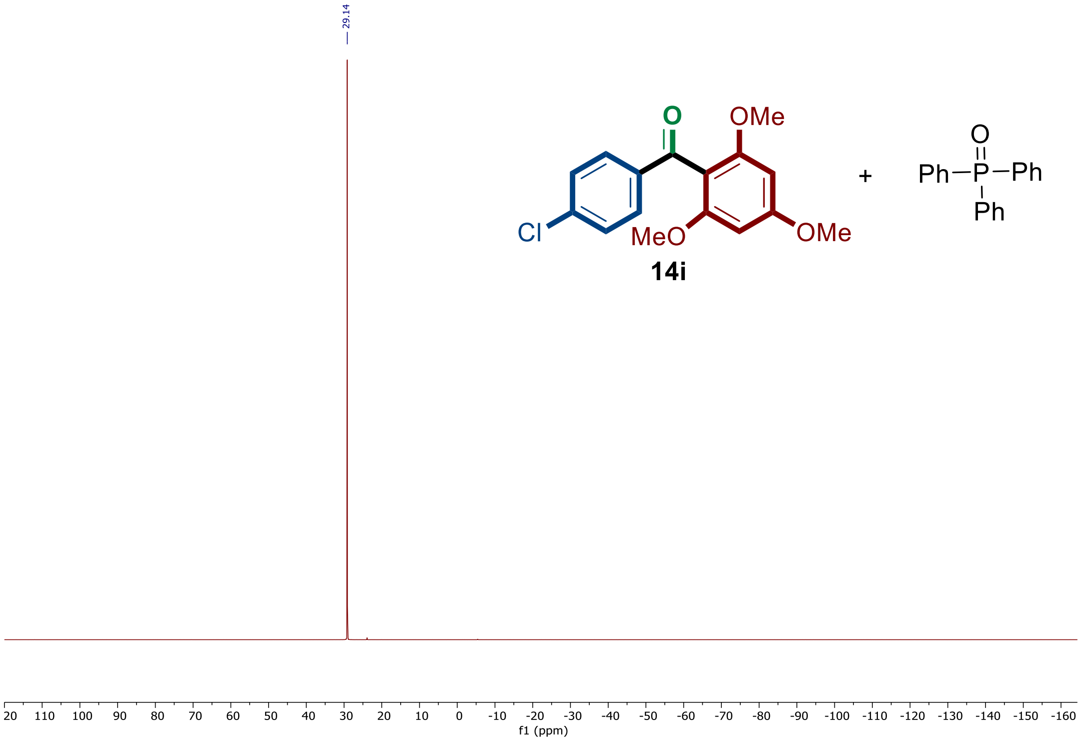

^{31}P NMR (162 MHz, $CDCl_3$) of the crude reaction mixture of (**14i**)

3. References:

1- Zhou, J. J.; Meng, Y. N.; Liu, L. G.; Liu, Y. X.; Xu, Z.; Lu, X.; Zhou, B.; Ye, L. W. Copper-catalyzed enantioselective diyne cyclization via C(sp2)–O bond cleavage. *Chem. Sci.*, **2023**, *14*, 3493-3500.

2- Kos, P.; Savka, R.; Plenio, H.; Fast Olefin Metathesis: Synthesis of 2-Aryloxy-SubstitutedHoveyda-Type Complexes and Application in Ring-Closing Metathesis. *Adv. Synth. Catal.* **2013**, *355*, 439 – 447.

3- Matsumoto, T.; Urano, Y.; Takahashi, Y.; Mori, Y.; Terai, T.; Nagano, T. In Situ Evaluation of Kinetic Resolution Catalysts for Nitroaldol by Rationally Designed Fluorescence Probe. *J. Org. Chem.* **2011**, *76*, 3616–3625.

4- Sui, G.; Lv, Q.; Song, X.; Guo, H.; Dai, J.; Ren, L.; Lee, C. S.; Zhou, W.; Hao, H. D. Chemoselective Reduction of Aldehydes: Via a Combination of $NaBH_4$ and Acetylacetone. *New J. Chem.* **2019**, *43*, 15793–15796.

5- Adamek, J.; Kuznik, A.; Pazdzierniok-Holewa, A.; Grymel, M.; Kozicka, D.; Mierzwa, D.; Erfurt, K. 1-Hydroxyalkylphosphonium Salts-Synthesis and Properties. *Molecules* **2024**, *29*, 18.

6- Babu, K. N.; Massarwe, F.; Shioukhi, I.; Masarwa, A. Sequential Selective C−H and C(Sp3)−+P Bond Functionalizations: An Entry to Bioactive Arylated Scaffolds. *Angew. Chem. Int. Ed.* **2021**, *60*, 26199–26209.

7- Hazra, G.; Masarwa, A. Synthesis and Functionalization of Thiophosphonium Salts: A Divergent Approach to Access Thioether, Thioester, and Dithioester Derivatives. *Org. Lett.* **2023**, *25*, 6396–6400.

8- Guo, M. M.; Qin, G. Q.; Jiang, X. Y.; Xu, H.; Ma, M.; Shen, Z. L.; Chu, X. Q. Aerobic Coupling of Organophosphonium Salts with Alkenes: Catalyst-Free C(Sp3)−C(Sp2) Bond Formation. *Adv. Synth. Catal.* **2023**, *365*, 1871–1876.

9- Maity, P.; Shacklady-Mcatee, D. M.; Yap, G. P. A.; Sirianni, E. R.; Watson, M. P. Nickel-Catalyzed Cross Couplings of Benzylic Ammonium Salts and Boronic Acids: Stereospecific Formation of Diarylethanes via C-N Bond Activation. *J. Am. Chem. Soc.* **2013**, *135*, 280–285.

10- Böβ, E.; Hillringhaus, T.; Nitsch, J.; Klussmann, M. Lewis Acid-Catalysed One Pot Synthesis of Substituted Xanthenes. *Org. Biomol. Chem.* **2011**, *9*, 1744.

11- Bedford, R. B.; Huwe, M.; Wilkinson, M. C. Iron-Catalysed Negishi Coupling of Benzylhalides and Phosphates. *Chem. Commun.* **2009**, 600–602.

12- Yurino, T.; Hachiya, A.; Suzuki, K.; Ohkuma, T. Selective Conversion of Benzylic Phosphates into Diarylmethanes Through Al(OTf) 3 -Catalyzed Friedel–Crafts-Type Benzylation. *European J. Org. Chem.* **2020**, *2020*, 2225–2232.

13- Yurino, T.; Hachiya, A.; Suzuki, K.; Ohkuma, T. Selective Conversion of Benzylic Phosphates into Diarylmethanes Through Al(OTf) 3 -Catalyzed Friedel–Crafts-Type Benzylation. *European J. Org. Chem.* **2020**, *2020*, 2225–2232.

14- Shi, J.; Yuan, T.; Wang, R.; Zheng, M.; Wang, X. Boron Carbonitride Photocatalysts for Direct Decarboxylation: The Construction of C(Sp $_3$)–N or C(Sp $_3$)–C(Sp $_2$) Bonds with Visible Light. *Green Chem.* **2021**, *23*, 3945–3949.

15- Wu, L.; Jiang, R.; Yang, J.-M.; Wang, S.-Y.; Ji, S.-J. In(OTf)$_3$ Catalyzed C3-Benzylation of Indoles with Benzyl Alcohols in Water. *RSC Adv.* **2013**, *3*, 5459.

16- Alacid, E.; Nájera, C. First Cross-Coupling Reaction of Potassium Aryltrifluoroborates with Organic Chlorides in Aqueous Media Catalyzed by an Oxime-Derived Palladacycle. *Org. Lett.* **2008**, *10*, 5011–5014.

17- Zhang, J.; Lu, G.; Xu, J.; Sun, H.; Shen, Q. Nickel-Catalyzed Reductive Cross-Coupling of Benzyl Chlorides with Aryl Chlorides/Fluorides: A One-Pot Synthesis of Diarylmethanes. *Org. Lett.* **2016**, *18*, 2860–2863.

18- Chandrasekhar, S.; Khatun, S.; Rajesh, G.; Reddy, C. R. B (C $_6$ F $_5$) $_3$: An Efficient Catalyst for Reductive Alkylation of Alkoxy Benzenes and for Synthesis of Triarylmethanes Using Aldehydes. *Tetrahedron Lett.* **2009**, *50*, 6693–6697.

19- Fan, S.; He, C.-Y.; Zhang, X. Direct Pd-Catalyzed Benzylation of Highly Electron-Deficient Perfluoroarenes. *Chem. Commun.* **2010**, *46*, 4926.

20- Cheng, X.; Wang, L.; Liu, Y.; Wan, X.; Xiang, Z.; Li, R. Molecular Iodine-Catalysed Reductive Alkylation of Indoles : Late-Stage Diversification for Bioactive Molecules. *Eur. J. Org. Chem.* **2022**, *2022*, e202200502.

21- Rashid, A.; Lone, W. I.; Dogra, P.; Rashid, S.; Bhat, B. A. HFIP-Mediated C-3-Alkylation of Indoles and Synthesis of Indolo[2,3-b]Quinolines & Related Natural Products†. *Org. Biomol. Chem* **2024**, *22*, 3502–3509.

22- Gui, R.; Li, C.-J. Regiospecific Deoxygenative Deuteration of Ketones via HOME Chemistry. Org. Chem. Front. **2023**, *10*, 1767–1772.

23- Jereb, M.; Vražič, D. Iodine-Catalyzed Disproportionation of Aryl-Substituted Ethers under Solvent-Free Reaction Conditions. *Org. Biomol. Chem.* **2013**, *11*, 1978.

24- Zhang, J.; Wang, Z.; Wang, Y.; Wan, C.; Zheng, X.; Wang, Z. A Metal-Free Catalytic System for

the Oxidation of Benzylic Methylenes and Primary Amines under Solvent-Free Conditions. *Green Chem.* **2009**, *11*, 1973.

25- Gautam, P.;,Bhanage, B. M. Palladacycle-Catalyzed Carbonylative Suzuki−Miyaura Coupling with High Turnover Number and Turnover Frequency. *J. Org. Chem.* **2015**, *80*, 7810−7815.

26- Li, H.; Xu, Y.; Shi, E.; Wei, W.; Suo, X.; Wan, X. Synthesis of Arylketones by Ruthenium-Catalyzed Cross-Coupling of Aldehydes with Arylboronic Acids. *Chem. Commun.* **2011**, *47*, 7880.

27- Vekariya, R. H.; Aubé, J. Hexafluoro-2-Propanol-Promoted Intermolecular Friedel–Crafts Acylation Reaction. *Org. Lett.* **2016**, *18*, 3534–3537.

28- O'Keefe, B. M.; Simmons, N.; Martin, S. F. Carbonylative Cross-Coupling of Ortho-Disubstituted Aryl Iodides. Convenient Synthesis of Sterically Hindered Aryl Ketones. *Org. Lett.* **2008**, *10*, 5301–5304.

29- Gu, L.; Jin, C.; Liu, J.; Zhang, H.; Yuan, M.; Li, G. Acylation of Indoles via Photoredox Catalysis: A Route to 3-Acylindoles. *Green Chem.* **2016**, *18*, 1201–1205.

30- Wertz, S.; Leifert, D.; Studer A. Cross Dehydrogenative Coupling via Base-Promoted Homolytic Aromatic Substitution (BHAS): Synthesis of Fluorenones and Xanthones *Org. Lett.* **2013**, *15*, 4, 928–931.
